# A systematic review and meta-analysis of circulating 25-hydroxyvitamin D concentration and vitamin D status worldwide

**DOI:** 10.1093/pubmed/fdaf080

**Published:** 2025-07-13

**Authors:** Eleanor Dunlop, Ngoc Minh Pham, Dong Van Hoang, Hajar Mazahery, Belinda Neo, Jillian Shrapnel, Aliki Kalmpourtzidou, Kevin E K Chai, Leo Ng, Lucinda J Black

**Affiliations:** Institute for Physical Activity and Nutrition (IPAN), School of Exercise and Nutrition Sciences, Deakin University, Gheringhap Street, Geelong, VIC 3220, Australia; Curtin School of Population Health, Curtin University, Kent Street, Bentley, WA 6102, Australia; Curtin School of Population Health, Curtin University, Kent Street, Bentley, WA 6102, Australia; Curtin School of Population Health, Curtin University, Kent Street, Bentley, WA 6102, Australia; Curtin School of Population Health, Curtin University, Kent Street, Bentley, WA 6102, Australia; Curtin School of Population Health, Curtin University, Kent Street, Bentley, WA 6102, Australia; Curtin School of Population Health, Curtin University, Kent Street, Bentley, WA 6102, Australia; Department of Sustainable Food Process, Università Cattolica Del Sacro Cuore, Via Stefano Leonida Bissolati Cremona, CR 26100, Italy; Curtin School of Population Health, Curtin University, Kent Street, Bentley, WA 6102, Australia; Curtin School of Population Health, Curtin University, Kent Street, Bentley, WA 6102, Australia; School of Health Sciences, Swinburne University of Technology, John Street, Hawthorn, VIC 3122, Australia; Institute for Physical Activity and Nutrition (IPAN), School of Exercise and Nutrition Sciences, Deakin University, Gheringhap Street, Geelong, VIC 3220, Australia; Curtin School of Population Health, Curtin University, Kent Street, Bentley, WA 6102, Australia

**Keywords:** 25-hydroxyvitamin D, vitamin D status

## Abstract

**Background:**

Vitamin D deficiency is a public health concern; however, data on its global prevalence are limited. We reported pooled mean circulating 25-hydroxyvitamin D (25(OH)D) concentration and estimated the prevalence of concentration according to commonly reported thresholds for general, healthy populations worldwide.

**Methods:**

We searched MEDLINE, Embase, Web of Science, Global Index Medicus and grey literature sites. We included cross-sectional and cohort studies published since 2011 that reported circulating 25(OH)D concentration in general, healthy populations of all ages. Using random-effects meta-analysis, we pooled data on circulating 25(OH)D concentration and prevalence estimates according to thresholds by continent, country, latitude, sex, adults/children, season, assay, and study quality. PROSPERO registration: CRD42021242466.

**Results:**

Eligible publications (*n* = 586) included 2 370 136 eligible participants across 102 countries. The pooled mean (95% confidence interval [CI]) overall circulating 25(OH)D concentration was 53.9 (52.6–55.1) nmol/L (529 publications; 1 412 281 participants). The pooled prevalence of concentration < 30, < 50, and < 75 nmol/L was 18, 47, and 75%, respectively (403 studies; 1 508 830 participants).

**Conclusions:**

Low vitamin D status is prevalent across general, healthy populations worldwide. Governments, health organizations and policy makers could use these findings to identify regions in need of public health strategies for improving vitamin D status.

## Introduction

Vitamin D is vital for healthy bones; deficiency may lead to osteomalacia or rickets. Vitamin D may also have a beneficial role in Alzheimer’s disease, hypertension, schizophrenia, type 2 diabetes, irritable bowel syndrome, and all-cause mortality.^1–3^ Vitamin D deficiency places a considerable burden on individuals, services, and economies.^4^ Cost-effective public health programmes, such as fortification of staple foods, and/or supplementation programmes for at-risk subpopulations (e.g. infants, the elderly, pregnant, or lactating women) are already used in some countries to promote vitamin D sufficiency.^4^

Vitamin D status is commonly assessed by circulating 25-hydroxyvitamin D (25(OH)D) concentration. Earlier 25(OH)D assays have produced highly variable results, leading to under- and overestimation of the prevalence of vitamin D deficiency in national surveys.^5^ Whilst issues of bias remain,^5^ a greater proportion of studies now use 25(OH)D assays certified to the reference measurement procedures (RMPs) developed under the Vitamin D Standardization Programme (VDSP) or retrospectively harmonized data.^6^^,^^7^

Population-level data on vitamin D status based on modern analytical methods are needed to determine where public health strategies could be implemented or optimized. Earlier systematic reviews relating to global vitamin D status are outdated, did not focus on data that represent overall populations, or did not report pooled mean circulating 25(OH)D concentration.^8^^,^^9^ A previous systematic review and meta-analysis included studies conducted in general populations and published from 1990 to 2011, reporting pooled mean circulating 25(OH)D concentration and the proportion of mean values < 25 and < 50 nmol/L.^9^ We conducted an updated and improved systematic review and meta-analysis of studies reporting mean circulating 25(OH)D concentration and/or true prevalence of 25(OH)D concentration according to thresholds (<30, < 50, < 75 nmol/L) that are widely used as cut-points for vitamin D deficiency, insufficiency, and sufficiency^10^^,^^11^ in general populations and cohorts of healthy study participants globally.

## Methods

This study was conducted and reported according to the PRISMA guidelines.^12^ The protocol^13^ was registered with PROSPERO: registration number CRD42021242466. Following the initial registration in April 2021, the protocol was updated to include searches of five databases rather than one, data extraction by two reviewers rather than one, and inclusion of newborns, overweight/obese individuals and pregnant and menopausal women. Those populations were subsequently excluded to balance quality with feasibility and provide targeted data representative of general, healthy populations. Neither patients nor members of the public were involved in the design, conduct, reporting, or dissemination plans of this research.

### Literature search and study selection

With a health sciences faculty librarian, we developed search strategies for Ovid MEDLINE (Table 1), Ovid Embase, Scopus, Web of Science, and Global Index Medicus (Supplementary Table 1). Search strategies were designed with minimal use of ‘NOT’ statements to maximize capture of relevant articles; except for animal studies, any ‘NOT’ statements were limited to title and keyword fields. On 16 August 2022, we searched for studies that were published from 1 March 2011, following on from the census date of an earlier meta-analysis.^9^ Searches were repeated on 16 June 2023.

**Table 1:** Example search strategy.

Medline (Ovid)	
1	(‘vitamin D’ or ‘vitamin D3’ or ‘25-hydroxyvitamin D’ or 25-hydroxyvitamin D3” or ‘25(OH)D’ or ‘25(OH)D3’ or ‘calcidiol’).ti,ab,kw,sh.
2	(‘dihydroxycholecalciferol’ or ‘dihydroxycholecalciferols’ or ‘case reports’ or ‘case series’ or ‘case report’ or ‘case control’ or ‘case–control’ or ‘clinical trial’ or ‘controlled trial’ or ‘review’ or ‘systematic review’ or ‘systematic-review’ or experiment or experimental or in-vivo or in-vitro or ‘in vivo’ or ‘in vitro’ or mechanism or cells).sh,ti,kw.
3	Exp animals/not humans.sh.
4	1 not 2
5	4 not 3
6	Limit 5 to dt = 20 110 301–20 220 920
7	Limit 6 to English language
8	Limit 7 to journal article

Studies that reported mean or median 25(OH)D concentration in groups of children and/or adults representative of general or healthy populations were included. Case–control, case reports, case series and intervention studies were excluded, along with studies that used convenience sampling, to avoid related bias. Studies were excluded if they focused on: neonates (aged < 28 days); people with health conditions included in the International Classification of Diseases,^14^ including overweight, obesity and pregnancy; institutionalized people (except residents of aged care facilities); people with a specific occupational, lifestyle (e.g. parents, elite athletes) or racial/ethnic trait. Studies were excluded if all blood draws occurred prior to 2002 to capture data representative of increasingly indoor-based lifestyles^15^ and assays used over the preceding two decades; studies that commenced prior were included if blood draw continued into 2002.

As we anticipated a large number of search results, a two-step initial screening process was conducted to allow manual removal of irrelevant articles using keywords that would otherwise have been used in a ‘NOT’ statement. Following automated deduplication of retrieved articles in R version 4.3.1 (R Core Team, Vienna, Austria), abstracts were loaded into the validated tool, Research Screener,^16^ along with 12 ‘seed’ (exemplar) articles. Research Screener uses machine learning to continually rank, re-rank and present abstracts for screening in order of relevance based on the content of the ‘seed’ articles. The loop process continues until all abstracts have been reviewed, or the 50% evidence-based threshold at which the tool has been validated to have presented 100% of relevant articles.^16^ Two authors (ED, DVH) independently screened 3000 titles and abstracts using Research Screener. During this process, common keywords (‘patients’, ‘randomi^*^’, ‘meta-analysis’, ‘controls’, ‘rat’, ‘mice’, ‘pig’) amongst irrelevant abstracts were noted. The remaining file of unscreened abstracts was manually screened for those keywords and irrelevant articles removed. The remaining abstracts were reloaded into Research Screener for a second phase of independent title and abstract screening by ED and DVH, which was continued beyond the 50% threshold until 10 consecutive rounds (500 abstracts) with zero relevant records was achieved.

A final screen was conducted (ED, JS) to exclude studies that focused on people with specific characteristics (pregnancy, occupation, ethnic subpopulation). ED and JS independently reviewed full texts and manually screened the bibliographies of included articles.

Records identified in the June 2023 search were deduplicated in EndNote 20 and uploaded to Research Screener with the original 12 seed articles. Titles and abstracts were independently screened by two authors (ED, JS, or NMP). From a pool of five authors (ED, NMP, JS, HM, BN), full texts were screened independently by two authors.

Where multiple articles reported on the same study population, that with the most recent and comprehensive 25(OH)D data was retained. For recurring national surveys and longitudinal studies, the most recent data were included.

### Data extraction

From a pool of six authors (ED, NMP, JS, HM, BN, AK), data were extracted independently by two authors using a predefined, standardized Microsoft Access form. Data were extracted for 25(OH)D concentration (mean/median with measures of variance and prevalence of concentration < 30, < 50, and < 75 nmol/L) and study and participant characteristics: country, continent, sample size/s, sex, age, year, study design, season of blood draw (classified as winter/spring summer/autumn), assay and use of a certified assay or harmonized data. Any missing data were requested from authors. Data on latitude, ultraviolet (UV) index^17^ and World Bank income classifications by country were merged into our dataset. From a pool of five authors (ED, NMP, JS, HM, BN), study quality was assessed independently by two authors using a tool for assessing risk of bias in prevalence studies.^18^ Any inconsistencies in study selection or data extraction were resolved through discussion.

### Statistical analysis

Statistical analyses were conducted using STATA version 18.0.^19^ Descriptive statistics were used to present characteristics of included studies, including mean (standard deviation [SD]) for normally distributed data or median (interquartile range for non-normally distributed data, or frequency (%) for categorical data. Mean and SD were calculated where necessary as: mean = (minimum +2^*^median + maximum)/4 or mean = (25^th^ percentile + median + 75^th^ percentile)/3; SD = (75^th^ percentile-25^th^ percentile)/1.35) or SD = (√n ^*^95% confidence interval [95%CI])/3.92).^20^^,^^21^ If parameters were neither reported nor made available upon request, studies were excluded to avoid imputing values for non-random missing data. We converted 25(OH)D concentration data from ng/mL (×2.496^22^) to nmol/L when necessary. Various cut points for 25(OH)D concentration were used across articles; we grouped these by < 30, < 50 and < 75 nmol/L.^10^^,^^11^

We conducted three types of meta-analyses, namely: (i) a meta-analysis of the means of circulating 25(OH)D concentration and prevalence of 25(OH)D concentration by the thresholds of < 30 nmol/L, < 50 nmol/L, and <75 nmol/L, in order to obtain overall pooled estimates for all studies and by subgroups. This was achieved using a fixed-effect model, which is suitable when there is no significant level of heterogeneity (*I*^2^ statistic < 50%) between studies, such as those conducted in the same country;

(ii) a meta-analysis of the same data for the calculation of overall summary estimates for circulating 25(OH)D concentration and to conduct sub-group analyses by country, region and other characteristics. This was achieved using a random effect model for meta-analysis, as previous systematic reviews^8^^,^^9^ identified inherent heterogeneity between studies conducted across different populations; and (iii) a meta-analysis of all relevant data from nationally representative surveys only, providing an estimate of the overall prevalence of circulating 25(OH)D concentrations < 30 and < 50 nmol/L and by region, country and sex.

Heterogeneity across studies or regions was assessed in Forest plots, using Cochran’s Q test for statistical significance, and the *I*^2^ index to indicate low (25%), moderate (50%), and high (75%) levels of heterogeneity.^23^ Meta-regression analyses^24^ were conducted by sex^8^^,^^25^^,^^26^ to examine how multiple study-level characteristics (region, latitude, certified/non-certified assay, study design, adults/children, study quality, and country income classification) influence the single effect size.

Cumulative meta-analysis was undertaken, using the year of blood draw (mid-point for studies with multiple years of data collection) and stratified by the mid-point of all blood draw years, to describe temporal trends in pooled estimates of mean circulating 25(OH)D concentration. Cumulative meta-analysis shows how the overall estimate changes as each study is added to the pool. Publication bias was qualitatively detected with funnel plots; asymmetry was examined using Egger’s test.^27^ A 2-sided *P* < 0.05 was considered statistically significant.

## Results

**Figure 1:**
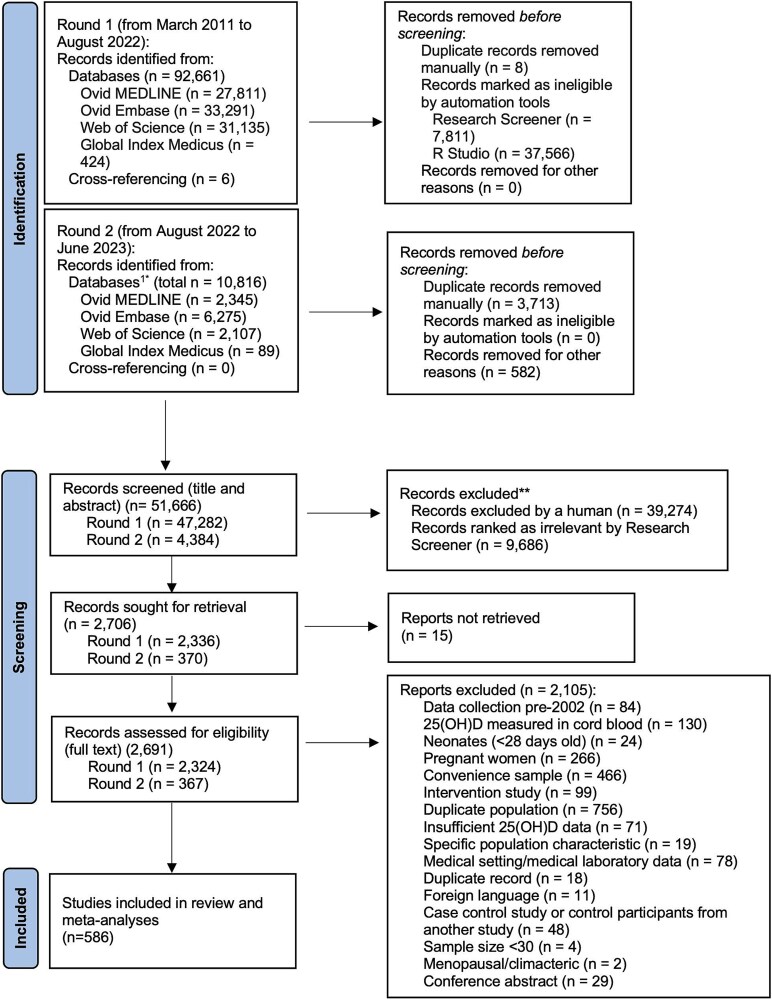
PRISMA flow diagram.

Searches conducted in August 2022 returned 92 661 results, with 47 282 publications remaining following deduplication and 2324 remaining following title and abstract screening (Fig. 1). Searches conducted in June 2023 returned a further 10 816 results, with 4384 publications remaining following deduplication and 367 remaining following title and abstract screening. Of the publications identified from both searches, we excluded 2105 ineligible publications during full-text screening and included six studies from bibliography screens (Fig. 1). A total of 586 eligible publications were included (Supplementary Table 2).

Studies reporting 25(OH)D concentration or vitamin D status by thresholds were conducted across six continents and 102 countries, including 2 370 136 eligible participants aged from 0 to 116 years. The 25(OH)D assay used was reported in 559 publications; 77 studies reported using a certified assay or retrospectively harmonized data.^6^^,^^7^ Study quality scores ranged from 4 to 10 (Supplementary Table 3).^18^ Publication bias was evident (*P* < 0.001) (Supplementary Fig. 1).

**Figure 2:**
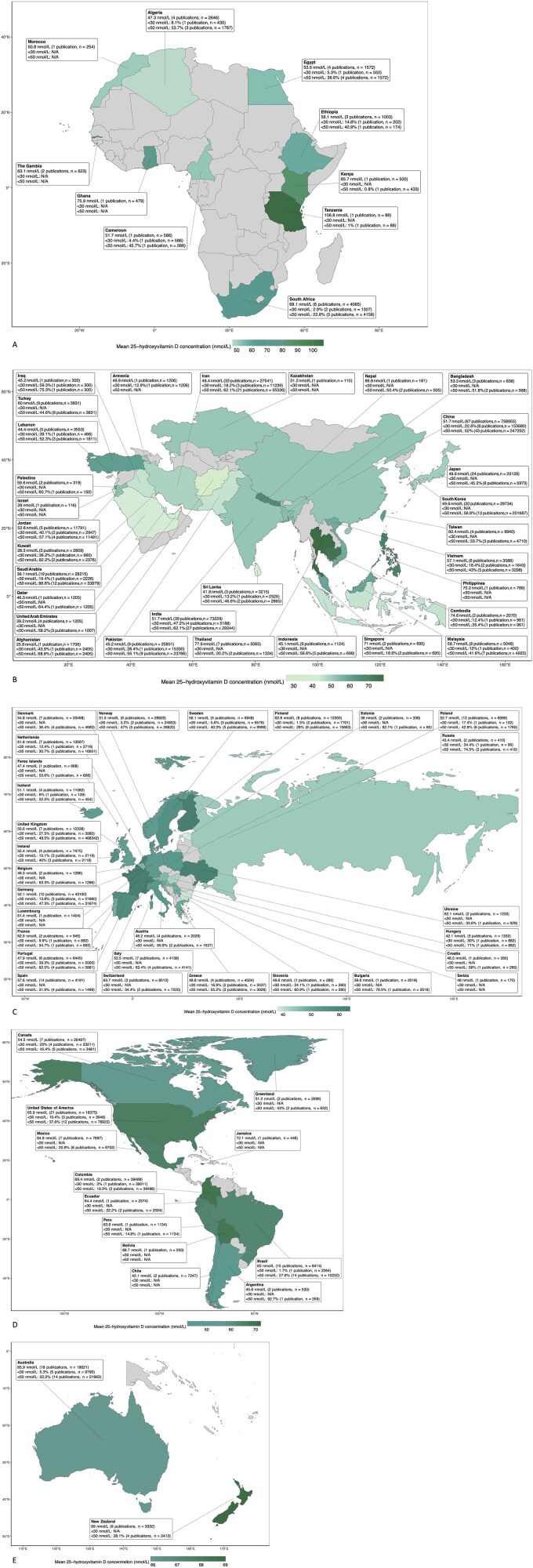
Maps of pooled data for (A) Africa, (B) Asia (C) Europe, (D) Americas (E) Oceania. N/a: Data not available.

Mean or median 25(OH)D concentration was reported in 529 publications conducted across six continents and 87 countries (Table 2, Fig. 2, Supplementary Tables 4–5, Supplementary Figs. 2–13). The overall pooled mean (95% CI) 25(OH)D concentration was 53.9 (52.6; 55.1) nmol/L (Table 2), and 8 and 35% of studies reported mean concentration < 30 and < 50 nmol/L, respectively. The pooled mean 25(OH)D concentration was ~ 6 nmol/L higher for men than women and slightly higher for adults than children. The pooled mean concentration was ~9 nmol/L higher during summer/autumn than winter/spring and ~6 nmol/L higher for upper-middle-income than low-income countries (Supplementary Table 5). Heterogeneity was consistently high (*I*^2^ *>* 90% in most cases) (Table 2 and Supplementary Materials). The cumulative mean circulating 25(OH)D concentration for studies with mid-point blood draw 2012 onwards was ~4 nmol/L significantly lower than for those with mid-point blood draw pre-2012 (*P* = 0.025). Whilst there was no significant change in vitamin D concentration during/after 2012 (regression coefficient = 0.12; *P* for trend = 0.714), a significant decreasing trend was observed in studies with blood draw pre-2012 (regression coefficient = −0.39; *P* for trend <0.001).

**Table 2:** Mean circulating 25-hydroxyvitamin D concentration (nmol/L) by region, sex, and age group.

	n (*publications*)	n (*data points*)	n (*countries*)	n *(participants)*	Mean 25(OH)D (nmol/L)	95% CI	*I^2^ (%)*	
All regions	529	554	87	1 412 281	53.89	52.64; 55.14	100.0	
Male	226	231	72	209 403	56.48	55.30; 57.65	100.0	
Female	273	276	73	269 143	50.91	49.75; 52.08	100.0	
Adults	327	331	68	1 044 021	54.73	53.02; 56.42	100.0	
Children	180	190	62	379 642	53.48	50.30; 56.66	100.0	
Africa	24	27	12	16 783	62.24	53.92; 70.55	99.9	
Male	11	12	9	4519	73.92	64.82; 83.01	99.5	
Female	13	13	9	5819	65.45	56.10; 74.80	99.7	
Adults	12	12	8	4529	60.84	51,75; 69.92	99.7	
Children	11	12	6	7757	67.11	55.20; 79.02	99.9	
Asia	286	302	30	1 027 681	50.67	48.99; 52.36	100.0	
Male	129	129	24	136 841	53.58	52.41; 54.76	100.0	
Female	164	164	26	194 098	45.98	44.69; 47.28	100.0	
Adults	171	173	21	770 176	53.08	50.12; 56.05	100.0	
Children	100	108	26	277 451	47.64	43.34; 51.94	100.0	
Europe	137	141	31	230 467	53.51	51.73; 55.29	99.9	
Male	51	54	28	39 666	55.04	51.59; 58.50	99.6	
Female	59	62	26	43 663	52.86	50.88; 54.83	99.4	
Adults	100	101	30	192 432	52.84	50.62; 55.05	99.9	
Children	34	35	18	26 621	55.93	52.89; 58.97	99.5	
North America	36	37	4	56 161	62.31	57.30; 67.32	100.0	
Male	13	13	4	14 764	62.24	56.34; 68.15	100.0	
Female	16	16	5	16 305	64.22	61.88; 66.56	99.9	
Adults	21	21	3	41 255	66.70	59,91; 73.50	100.0	
Children	14	14	4	25 913	63.99	58.66; 69.32	99.5	
South America	25	25	8	59 836	61.13	55.62; 66.63	99.9	
Male	10	11	5	6039	59.48	49.63; 69.34	99.9	
Female	11	11	5	5906	59.45	51.96; 66.94	99.9	
Adults	9	9	4	19 131	55.10	50.04; 60.17	99.4	
Children	15	15	6	39 104	62.20	54.56; 69.84	99.9	
Oceania	24	24	2	21 353	66.75	56.99; 76.50	99.9	
Male	12	12	2	7574	68.98	64.25; 73.71	98.5	
Female	10	10	2	3640	68.99	63.26; 74.71	98.2	
Adults	15	15	2	16 498	65.00	50.92; 79.09	99.9	
Children	6	6	2	2796	70.30	63.57; 77.02	97.9	

25(OH)D, 25-hydroxyvitamin D; CI, confidence interval.

Multivariate meta-regression analysis showed that, for men, the mean circulating 25(OH)D concentration was significantly higher for Africa and Oceania than Asia. For women, the mean circulating 25(OH)D concentration was higher for Africa, North and South America and Oceania than Asia, and in children than adults (Supplementary Tables 10–13). As a continuous variable, latitude was negatively associated with mean 25(OH)D concentration in simple meta-regression (Supplementary Fig. 6a–c).

**Figure 3:**
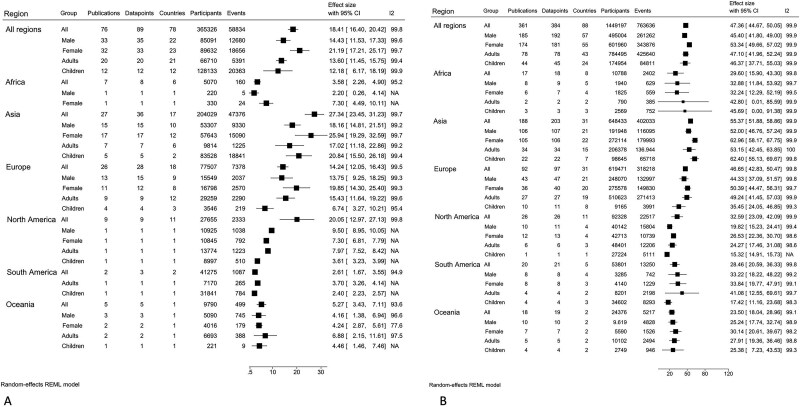
Prevalence of 25-hydroxyvitamin D concentration by region (A) < 30 and (B) < 50 nmol/L.

Circulating 25(OH)D concentration was categorized by thresholds in 403 publications across 92 countries (Figs 2 and 3, Supplementary Tables 6–9, Supplementary Figs 2 and 14–21). The overall pooled prevalence of circulating 25(OH)D concentration < 30, < 50 and < 75 nmol/L was 18, 47, and 75%, respectively (Fig. 3). The pooled prevalence of 25(OH)D concentration below each threshold was consistently higher in women than men. The pooled prevalence of circulating 25(OH)D concentration < 30 and < 50 nmol/L was marginally higher in adults than children, at low latitude than high latitude, in winter/spring than summer/autumn, when a certified assay/standardized data was not used, when study quality was lower and in low-income countries compared to higher-income countries. Using data from nationally representative surveys only (Supplementary File 2; Supplementary Fig. 22a–h), the pooled prevalence of circulating 25(OH)D concentration < 30 (20%) and < 50 (49%) nmol/L was marginally higher.

## Discussion

### Main finding of this study

This study has provided population-representative data on mean circulating 25(OH)D concentration and prevalence of circulating 25(OH)D concentration < 30, < 50, and < 75 nmol/L in general, healthy, populations, using the largest number of studies to date. Our findings suggest that low vitamin D status is prevalent in many regions worldwide. The data generated indicate regions and population characteristics that warrant comprehensive analysis of environmental, behavioural and policy contexts in order that suitable public health strategies for improving vitamin D status may be developed if needed. The prevalence of circulating 25(OH)D concentration < 30 nmol/L, was higher in women than men, at latitude <40^o^ compared to latitude ≥40^o^, and in low-income countries compared to higher-income countries.

### What is already known on this topic

Other systematic reviews and meta-analyses of vitamin D status have shown a higher prevalence of low vitamin D status in women compared to men,^8^^,^^25^^,^^26^ whilst another did not.^9^ Higher prevalence of 25(OH)D concentration < 30 nmol/L at latitude 20-40^o^ N compared to other latitudes was shown in a recent meta-analysis of data from 308 studies.^8^ That meta-analysis showed that lower-middle-income countries had the highest prevalence of 25(OH)D concentration < 30 nmol/L based on data from one low-income and nine lower-middle-income countries;^8^ our study included two low-income and 12 lower-middle-income countries.

Potential sex-specific influences on circulating 25(OH)D concentration, such as sex hormone levels, have been proposed; however, inconsistencies in 25(OH)D concentration between sexes at the individual study level suggest that lifestyle factors, which may be culture-specific, play a key role.^28^ Latitude relates to the influence of sun exposure on circulating 25(OH)D concentration, but may not necessarily predict vitamin D status alone.^26^ Further, whilst low vitamin D intake and status have been shown to be prevalent across lower- and higher-income countries, there has been disparity in reporting between the two.^29^ This was evident in our meta-analysis, with estimated prevalence of concentration < 30 nmol/L based on 19 publications for low/low-middle-income countries versus 58 publications for upper-middle/high-income countries. It could be helpful for funding providers to prioritize support of collection of high-quality data in underrepresented regions, such as low-income countries.

Other environmental and lifestyle factors contribute to vitamin D status, including dietary preferences and behaviours (including supplement use), the dietary supply of vitamin D (including availability of fortified foods), genetic differences in vitamin D metabolism, religious and cultural practices—particularly related to clothing—and opportunity to generate vitamin D via sun exposure, which may relate to latitude, season, skin pigmentation, outdoor physical activity, sun protective behaviours and occupation.^29^ It is important that region-specific strategies to support vitamin D sufficiency are tailored to population- and region-specific characteristics such as economic situation, latitude and climate.

### What this study adds

Our study updates a previous systematic review and meta-analysis^9^ with a more comprehensive literature search of five databases, compared to two in the previous study. In that previous study, 37% of studies reported mean concentration <50 nmol/L.^9^ Using that method, our findings were similar at 35%. However, that method derives prevalence from mean concentration, which is expected to be derived from some values below, and some values above, the mean, and does not reflect the true proportion of the population with concentration < 50 nmol/L. When we used true prevalence data, our estimate of overall mean concentration <50 nmol/L was higher (47%). We further built on that earlier study with cumulative meta-analysis, showing an apparent temporal reduction in mean circulating 25(OH)D concentration. Whilst assays for 25(OH)D have generally improved, the ongoing variation in data generated by non-certified assays may contribute to, or mask, any true temporal change.

Other systematic reviews and meta-analyses^8^^,^^9^ have reported pooled mean circulating 25(OH)D concentration and/or the prevalence of concentration by thresholds. Our findings largely support those from a meta-analysis that indicated global prevalence of circulating 25(OH)D concentration at < 30, 50, and 75 nmol/L as 15.7, 47.9, and 76.6%, respectively.^8^ The authors of that study used a keyword-restricted search strategy and included data collected from health service databases, whilst we did not.

The major strength of our study was the use of a broad search strategy to avoid inadvertent loss of eligible studies. This resulted in a pooled sample of data from the largest number of studies on mean circulating 25(OH)D concentration and prevalence of concentration < 30, < 50 and < 75 nmol/L. We conducted cumulative meta-analysis to explore potential temporal changes and sub-group analyses by continent, country, sex, and by adults and children; however, pooling studies over time does not account for changing study methodologies, population characteristics and improvements in assay technologies.

### Limitations of this study

Our study was hindered by the lack of data on factors that may influence vitamin D status, e.g. sun exposure, skin type and vitamin D intake from food and supplements. We encountered poor reporting in some studies, including lack of information on sampling methods and sample collection timeframes, addressed, in part, by sub-group analysis by study quality score. Whilst we endeavoured to avoid including duplicated study populations, we could not verify that there were no overlapping study populations across separate studies. There was evidence of publication bias, heterogeneity was consistently high, and in some meta-analyses there was wide dispersion around average pooled estimates. Due to high variability and heterogeneity, the comparability of included studies may be limited, e.g. due to differing socioeconomic and environmental contexts; hence the results obtained from the pooled analysis should be interpreted with caution. Future studies could explore any differences between climatic regions (e.g. tropical versus temperate). Further systematic reviews and meta-analyses are waranted to assess circulating 25(OH)D concentration and vitamin D status in specific populations (e.g. pregnant and overweight/obese individuals) that are important to consider in public health policy and interventions.

Relatively few studies reported using a certified assay or retrospectively harmonized data. Some studies reported use of a NIST standard reference material, but not that the laboratory undertook regular verification of their assay, as required by certification schemes.^30^ Our overall pooled estimates are subject to any bias that exists in data included from studies that did not use a certified assay or harmonized data. Conducting and publishing studies that use a certified 25(OH)D assay or retrospectively harmonized data^5^ should become an expected standard in vitamin D research, by researchers and publishers alike, as a priority to allow comparability, accuracy and reliability in future research. It is hoped that the proportion of published studies using standardized 25(OH)D measures has, and will continue to, steadily increase since the updated search for this review. It will be important to conduct updated meta-analyses as this trend occurs.

There was considerable variation in the cut-points used to report prevalence of concentration according to thresholds across included publications. The inability to categorize all data within a standard set of thresholds limited the sample size for analyses by cut-points. International consensus on thresholds to be used for vitamin D status could lead to a standardized approach to reporting and maximize data pooling potential. The exclusive use of standardized 25(OH)D measurements could help in developing sufficient data to support such consensus.

## Conclusion

This systematic review and meta-analysis demonstrate that low vitamin D status remains prevalent in general populations worldwide. Governments, health organizations and policy makers could use the data generated through this study to identify regions in need of public health strategies for improving vitamin D status.

## Supplementary Material

Dunlop-et-al-Global-vitamin-D-status-Supplementary-file-1_fdaf080

Dunlop-et-al-Global-vitamin-D-status-Supplementary-file-2_fdaf080

## Data Availability

The data underlying this article will be shared on reasonable request to the corresponding author.

## Notes

*Conflict of interest*: Authors worked with dsm-firmenich to develop the scope and key questions of this review. Authors wrote the protocol and developed search strategies, which were approved by dsm-firmenich. Dsm-firmenich had no role in the conduct of the study (study selection, quality assessment, data extraction or management, analysis, interpretation of the data), or preparation, review or approval of the findings. At the time of submission, KEKC and LN are the developers of the Research Screener software and receive financial remuneration to maintain the hosting platform and its related requirements. The authors declare no other competing interests.

## Notes

**Eleanor Dunlop,** Research Fellow

**Ngoc Minh Pham,** Lecturer

**Dong Van Hoang,** Research Fellow

**Hajar Mazahery,** Research Fellow

**Belinda Neo,** PhD Student

**Jillian Shrapnel,** Research Associate

**Aliki Kalmpourtzidou,** Nutritionist

**Kevin E.K. Chai,** Lead Data Scientist

**Leo Ng,** Associate Professor

**Lucinda J. Black,** Professor
