# Supplementary material for: A systematic review and meta-analysis of circulating 25-hydroxyvitamin D concentration and vitamin D status worldwide
Source: J Public Health (Oxf). 2025 Jul 13;47(4):e520–9. doi: 10.1093/pubmed/fdaf080 (PMC12670000; doi:10.1093/pubmed/fdaf080)
Supplement: Dunlop-et-al-Global-vitamin-D-status-Supplementary-file-1_fdaf080 [file dunlop-et-al-global-vitamin-d-status-supplementary-file-1_fdaf080.docx]

*Supplementary File 1*

**Title:** A systematic review and meta-analysis of vitamin D status in healthy populations globally

**Authors:** Eleanor Dunlop ^1,2^, Ngoc Minh Pham ^2^, Dong Van Hoang ^2^, Hajar Mazahery ^2^, Belinda Neo ^2^, Jillian Shrapnel ^2^, Aliki Kalmpourtzidou ^3^, Lucinda J Black ^1,2^

**Author affiliations:**

^1^ Deakin University, Geelong, Institute for Physical Activity and Nutrition (IPAN), School of Exercise and Nutrition Sciences. e.dunlop@deakin.edu.au; lucinda.black@deakin.edu.au

^2^ Curtin School of Population Health, Curtin University, Kent Street, Bentley WA 6102, Australia. eleanor.dunlop@curtin.edu.au; minh.n.pham@curtin.edu.au; vandong.hoang@curtin.edu.au; jill.shrapnel@curtin.edu.au; hajar.mazahery@curtin.edu.au; belinda.neo@curtin.edu.au

^3^ Department of Sustainable Food Process, Università Cattolica Del Sacro Cuore, Cremona, Italy. alikikalb@hotmail.com

**Table of contents***

[Supplementary Table 1: Search strategies 5](#_Toc189144727)

[Supplementary Table 2: Study characteristics 7](#_Toc189144728)

[Bibliography* 32](#_Toc189144729)

[Supplementary Table 3: Study quality scores ^a^ 58](#_Toc189144730)

[Supplementary Table 4: Pooled mean circulating 25-hydroxyvitamin D concentration by country 97](#_Toc189144731)

[Supplementary Table 5: Pooled mean circulating 25-hydroxyvitamin D concentration by latitude, season, use of a certified assay/standardised data, study quality and country income classification 99](#_Toc189144732)

[Supplementary Table 6: Prevalence of circulating 25-hydroxyvitamin D concentration <30, <50 and <75 nmol/L by region and sex 100](#_Toc189144733)

[Supplementary Table 7: Prevalence of circulating 25-hydroxyvitamin D concentration <30, <50 and <75 nmol/L by region and adults and children 102](#_Toc189144734)

[Supplementary Table 8: Pooled prevalence according to commonly reported thresholds by country 104](#_Toc189144735)

[Supplementary Table 9: Pooled prevalence estimates according to commonly reported thresholds by latitude, season, use of a certified assay/standardised data, study quality and country income classification 110](#_Toc189144736)

[Supplementary Table 10: Univariate meta-regression analysis mean circulating 25-hydroxyvitamin D concentration: Men 112](#_Toc189144737)

[Supplementary Table 11: Multi-variate meta-regression analysis of mean circulating 25-hydroxyvitamin D concentration: Men 113](#_Toc189144738)

[Supplementary Table 12: Univariate meta-regression analysis mean circulating 25-hydroxyvitamin D concentration: Women 114](#_Toc189144739)

[Supplementary Table 13: Multi-variate meta-regression analysis mean circulating 25-hydroxyvitamin D concentration: Women 115](#_Toc189144740)

[Supplementary Figure 1: Publication bias 116](#_Toc189144741)

[Supplementary Figure 2: World map of pooled mean 25-hydroxyvitamin D concentration (nmol/L) 117](#_Toc189144742)

[Supplementary Figure 3: Forest plot for overall pooled mean circulating 25-hydroxyvitamin D concentration 118](#_Toc189144743)

[Supplementary Figure 4a: Forest plot for pooled mean circulating 25-hydroxyvitamin D concentration by country – Africa 128](#_Toc189144744)

[Supplementary Figure 4b: Forest plot for pooled mean circulating 25-hydroxyvitamin D concentration by country – Asia 129](#_Toc189144745)

[Supplementary Figure 4c: Forest plot for pooled mean circulating 25-hydroxyvitamin D concentration by country – Europe 136](#_Toc189144746)

[Supplementary Figure 4d: Forest plot for pooled mean circulating 25-hydroxyvitamin D concentration by country – North America 140](#_Toc189144747)

[Supplementary Figure 4e: Forest plot for pooled mean circulating 25-hydroxyvitamin D concentration by country – South America 141](#_Toc189144748)

[Supplementary Figure 4f: Forest plot for pooled mean circulating 25-hydroxyvitamin D concentration by country - Oceania 142](#_Toc189144749)

[Supplementary Figure 5: Forest plot for pooled mean circulating 25-hydroxyvitamin D concentration by high (≥40^o^) and low (<40^o^) latitude 143](#_Toc189144750)

[Supplementary Figure 6a: Bubble plot of circulating 25-hydroxyvitamin D concentration by latitude in all participants 151](#_Toc189144751)

[Supplementary Figure 6b: Bubble plot of circulating 25-hydroxyvitamin D concentration by latitude in men 152](#_Toc189144752)

[Supplementary Figure 6c: Bubble plot of circulating 25-hydroxyvitamin D concentration by latitude in women 153](#_Toc189144753)

[Supplementary Figure 7: Forest plot for pooled mean circulating 25-hydroxyvitamin D concentration in men 154](#_Toc189144754)

[Supplementary Figure 8: Forest plot for pooled mean circulating 25-hydroxyvitamin D concentration in women 158](#_Toc189144755)

[Supplementary Figure 9: Forest plot for pooled mean circulating 25-hydroxyvitamin D concentration by adults and children 163](#_Toc189144756)

[Supplementary Figure 10: Forest plot for pooled mean circulating 25-hydroxyvitamin D concentration by season 174](#_Toc189144757)

[Supplementary Figure 11: Forest plot for pooled mean circulating 25-hydroxyvitamin D concentration by use of certified assay/harmonised data 177](#_Toc189144758)

[Supplementary Figure 12: Forest plot for pooled mean circulating 25-hydroxyvitamin D concentration by study quality 185](#_Toc189144759)

[Supplementary Figure 13: Cumulative* meta-analysis of mean circulating 25(OH)D concentration stratified by mid-point (2012) of all blood draw years 194](#_Toc189144760)

[Supplementary Figure 14a: Forest plot for pooled prevalence estimate of circulating 25-hydroxyvitamin D concentration <30 nmol/L according to commonly reported thresholds by continent 204](#_Toc189144761)

[Supplementary Figure 14b: Forest plot for pooled prevalence estimate of circulating 25-hydroxyvitamin D concentration <50 nmol/L according to commonly reported thresholds by continent 207](#_Toc189144762)

[Supplementary Figure 15a: Forest plot for pooled prevalence estimate of circulating 25-hydroxyvitamin D concentration <30 nmol/L by country 215](#_Toc189144763)

[Supplementary Figure 15b: Forest plot for pooled prevalence estimate of circulating 25-hydroxyvitamin D concentration <50 nmol/L by country – Africa 219](#_Toc189144764)

[Supplementary Figure 15c: Forest plot for pooled prevalence estimate of circulating 25-hydroxyvitamin D concentration <50 nmol/L by country – Asia 220](#_Toc189144765)

[Supplementary Figure 15d: Forest plot for pooled prevalence estimate of circulating 25-hydroxyvitamin D concentration <50 nmol/L by country – Europe 226](#_Toc189144766)

[Supplementary Figure 15e: Forest plot for pooled prevalence estimate of circulating 25-hydroxyvitamin D concentration <50 nmol/L by country – North America 230](#_Toc189144767)

[Supplementary Figure 15f: Forest plot for pooled prevalence estimate of circulating 25-hydroxyvitamin D concentration <50 nmol/L by country – South America 231](#_Toc189144768)

[Supplementary Figure 15g: Forest plot for pooled prevalence estimate of circulating 25-hydroxyvitamin D concentration <50 nmol/L by country – Oceania 232](#_Toc189144769)

[Supplementary Figure 16a: Forest plot for prevalence estimates <30 nmol/L by high (≥40^o^) and low (<40^o^) latitude 233](#_Toc189144770)

[Supplementary Figure 16a: Forest plot for prevalence estimates <50 nmol/L by high (≥40^o^) and low (<40^o^) latitude 235](#_Toc189144771)

[Supplementary Figure 17a: Forest plot for prevalence estimates according to commonly reported thresholds by sex_men 243](#_Toc189144772)

[Supplementary Figure 17b: Forest plot for prevalence estimates according to commonly reported thresholds by sex_women 248](#_Toc189144773)

[Supplementary Figure 18a: Forest plot for prevalence estimates <30 nmol/L by adults and children 253](#_Toc189144774)

[Supplementary Figure 18b: Forest plot for prevalence estimates <50 nmol/L by adults and children 254](#_Toc189144775)

[Supplementary Figure 19a: Forest plot for prevalence estimates <30 nmol/L by season 257](#_Toc189144776)

[Supplementary Figure 19b: Forest plot for prevalence estimates <50 nmol/L by season 258](#_Toc189144777)

[Supplementary Figure 20a: Forest plot for prevalence estimates <30 nmol/L by use of certified assay/harmonised data 260](#_Toc189144778)

[Supplementary Figure 20b: Forest plot for prevalence estimates <50 nmol/L by use of certified assay/harmonised data 262](#_Toc189144779)

[Supplementary Figure 21a: Forest plot for prevalence estimates <30 nmol/L by study quality 270](#_Toc189144780)

[Supplementary Figure 21b: Forest plot for prevalence estimates <50 nmol/L by study quality 272](#_Toc189144781)

****Please see Supplementary File 2 for Supplementary Figures 22a-22h***

# Supplementary Table 1: Search strategies

| **Medline (Ovid)** | |
| --- | --- |
| 1 | (“vitamin D” or “vitamin D3” or “25-hydroxyvitamin D” or 25-hydroxyvitamin D3” or “25(OH)D” or “25(OH)D3” or “calcidiol”).ti,ab,kw,sh. |
| 2 | (“dihydroxycholecalciferol” or “dihydroxycholecalciferols” or “case reports” or “case series” or “case report” or “case control” or “case-control” or “clinical trial” or “controlled trial” or “review” or “systematic review” or “systematic-review” or experiment or experimental or in-vivo or in-vitro or “in vivo” or “in vitro” or mechanism or cells).sh,ti,kw. |
| 3 | Exp animals/ not humans.sh. |
| 4 | 1 not 2 |
| 5 | 4 not 3 |
| 6 | Limit 5 to dt=20110301-20220920 |
| 7 | Limit 6 to English language |
| 8 | Limit 7 to journal article |

| **Embase (Ovid)** | |
| --- | --- |
| 1 | (“vitamin D” or “vitamin D3” or “25-hydroxyvitamin D” or 25-hydroxyvitamin D3” or “25(OH)D” or “25(OH)D3” or “calcidiol”).sh,ti,ab,kw. |
| 2 | (“dihydroxycholecalciferol” or “dihydroxycholecalciferols” or “case reports” or “case series” or “case report” or “case control” or “case-control” or “clinical trial” or “controlled trial” or “review” or “systematic review” or “systematic-review” or experiment or experimental or in-vivo or in-vitro or “in vivo” or “in vitro” or mechanism or cells).sh,ti,kw. |
| 3 | (exp animal/ or exp invertebrate/ or nonhuman/ or animal experiment/ or animal tissue/ or animal model/ or exp plant/ or exp fungus/) not exp human/ not human tissue.sh. |
| 4 | 1 not 2 |
| 5 | 4 not 3 |
| 6 | Limit 5 to dc=20110301-20220920 |
| 7 | Limit 6 to English language |
| 8 | Limit 7 to journal article |

| **Web of Science** | |
| --- | --- |
| 12 | **#10 AND #11** |
| 11 | **(DT=(Article)) AND LA=(English) AND DOP=(2022-08-16/2023-06-16)** |
| 10 | **#8 not #9** |
| 9 | **SU=Veterinary Sciences** |
| 8 | #4 not #7 |
| 7 | #5 OR #6 |
| 6 | **AK=(“dihydroxycholecalciferol” or “dihydroxycholecalciferols” or “case reports” or “case series” or “case report” or “case control” or “case-control” or “clinical trial” or “controlled trial” or “review” or “systematic review” or “systematic-review” or experiment or experimental or in-vivo or in-vitro or “in vivo” or “in vitro” or mechanism or cells)** |
| 5 | **TI=(“dihydroxycholecalciferol” or “dihydroxycholecalciferols” or “case reports” or “case series” or “case report” or “case control” or “case-control” or “clinical trial” or “controlled trial” or “review” or “systematic review” or “systematic-review” or experiment or experimental or in-vivo or in-vitro or “in vivo” or “in vitro” or mechanism or cells)** |
| 4 | **#3 OR #2 OR #1** |
| 3 | **AK=(“vitamin D” OR “vitamin D3” OR “25-hydroxyvitamin D” OR “25-hydroxivitamin D3” OR “25(OH)D” OR “25(OH)D3”)** |
| 2 | **AB=(“vitamin D” OR “vitamin D3” OR “25-hydroxyvitamin D” OR “25-hydroxyvitamin D3” OR “25(OH)D” OR “25(OH)D3” OR “calcidiol”)** |
| 1 | **TI=(“vitamin D” OR “vitamin D3” OR “25-hydroxyvitamin D” OR “25-hydroxyvitamin D3” OR “25(OH)D” OR “25(OH)D3” OR “calcidiol”)** |

| **Global Index Medicus** |
| --- |
| ((tw:("vitamin D" OR "vitamin D3" OR "25-hydroxyvitamin D" OR "25-hydroxyvitamin D3" OR "25(O D" H)OR "25(OH)D3" OR "calcidiol")) AND NOT (ti:("dihydroxycholecalciferol" OR "dihydroxycholecalciferols" OR "case reports" OR "case series" OR "case report" OR "case control" OR "case-control" OR "clinical trial" OR "controlled trial" OR "review" OR "systematic review" OR "systematic review" OR "systematic-review" OR experiment OR experimental OR in-vivo OR in-vitro OR "in vivo" OR "in vitro" OR mechanism OR cells OR cell))) AND ( mj:("Humans") AND la:("en")) AND (year_cluster:[2011 TO 2022]) |

# Supplementary Table 2: Study characteristics

| Author, year | Study location | Eligible subjects  (*n*) | Overall study quality score | 25-hydroxyvitamin D assay | Certified assay or harmonised data (yes/no) |
| --- | --- | --- | --- | --- | --- |
| Abdeen, 2015 ^1^ | Hebron and Gaza, Palestine | 169 | 8 | RIA | No |
| Abidin, 2021^2^ | Klang Valley and Semenyih, Malaysia | 141 | 6 | CMIA | No |
| Abrahão, 2021 ^3^ | India | 819 | 6 | CLIA | No |
| Abu Shady,  2016 ^4^ | Gizeh, Egypt | 200 | 6 | EIA | No |
| Acherjya, 2020 ^5^ | Jashore, Bangladesh | 152 | 6 | CLIA | No |
| Afkhami-Ardekani, 2019 ^6^ | Yazd,  Iran | 700 | 8 | CLIA | No |
| Aggarwal, 2021^7^ | Chandigarh,  India | 915 | 7 | CLIA | No |
| Akman, 2011 ^8^ | Ankara, Turkey | 849 | 7 | HPLC | No |
| Al Hafidh, 2020 ^9^ | Mosul, Iraq | 300 | 9 | ELFA | No |
| Al Shaikh, 2020 ^10^ | Saudi Arabia | 3613 | 6 | CLIA | No |
| Al Shaikh, 2016 ^11^ | Saudi Arabia | 2110 | 6 | CLIA | No |
| Al-Dabhani, 2017 ^12^ | Qatar | 1176 | 6 | CLIA | No |
| Al-Daghri, Sabico, Al-Saleh; 2016 ^13^ | Riyadh, Saudi Arabia | 4183 | 6 | ECLIA | No |
| Al-Daghri, 2022 ^14^ | Riyadh, Saudi Arabia | 2938 | 6 | CLIA | Yes |
| Al-Eisa, 2016 ^15^ | Riyadh, Saudi Arabia | 85 | 6 | ELISA | No |
| Al-Ghamdi, 2012 ^16^ | Jeddah,  Saudi Arabia | 300 | 6 | ECLIA | No |
| Al-Raddadi, 2018 ^17^ | Jeddah,  Saudi Arabia | 421 | 8 | CLIA | No |
| Al-Saleh, 2015 ^18^ | Riyadh, Saudi Arabia | 2226 | 8 | ECLIA | Yes |
| Al-Sumaih, 2021 ^19^ | Saudi Arabi | 2641 | 9 | N/A | No |
| Al-Taiar, 2018 ^20^ | Kuwait | 1416 | 10 | LC-MS/MS | No |
| AlAnouti, 2022 ^21^ | United Arab Emirates | 399 | 6 | CLIA | No |
| Albalawi, 2022 ^22^ | Saudi Arabia | 3432 | 6 | CLIA | No |
| AlBuhairan, 2015 ^23^ | Saudi Arabia | 12575 | 10 | CLIA | No |
| ALbuloshi, 2022 ^24^ | Kuwait | 237 | 6 | LC-MS/MS | Yes |
| Alghadir, 2017 ^25^ | Mansoura, Egypt | 250 | 7 | EIA | No |
| Ali, 2020 ^26^ | France | 53 | 6 | RIA | No |
| Alyahya, 2014 ^27^ | Kuwait | 232 | 6 | RIA | Yes |
| Amaliya, 2015 ^28^ | West Java, Indonesia | 98 | 6 | HPLC | No |
| Amanzholkyzy, 2018 ^29^ | Western Kazakhstan Region, Kazakhstan | 110 | 6 | ECLIA | No |
| Andersen, Jakobsen, and Laurberg, 2013 ^30^ | Ilulissat; Saqqaq, Greenland | 97 | 8 | RIA | No |
| Andersen, Brot, Jakobsen, et al., 2013 ^31^ | Copenhagen; Frederiksberg;  Denmark | 106 | 8 | HPLC | Yes |
| Andersen, 2018 ^32^ | Greenland | 535 | 9 | LC-MS/MS | No |
|  | Nuuk; Tasiilaq, Greenland | 150 | 9 | LC-MS/MS | No |
| Ando, 2018 ^33^ | Tokyo, Japan | 574 | 6 | CPBA | No |
| Angeles-Agdeppa, 2021 ^34^ | Manila; Quezon; Cagayan province; Butuan; Northern Samar province; Siquijor; Butuan; Sultan Kudarat Province; Philippines | 789 | 8 | ECLIA | No |
| Antczak-Domagała, 2019 ^35^ | Łódź, Poland | 187 | 4 | N/A | No |
| Ara, 2023 ^36^ | Bangladesh | 298 | 6 | ECLIA | No |
| Arabi, 2012 ^37^ | Greater Beirut, Lebanon | 192 | 8 | RIA | Yes |
| Arabi, 2021 ^38^ | Greater Beirut, Lebanon | 466 | 8 | ECLIA | No |
| Arazi, 2019 ^39^ | Iran | 182 | 7 | ELISA | No |
| Arnljots, 2017 ^40^ | Sweden | 545 | 8 | CLIA | Yes |
| Arora, 2020 ^41^ | Ahmadabad, India | 2412 | 5 | N/A | No |
| Asakura, 2020 ^42^ | Hokkaido and Kumamoto, Japan | 107 | 6 | LC-MS/MS | Yes |
| Asante, 2023 ^43^ | Nord-Trøndelag, Norway | 717 | 7 | CLIA | No |
| Ashraf, 2012 ^44^ | Birmingham, USA | 62 | 6 | LC-MS/MS | No |
| Aspelund, 2019 ^45^ | Reykjavik, Iceland | 10501 | 8 | CLIA | Yes |
| Au, 2012 ^46^ | Boston, USA | 145 | 6 | RIA | No |
| Augusto, 2015 ^47^ | Rio Branco, Brazil | 702 | 9 | HPLC | No |
| Awasthi, 2022 ^48^ | Bangalore; Bhubaneswar; Chandigarh; Dibrugarh; Jodhpur; Lucknow; Patna; Srinagar; Thiruvananthapuram; and Udupi, India | 2268 | 8 | CLIA | No |
| Azmathullah, 2016 ^49^ | South India, India | 52 | 8 | CLIA | No |
| Bachhel, 2015 ^50^ | Punjab, India | 150 | 5 | N/A | No |
| Bacon, 2016 ^51^ | Bay of Plenty and Rotorua Lakes, New Zealand | 566 | 8 | HPLC | Yes |
| Baek, 2021 ^52^ | Seoul, South Korea | 487 | 4 | N/A | No |
| Bano, 2018 ^53^ | Italy | 149 | 5 | CLIA | No |
| Bansal, 2014 ^54^ | New York; Maryland; North Carolina; Illinois; Minnesota; and California, USA | 6459 | 8 | HPLC | No |
| Barth-Jaeggi, 2020 ^55^ | Tajikistan | 1913 | 10 | ELISA | No |
| Basińska-Lewandowska, 2021 ^56^ | Łódź, Poland | 132 | 5 | CBPA | No |
| Batieha, 2011 ^57^ | Jordan | 4590 | 9 | RIA | No |
| Beer, 2020 ^58^ | Colombia | 39011 | 9 | CLIA | No |
| Bennouar, 2022 ^59^ | Blida, Algeria | 451 | 6 | CPBA | No |
| Berry, 2011 ^60^ | Britain, UK | 6789 | 10 | EIA | Yes |
| Beydoun, 2021 ^61^ | Baltimore, USA | 1760 | 8 | CLIA | No |
| Bhat, 2018 ^62^ | Lucknow, India | 241 | 7 | RIA | No |
| Bhatt, 2014 ^63^ | Delhi, India | 137 | 6 | RIA | No |
| Bhattacharjee, 2019 ^64^ | West Bengal, India | 206 | 4 | N/A | No |
| Biben, 2017 ^65^ | Bandung and Sumedang, Indonesia | 263 | 8 | ECLIA | No |
| Bilinski, 2021 ^66^ | Poland | 128 | 6 | RIA | No |
| Binobead, 2019 ^67^ | Riyadh, Saudi Arabia | 63 | 6 | CLIA | No |
| Binu, 2019 ^68^ | Vaniyambadi, India | 1565 | 4 | N/A | No |
| Blomberg, 2017 ^69^ | Massachusetts, USA | 1418 | 8 | RIA | No |
| Bogazzi, 2011 ^70^ | Italy | 241 | 6 | RIA | No |
| Bojar, 2020 ^71^ | Lublin, Poland | 396 | 6 | CLIA | No |
| Bonakdaran, 2016 ^72^ | Mashhad, Iran | 233 | 7 | CPBA | No |
| Borissova, 2013 ^73^ | Bulgaria | 2016 | 6 | LC-MS/MS | No |
| Boucher-Berry, 2012 ^74^ | New York, USA | 106 | 6 | RIA | No |
| Bowman, 2012 ^75^ | Oregon, USA | 104 | 7 | RIA | No |
| Braithwaite, 2015 ^76^ | West Kiang, Gambia | 237 | 8 | CLIA | Yes |
| Buchebner, 2019 ^77^ | Malmo, Sweden | 382 | 8 | HPLC | Yes |
| Buchman, 2021 ^78^ | Berlin, Germany | 416 | 8 | CLIA | No |
| Cabral, 2018 ^79^ | Porto, Portugal | 521 | 8 | CLIA | No |
| Cai, 2023 ^80^ | Harbin, China | 2352 | 7 | UPLC | No |
| Cairncross, 2016 ^81^ | New Zealand | 1329 | 6 | LC-MS/MS | No |
| Cantio, 2022 ^82^ | Odense, Denmark | 833 | 7 | LC-MS/MS | No |
| Capuano, 2021 ^83^ | Campania, Italy | 1200 | 8 | ELISA | No |
| Carrelli, 2011 ^84^ | New York, USA | 203 | 6 | RIA | No |
| Carrillo-Vega, 2017 ^85^ | Mexico | 1128 | 9 | CMIA | No |
| Casey, 2019 ^86^ | Multiple, Europe | 4495 | 8 | LC-MS/MS | No |
| Cashman, 2022 ^87^ | Ireland | 246 | 9 | LC-MS/MS | Yes |
| Cashman, 2013 ^88^ | Ireland | 1132 | 9 | ELISA | Yes |
| Castillo-Valenzuela, 2023 ^89^ | Santiago; Antofagasta; Concepcion, Chile | 1235 | 9 | ELISA | No |
| Cediel, 2016 ^90^ | Santiago, Chile | 426 | 9 | CLIA | No |
| Chailurkit, 2012 ^91^ | Thailand | 2641 | 10 | LC-MS/MS | No |
| Chan, 2012 ^92^ | Hong Kong, China | 939 | 8 | RIA | No |
| Chaudhry, 2018 ^93^ | Gaza; West Bank, Palestine | 150 | 5 | N/A | No |
| Checkley, 2015 ^94^ | Lima; Tumbes, Peru | 1134 | 9 | CLIA | No |
| Chen, 2018 ^95^ | Yilan County, Taiwan | 1839 | 7 | N/A | No |
| Chen, 2019 ^96^ | Shenyang, China | 629 | 6 | LC-MS/MS | No |
| Chen, 2017 ^97^ | China | 6014 | 9 | RIA | Yes |
| Chen, 2016 ^98^ | Taiwan | 1556 | 6 | RIA | No |
| Chen, 2015 ^99^ | Qujing, China | 1078 | 6 | CLIA | No |
| Cheong, 2020 ^100^ | Singapore | 400 | 6 | ECLIA | No |
| Chiang, 2017 ^101^ | South Africa; USA; Jamaica; Ghana; Seychelles | 2242 | 8 | LC-MS/MS | No |
| Chlebna-Sokół, 2019 ^102^ | Łódź; Poznań; Lublin; Szczecin; Białystok; and Upper-Silesian Conurbation, Poland | 720 | 6 | ECLIA | Yes |
| Choi, 2014 ^103^ | South Korea | 260 | 7 | RIA | No |
| Choi, 2017 ^104^ | Ganghwa Island, South Korea | 651 | 8 | CLIA | No |
| Chua, 2020 ^105^ | Krau Wildlife Reserve, Malaysia | 555 | 7 | ECLIA | No |
| Chuang, 2016 ^106^ | Taipei; Yangmei; Miaoli; Changhua; Hualien; Chiayi; Kaohsiung, Taiwan | 5230 | 8 | ELISA | Yes |
| Chuc, 2019 ^107^ | Hung Yen, Vietnam | 327 | 6 | N/A | No |
| Cirillo, 2022 ^108^ | Molise, Italy | 979 | 8 | CLIA | No |
| Clark, 2021 ^109^ | Toluca, Mexico | 275 | 6 | LC-MS/MS | No |
| Cobayashi, 2015 ^110^ | Acrelândia, Brazil | 974 | 6 | HPLC | No |
| Contreras-Manzano, 2021 ^111^ | Mexico | 1262 | 9 | CMIA | No |
| Conzade, 2017 ^112^ | Augsburg, Germany | 1040 | 7 | ECLIA | No |
| Courraud, 2020 ^113^ | Siorapaluk; Qaanaaq; and Nuuk,Greenland | 177 | 8 | LC-MS/MS | Yes |
| Cremers, 2011 ^114^ | Netherlands | 287 | 6 | ELISA | No |
| Croll, 2021 ^115^ | Netherlands | 2716 | 8 | ECLIA | No |
| Cui, 2022 ^116^ | USA | 71685 | 9 | LC-MS/MS | Yes |
| da Silva, 2018 ^117^ | Aiquara, Brazil | 91 | 7 | CMIA | No |
| Dalgård, 2011 ^118^ | Faroe Islands | 668 | 9 | LC-MS/MS | No |
| Das, 2022 ^119^ | Bauniabadh; Mirpur; and Matlab, Bangladesh | 216 | 6 | ELISA | No |
| Davarzani, 2021 ^120^ | Tehran, Iran | 270 | 6 | ELISA | No |
| de Koning, 2017 ^121^ | Amsterdam, Netherlands | 892 | 7 | RIA | No |
| de la Cruz-Góngora, 2021 ^122^ | Mexico | 803 | 8 | CLIA | No |
| de la Cruz-Góngora, 2019 ^123^ | Champotón; Campeche; Mérida; and Valladolid, Mexico | 783 | 8 | CLIA | No |
| de Menezes-Júnior, 2023 ^124^ | Iron Quadrangle, Brazil | 1709 | 7 | ECLIA | No |
| de Oliveira, 2020 ^125^ | Rio de Janeiro; Fortaleza; Brasília; and Porto Alegre, Brazil | 1152 | 7 | CLIA | No |
| de Oliveira, 2021 ^126^ | São Paulo, Brazil | 652 | 8 | CLIA | No |
| Del Brutto, 2015 ^127^ | Atahualpa, Ecuador | 220 | 8 | CLIA | No |
| Delinocente, 2022 ^128^ | England, UK | 3205 | 9 | CLIA | Yes |
| Delshad, 2019 ^129^ | Auckland, New Zealand | 507 | 6 | LC-MS/MS | No |
| Deng, 2022 ^130^ | Duijangyan, China | 590 | 6 | CPBA | No |
| Denova-Gutiérrez, 2019 ^131^ | Mexico | 533 | 6 | CLIA | No |
| Dhore, 2013 ^132^ | Amravati, India | 62 | 6 | CLIA | No |
| Diederichsen, 2017 ^133^ | Denmark | 1006 | 8 | LC-MS/MS | No |
| Diekmann, 2013 ^134^ | Nuremberg, Germany | 115 | 7 | ELISA | No |
| Dimakopoulos, 2019 ^135^ | Greece | 1084 | 8 | ECLIA | No |
| Divanoglou, 2021 ^136^ | Velestino, Greece | 98 | 8 | HPLC | No |
| Djennane, 2014 ^137^ | Tizi-Ouzou, Algeria | 435 | 6 | ECLIA | No |
| Dogan-Sander, 2021 ^138^ | Leipzig, Germany | 7162 | 8 | ECLIA | No |
| Dong, 2014 ^139^ | Wenchuan, China | 206 | 8 | RIA | No |
| Drali, 2021 ^140^ | Algiers, Algeria | 1016 | 6 | ELFA | No |
| Duarte, 2020 ^141^ | Portugal | 3092 | 9 | CLIA | Yes |
| Ebrahimi, 2014 ^142^ | Shahroud, Iran | 1047 | 8 | EIA | No |
| Egeland, 2011 ^143^ | Canada | 1901 | 8 | CLIA | No |
| El Badawy, 2015 ^144^ | Zagazig, Egypt | 550 | 9 | ELISA | No |
| El-Khateeb, 2019 ^145^ | Jordan | 3954 | 9 | CMIA | No |
| El Maataoui, 2016 ^146^ | Morocco | 254 | 6 | ECLIA | Yes |
| Ellul, 2020 ^147^ | Southwest Victoria, Australia | 438 | 7 | LC-MS/MS | No |
| Emmerson, 2018 ^148^ | Northwest, UK | 322 | 6 | LC-MS/MS | Yes |
| Erasmus, 2022 ^149^ | Bellville, South Africa | 589 | 6 | CLIA | No |
| Erdönmez, 2011 ^150^ | Turkey | 301 | 7 | ELIA | No |
| Ewendt, 2023 ^151^ | Halle, Germany | 2074 | 7 | HPLC | No |
| Fabian, 2012 ^152^ | Burgenland, Austria | 102 | 6 | HPLC | No |
| Farber, 2021 ^153^ | Portugal | 43 | 6 | CLIA | No |
| Fassula, 2021 ^154^ | Santa Catarina, Brazil | 605 | 8 | CLIA | No |
| Feehan, 2022 ^155^ | Northern Ireland, UK | 69 | 7 | HPLC | No |
| Flores, 2021 ^156^ | Mexico | 4691 | 10 | CMIA | No |
| Forney, 2017 ^157^ | Louisiana, USA | 63 | 6 | ELISA | No |
| Fox, 2023 ^158^ | Bonn, Germany | 2576 | 7 | CLIA | No |
| Galeazzi, 2023 ^159^ | Ancona, Italy | 470 | 7 | CLIA | No |
| Ganie, 2022 ^160^ | Kashmir, India | 1732 | 8 | CLIA | No |
| Gannagé-Yared, 2018 ^161^ | Great Beirut and Mount Lebanon, Lebanon | 969 | 9 | CLIA | No |
| Gao, 2017 ^162^ | Shanghai, China | 2251 | 7 | ECLIA | No |
| García-Dorta, 2021 ^163^ | Canary Islands, Spain | 876 | 8 | CLIA | No |
| Garg, 2018 ^164^ | West Bengal, India | 197 | 9 | N/A | No |
| Garg, 2014 ^165^ | New Delhi, India | 3175 | 6 | RIA | No |
| Ge, 2017 ^166^ | China | 1084 | 6 | ELISA | No |
| Gebreegziabher, 2013 ^167^ | Rift Valley, Ethiopia | 202 | 6 | ELISA | No |
| Ghobadi, 2019 ^168^ | Iran | 240 | 8 | ELISA | No |
| Giallauria, 2012 ^169^ | Baltimore, USA | 1228 | 7 | LC-MS/MS | No |
| Gill, 2014 ^170^ | Adelaide, Australia | 2413 | 8 | ELISA | No |
| Gómez Alonso, 2019 ^171^ | Oviedo, Spain | 290 | 8 | RIA | No |
| González-Molero, 2012 ^172^ | Andalusia, Spain | 412 | 7 | ECLIA | No |
| González-Molero, 2011 ^173^ | Asturias, Spain | 1262 | 6 | ECLIA | No |
| Goodwill, 2018 ^174^ | Melbourne, Australia | 252 | 8 | CLIA | No |
| Goswami, 2021 ^175^ | Agartala, India | 1000 | 4 | N/A | No |
| Grant, 2009 ^176^ | Auckland, New Zealand | 353 | 8 | RIA | No |
| Greene-Finestone, 2011 ^177^ | Multiple, Canada | 1912 | 9 | CLIA | Yes |
| Grineva, 2013 ^178^ | Saint Petersburg, Russia | 320 | 6 | EIA | No |
| Guan, 2020 ^179^ | Lanzhou, China | 100038 | 8 | EIA | No |
| Gudmundsdottir, 2020 ^180^ | Reykjavik, Iceland | 315 | 6 | ECLIA | No |
| Ha, 2013 ^181^ | Suwon, South Korea | 310 | 6 | CLIA | No |
| Habibesadat, 2014 ^182^ | Khorasan, Iran | 361 | 8 | EIA | No |
| Hacker-Thompson, 2012 ^183^ | San Franciso, USA | 122 | 6 | LC-MS/MS | No |
| Hanks, 2012 ^184^ | Birmingham, USA | 36 | 6 | LC-MS/MS | No |
| Hansen, 2015 ^185^ | Massachusetts, USA | 3599 | 8 | RIA | No |
| Hao, 2014 ^186^ | Shanghai, China | 1001 | 7 | ECLIA | No |
| Hasan, 2017 ^187^ | Sharjah, United Arab Emirates | 198 | 6 | ELISA | No |
| Haslam, 2014 ^188^ | Atlanta, USA | 194 | 8 | RIA | No |
| Hata, 2023 ^189^ | Kawasaki, Japan | 599 | 6 | ELISA | No |
| Hauksson, 2016 ^190^ | Reykjavik, Iceland | 153 | 8 | RIA | No |
| Hekimoğlu, 2023 ^191^ | Trabzon, Turkey | 140 | 6 | CLIA | No |
| Hekimsoy, 2010 ^192^ | Manisa, Turkey | 391 | 8 | HPLC | No |
| Henning, 2023 ^193^ | Europe | 642 | 6 | LC-MS/MS | No |
| Herrador, 2014 ^194^ | Ethiopia | 627 | 8 | CLIA | No |
| Hien, 2012 ^195^ | Vietnam | 477 | 8 | RIA | No |
| Hill, 2016 ^196^ | Newcastle Upon Tyne, UK | 775 | 8 | RIA | No |
| Hirani, 2013 ^197^ | Sydney, Australia | 1659 | 8 | RIA | No |
| Hirani, 2012 ^198^ | England, UK | 2070 | 9 | RIA | Yes |
| Hirschler, 2014 ^199^ | San Antonio de los Cobres, Argentina | 355 | 8 | CMIA | No |
| Hirschler, 2019 ^200^ | Chicoana, Argentina | 175 | 9 R | RIA | No |
| Hoevenaar-Blom, 2019 ^201^ | Utrecht, Netherlands | 150 | 6 | LC-MS/MS | Yes |
| Hoge, 2015 ^202^ | Wallonia, Belgium | 697 | 8 | CLIA | Yes |
| Horton-French, 2021 ^203^ | Australia | 1092 | 9 | LC-MS/MS | Yes |
| Houghton, 2019 ^204^ | Emali, Kenya | 259 | 6 | LC-MS/MS | No |
| Houston, 2011 ^205^ | North Carolina; California; Maryland; Pennsylvania, USA | 988 | 7 | LC-MS/MS | No |
| Hribar, 2020 ^206^ | Slovenia | 125 | 8 | LC-MS/MS | No |
| Hu, 2021 ^207^ | China | 1568 | 9 | LC-MS/MS | No |
| Hu, Zhi, Ma et al., 2022 ^208^ | Lishui, China | 1926 | 7 | CLIA | No |
| Hu, Jiang, Lu et al., 2022 ^209^ | China | 64391 | 9 | LC-MS/MS | No |
| Huang, 2011 ^210^ | Ontario, Canada | 356 | 6 | CLIA | No |
| Huang, 2013 ^211^ | Harbin, China | 2708 | 9 | HPLC | No |
| Huang, 2019 ^212^ | China | 13565 | 7 | CLIA | Yes |
| Huang, 2022 ^213^ | Ganzi, China | 1815 | 6 | LC-MS/MS | No |
| Huang, 2023 ^214^ | Guangzhou, China | 2036 | 8 | LC-MS/MS | No |
| Hurskainen, 2012 ^215^ | Kuopio, Finland | 1756 | 9 | HPLC | No |
| Husemoen, 2016 ^216^ | Denmark | 748 | 6 | HPLC | Yes |
| Hussain, 2020 ^217^ | Punjab, Pakistan | 510 | 5 | RIA | No |
| Huta-Osiecka, 2017 ^218^ | Poland | 35 | 6 | ELISA and CLIA | No |
| Hutchings, 2022 ^219^ | Armenia | 1206 | 9 | LC-MS/MS | No |
| Ikonen, 2021 ^220^ | Finland | 3650 | 9 | LC-MS/MS | Yes |
| Imran, 2019 ^221^ | Jakarta, Indonesia | 45 | 7 | LC-MS/MS | No |
| Jääskeläinen, 2017 ^222^ | Finland | 4051 | 10 | CLIA | Yes |
| Jain, 2011 ^223^ | Delhi, India | 98 | 6 | RIA | No |
| Jakab, 2017 ^224^ | Debrecen, Hungary | 206 | 6 | HPLC | Yes |
| Jamali, 2013 ^225^ | Rafsanjan, Iran | 250 | 8 | RIA | No |
| Jamka, 2021 ^226^ | Poland | 92 | 6 | EIA | No |
| Jang, 2013 ^227^ | Gwacheon, South Korea | 320 | 6 | RIA | No |
| Janmohamed, 2020 ^228^ | Mongolia | 938 | 9 | ELFA | No |
| Janssen, 2013 ^229^ | Netherlands | 400 | 5 | CLIA | No |
| Jarosz, 2021 ^230^ | Toronto, Canada | 716 | 6 | CLIA | No |
| Jayatissa, 2019 ^231^ | Sri Lanka | 2525 | 9 | CLIA | No |
| Jeddi, 2013 ^232^ | Kawar, Iran | 476 | 9 | HPLC | No |
| Jeenduang, 2020 ^233^ | Nakhon Si Thammarat, Thailand | 340 | 7 | ECLIA | No |
| Jeenduang, 2018 ^234^ | Nakhon Si Thammarat, Thailand | 839 | 6 | ECLIA | No |
| Jelmila, 2020 ^235^ | Padang, Indonesia | 93 | 8 | ELISA | No |
| Jeon, 2017 ^236^ | South Korea | 740 | 6 | CLIA | No |
| Jiajue, 2021^237^ | Beijing, China | 1635 | 8 | ECLIA | No |
| Jiang, 2020 ^238^ | China | 14302 | 7 | ECLIA | No |
| Johansson, 2011 ^239^ | Sweden | 2878 | 9 | RIA | No |
| Jones, 2020 ^240^ | Central Coast, Australia | 619 | 6 | HPLC | No |
| Joukar, 2020 ^241^ | Guilan, Iran | 9520 | 8 | ECLIA | No |
| Juwita, 2021 ^242^ | Java, Indonesia | 109 | 6 | ELISA | No |
| Kaddam, 2017 ^243^ | Saudi Arabia | 6139 | 8 | CLIA | No |
| Kandhro, 2019 ^244^ | Pakistan | 1244 | 7 | CMIA | No |
| Kaneva, 2022 ^245^ | Russia | 90 | 5 | EIA | No |
| Kapil, 2017 ^246^ | Himachal Pradesh, India | 626 | 8 | CLIA | No |
| Kapil, 2018 ^247^ | Himachal Pradesh, India | 1222 | 8 | CLIA | No |
| Karagüzel, 2014 ^248^ | Trabzon, Turkey | 746 | 6 | HPLC | No |
| Karhapää, 2012 ^249^ | Kuopio, Finland | 909 | 6 | N/A | No |
| Karimi, 2019 ^250^ | Fars, Iran | 413 | 7 | HPLC | No |
| Karin, 2018 ^251^ | Croatia | 260 | 8 | CPBA | Yes |
| Karuppusami, 2020 ^252^ | Vellore, India | 373 | 8 | CLIA | No |
| Kassem, 2020 ^253^ | Hadera, Israel | 116 | 6 | CLIA | No |
| Kaykhaei, 2011 ^254^ | Iran | 993 | 6 | CLIA | No |
| Ke, 2015 ^255^ | Macao, China | 566 | 8 | ECLIA | No |
| Ke, 2018 ^256^ | Sydney, Australia | 162 | 6 | RIA | No |
| Kensara, 2019 ^257^ | Mecca, Saudi Arabia | 704 | 6 | CPBA | No |
| Kensarah, 2015 ^258^ | Mecca, Saudi Arabia | 503 | 8 | ECLIA | No |
| Khader, 2011 ^259^ | Jordan | 3234 | 9 | RIA | No |
| Khadilkar, 2022 ^260^ | India | 2500 | 8 | LC-MS/MS | No |
| Khan, 2019 ^261^ | Karachi, Pakistan | 167 | 6 | CLIA | No |
| Khan, 2012 ^262^ | Karachi, Pakistan | 305 | 8 | ECLIA | No |
| Khan, 2020 ^263^ | Dharan ; Itahari, Nepal | 181 | 7 | ELISA | No |
| Khayyatzadeh, 2018 ^264^ | Mashhad ;Sabzevar, Iran | 965 | 9 | ECLIA | No |
| Khor, 2011 ^265^ | Kuala Lumpur, Malaysia | 402 | 9 | CLIA | No |
| Khosravi-Boroujeni, 2017 ^266^ | Iran | 370 | 6 | ELISA | No |
| Khwanchuea, 2022 ^267^ | Thailand | 205 | 5 | ELISA | No |
| Kim, 2012 ^268^ | Chungju, South Korea | 1039 | 8 | CLIA | No |
| Kim, 2020 ^269^ | South Korea | 2872 | 10 | CLIA | No |
| Kim, 2013 ^270^ | Seoul, South Korea | 186 | 6 | RIA | No |
| Kim, 2022 ^271^ | South Korea | 139599 | 6 | ECLIA | No |
| Kimlin, 2014 ^272^ | Australia | 1002 | 9 | CLIA | Yes |
| Kılınç, 2019 ^273^ | Turkey | 180 | 6 | ECLIA | No |
| Klenk, 2013 ^274^ | Ulm, Germany | 1418 | 7 | ECLIA | No |
| Koda, 2023 ^275^ | Gifu, Japan | 612 | 5 | CPBA | No |
| Kopiczko, 2020 ^276^ | Warsaw, Poland | 100 | 8 | CLIA | Yes |
| Kouda, 2013 ^277^ | Hamamatsu, Japan | 400 | 6 | RIA | No |
| Kruavit, 2012 ^278^ | Bangkok, Thailand | 93 | 6 | RIA | No |
| Kull, 2012 ^279^ | LääNe-Viru, Estonia | 273 | 7 | RIA | No |
| Kuraoka, 2022 ^280^ | Japan | 3624 | 9 | LC-MS/MS | No |
| Kutlay, 2014 ^281^ | Ankara, Turkey | 876 | 8 | RIA | No |
| Kuwabara, 2020 ^282^ | Kyoto, Japan | 148 | 6 | RIA | No |
| Laaksi, 2023 ^283^ | Finland | 412 | 5 | ECLIA | No |
| Laillou, 2013 ^284^ | Vietnam | 485 | 8 | HPLC | No |
| Laird, 2018 ^285^ | Ireland | 5356 | 10 | LC-MS/MS | Yes |
| Lam, 2016 ^286^ | Perth, Australia | 176 | 6 | EIA | No |
| Langrebe, 2021 ^287^ | Denmark | 2211 | 7 | ECLIA | No |
| Larcombe, 2012 ^288^ | Lac Brochet, Canada | 46 | 6 | ELISA | No |
| Larijani, 2016 ^289^ | Tehran, Iran | 444 | 8 | EIA | No |
| Larsen, 2022 ^290^ | Tromso, Norway | 20438 | 7 | LC-MS/MS | Yes |
| Lategan, 2016 ^291^ | Mangaung, South Africa | 339 | 7 | CLIA | No |
| Lee, 2011 ^292^ | Europe | 3151 | 8 | RIA | No |
| Lee, 2017 ^293^ | South Korea | 2942 | 6 | CLIA | No |
| Lee, 2020 ^294^ | South Korea | 533 | 6 | CLIA | No |
| Lee, 2021 ^295^ | Yangpyeong, South Korea | 2721 | 6 | CLIA | No |
| Leirós, 2022 ^296^ | Santiago De Compostela, Spain | 113 | 6 | CLIA | No |
| Leitão, 2018 ^297^ | Mainland Portugal | 789 | 8 | ECLIA | No |
| Lemming, 2022 ^298^ | Riksmaten, Sweden | 166 | 9 | HPLC | Yes |
| Leung, 2017 ^299^ | Hong Kong, China | 5276 | 6 | EIA | No |
| Li, 2019 ^300^ | Henan, China | 1528 | 6 | ECLIA | No |
| Li, 2017 ^301^ | Shanghai, China | 967 | 8 | HPLC | No |
| Li, 2016 ^302^ | Beijing; Wuhan; Guangzhou; Shanghai; and Chongqing, China | 1436 | 7 | CLIA | No |
| Li, Li, Li et al., 2020 ^303^ | Chengdu; Nanchong; Luzhou; Maerkang; Guangyuan; and Panzhihua, China | 2271 | 6 | LC-MS/MS | No |
| Li, Wei, Xue et al., 2018 ^304^ | Southwest China | 1514 | 8 | HPLC | No |
| Li, Ding, Cao et al., 2018 ^305^ | Ningxia, China | 1582 | 8 | ELISA | No |
| Li, 2021 ^306^ | Beijing; Tianjin; and Shijiazhuang, China | 3090 | 8 | LC-MS/MS | No |
| Li, Huang, Xiao et al., 2020 ^307^ | China | 10696 | 9 | CLIA | No |
| Liaqat, 2019 ^308^ | Mianwali, Pakistan | 380 | 4 | N/A | No |
| Lim, 2012 ^309^ | Seongnam City, South Korea | 921 | 8 | LC-MS/MS | No |
| Lima-Costa, 2020 ^310^ | Brazil | 2264 | 9 | CMIA | Yes |
| Lin, 2021 ^311^ | UK | 449943 | 9 | CLIA | Yes |
| Lingham, 2019 ^312^ | Busselton, Australia | 4112 | 9 | CMIA | No |
| Lingham, 2021 ^313^ | Perth, Australia | 1260 | 9 | LC-MS/MS | No |
| Liu, Ma, Wei et al., 2018 ^314^ | Gansu, China | 11157 | 6 | CLIA | No |
| Liu, Li, Zhao et al., 2018 ^315^ | Beijing; Suzhou; Guangzhou; Zhengzhou; Lanzhou; Xingtai; and Hebei, China | 563 | 9 | LC-MS/MS | No |
| Liu, 2020 ^316^ | Xiayi; Zhongxiang; Laizhou; Yongfu; Sanshui; Mayang; Chengmai; and Rudong, China | 2493 | 9 | ELISA | No |
| Liu, Brock, Brennan- Speranza, 2023 ^317^ | Perth, Australia | 2717 | 5 | RIA | No |
| Liu, 2021 ^318^ | Shenyang, China | 131 | 6 | LC-MS/MS | No |
| Lopes, 2014 ^319^ | Sao Paulo, Brazil | 908 | 6 | RIA | No |
| Lourenço, 2014 ^320^ | Acrelândia, Brazil | 436 | 8 | HPLC | No |
| Lu, 2012 ^321^ | Beijing and Shanghai, China | 3210 | 8 | RIA | No |
| Lu, 2017 ^322^ | Shanghai; Zhejiang; Jiangsu; Anhui; and Jiangxi Province, China | 12321 | 8 | CLIA | No |
| Luo, 2021 ^323^ | China | 4236 | 7 | N/A | No |
| Lutsey, 2016 ^324^ | Minneapolis, Minnesota; Forsyth County, North Carolina; Washington County, Maryland; and Jackson, Mississippi, USA | 160 | 8 | LC-MS/MS | No |
| Luxwolda, 2013 ^325^ | Tanzania | 88 | 6 | LC-MS/MS | No |
| Lwow, 2020 ^326^ | Wroclaw, Poland | 318 | 6 | RIA | No |
| Macdonald, 2011 ^327^ | Scotland, UK | 314 | 7 | ELISA | No |
| Macdonell, 2021 ^328^ | New Zealand | 285 | 6 | LC-MS/MS | No |
| Macdonell, 2016 ^329^ | New Zealand | 292 | 6 | LC-MS/MS | No |
| Maddah, 2009 ^330^ | Guilan, Iran | 646 | 8 | ELISA | No |
| Madsen, 2014 ^331^ | Copenhagen, Denmark | 755 | 8 | LC-MS/MS | Yes |
| Majumdar, 2011 ^332^ | India | 441 | 6 | EIA | No |
| Malacova, 2019 ^333^ | Australia | 5034 | 9 | LC-MS/MS | Yes |
| Mallah, 2011 ^334^ | Amman, Jordan | 300 | 6 | ELISA | No |
| Manios, 2017 ^335^ | Attica; Aitoloakarnania; Thessaloniki; and Iraklio, Greece | 2353 | 9 | CLIA | Yes |
| Mansuri, 2016 ^336^ | Sandy Lake, Canada | 445 | 7 | CLIA | No |
| Marasinghe, 2015 ^337^ | Ragama, Sri Lanka | 340 | 8 | CLIA | No |
| Martini, 2013 ^338^ | São Paulo, Brazil | 1611 | 7 | HPLC | No |
| Marwaha, 2011 ^339^ | Delhi, India | 1600 | 6 | RIA | No |
| Marzban, 2021 ^340^ | Bushehr, Iran | 1806 | 8 | ELISA | No |
| Masoud, 2020 ^341^ | Riyadh, Saudi Arabia | 170 | 8 | ECLIA | Yes |
| Masson, 2015 ^342^ | Lazio, Italy | 1835 | 6 | ECLIA | No |
| Mat, 2018 ^343^ | Kuala Lumpur, Malaysia | 1011 | 7 | N/A | No |
| Mata-Granados, 2013 ^344^ | Cordoba, Spain | 232 | 7 | HPLC | No |
| Mavroeidi, 2013 ^345^ | Aberdeen, UK | 355 | 7 | EIA | No |
| Mayer, 2017 ^346^ | Pilsen, Czech Republic | 1023 | 9 | CLIA | No |
| Mba, 2022 ^347^ | Bafut; Mbankomo; Bamenda; and Yaoundé, Cameroon | 586 | 8 | LC-MS/MS | Yes |
| McCullough, 2021 ^348^ | USA | 654 | 6 | LC-MS/MS | No |
| Meems, 2015 ^349^ | Netherlands | 8726 | 6 | LC-MS/MS | No |
| Mehboobali, 2015 ^350^ | Karachi, Pakistan | 858 | 6 | CLIA | No |
| Menant, 2012 ^351^ | Sydney, Australia | 463 | 8 | CLIA | No |
| Menezes, 2023 ^352^ | Bahia, Brazil | 185 | 5 | Not reported | No |
| Meng, 2017 ^353^ | Beijing, China | 912 | 6 | RIA | No |
| Meo, 2016 ^354^ | Riyadh, Saudi Arabia | 113 | 6 | CLIA | No |
| Meoli, 2021 ^355^ | Switzerland | 298 | 8 | CLIA | Yes |
| Merchant, 2018 ^356^ | Singapore | 295 | 8 | ECLIA | No |
| Meshkibaf, 2021 ^357^ | Fars, Iran | 196 | 6 | ELISA | No |
| Michaëlsson, 2017 ^358^ | Uppsala, Sweden | 5002 | 7 | LC-MS/MS | Yes |
| Michalus, 2013 ^359^ | Lodz, Poland | 155 | 6 | ECLIA | Yes |
| Middelkoop, 2022 ^360^ | Cape Town, South Africa | 1823 | 8 | LC-MS/MS | No |
| Mielgo-Ayuso, 2017 ^361^ | Europe | 1058 | 9 | ELISA | No |
| Miettinen, 2014 ^362^ | Finland | 2822 | 9 | CMIA | Yes |
| Milagres, 2020 ^363^ | Vicosa, Brazil | 378 | 8 | CLIA | No |
| Milagres, 2021 ^364^ | Vicosa, Brazil | 378 | 8 | CLIA | No |
| Mirza, 2023 ^365^ | Uttarakhand, India | 300 | 6 | CLIA | No |
| Mogire, 2021 ^366^ | Kenya; Uganda; Burkina Faso; The Gambia; and South Africa, Africa | 4509 | 6 | CMIA | No |
| Mohammad, 2021 ^367^ | Syria | 197 | 6 | ECLIA | No |
| Moran, 2013 ^368^ | Llerena-Zafra, Spain | 195 | 6 | RIA | No |
| Muhairi, 2013 ^369^ | Al Ain, United Arab Emirates | 315 | 8 | RIA | No |
| Mukhopadhyay, Ghosh, Bhattacharjee et al., 2019 ^370^ | Birbhum, India | 405 | 6 | CLIA | No |
| Mukhopadhyay, Ghosh, Pandit et al., 2019 ^371^ | West Bengal, India | 405 | 6 | CLIA | No |
| Mustafa, 2021 ^372^ | India | 39311 | 9 | CLIA | No |
| Mutt, 2019 ^373^ | Oulu, Finland | 636 | 7 | EIA | No |
| Naganuma, 2022 ^374^ | Otawara, Japan | 492 | 6 | RIA | No |
| Nakamura, 2011 ^375^ | Muramatsu, Japan | 751 | 8 | CPBA | No |
| Nakamura, 2020 ^376^ | Niigata, Japan | 1044 | 8 | CLIA | No |
| Nakamura, Tsujiguchi, Hara et al., 2019 ^377^ | Ishikawa, Japan | 619 | 8 | RIA | No |
| Nakamura, Hui, Ukawa et al., 2019^378^ | Hokkaido, Japan | 512 | 6 | LC-MS/MS | No |
| Nakaoka, 2021 ^379^ | Tokyo, Japan | 175 | 6 | RIA | No |
| Nakhaee, 2019 ^380^ | Birjand, Iran | 400 | 6 | ECLIA | No |
| Nälsén, 2020 ^381^ | Sweden | 268 | 8 | LC–MS | Yes |
| Narchi, 2015 ^382^ | Al-'Ayn, United Arab Emirates | 293 | 8 | ECLIA | No |
| Navarrete-Reyes, 2015 ^383^ | Mexico City, Mexico | 331 | 9 | ELISA | No |
| Neyestani, 2012 ^384^ | Iran | 1111 | 9 | CPBA | No |
| Nguyen, 2020 ^385^ | Can Tho, Vietnam | 794 | 8 | HPLC | No |
| Nguyen, 2012 ^386^ | Vietnam | 491 | 9 | ECLIA | No |
| Ni Chaoimh, 2018 ^387^ | Cork, Ireland | 741 | 8 | HPLC | Yes |
| Niafar, 2009 ^388^ | Tabriz, Iran | 300 | 8 | CLIA | No |
| Nichols, 2015 ^389^ | Jordan | 915 | 9 | LC-MS/MS | Yes |
| Nichols, 2012 ^390^ | Jordan | 2032 | 9 | LC-MS/MS | Yes |
| Nielsen, 2016 ^391^ | Nuuk, Greenland | 2877 | 9 | LC-MS/MS | No |
| Niimi, 2022 ^392^ | Nagasaki, Japan | 480 | 6 | CLIA | No |
| Nikooyeh, 2017 ^393^ | Iran | 1406 | 10 | ELISA | No |
| Nikooyeh, 2017^394^ | Iran | 667 | 9 | ELISA | No |
| Nurbazlin, 2013 ^395^ | Malaysia | 400 | 6 | ECLIA | No |
| Ó Breasail, 2023 ^396^ | Gambia | 386 | 4 | RIA | Yes |
| O'Brien, 2017 ^397^ | Alaska, USA | 109 | 8 | LC-MS/MS | No |
| Öberg, 2014 ^398^ | Tromso, Norway | 890 | 9 | LC-MS/MS | Yes |
| Okan, 2020 ^399^ | Tokat, Turkey | 72 | 6 | ECLIA | No |
| Oliveira, 2021 ^400^ | Rio De Janeiro, Brazil | 511 | 8 | CLIA | No |
| Olsen, 2014 ^401^ | Norway | 310 | 8 | LC-MS/MS | No |
| Orces, 2015 ^402^ | Ecuador | 2374 | 8 | LC-MS/MS | No |
| Oshiro, 2022 ^403^ | Hawaii, USA | 223 | 8 | LC-MS/MS | No |
| Oudshoorn, 2012 ^404^ | Rotterdam, Netherlands | 426 | 6 | RIA | No |
| Oussedik-Lehtihet, 2017 ^405^ | Algiers, Algeria | 336 | 7 | ECLIA | No |
| Overby, 2014 ^406^ | Vest-Agder, Norway | 92 | 6 | LC-MS/MS | No |
| Paes-Silva, 2018 ^407^ | Reclife, Brazil | 411 | 8 | HPLC | No |
| Pan, 2020 ^408^ | Chandanngar, India | 260 | 8 | RIA | No |
| Panahi, 2022 ^409^ | Bushehr, Iran | 400 | 8 | ELISA | No |
| Pang, 2021 ^410^ | China | 1435 | 5 | CLIA | No |
| Pankiv, 2020 ^411^ | Ukraine | 304 | 5 | CLIA | No |
| Pannu, 2017 ^412^ | Victoria, Australia | 2669 | 8 | CLIA | No |
| Pantovic, 2019 ^413^ | Serbia | 87 | 6 | CLIA | No |
| Park, 2015 ^414^ | Seoul, South Korea | 535 | 6 | CLIA | No |
| Park, 2018 ^415^ | South Korea | 39759 | 10 | RIA | Yes |
| Passi-Solar, 2020 ^416^ | Chile | 6113 | 9 | LC-MS/MS | No |
| Patel, 2016 ^417^ | Gujarat, India | 181 | 8 | CMIA | No |
| Patel, 2017 ^418^ | Gujarat, India | 120 | 7 | CMIA | No |
| Patel, 2023 ^419^ | UK | 2842 | 9 | LC-MS/MS | Yes |
| Patriota, 2022 ^420^ | Lausanne, Switzerland | 6485 | 5 | HPLC | No |
| Pazaitou-Panayiotou, 2012 ^421^ | Thessaloniki, Greece | 489 | 6 | ECLIA | No |
| Peng, 2013 ^422^ | Suzhou, China | 1553 | 9 | ELISA | No |
| Pérez-Bravo, 2022 ^423^ | Chile | 1134 | 6 | ELISA | No |
| Peters, 2012 ^424^ | Sao Paulo, Brazil | 160 | 6 | RIA | No |
| Petrenya, 2020 ^425^ | Norway | 4465 | 8 | CLIA | Yes |
| Petry, 2020 ^426^ | Oman | 1036 | 9 | N/A | No |
| Pilz, 2012 ^427^ | Australia | 961 | 6 | RIA | No |
| Poh, 2016 ^428^ | Indonesia | 276 | 9 | IADS | No |
| Poopedi, 2022 ^429^ | Johannesburg, South Africa | 200 | 6 | CLIA | Yes |
| Pouraram, 2018 ^430^ | Iran | 32770 | 9 | ELICA | No |
| Pulungan, 2018 ^431^ | Rampasasa, Indonesia | 58 | 8 | CLIA | No |
| Pusparini, 2016 ^432^ | Jakarta, Indonesia | 160 | 7 | CLIA | No |
| Qiao, 2013 ^433^ | Guiyang, China | 1510 | 9 | RIA | No |
| Quah, 2018 ^434^ | Malaysia | 1016 | 9 | CLIA | No |
| Rabenberg, 2018 ^435^ | Berlin, Germany | 17010 | 8 | CLIA | Yes |
| Rad, 2015 ^436^ | Tehran, Iran | 257 | 6 | HPLC | No |
| Rafraf, 2014 ^437^ | Bukan, Iran | 216 | 9 | ELISA | No |
| Rahmadhani, 2017 ^438^ | Kuala Kangsar, Malaysia | 678 | 8 | ECLIA | No |
| Raina, 2014 ^439^ | Jammu, India | 702 | 6 | RIA | No |
| Ramankutty, 2014 ^440^ | Perth, Australia | 461 | 6 | ELISA | No |
| Raposo, 2017 ^441^ | Portugal | 500 | 9 | CLIA | No |
| Rastad, 2021 ^442^ | Iran | 2596 | 10 | CLIA | No |
| Raszewski, 2019 ^443^ | Lublin, Poland | 170 | 7 | ELISA | No |
| Rathnayake, 2021 ^444^ | Galle, Sri Lanka | 350 | 6 | ELISA | No |
| Rathod, 2015 ^445^ | Switzerland | 1293 | 6 | LC-MS/MS | No |
| Raulio, 2017 ^446^ | Finland | 1575 | 9 | CLIA | Yes |
| Rautenbach, 2022 ^447^ | South Africa | 660 | 7 | ECLIA | No |
| Rehman, 2020 ^448^ | Quetta, Pakistan | 142 | 8 | ELISA | No |
| Rezaei, 2023 ^449^ | Birjand, Iran | 1319 | 7 | ELISA | No |
| Riaz, 2016 ^450^ | Pakistan | 4830 | 8 | ECLIA | No |
| Rips, 2023 ^451^ | Voru, Estonia | 65 | 6 | CLIA | No |
| Riverin, 2014 ^452^ | Quebec, Canada | 52 | 8 | RIA | No |
| Riverin, 2013 ^453^ | James Bay, Canada | 944 | 8 | RIA | No |
| Robinson, 2017 ^454^ | Guatemala, El Salvador, the Dominican Republic, Honduras, Nicaragua, Costa Rica, Panama, Belize, and Mexico (Nine MesoAmerican Countries) | 223 | 6 | CLIA | No |
| Rodríguez-Rodríguez, 2014 ^455^ | Madrid, Spain | 94 | 8 | CLIA | No |
| Rodríguez-Rodríguez, Ortega, González-Rodríguez et al., 2011 ^456^ | Madrid, Spain | 149 | 8 | CLIA | No |
| Rosendahl, 2017 ^457^ | Finland | 171 | 8 | CLIA | No |
| Ruelas, 2020 ^458^ | Colima, Mexico | 227 | 5 | ELISA | No |
| Rufus-Membere, 2019 ^459^ | Geelong, Australia | 357 | 8 | CLIA | No |
| Saberi-Karimian, 2023 ^460^ | Iran | 315 | 5 | HPLC | No |
| Sacheck, 2011 ^461^ | Boston, USA | 263 | 6 | RIA | No |
| Sadiq, 2018 ^462^ | Lahore, Pakistan | 858 | 6 | ECLIA | No |
| Saki, 2017 ^463^ | Shiraz, Iran | 477 | 8 | HPLC | No |
| Salem-Sokhn, 2021 ^464^ | North,Beirut, Mount Lebanon; Lebanon | 376 | 6 | CPBA | No |
| Sales, 2023 ^465^ | Sao Paulo, Brazil | 288 | 7 | CLIA | No |
| Samefors, 2014 ^466^ | Sweden | 333 | 6 | HPLC | No |
| Santos, 2019 ^467^ | Brazil | 420 | 8 | CLIA | No |
| Santos, 2017 ^468^ | Portugal | 1500 | 9 | ECLIA | No |
| Santos Araújo, 2017 ^469^ | Joao Pessoa, Brazil | 220 | 8 | CLIA | No |
| Sanwalka, 2013 ^470^ | Pune, India | 120 | 7 | RIA | No |
| Sari, 2021 ^471^ | Sumatra, Indonesia | 56 | 6 | ELISA | No |
| Sarma, 2019 ^472^ | Guwahati, India | 500 | 8 | RIA | No |
| Schramm, 2017 ^473^ | Germany | 4149 | 8 | ECLIA | No |
| Seo, 2012 ^474^ | Seoul, South Korea | 484 | 8 | CLIA | No |
| Seo, 2013 ^475^ | Ansan, South Korea | 799 | 8 | CLIA | No |
| Shah, 2014 ^476^ | Pakistan | 189 | 5 | N/A | No |
| Shaheen, 2011 ^477^ | Hertfordshire, UK | 2943 | 8 | CLIA | No |
| Shanyhin, 2022 ^478^ | Ukraine | 928 | 5 | N/A | No |
| Sharma, 2017 ^479^ | Britain, UK | 1556 | 10 | LC-MS/MS | No |
| Sharma, 2018 ^480^ | Lahaul ; Spiti, India | 105 | 8 | CLIA | No |
| Sheen, 2016 ^481^ | Seoul, South Korea | 473 | 6 | CLIA | No |
| Shen, 2017 ^482^ | Shanghai. China | 60 | 7 | RIA | No |
| Sherchand, 2022 ^483^ | Sunsari ; Morang, Nepal | 324 | 6 | CLIA | No |
| Sherief, 2021 ^484^ | Sharkia, Egypt | 572 | 7 | RIA | No |
| Shetty, 2014 ^485^ | India | 252 | 8 | CLIA | No |
| Shi, 2022 ^486^ | China | 54269 | 10 | HPLC | No |
| Shin, 2021 ^487^ | Seongnam, South Korea | 518 | 9 | CLIA | No |
| Shinkov, 2015 ^488^ | Bulgaria | 1952 | 8 | LC-MS/MS | No |
| Shridhar, 2019 ^489^ | Andhra Pradesh, India | 2699 | 7 | HPLC | No |
| Silva, 2021^490^ | Porto, Portugal | 335 | 6 | ECLIA | No |
| Singh, Baalasubramanian, Kalaivani et al., 2022 ^491^ | Jharkhand, India | 95 | 8 | LC-MS/MS | No |
| Singh, Jalan, Bhardwaj et al., 2022 ^492^ | Jodhpur, India | 200 | 6 | CLIA | No |
| Smith, 2016 ^493^ | Cambodia | 725 | 9 | ELISA | No |
| Soininen, 2016 ^494^ | Kuopio, Finland | 374 | 8 | CLIA | No |
| Sonderman, 2012 ^495^ | USA | 225 | 6 | CLIA | No |
| Song, Kim, Choi et al., 2014 ^496^ | Kanghwa; Gwangju, South Korea | 807 | 6 | RIA | No |
| Song, Kweon, Choi et al., 2014 ^497^ | Gwangju, South Korea | 8976 | 9 | CIMA | No |
| Soontrapa, 2015 ^498^ | Khon Kaen, Thailand | 66 | 6 | ECLIA | No |
| Souberbielle, 2016 ^499^ | France | 892 | 6 | CLIA | No |
| Sousa, 2019 ^500^ | Brazil | 153 | 7 | CLIA | No |
| Srimani, 2017 ^501^ | West Bengal, India | 59 | 8 | EIA | No |
| Standahl Olsen, 2013 ^502^ | Norway | 218 | 7 | HPLC | Yes |
| Stephan, 2023 ^503^ | USA | 5369 | 8 | LC-MS/MS | No |
| Su, 2020 ^504^ | China | 2790 | 8 | LC-MS/MS | No |
| Suárez-Calleja, 2021 ^505^ | Asturias, Spain | 138 | 7 | HPLC | No |
| Sulimani, 2016 ^506^ | Riyadh, Saudi Arabia | 2000 | 8 | ECLIA | Yes |
| Summerhays, 2020 ^507^ | Sweden | 4659 | 8 | CMIA | No |
| Sun, 2014 ^508^ | Tokyo, Japan | 107 | 6 | ELFA | No |
| Sundarakumar, 2021 ^509^ | Srinivaspura, India | 1546 | 7 | CLIA | No |
| Sung, 2022 ^510^ | Seongnam, South Korea | 432 | 6 | ELISA | No |
| Surve, Begum, Chauhan et al., 2021 ^511^ | Bombay, India | 201 | 6 | CLIA | No |
| Suryanarayana, 2018 ^512^ | India | 298 | 9 | RIA | No |
| Szabo, 2017 ^513^ | Hungary | 664 | 9 | CLIA | No |
| Szili, 2018 ^514^ | Hungary | 462 | 9 | CLIA | Yes |
| Szternel, 2018 ^515^ | Kujawsko-Pomorskie, Poland | 284 | 6 | CLIA | No |
| Talaei, 2012 ^516^ | Arak, Iran | 420 | 8 | RIA | No |
| Tamer, 2012 ^517^ | Turkey | 276 | 6 | RIA | No |
| Tandon, 2014 ^518^ | India | 312 | 6 | ECLIA | No |
| Teng, 2022 ^519^ | Anhui, China | 617 | 7 | LC-MS/MS | No |
| Terán, 2018 ^520^ | Bolivia | 203 | 6 | EIA | No |
| Terock, 2021 ^521^ | Northeast Germany | 5783 | 8 | IDS | No |
| Thompson, 2021 ^522^ | Tasmania, Australia | 1096 | 8 | RIA | Yes |
| Thorisdottir, 2016 ^523^ | Iceland | 139 | 8 | ECLIA | No |
| Thornton, 2013 ^524^ | Bogota, Colombia | 475 | 9 | CPBA | No |
| Tian, 2017 ^525^ | Gansu, China | 5981 | 8 | CLIA | No |
| Tokida, 2021 ^526^ | Obuse, Japan | 411 | 8 | ECLIA | No |
| Tolppanen, 2012 ^527^ | England, UK | 7560 | 8 | HPLC | Yes |
| Trummer, 2012 ^528^ | Austria | 1435 | 6 | ELISA | No |
| Tse, 2016 ^529^ | Nunavut, Canada | 279 | 6 | CLIA | Yes |
| Tsugawa, 2022 ^530^ | Kyoto, Japan | 39 | 6 | LC-MS/MS | No |
| Tuffaha, 2015 ^531^ | Saudi Arabia | 5004 | 7 | N/A | No |
| Tung, 2021 ^532^ | Hong Kong, China | 208 | 7 | LC-MS/MS | Yes |
| Vahid, 2023 ^533^ | Luxembourg | 1404 | 4 | N/A | No |
| Vajdi, 2020 ^534^ | Azarbaijian East, Iran | 525 | 8 | CLIA | No |
| VanSickle, 2020 ^535^ | USA | 74 | 6 | LC-MS/MS | No |
| Vázquez-Lorente, 2022 ^536^ | Granada, Spain | 78 | 5 | LC-MS/MS | No |
| Verhoeven, 2011 ^537^ | Antwerp, Belgium | 589 | 6 | N/A | No |
| Vignali, 2017 ^538^ | Pescopagano, Italy | 620 | 8 | RIA | No |
| Vioque, 2019 ^539^ | Valencia, Spain | 156 | 5 | HPLC | Yes |
| Voortman, 2015 ^540^ | Rotterdam, Netherlands | 4167 | 7 | LC-MS/MS | No |
| Wakayo, 2015 ^541^ | Adama, Ethiopia | 174 | 8 | LC-MS/MS | Yes |
| Wang, 2016 ^542^ | Shanghai, China | 2960 | 6 | ECLIA | No |
| Wang, 2014 ^543^ | Changsha, China | 516 | 6 | RIA | No |
| Wang, 2022 ^544^ | Xi’an, China | 455 | 6 | ECLIA | No |
| Wang, Chen, Zhang et al., 2019 ^545^ | Shanghai, China | 706 | 6 | LC-MS/MS | No |
| Wang, Sun, Ge et al., 2019 ^546^ | Henan, China | 484 | 6 | ELISA | No |
| Wang, Han, Wang et al., 2019 ^547^ | Zhengzhou; Luoyang; and Jiaozuo, China | 1559 | 6 | N/A | No |
| Wang, 2017 ^548^ | Anshan, China | 174 | 7 | ECLIA | No |
| Wang, 2015 ^549^ | China | 538 | 8 | RIA | No |
| Weiler, 2023 ^550^ | Canada | 21770 | 9 | CLIA | Yes |
| Weldegiorgis, 2020 ^551^ | Dalian, China | 2259 | 6 | ECLIA | No |
| Wolters, 2022 ^552^ | Europe | 3606 | 10 | CLIA | No |
| Wyskida, 2018 ^553^ | Poland | 3472 | 10 | ELISA | No |
| Xia, 2019 ^554^ | Beijing, China | 785 | 6 | RIA | No |
| Xiao, 2020 ^555^ | China | 6091 | 10 | CLIA | No |
| Xiao, 2022 ^556^ | Beijing, China | 10482 | 9 | CLIA | No |
| Xie, 2019 ^557^ | Xinjiang; Sichuan; Hunan; Guangdong; Shanghai; Beijing; and Heilongjiang, China | 1684 | 10 | LC-MS/MS | No |
| Xu, 2020 ^558^ | Shanghai, China | 861 | 6 | ECLIA | No |
| Yan, 2018 ^559^ | Shanghai, China | 4592 | 6 | ECLIA | No |
| Yan, 2019 ^560^ | Jinzhong, China | 302 | 7 | LC-MS/MS | No |
| Yang, 2022 ^561^ | Wuzhong; Shizuishan, China | 1893 | 8 | CLIA | No |
| Yang, 2016 ^562^ | South Korea | 3720 | 10 | CLIA | No |
| Yao, 2019 ^563^ | Hainan, China | 943 | 6 | RIA | No |
| Yao, 2014 ^564^ | Keelung, Taiwan | 1315 | 9 | ECLIA | Yes |
| Yeap, 2021 ^565^ | Selangor, Malayasia | 386 | 7 | LC-MS/MS | Yes |
| Yoshihara, 2014 ^566^ | Niigata, Japan | 674 | 6 | N/A | No |
| Yoshimura, 2013 ^567^ | Tokyo; Wakayama; Japan | 1638 | 6 | RIA | No |
| Younes, 2019 ^568^ | Lebanon | 974 | 6 | CLIA | No |
| Young, 2023 ^569^ | Afghanistan | 662 | 9 | CLIA | No |
|  | Afghanistan | 1044 | 9 | CLIA | No |
|  | Cambodia | 646 | 9 | ECLIA | No |
|  | Cambodia | 699 | 9 | ECLIA | No |
|  | Pakistan | 6943 | 9 | CLIA | No |
|  | Pakistan | 8387 | 8 | CLIA | No |
|  | Vietnam | 315 | 8 | CLIA | No |
|  | Vietnam | 528 | 8 | HPLC | No |
| Yousef, 2017 ^570^ | Jeddah, Saudi Arabia | 200 | 5 | N/A | No |
| Yuan, 2023 ^571^ | Shanghai; Beijing; Guangzhou; and Lanzhou, China | 2471 | 4 | HPLC | No |
| Yunara, 2020 ^572^ | Surabaya, Indonesia | 75 | 8 | CMIA | No |
| Zamoiski, 2014 ^573^ | Torreon, Mexico | 512 | 8 | CLIA | No |
| Zelzer, 2021 ^574^ | Graz, Austria | 390 | 8 | LC-MS/MS | No |
| Zhang, Cheng, Wang, 2021 ^575^ | Henan, China | 1714 | 8 | ELISA | No |
| Zhang, Chen, Duan, 2021 ^576^ | Sichuan; Guizhou; and Yunnan, China | 7926 | 8 | N/A | No |
| Zhang, 2016 ^577^ | Kuwait | 960 | 9 | ECLIA | Yes |
| Zhang, 2014 ^578^ | Harbin, China | 1488 | 8 | UPLC | No |
| Zhao, 2022 ^579^ | Lanzhou, China | 5543 | 8 | ELISA | No |
| Zhou, 2015 ^580^ | Adelaide, Australia | 221 | 8 | RIA | No |
| Zhou, 2022 ^581^ | Xinhua County in Hunan Province; Sansui County in Guizhou Province; and Gong’an County in Hubei Province, China | 672 | 5 | ELISA | No |
| Zhu, 2018 ^582^ | Shanghai, China | 508 | 8 | ELISA | No |
| Zhuang, 2021 ^583^ | Kadoori, China | 512726 | 6 | CLIA | Yes |
| Zou, 2021 ^584^ | Ali; Shigatse; Lhasa; Nyingch, Tibet | 1246 | 7 | ECLIA | No |
| Zumaraga, 2021 ^585^ | Metro Manila, Philippines | 833 | 9 | ECLIA | No |
| Zupo, 2023 ^586^ | Apulia, Italy | 479 | 5 | CLIA | No |

CBPA, competitive protein-binding assay; CLIA, chemiluminescence immunoassay; CMIA, chemiluminescent microparticle immunoassay; EIA, enzyme immunoassay; ECLIA, electrochemiluminescence immunoassay; ELFA, enzyme linked fluorescent assay; ELISA, enzyme-linked immunosorbent assay; HPLC, high performance liquid chromatography; N/A, Not available; LC-MS/MS, liquid chromatography with tandem mass spectrometry; RIA, radioimmunoassay; UPLC, Ultra-performance liquid chromatography.

# Bibliography*

1. Abdeen Z, Ramlawi Ad, Qaswari R, Alrub AA, Dary O, Rambeloson Z, et al. Predicted efficacy of the Palestinian wheat flour fortification programme: Complementary analysis of biochemical and dietary data. *Public Health Nutr*. 2015;18(8):1358-68.

2. Abidin NZ, Mitra SR. Total vs. bioavailable: Determining a better 25 (OH) D index in association with bone density and muscle mass in postmenopausal women. *Metabolites*. 2021;11(1):23.

3. Abrahão GP, Santos MC, Vieira Filho JPB, Dal Fabbro AL, Franco LJ, Moises RS. Serum 25-hydroxyvitamin D concentration and its association with glucose intolerance in an Indigenous population. *Clin Nutr*. 2021;40(3):1318-22.

4. Abu Shady MM, Youssef MM, Salah El-Din EM, Abdel Samie OM, Megahed HS, Salem SME, et al. Predictors of serum 25-Hydroxyvitamin D concentrations among a sample of Egyptian schoolchildren. *Scientific World Journal*. 2016;2016:8175768.

5. Acherjya GK, Ali M, Tarafder K, Yeasmin S. Hypovitaminosis D and its relationship with diabetes mellitus among the postmenopausal women in Jashore, Bangladesh. *Indian J Endocrinol Metab*. 2020;24(6):512-7.

6. Afkhami-Ardekani O, Afkhami-Ardekani A, Namiranian N, Afkhami-Ardekani M, Askari M. Prevalence and predictors of vitamin D insufficiency in adult population of

Yazd - The sun province in center of Iran. *Diabetes Metab Syndr*. 2019;13(5):2843-7.

7. Aggarwal A, Ram S, Garg A, Pal R, Bhansali A, Singh P, et al. Metabolic bone profile of healthy adult North Indian population from Chandigarh urban bone epidemiological study (CUBES). *Ind J Clin Biochem*. 2021;36(1):67-73.

8. Akman AO, Tumer L, Hasanoglu A, Ilhan M, Caycı B. Frequency of vitamin D insufficiency in healthy children between 1 and 16 years of age in Turkey. *Pediatr Int*. 2011;53(6):968-73.

9. Al Hafidh N, Aljammas E, Al-Kataan M. Vitamin D status and mitochondrial function in children. *J Med J*. 2020;54(4):189-96.

10. Al Shaikh A, Farahat F, Abaalkhail B, Kaddam I, Aseri K, Al Saleh Y, et al. Prevalence of obesity and overweight among school-aged children in Saudi Arabia and its association with vitamin D status. *Acta Biomed*. 2020;91(4):e2020133.

11. Al Shaikh AM, Abaalkhail B, Soliman A, Kaddam I, Aseri K, Al Saleh Y, et al. Prevalence of vitamin D deficiency and calcium homeostasis in Saudi children. *J Clin Res Pediatr Endocrinol*. 2016;8(4):461-7.

12. Al-Dabhani K, Tsilidis KK, Murphy N, Ward HA, Elliott P, Riboli E, et al. Prevalence of vitamin D deficiency and association with metabolic syndrome in a Qatari population. *Nutr Diabetes*. 2017;7(4):e263.

13. Al-Daghri NM, Sabico S, Al-Saleh Y, Al-Attas OS, Alnaami AM, AlRehaili MM, et al. Calculated adiposity and lipid indices in healthy Arab children as influenced by vitamin D status. *Journal of Clinical Lipidology*. 2016;10(4):775-81.

14. Al-Daghri NM, Yakout S, Sabico S, Wani K, Hussain SD, Aljohani N, et al. Establishing the prevalence of osteomalacia in Arab adolescents using biochemical markers of bone health. *Nutrients*. 2022;14(24):5354.

15. Al-Eisa ES, Alghadir AH, Gabr SA. Correlation between vitamin D levels and muscle fatigue risk factors based on physical activity in healthy older adults. *Clin Interv Aging*. 2016;11:513-22.

16. Al-Ghamdi MA, Lanham-New SA, Kahn JA. Differences in vitamin D status and calcium metabolism in Saudi Arabian boys and girls aged 6 to 18 years: Effects of age, gender, extent of veiling and physical activity with concomitant implications for bone health. *Public Health Nutr*. 2012;15(10):1845-53.

17. Al-Raddadi R, Bahijri S, Borai A, AlRaddadi Z. Prevalence of lifestyle practices that might affect bone health in relation to vitamin D status among female Saudi adolescents. *Nutrition*. 2018;45:108-13.

18. Al-Saleh Y, Al-Daghri NM, Khan N, Alfawaz H, Al-Othman AM, Alokail MS, et al. Vitamin D status in Saudi school children based on knowledge. *BMC Pediatr*. 2015;15(1):53.

19. Al-Sumaih I, Donnelly M, O’Neill C. The use of finite mixture models to examine the serum 25(OH)D levels among Saudis. *PLoS One*. 2021;16(11):e0260748.

20. Al-Taiar A, Rahman A, Al-Sabah R, Shaban L, Al-Harbi A. Vitamin D status among adolescents in Kuwait: A cross-sectional study. *BMJ Open*. 2018;8(7):e021401.

21. AlAnouti F, Ahmad AS, Wareth LA, Dhaheri AA, Oulhaj A, Junaibi AA, et al. Associations between serum 25-hydroxyvitamin D, body mass index and body fat composition among Emirati population: Results from the UAE healthy future study. *Front Endocrinol (Lausanne)*. 2022;13:954300.

22. Albalawi O, Almubark R, Almarshad A, Alqahtani AS. The prevalence of vitamin and mineral deficiencies and high levels of non-essential heavy metals in Saudi Arabian adults. *Healthcare*. 2022;10(12).

23. AlBuhairan FS, Tamim H, Al Dubayee M, AlDhukair S, Al Shehri S, Tamimi W, et al. Time for an adolescent health surveillance system in Saudi Arabia: Findings from “Jeeluna”. *Journal of Adolescent Health*. 2015;57(3):263-9.

24. ALbuloshi T, Kamel AM, Spencer JPE. Factors associated with low vitamin D status among older adults in Kuwait. *Nutrients*. 2022;14(16):3342.

25. Alghadir AH, Gabr SA, Al-Eisa ES. Mechanical factors and vitamin D deficiency in schoolchildren with low back pain: Biochemical and cross-sectional survey analysis. *J Pain Res*. 2017;10:855-65.

26. Ali P, Labriffe M, Navasiolava N, Custaud M-A, Dinomais M, Annweiler C, et al. Vitamin D concentration and focal brain atrophy in older adults: A voxel-based morphometric study. *Ann Clin Transl Neurol*. 2020;7(4):554-8.

27. Alyahya K, Lee WTK, Al-Mazidi Z, Morgan J, Lanham-New S. Risk factors of low vitamin D status in adolescent females in Kuwait: Implications for high peak bone mass attainment. *Arch Osteoporos*. 2014;9:178.

28. Amaliya A, Laine ML, Delanghe JR, Loos BG, Van Wijk AJ, Van der Velden U. Java project on periodontal diseases: Periodontal bone loss in relation to environmental and systemic conditions. *J Clin Periodontol*. 2015;42(4):325-32.

29. Amanzholkyzy A, Nurgalieva RE, Dosimov AZ, Stankevicius E, Kaldybaeva AT. Ethnic manifestations of gene polymorphisms of vitamin D receptor (VDR) in adolescents of Western Kazakhstan Region. *J Natl Med Assoc*. 2018;110(1):78-83.

30. Andersen S, Jakobsen A, Laurberg P. Vitamin D status in North Greenland is influenced by diet and season: Indicators of dermal 25-hydroxy vitamin D production north of the Arctic Circle. *Br J Nutr*. 2013;110(1):50-7.

31. Andersen R, Brot C, Jakobsen J, Mejborn H, Mølgaard C, Skovgaard LT, et al. Seasonal changes in vitamin D status among Danish adolescent girls and elderly women: The influence of sun exposure and vitamin D intake. *Eur J Clin Nutr*. 2013;67(3):270-4.

32. Andersen S, Noahsen P, Rex KF, Fleischer I, Albertsen N, Jørgensen ME, et al. Serum 25-hydroxyvitamin D, calcium and parathyroid hormone levels in Native and European populations in Greenland. *Br J Nutr*. 2018;119(4):391-7.

33. Ando E, Morisaki N, Asakura K, Sasaki S, Fujiwara T, Horikawa R. Serum 25-hydroxyvitamin D levels showed strong seasonality but lacked association with vitamin D intake in 3-year-old Japanese children. *Br J Nutr*. 2018;120(9):1034-44.

34. Angeles-Agdeppa I, Tanda KV. Vitamin D status and usual nutrient intake of Filipino children aged 6–12 years in selected areas in the Philippines: A 2018 National Nutrition Survey. *J Nutr Metab*. 2021;2021:8515607.

35. Antczak-Domagała K, Magierska J, Łucka A, Arabska J, Kłoszewska I, Sobów T, et al. Evaluation of the relationship between vitamin D concentration and cognitive performance in community dwelling elderly people. *J Clin Psychiatry*. 2019;19(1):19–24.

36. Ara G, Little DC, Mamun A-A, de Roos B, Grieve E, Khanam M, et al. Factors affecting the micronutrient status of adolescent girls living in complex agro-aquatic ecological zones of Bangladesh. *Sci Rep*. 2023;13(1):6631.

37. Arabi A, Baddoura R, El-Rassi R, El-Hajj Fuleihan G. PTH level but not 25 (OH) vitamin D level predicts bone loss rates in the elderly. *Osteoporos Int*. 2012;23(3):971-80.

38. Arabi A, Chamoun N, Nasrallah MP, Tamim HM. Vitamin D deficiency in Lebanese adults: Prevalence and predictors from a cross-sectional community-based study. *Int J Endocrinol*. 2021;2021:3170129.

39. Arazi H, Eghbali E. 25-Hydroxyvitamin D levels and its relation to muscle strength, maximal oxygen consumption, and body mass index in young and middle adulthood women. *Int J Womens Health*. 2019;11:57-64.

40. Arnljots R, Thorn J, Elm M, Moore M, Sundvall P-D. Vitamin D deficiency was common among nursing home residents and associated with dementia: A cross sectional study of 545 Swedish nursing home residents. *BMC Geriatr*. 2017;17(1):229.

41. Arora B, Patel SS, Saboo BD. The prevalence of prediabetes and associated conditions in Ahmedabad population. *Int J Diabetes Dev Ctries*. 2020;40:61-9.

42. Asakura K, Etoh N, Imamura H, Michikawa T, Nakamura T, Takeda Y, et al. Vitamin D status in Japanese adults: Relationship of serum 25-hydroxyvitamin D with simultaneously measured dietary vitamin D intake and ultraviolet ray exposure. *Nutrients*. 2020;12(3):743.

43. Asante EO, Mai X-M, Eldholm RS, Skjellegrind HK, Kolberg M, Brumpton BM, et al. Vitamin D status over time and cognitive function in Norwegian older adults: A prospective cohort of the HUNT study. *J Nutr Health Aging*. 2023;27(1):30-7.

44. Ashraf AP, Fisher G, Alvarez J, Dudenbostel T, Calhoun DA, Szalai AJ, et al. Associations of C-reactive protein to indices of vascular health and the influence of serum 25(OH)D status in healthy adults. *J Nutr Metab*. 2012;2012:475975.

45. Aspelund T, Grübler MR, Smith AV, Gudmundsson EF, Keppel M, Cotch MF, et al. Effect of genetically low 25-hydroxyvitamin D on mortality risk: Mendelian randomization analysis in 3 large European cohorts. *Nutrients*. 2019;11(1):74.

46. Au LE, Economos CD, Goodman E, Must A, Chomitz VR, Sacheck JM. Vitamin D intake and serum vitamin D in ethnically diverse urban schoolchildren. *Public Health Nutr*. 2012;15(11):2047-53.

47. Augusto RA, Cobayashi F, Cardoso MA. Associations between low consumption of fruits and vegetables and nutritional deficiencies in Brazilian schoolchildren. *Public Health Nutr*. 2015;18(5):927-35.

48. Awasthi S, Kumar D, Mahdi AA, Agarwal GG, Pandey AK, Parveen H, et al. Prevalence of specific micronutrient deficiencies in urban school going children and adolescence of India: A multicenter cross-sectional study. *PLoS One*. 2022;17(5):e0267003.

49. Azmathullah AB, Kirubharan S, Jeyasubramanian L, Anbarasan P, Kandasamy V, Natesan S, et al. Study of vitamin D levels in postmenopausal women. *J Evolution Med Dent Sci*. 2016;5(66):4740-4.

50. Bachhel R, Singh NR, Sidhu JS. Prevalence of vitamin D deficiency in North-west Punjab population: A cross-sectional study. *Int J Appl Basic Med Res*. 2015;5(1):7-11.

51. Bacon CJ, Kerse N, Hayman KJ, Moyes SA, Teh RO, Kepa M, et al. Vitamin D status of Māori and non-Māori octogenarians in New Zealand: A Cohort Study (LiLACS NZ). *Asia Pac J Clin Nutr*. 2016;25(4):885-97.

52. Baek HS, Won HY, Kim JH, Ha EK, Jee HM, Shin YH, et al. Association of phthalate exposure and airway dysfunction with mediation by serum periostin. *Pediatr Allergy Immunol*. 2021;32(8):1681-90.

53. Bano G, Pigozzo S, Piovesan F, Mazzochin M, Dianin M, Bedogni M, et al. Influence of serum 25-hydroxyvitamin D levels, fat-free mass, and fat mass on bone density, geometry and strength, in healthy young and elderly adults. *Exp Gerontol*. 2018;113:193-8.

54. Bansal N, Zelnick L, Robinson-Cohen C, Hoofnagle AN, Ix JH, Lima JA, et al. Serum parathyroid hormone and 25-hydroxyvitamin D concentrations and risk of incident heart failure: The Multi-Ethnic Study of Atherosclerosis. *J Am Heart Assoc*. 2014;3(6):e001278.

55. Barth-Jaeggi T, Zandberg L, Bahruddinov M, Kiefer S, Rahmarulloev S, Wyss K. Nutritional status of Tajik children and women: Transition towards a double burden of malnutrition. *Matern Child Nutr*. 2020;16(2):e12886.

56. Basińska-Lewandowska M, Lewiński A, Horzelski W, Skowrońska-Jóźwiak E. Effect of summer sunshine exposure on vitamin D status in young and middle age poles: Is 30 ng/mL vitamin D cut-off really suitable for the Polish population? *Int J Environ Res Public Health*. 2021;18(15):8116.

57. Batieha A, Khader Y, Jaddou H, Hyassat D, Batieha Z, Khateeb M, et al. Vitamin D status in Jordan: Dress style and gender discrepancies. *Ann Nutr Metab*. 2011;58(1):10-8.

58. Beer RJ, Herrán OF, Villamor E. Prevalence and correlates of vitamin D deficiency in a tropical setting: Results from a nationally representative survey. *Am J Clin Nutr*. 2020;112(4):1088-98.

59. Bennouar S, Bachir Cherif A, Makrelouf M, Ait Abdelkader B, Taleb A, Abdi S. Reconsidering vitamin D optimal values based on parathyroid hormone levels in a North Algerian cohort: Stratification by gender and season. *Arch Osteoporos*. 2022;17(1):100.

60. Berry DJ, Hesketh K, Power C, Hyppönen E. Vitamin D status has a linear association with seasonal infections and lung function in British adults. *Br J Nutr*. 2011;106(9):1433-40.

61. Beydoun MA, Ng AE, Fanelli-Kuczmarski MT, Hossain S, Beydoun HA, Evans MK, et al. Vitamin D status and its longitudinal association with changes in patterns of sleep among middle-aged urban adults. *Journal of Affective Disorders*. 2021;282:858-68.

62. Bhat KA, Kakaji M, Awasthi A, Shukla M, Dubey M, Srivastava R, et al. High prevalence of osteoporosis and morphometric vertebral fractures in Indian males aged 60 years and above: Should age for screening be lowered? *J Clin Densitom*. 2018;21(4):517-23.

63. Bhatt SP, Misra A, Sharma M, Guleria R, Pandey RM, Luthra K, et al. Vitamin D insufficiency is associated with abdominal obesity in urban Asian Indians without diabetes in North India. *Diabetes Technol Ther*. 2014;16(6):392-7.

64. Bhattacharjee R, Biswas D, Pandit K, Chatterjee P, Chowdhury S, Ghosh S, et al. A study to compare the metabolic health anthropologic and biochemical between scheduled tribe and non-scheduled tribe population in underdeveloped parts in the district of Birbhum, West Bengal: A population based observational study. *J Indian Med Assoc*. 2019;117(11):33-5.

65. Biben V, Defi IR, Nugraha GI, Setiabudiawan B. Vitamin D status and its impact on body composition in elderly community-dwelling individuals in Bandung and Sumedang, West Java Province, Indonesia. *Asian J Epidemiol*. 2017;10(2):63-9.

66. Bilinski WJ, Szternel L, Siodmiak J, Paradowski PT, Domagalski K, Sypniewska G. Low serum 25-hydroxyvitamin D level does not adversely affect bone turnover in prepubertal children. *Nutrients*. 2021;13(10):3324.

67. Binobead MA, Al-Qahtani WH, Al Bader NA, AlSedairy SA, Arzoo S. Prevalence of vitamin D deficiency and the effect of anthropometric and lifestyle factors on vitamin D status in healthy women residing in Riyadh. *Progr Nutr*. 2019;21(2):299-308.

68. Binu AJ, Cherian KE, Kapoor N, Jebasingh FK, Asha HS, Paul TV. Bone health after fifth decade in rural ambulatory South Indian postmenopausal women. *Indian J Community Med*. 2019;44(3):205-8.

69. Blomberg M, Rifas-Shiman SL, Camargo CAJ, Gold DR, Asgari MM, Thyssen JP, et al. Low maternal prenatal 25-hydroxy vitamin D blood levels are associated with childhood atopic dermatitis. *J Invest Dermatol*. 2017;137(6):1380-4.

70. Bogazzi F, Rossi G, Lombardi M, Tomisti L, Sardella C, Manetti L, et al. Vitamin D status may contribute to serum insulin-like growth factor I concentrations in healthy subjects. *J Endocrinol Invest*. 2011;34(8):e200-e3.

71. Bojar I, Raczkiewicz D, Sarecka-Hujar B. Depression, metabolic syndrome, serum TSH, and vitamin D concentrations in rural and urban postmenopausal women. *Medicina (Kaunas)*. 2020;56(10):511.

72. Bonakdaran S, Fakhraee F, Karimian MS, Mirhafez SR, Rokni H, Mohebati M, et al. Association between serum 25-hydroxyvitamin D concentrations and prevalence of metabolic syndrome. *Adv Med Sci*. 2016;61(2):219-23.

73. Borissova AM, Shinkov A, Vlahov J, Dakovska L, Todorov T, Svinarov D, et al. Vitamin D status in Bulgaria—Winter data. *Arch Osteoporos*. 2013;8:133.

74. Boucher-Berry C, Speiser PW, Carey DE, Shelov SP, Accacha S, Fennoy I, et al. Vitamin D, osteocalcin, and risk for adiposity as comorbidities in middle school children. *Journal of Bone and Mineral Research*. 2012;27(2):283-93.

75. Bowman GL, Silbert LC, Howieson D, Dodge HH, Traber MG, Frei B, et al. Nutrient biomarker patterns, cognitive function, and MRI measures of brain aging. *Neurology*. 2012;78(4):241-9.

76. Braithwaite VS, Jones KS, Schoenmakers I, Silver M, Prentice A, Hennig BJ. Vitamin D binding protein genotype is associated with plasma 25OHD concentration in West African children. *Bone*. 2015;74:166-70.

77. Buchebner D, Bartosch P, Malmgren L, McGuigan FE, Gerdhem P, Akesson KE. Association between vitamin D, frailty, and progression of frailty in community-dwelling older women. *J Clin Endocrinol Metab*. 2019;104(12):6139-47.

78. Buchmann N, Eckstein N, Spira D, Demuth I, Steinhagen-Thiessen E, Norman K. Vitamin D insufficiency is associated with metabolic syndrome independent of insulin resistance and obesity in young adults - The Berlin Aging Study II. *Diabetes Metab Res Rev*. 2021;37(8):e3457.

79. Cabral M, Araújo J, Lopes C, Barros H, Guimarães JT, Severo M, et al. Relationship between dietary vitamin D and serum 25-hydroxyvitamin D levels in Portuguese adolescents. *Public Health Nutr*. 2018;21(2):325-32.

80. Cai M, Wang Y, Liu T, Huang Y. Interaction between vitamin D status and calcium intake in association with blood pressure and hypertension. *Journal of Bone and Mineral Research*. 2023;69(2):81-9.

81. Cairncross C, Grant C, Stonehouse W, Conlon C, McDonald B, Houghton L, et al. The relationship between vitamin D status and allergic diseases in New Zealand preschool children. *Nutrients*. 2016;8(6):326.

82. Cantio E, Bilenberg N, Nørgaard SM, Beck IH, Möller S, Cantio C, et al. Vitamin D status in pregnancy and childhood associates with intelligence quotient at age 7 years: An Odense child cohort study. *Aust N Z J Psychiatry*. 2023;57(7):1062-72.

83. Capuano R, Marchese F, Sica R, Capuano E, Manilia M, Iannone AG, et al. Epidemiologic data of vitamin D deficiency and its implication in cardio-cerebrovascular risk in a Southern Italian population. *J Nutr Metab*. 2021;2021:5550222.

84. Carrelli AL, Walker MD, Lowe H, McMahon DJ, Rundek T, Sacco RL, et al. Vitamin D deficiency is associated with subclinical carotid atherosclerosis: The Northern Manhattan study. *Stroke*. 2011;42(8):2240-5.

85. Carrillo-Vega MF, García-Peña C, Gutiérrez-Robledo LM, Pérez-Zepeda MU. Vitamin D deficiency in older adults and its associated factors: A cross-sectional analysis of the Mexican Health and Aging Study. *Arch Osteoporos*. 2017;12(1):8.

86. Casey C, Woodside JV, McGinty A, Young IS, McPeake J, Chakravarthy U, et al. Factors associated with serum 25-hydroxyvitamin D concentrations in older people in Europe: The EUREYE study. *Eur J Clin Nutr*. 2019;73(2):319-28.

87. Cashman KD, Kehoe L, Kearney J, McNulty B, Walton J, Flynn A. Adequacy of calcium and vitamin D nutritional status in a nationally representative sample of Irish teenagers aged 13-18 years. *Eur J Nutr*. 2022;61(8):4001-14.

88. Cashman KD, Muldowney S, McNulty B, Nugent A, FitzGerald AP, Kiely M, et al. Vitamin D status of Irish adults: Findings from the National Adult Nutrition Survey. *Br J Nutr*. 2013;109(7):1248-56.

89. Castillo-Valenzuela O, Duarte L, Arredondo M, Iñiguez G, Villarroel L, Pérez-Bravo F. Childhood obesity and plasma micronutrient deficit of Chilean children between 4 and 14 years old. *Nutrients*. 2023;15(7):1707.

90. Cediel G, Corvalán C, López de Romaña D, Mericq V, Uauy R. Prepubertal adiposity, vitamin D status, and insulin resistance. *Pediatrics*. 2016;138(1):e20160076.

91. Chailurkit L-o, Aekplakorn W, Ongphiphadhanakul B. The association between vitamin D status and type 2 diabetes in a Thai population, a cross-sectional study. *Clinical Endocrinology*. 2012;77(5):658-64.

92. Chan R, Chan D, Woo J, Ohlsson C, Mellström D, Kwok T, et al. Not all elderly people benefit from vitamin D supplementation with respect to physical function: Results from the Osteoporotic Fractures in Men Study, Hong Kong. *J Am Geriatr Soc*. 2012;60(2):290-5.

93. Chaudhry AB, Hajat S, Rizkallah N, Abu-Rub Aa. Risk factors for vitamin A and D deficiencies among children under-five in the state of Palestine. *Confl Health*. 2018;12:13.

94. Checkley W, Robinson CL, Baumann LM, Hansel NN, Romero KM, Pollard SL, et al. 25-hydroxy vitamin D levels are associated with childhood asthma in a population-based study in Peru. *Clin Exp Allergy*. 2015;45(1):273-82.

95. Chen C-H, Liu L-K, Chen M-J, Lee W-J, Lin M-H, Peng L-N, et al. Associations between vitamin D deficiency, musculoskeletal health, and cardiometabolic risk among community-living people in Taiwan: Age and sex-specific relationship. *Medicine (Baltimore)*. 2018;97(52):e13886.

96. Chen F, Wang Y, Guo Y, Wang J, Yang A, Lv Q, et al. Specific higher levels of serum uric acid might have a protective effect on bone mineral density within a Chinese population over 60 years old: A cross-sectional study from northeast China. *Clin Interv Aging*. 2019;14:1065-73.

97. Chen J, Yun C, He Y, Piao J, Yang L, Yang X. Vitamin D status among the elderly Chinese population: A cross-sectional analysis of the 2010–2013 China national nutrition and health survey (CNNHS). *Nutrition Journal*. 2017;16:3.

98. Chen L-W, Chien C-Y, Hsieh C-W, Chang L-C, Huang M-H, Huang W-Y, et al. The associations between Helicobacter pylori infection, serum vitamin D, and metabolic syndrome: A community-based study. *Medicine (Baltimore)*. 2016;95(18):e3616.

99. Chen W, Zhang X, Wang H, Zhang W, Xu Y, Zheng S. The epidemic investigation of serum 25-hydroxy vitamin D levels in the adults in Qujing area of Yunnan province in China. *International Journal of Clinical and Experimental Pathology*. 2015;8(8):9597-601.

100. Cheong M, Chew STH, Oliver J, Baggs G, Low YL, How CH, et al. Nutritional biomarkers and associated factors in community-dwelling older adults: Findings from the SHIELD study. *Nutrients*. 2020;12(11):3329.

101. Chiang D, Kramer H, Luke A, Cooper R, Aloia J, Bovet P, et al. 25-Hydroxyvitamin D and blood pressure: A plateau effect in adults with African ancestry living at different latitudes. *J Hypertens*. 2017;35(5):968-74.

102. Chlebna-Sokół D, Konstantynowicz J, Abramowicz P, Kulik-Rechberger B, Niedziela M, Obuchowicz A, et al. Evidence of a significant vitamin D deficiency among 9-13-year-old Polish children: Results of a multicentre study. *Eur J Nutr*. 2019;58(5):2029-36.

103. Choi DP, Oh SM, Lee J-M, Cho HM, Lee WJ, Song B-M, et al. Serum 25-hydroxyvitamin D and insulin resistance in apparently healthy adolescents. *PLoS One*. 2014;9(7):e103108.

104. Choi HR, Lee SW, Yeom H, Jeon D-H, Kim HC, Youm Y. Association between vitamin D status and asymmetric dimethylarginine (ADMA) concentration in the Korean elderly population. *Maturitas*. 2017;102:13-7.

105. Chua EY, Mohd Shariff Z, Sulaiman N, Appannah G, Yong HY. Associations of serum 25-Hydroxyvitamin D with adiposity and at-risk lipid profile differ for Indigenous (Orang Asli) male and female adults of Peninsular Malaysia. *Int J Environ Res Public Health*. 2020;17(8).

106. Chuang S-C, Chen H-L, Tseng W-T, Wu I-C, Hsu C-C, Chang H-Y, et al. Circulating 25-hydroxyvitamin D and physical performance in older adults: A nationwide study in Taiwan. *Am J Clin Nutr*. 2016;104(5):1334-44.

107. Chuc DV, Hung NX, Trang VT, Linh DV, Khue PM. Nutritional status of children aged 12 to 36 months in a rural district of Hungyen Province, Vietnam. *Biomed Res Int*. 2019;2019:6293184.

108. Cirillo M, Bilancio G, Cavallo P, Costanzo S, De Curtis A, Di Castelnuovo A, et al. Correlates of calcidiol deficiency in adults - Cross-sectional, observational, population-based study. *Nutrients*. 2022;14(3):459.

109. Clark P, Montiel-Ojeda D, Chico-Barba LG, López-González D, Méndez-Sánchez L, Guagnelli-Martínez MA. Vitamin D concentration and its association with parathyroid hormone in children and adolescents. *Bol Med Hosp Infant Mex*. 2021;78(4):265-72.

110. Cobayashi F, Lourenço BH, Cardoso MA. 25-Hydroxyvitamin D₃ levels, BsmI polymorphism and insulin resistance in Brazilian Amazonian children. *Int J Mol Sci*. 2015;16(6):12531-46.

111. Contreras-Manzano A, Mejía-Rodríguez F, Villalpando S, Rebollar R, Flores-Aldana M. Vitamin D status in Mexican women at reproductive age, Ensanut 2018-19. *Salud Publica Mex*. 2021;63(3 May-Jun):394-400.

112. Conzade R, Koenig W, Heier M, Schneider A, Grill E, Peters A, et al. Prevalence and predictors of subclinical micronutrient deficiency in German older adults: Results from the population-based KORA-age study. *Nutrients*. 2017;9(12):1276.

113. Courraud J, Quist JS, Kontopodi E, Blomberg Jensen M, Bjerrum PJ, Helge JW, et al. Dietary habits, metabolic health and vitamin D status in Greenlandic children. *Public Health Nutr*. 2020;23(5):904-13.

114. Cremers E, Thijs C, Penders J, Jansen E, Mommers M. Maternal and child's vitamin D supplement use and vitamin D level in relation to childhood lung function: The KOALA Birth Cohort Study. *Thorax*. 2011;66(6):474-80.

115. Croll PH, Boelens M, Vernooij MW, van de Rest O, Zillikens MC, Ikram MA, et al. Associations of vitamin D deficiency with MRI markers of brain health in a community sample. *Clin Nutr*. 2021;40(1):72-8.

116. Cui A, Xiao P, Ma Y, Fan Z, Zhou F, Zheng J, et al. Prevalence, trend, and predictor analyses of vitamin D deficiency in the US population, 2001-2018. *Frontiers in Nutrition*. 2022;9:965376.

117. da Silva JRD, Freire IV, Ribeiro ÍJS, Dos Santos CS, Casotti CA, Dos Santos DB, et al. Improving the comprehension of sarcopenic state determinants: An multivariate approach involving hormonal, nutritional, lifestyle and genetic variables. *Mech Ageing Dev*. 2018;173:21-8.

118. Dalgård C, Petersen MS, Weihe P, Grandjean P. Vitamin D status in relation to glucose metabolism and type 2 diabetes in septuagenarians. *Diabetes Care*. 2011;34(6):1284-8.

119. Das S, Hasan MM, Mohsin M, Jeorge DH, Rasul MG, Khan A-R, et al. Sunlight, dietary habits, genetic polymorphisms and vitamin D deficiency in urban and rural infants of Bangladesh. *Sci Rep*. 2022;12(1):3623.

120. Davarzani S, Djafarian K, Clark CCT, Babaei N, Ghorbaninejad P, Ebaditabar M, et al. The interaction of aging with serum 25(OH)D and 1,25(OH)2 D status on muscle strength. *Int J Clin Pract*. 2021;75(10):e14510.

121. de Koning EJ, Verweij L, Lips P, Beekman ATF, Comijs HC, van Schoor NM. The relationship between serum 25(OH)D levels and anxiety symptoms in older persons: Results from the Longitudinal Aging Study Amsterdam. *J Psychosom Res*. 2017;97:90-5.

122. De la Cruz-Góngora V, Salinas-Rodríguez A, Flores-Aldana M, Villalpando S. Etiology of anemia in older Mexican adults: The role of hepcidin, vitamin A and vitamin D. *Nutrients*. 2021;13(11):3814.

123. De la Cruz-Góngora V, Salinas-Rodríguez A, Villalpando S, Flores-Aldana M. Serum retinol but not 25 (OH) D status is associated with serum hepcidin levels in older Mexican adults. *Nutrients*. 2019;11(5):988.

124. de Menezes-Júnior LAA, Sabião TdS, de Moura SS, Batista AP, de Menezes MC, Carraro JCC, et al. Influence of sunlight on the association between 25-hydroxyvitamin D levels and sleep quality in Brazilian adults: A population-based study. *Nutrition*. 2023;110:112008.

125. de Oliveira CL, Cureau FV, Cople-Rodrigues CDS, Giannini DT, Bloch KV, Kuschnir MCC, et al. Prevalence and factors associated with hypovitaminosis D in adolescents from a sunny country: Findings from the ERICA survey. *J Steroid Biochem Mol Biol*. 2020;199:105609.

126. de Oliveira DL, Dokkedal-Silva V, Fernandes GL, Kim LJ, Tufik S, Andersen ML. Sleep duration as an independent factor associated with vitamin D levels in the EPISONO cohort. *J Clin Sleep Med*. 2021;17(12):2439-49.

127. Del Brutto OH, Mera RM, Macias J, Morales G, Zambrano M. Cerebrovascular correlates of vitamin D deficiency in older adults living near the equator: Results from the Atahualpa Project. *Int J Stroke*. 2015;10(8):1301-3.

128. Delinocente MLB, Luiz MM, de Oliveira DC, de Souza AF, Ramírez PC, de Oliveira Máximo R, et al. Are serum 25-hydroxyvitamin D deficiency and insufficiency risk factors for the incidence of dynapenia? *Calcif Tissue Int*. 2022;111(6):571-9.

129. Delshad M, Beck KL, Conlon CA, Mugridge O, Kruger MC, Jensen BP, et al. Wintertime vitamin D status and its related risk factors among children living in Auckland, New Zealand. *N Z Med J*. 2019;132(1504):67-76.

130. Deng J, Guo S, Liu X, Jiang Y. The interaction between vitamin D and diets on serum lipids in Chinese Han adolescents. *Clin Lab*. 2022;68(1).

131. Denova-Gutiérrez E, Muñoz-Aguirre P, López D, Flores M, Medeiros M, Tamborrel N, et al. Low serum vitamin D concentrations are associated with insulin resistance in Mexican children and adolescents. *Nutrients*. 2019;11(9):2109.

132. Dhore R, Wasnik VR. Vitamin D status of apparently healthy early adolescents in Amravati City of Maharashtra, India. *Int J Collab Res Intern Med Public Health*. 2013;5(9):608-18.

133. Diederichsen SZ, Grønhøj MH, Mickley H, Gerke O, Steffensen FH, Lambrechtsen J, et al. CT-detected growth of coronary artery calcification in asymptomatic middle-aged subjects and association with 15 biomarkers. *JACC Cardiovasc Imaging*. 2017;10(8):858-66.

134. Diekmann R, Winning K, Bauer JM, Uter W, Stehle P, Lesser S, et al. Vitamin D status and physical function in nursing home residents: A 1-year observational study. *Z Gerontol Geriatr*. 2013;46(5):403-9.

135. Dimakopoulos I, Magriplis E, Mitsopoulou A-V, Karageorgou D, Bakogianni I, Micha R, et al. Association of serum vitamin D status with dietary intake and sun exposure in adults. *Clin Nutr ESPEN*. 2019;34:23-31.

136. Divanoglou N, Komninou D, Stea EA, Argiriou A, Papatzikas G, Tsakalof A, et al. Association of vitamin D receptor gene polymorphisms with serum vitamin D levels in a greek rural population (Velestino Study). *Lifestyle Genom*. 2021;14(3):81-90.

137. Djennane M, Lebbah S, Roux C, Djoudi H, Cavalier E, Souberbielle JC. Vitamin D status of schoolchildren in Northern Algeria, seasonal variations and determinants of vitamin D deficiency. *Osteoporos Int*. 2014;25(5):1493-502.

138. Dogan-Sander E, Mergl R, Willenberg A, Baber R, Wirkner K, Riedel-Heller SG, et al. Inflammation and the association of vitamin D and depressive symptomatology. *Nutrients*. 2021;13(6):1972.

139. Dong C, Ge P, Ren X, Zhao X, Wang J, Fan H, et al. The micronutrient status of children aged 24–60 months living in rural disaster areas one year after the wenchuan earthquake. *PLoS One*. 2014;9(2):e88444.

140. Drali O, Arab M, Lamdjadani N, Guechi Z, Berrah H. Vitamin D status in preschool children in Algeria. *Archives de Pédiatrie*. 2021;28(3):215-21.

141. Duarte C, Carvalheiro H, Rodrigues AM, Dias SS, Marques A, Santiago T, et al. Prevalence of vitamin D deficiency and its predictors in the Portuguese population: A nationwide population-based study. *Arch Osteoporos*. 2020;15(1):36.

142. Ebrahimi M, Khashayar P, Keshtkar A, Etemad K, Dini M, Mohammadi Z, et al. Prevalence of vitamin D deficiency among Iranian adolescents. *J Pediatr Endocrinol Metab*. 2014;27(7-8):595-602.

143. Egeland GM, Johnson-Down L, Cao ZR, Sheikh N, Weiler H. Food insecurity and nutrition transition combine to affect nutrient intakes in Canadian Arctic communities. *J Nutr*. 2011;141(9):1746-53.

144. El Badawy AA, Aboserea MM, El Seifi OS, Mortada EM, Bakry HM, Waly EH, et al. Vitamin D, parathormone and associated minerals among students in Zagazig district, Sharkia Governorate, Egypt. *Int J Vitam Nutr Res*. 2014;84(3-4):0173-82.

145. El-Khateeb M, Khader Y, Batieha A, Jaddou H, Hyassat D, Khawaja N, et al. Vitamin D deficiency and associated factors in Jordan. *SAGE Open Med*. 2019;7:2050312119876151.

146. El Maataoui A, Biaz A, El Machtani S, Bouhsain S, Dami A, El Maghraoui A, et al. Vitamin D status in healthy Moroccan men and women aged 50 years and older: A cross-sectional study. *Arch Osteoporos*. 2016;11(1):24.

147. Ellul S, Marx W, Collier F, Saffery R, Tang M, Burgner D, et al. Plasma metabolomic profiles associated with infant food allergy with further consideration of other early life factors. *Prostaglandins Leukot Essent Fatty Acids*. 2020;159:102099.

148. Emmerson AJB, Dockery KE, Mughal MZ, Roberts SA, Tower CL, Berry JL. Vitamin D status of White pregnant women and infants at birth and 4 months in North West England: A cohort study. *Matern Child Nutr*. 2018;14(1):e12453.

149. Erasmus R, Maepa S, Machingura I, Davids S, Raghubeer S, Matsha T. Vitamin D, vitamin D-binding proteins, and VDR polymorphisms in individuals with hyperglycaemia. *Nutrients*. 2022;14(15):3147.

150. Erdönmez D, Hatun S, Çizmecioğlu FM, Keser A. No relationship between vitamin D status and insulin resistance in a group of high school students. *J Clin Res Pediatr Endocrinol*. 2011;3(4):198-201.

151. Ewendt F, Schmitt M, Kluttig A, Kühn J, Hirche F, Kraus FB, et al. Association between vitamin D status and eryptosis–results from the German National Cohort Study. *Annals of Hematology*. 2023;102(6):1351-61.

152. Fabian E, Bogner M, Kickinger A, Wagner KH, Elmadfa I. Vitamin status in elderly people in relation to the use of nutritional supplements. *J Nutr Health Aging*. 2012;16(3):206-12.

153. Farber PL, Dias A, Freitas T, Pinho AC, Viggiano D, Saldanha C, et al. Evaluation of hemorheological parameters as biomarkers of calcium metabolism and insulin resistance in postmenopausal women. *Clinical Hemorheology and Microcirculation*. 2021;77(4):395-410.

154. Fassula AS, Gonzalez-Chica D, Giehl MC, Silva DAS, Cembranel F, Moreno YMF. Moderator role of vitamin D concentrations on the association between metabolic syndrome and C-reactive protein among adults. *Arch Endocrinol Metab*. 2021;64(6):695-703.

155. Feehan O, Armstrong DJ, Magee PJ, Pourshahidi LK, Strain JJ, Beggan L, et al. Vitamin D and bone health of older adults within care homes: An observational study. *Nutrients*. 2022;14(13):2680.

156. Flores ME, Rivera-Pasquel M, Valdez-Sánchez A, De la Cruz-Góngora V, Contreras-Manzano A, Shamah-Levy T, et al. Vitamin D status in Mexican children 1 to 11 years of age: An update from the Ensanut 2018-19. *Salud Publica Mex*. 2021;63(3 May-Jun):382-93.

157. Forney LA, Dietrich MA, Johannsen NM, Henagan TM, Tuuri G, Nelson AG, et al. Emerging relationships between vitamin D status, physical activity habits, and immune indices in college-aged females. *Int J Vitam Nutr Res*. 2017;87(1-2):49-58.

158. Fox FAU, Koch L, Breteler MMB, Ahmad Aziz N. 25-hydroxyvitamin D level is associated with greater grip strength across adult life span: A population-based cohort study. *Endocrine Connections*. 2023;12(4):e220501.

159. Galeazzi T, Quattrini S, Pjetraj D, Gatti S, Monachesi C, Franceschini E, et al. Vitamin D status in healthy Italian school-age children: A single-center cross-sectional study. *Italian Journal of Pediatrics*. 2023;49(1):27.

160. Ganie MA, Sahar T, Wani I, Rashid A, Robbani I, Nisar S, et al. Vitamin D status among Kashmiri tribal population: A cross-sectional community-based study. *Int J Environ Res Public Health*. 2022;156(2):348-56.

161. Gannagé-Yared MH, Sabbagh R, Chédid R. Relationship between 25 hydroxyvitamin D and lipid profile in Lebanese school children. *J Endocrinol Invest*. 2018;41(9):1043-9.

162. Gao C, Qiao J, Li SS, Yu WJ, He JW, Fu WZ, et al. The levels of bone turnover markers 25(OH)D and PTH and their relationship with bone mineral density in postmenopausal women in a suburban district in China. *Osteoporos Int*. 2017;28(1):211-8.

163. García-Dorta A, Medina-Vega L, Villacampa-Jiménez JJ, Hernández-Díaz M, Bustabad-Reyes S, González-Dávila E, et al. Baseline levels of vitamin D in a healthy population from a region with high solar irradiation. *Nutrients*. 2021;13(5):1647.

164. Garg S, Dasgupta A, Maharana SP, Paul B, Bandyopadhyay L, Bhattacharya A. Sun exposure and vitamin D in rural India: A cross-sectional study. *Indian J Public Health*. 2018;62(3):175-81.

165. Garg MK, Tandon N, Marwaha RK, Menon AS, Mahalle N. The relationship between serum 25-hydroxy vitamin D, parathormone and bone mineral density in Indian population. *Clinical Endocrinology*. 2014;80(1):41-6.

166. Ge H, Sun H, Wang T, Liu X, Li X, Yu F, et al. The association between serum 25-hydroxyvitamin D₃ concentration and serum lipids in the rural population of China. *Lipids Health Dis*. 2017;16:215.

167. Gebreegziabher T, Stoecker BJ. Vitamin D insufficiency in a sunshine-sufficient area: Southern Ethiopia. *Food Nutr Bull*. 2013;34(4):429-33.

168. Ghobadi S, Rostami ZH, Marzijarani MS, Faghih S. Association of vitamin D status and metabolic syndrome components in Iranian children. *Int J Prev Med*. 2019;10:77.

169. Giallauria F, Milaneschi Y, Tanaka T, Maggio M, Canepa M, Elango P, et al. Arterial stiffness and vitamin D levels: The Baltimore longitudinal study of aging. *J Clin Endocrinol Metab*. 2012;97(10):3717-23.

170. Gill TK, Hill CL, Shanahan EM, Taylor AW, Appleton SL, Grant JF, et al. Vitamin D levels in an Australian population. *BMC Public Health*. 2014;14(1):1001.

171. Gómez Alonso C, Díaz López JB, Rodríguez Rebollar A, Martínez Arias L, Martín Vírgala J, Martín Carro B, et al. Calcidiol levels and muscle function maintenance, functional capacity and bone mineral bone density in non-selected Spanish population. *Rev Osteoporos Metab Miner*. 2019;11(1):6-11.

172. González-Molero I, Rojo-Martínez G, Morcillo S, Gutiérrez-Repiso C, Rubio-Martín E, Almaraz MC, et al. Vitamin D and incidence of diabetes: A prospective cohort study. *Clin Nutr*. 2012;31(4):571-3.

173. González-Molero I, Morcillo S, Valdés S, Pérez-Valero V, Botas P, Delgado E, et al. Vitamin D deficiency in Spain: A population-based cohort study. *Eur J Clin Nutr*. 2011;65(3):321-8.

174. Goodwill AM, Campbell S, Simpson S, Jr., Bisignano M, Chiang C, Dennerstein L, et al. Vitamin D status is associated with executive function a decade later: Data from the Women’s Healthy Ageing Project. *Maturitas*. 2018;107:56-62.

175. Goswami B, Bhattacharjya H, Sengupta S, Bhattacharjee B. Hypovitaminosis D, dyslipidemia, and thyroid dysfunction among adolescents and their associations with blood pressure in a Northeastern City of India. *Indian J Community Med*. 2021;46(3):484-8.

176. Grant CC, Wall CR, Crengle S, Scragg R. Vitamin D deficiency in early childhood: Prevalent in the sunny South Pacific. *Public Health Nutr*. 2009;12(10):1893-901.

177. Greene-Finestone LS, Berger C, de Groh M, Hanley DA, Hidiroglou N, Sarafin K, et al. 25-Hydroxyvitamin D in Canadian adults: Biological, environmental, and behavioral correlates. *Osteoporos Int*. 2011;22(5):1389-99.

178. Grineva EN, Karonova T, Micheeva E, Belyaeva O, Nikitina IL. Vitamin D deficiency is a risk factor for obesity and diabetes type 2 in women at late reproductive age. *Aging (Albany NY)*. 2013;5(7):575-81.

179. Guan C, Fu S, Zhen D, Li X, Niu J, Cheng J, et al. Correlation of serum vitamin D with lipid profiles in middle-aged and elderly Chinese individuals. *Asia Pac J Clin Nutr*. 2020;29(4):839-45.

180. Gudmundsdottir SL, Hrafnkelsson H, Sigurdsson EL, Johannsson E. Serum 25-hydroxyvitamin D concentrations in 16-year-old Icelandic adolescent and its association with bone mineral density. *Public Health Nutr*. 2020;23(8):1329-33.

181. Ha C-D, Cho J-K, Lee S-H, Kang H-S. Serum vitamin D, physical activity, and metabolic risk factors in Korean children. *Med Sci Sports Exerc*. 2013;45(1):102-8.

182. Habibesadat S, Ali K, Shabnam JM, Arash A. Prevalence of vitamin D deficiency and its related factors in children and adolescents living in North Khorasan, Iran. *J Pediatr Endocrinol Metab*. 2014;27(5-6):431-6.

183. Hacker-Thompson A, Schloetter M, Sellmeyer DE. Validation of a dietary vitamin D questionnaire using multiple diet records and the block 98 health habits and history questionnaire in healthy postmenopausal women in Northern California. *J Acad Nutr Diet*. 2012;112(3):419-23.

184. Hanks LJ, Casazza K, Ashraf A, Fernandez JR. Calcium homeostasis may influence resting energy expenditure with effects most apparent in early pubertal girls. *Acta Paediatrica*. 2012;101(8):e363-e8.

185. Hansen JG, Gao W, Dupuis J, O'Connor GT, Tang W, Kowgier M, et al. Association of 25-Hydroxyvitamin D status and genetic variation in the vitamin D metabolic pathway with FEV1 in the Framingham Heart Study. *Respir Res*. 2015;16(1):81.

186. Hao Y, Ma X, Luo Y, Ni J, Dou J, Zhu J, et al. Additional role of serum 25-hydroxyvitamin D₃ levels in atherosclerosis in Chinese middle-aged and elderly men. *Clinical and Experimental Pharmacology and Physiology*. 2014;41(3):174-9.

187. Hasan HA, AbuOdeh ReO, Muda WAMBW, Mohamed HJBJ, Samsudin AR. Association of vitamin D receptor gene polymorphisms with metabolic syndrome and its components among adult Arabs from the United Arab Emirates. *Diabetes & Metabolic Syndrome*. 2017;11 Suppl 2:S531-S7.

188. Haslam A, Johnson MA, Hausman DB, Cress ME, Houston DK, Davey A, et al. Vitamin D status is associated with grip strength in centenarians. *J Nutr Gerontol Geriatr*. 2014;33(1):35-46.

189. Hata R, Miyamoto K, Abe Y, Sasaki T, Oguma Y, Tajima T, et al. Osteoporosis and sarcopenia are associated with each other and reduced IGF1 levels are a risk for both diseases in the very old elderly. *Bone*. 2023;166:116570.

190. Hauksson HH, Hrafnkelsson H, Magnusson KT, Johannsson E, Sigurdsson EL. Vitamin D status of Icelandic children and its influence on bone accrual. *J Bone Miner Metab*. 2016;34(5):580-6.

191. Hekimoğlu B, Erin R, Yılmaz HK. Comparison of cord blood and 6-month-old vitamin D levels of healthy term infants supplemented with 400 IU/day dose of vitamin D. *Eur J Clin Nutr*. 2023;77(2):182-8.

192. Hekimsoy Z, Dinç G, Kafesçiler S, Onur E, Güvenç Y, Pala T, et al. Vitamin D status among adults in the Aegean region of Turkey. *BMC Public Health*. 2010;10:782.

193. Henning T, Kochlik B, Ara I, González-Gross M, Fiorillo E, Marongiu M, et al. Patterns of dietary blood markers are related to frailty status in the FRAILOMIC validation phase. *Nutrients*. 2023;15(5):1142.

194. Herrador Z, Sordo L, Gadisa E, Buño A, Gómez-Rioja R, Iturzaeta JM, et al. Micronutrient deficiencies and related factors in school-aged children in Ethiopia: A cross-sectional study in Libo Kemkem and Fogera districts, Amhara Regional State. *PLoS One*. 2014;9(12):e112858.

195. Hien VTT, Lam NT, Skeaff CM, Todd J, McLean JM, Green TJ. Vitamin D status of pregnant and non-pregnant women of reproductive age living in Hanoi City and the Hai Duong province of Vietnam. *Matern Child Nutr*. 2012;8(4):533-9.

196. Hill TR, Granic A, Davies K, Collerton J, Martin-Ruiz C, Siervo M, et al. Serum 25-hydroxyvitamin D concentration and its determinants in the very old: The Newcastle 85+ Study. *Osteoporos Int*. 2016;27(3):1199-208.

197. Hirani V, Cumming RG, Blyth FM, Naganathan V, Le Couteur DG, Handelsman DJ, et al. Vitamin D status among older community dwelling men living in a sunny country and associations with lifestyle factors: The concord health and ageing in men project, Sydney, Australia. *J Nutr Health Aging*. 2013;17(7):587-93.

198. Hirani V. Vitamin D status and pain: Analysis from the Health Survey for England among English adults aged 65 years and over. *Br J Nutr*. 2012;107(7):1080-4.

199. Hirschler V, Maccallini G, Molinari C, Inés U, Castano LA, Group SAdlCS, et al. Association between vitamin D and Apo B concentrations in Argentinean Indian children. *Clin Chim Acta*. 2014;429:147-51.

200. Hirschler V, Molinari C, Maccallini G, Intersimone P, Gonzalez CD. Vitamin D levels and cardiometabolic markers in indigenous Argentinean children living at different altitudes. *Glob Pediatr Health*. 2019;6:2333794X18821942.

201. Hoevenaar-Blom MP, Wielders JP, Groeneveld H, de Leeuw E, Schmits RJ, Pepermans C, et al. Prevalence and determinants of vitamin D deficiency in infants and toddlers in the Netherlands: A pilot study. *Ann Clin Biochem*. 2019;56(5):613-8.

202. Hoge A, Donneau A-F, Streel S, Kolh P, Chapelle J-P, Albert A, et al. Vitamin D deficiency is common among adults in Wallonia (Belgium, 51°30' North): Findings from the Nutrition, Environment and Cardio-Vascular Health study. *Nutr Res*. 2015;35(8):716-25.

203. Horton-French K, Dunlop E, Lucas RM, Pereira G, Black LJ. Prevalence and predictors of vitamin D deficiency in a nationally representative sample of Australian adolescents and young adults. *Eur J Clin Nutr*. 2021;75(11):1627-36.

204. Houghton LA, Brown RC, Beaumont S, Jennings S, Bailey KB, Haszard JJ, et al. Micronutrient status differs among Maasai and Kamba preschoolers in a supplementary feeding programme in Kenya. *Matern Child Nutr*. 2019;15(3):e12805.

205. Houston DK, Tooze JA, Davis CC, Chaves PHM, Hirsch CH, Robbins JA, et al. Serum 25-hydroxyvitamin D and physical function in older adults: The Cardiovascular Health Study All Stars. *J Am Geriatr Soc*. 2011;59(10):1793-801.

206. Hribar M, Hristov H, Gregorič M, Blaznik U, Zaletel K, Oblak A, et al. Nutrihealth study: Seasonal variation in vitamin D status among the Slovenian adult and elderly population. *Nutrients*. 2020;12(6):1838.

207. Hu Y, Li S, Wang J, Zheng D, Zhang H, Yu W, et al. Threshold for relationship between vitamin D and parathyroid hormone in Chinese women of childbearing age. *Int J Environ Res Public Health*. 2021;18(24):13060.

208. Hu Z, Zhi X, Ma Y, Li J, Wang J, Zhu J, et al. The modification of individual factors on association between serum 25(OH)D and incident type 2 diabetes: Results from a prospective cohort study. *Frontiers in Nutrition*. 2022;9:1077734.

209. Hu Y, Jiang S, Lu J, Yang Z, Yang X, Yang L. Vitamin D status for chinese children and adolescents in CNNHS 2016-2017. *Nutrients*. 2022;14(22):4928.

210. Huang Y, Eapen E, Steele S, Grey V. Establishment of reference intervals for bone markers in children and adolescents. *Clin Biochem*. 2011;44(10-11):771-8.

211. Huang Y, Li X, Wang M, Ning H, A L, Li Y, et al. Lipoprotein lipase links vitamin D, insulin resistance, and type 2 diabetes: A cross-sectional epidemiological study. *Cardiovascular Diabetology*. 2013;12:17.

212. Huang T, Afzal S, Yu C, Guo Y, Bian Z, Yang L, et al. Vitamin D and cause-specific vascular disease and mortality: A Mendelian randomisation study involving 99,012 Chinese and 106,911 European adults. *BMC Med*. 2019;17(1):160.

213. Huang P, Ke G, Lin X, Wang Q, Lu W, Zeng L, et al. Correlation analysis between vitamin A, D, and E status with altitude, seasonal variation, and other factors, among children aged 0-6 years in a Chinese population living in the Tibetan plateau of Ganzi prefecture. *J Clin Lab Anal*. 2022;36(9):e24620.

214. Huang YY, Zhang WS, Jiang CQ, Zhu F, Jin YL, Cheng KK, et al. Mendelian randomization on the association of obesity with vitamin D: Guangzhou Biobank Cohort Study. *Eur J Clin Nutr*. 2023;77(2):195-201.

215. Hurskainen A-R, Virtanen JK, Tuomainen T-P, Nurmi T, Voutilainen S. Association of serum 25-hydroxyvitamin D with type 2 diabetes and markers of insulin resistance in a general older population in Finland. *Diabetes Metab Res Rev*. 2012;28(5):418-23.

216. Husemoen LL, Ebstrup JF, Mortensen EL, Schwarz P, Skaaby T, Thuesen BH, et al. Serum 25-hydroxyvitamin D and self-reported mental health status in adult Danes. *Eur J Clin Nutr*. 2016;70(1):78-84.

217. Hussain N, Said ASA, Baig MR. Levels of vitamin D and vitamin D pathway gene polymorphisms in adults: Results based on rural agriculture workers in Punjab province of Pakistan. *Progr Nutr*. 2020;22(2):485-92.

218. Huta-Osiecka A, Kasprzak Z, Wochna K, Nowak A. Serum 25-hydroxyvitamin D concentrations and selected diet components in postmenopausal women. *Acta Sci Pol Technol Aliment*. 2017;16(4):443-9.

219. Hutchings N, Babalyan V, Heijboer AC, Baghdasaryan S, Qefoyan M, Ivanyan A, et al. Vitamin D status in Armenian women: A stratified cross-sectional cluster analysis. *Eur J Clin Nutr*. 2022;76(2):220-6.

220. Ikonen H, Lumme J, Seppälä J, Pesonen P, Piltonen T, Järvelin M-R, et al. The determinants and longitudinal changes in vitamin D status in middle-age: A Northern Finland Birth Cohort 1966 study. *Eur J Nutr*. 2021;60(8):4541-53.

221. Imran Y, Aswar A, Rachmiyani I, Adriani D. Low 25-hydroxyvitamin D level increases carotid intima-media thickness in elderly women. *Universa Medicina*. 2019;38(2):114-20.

222. Jääskeläinen T, Itkonen ST, Lundqvist A, Erkkola M, Koskela T, Lakkala K, et al. The positive impact of general vitamin D food fortification policy on vitamin D status in a representative adult Finnish population: Evidence from an 11-y follow-up based on standardized 25-hydroxyvitamin D data. *Am J Clin Nutr*. 2017;105(6):1512-20.

223. Jain V, Gupta N, Kalaivani M, Jain A, Sinha A, Agarwal R. Vitamin D deficiency in healthy breastfed term infants at 3 months & their mothers in India: Seasonal variation & determinants. *Indian J Med Res*. 2011;133(3):267-73.

224. Jakab E, Kalina E, Petho Z, Pap Z, Balogh A, Grant WB, et al. Standardizing 25-hydroxyvitamin D data from the HunMen cohort. *Osteoporos Int*. 2017;28(5):1653-7.

225. Jamali Z, Asadikaram G, Mahmoodi M, Sayadi A, Jamalizadeh A, Saleh-Moghadam M, et al. Vitamin D status in female students and its relation to calcium metabolism markers, lifestyles, and polymorphism in vitamin D receptor. *Clin Lab*. 2013;59(3-4):407-13.

226. Jamka K, Adamczuk P, Skowrońska A, Bojar I, Raszewski G. Assessment of the effect of estradiol on biochemical bone turnover markers among postmenopausal women. *Annals of Agricultural and Environmental Medicine*. 2021;28(2):326-30.

227. Jang HB, Lee H-J, Park JY, Kang J-H, Song J. Association between serum vitamin D and metabolic risk factors in Korean schoolgirls. *Osong Public Health Res Perspect*. 2013;4(4):179-86.

228. Janmohamed A, Luvsanjamba M, Norov B, Batsaikhan E, Jamiyan B, Blankenship JL. Complementary feeding practices and associated factors among Mongolian children 6–23 months of age. *Matern Child Nutr*. 2020;16(S2):e12838.

229. Janssen HCJP, Emmelot-Vonk MH, Verhaar HJJ, van der Schouw YT. Vitamin D and muscle function: Is there a threshold in the relation? *Journal of the American Medical Directors Association*. 2013;14(8):627.e13-.e18.

230. Jarosz AC, Noori D, Zeitoun T, Garcia-Bailo B, El-Sohemy A. Variation in the vitamin D receptor gene, plasma 25-hydroxyvitamin D, and risk of premenstrual symptoms. *Genes Nutr*. 2021;16:15.

231. Jayatissa R, Lekamwasam S, Ranbanda JM, Ranasingha S, Perera AG, De Silva KH. Vitamin D deficiency among children aged 10-18 years in Sri Lanka. *Ceylon Med J*. 2019;64(4):146-54.

232. Jeddi M, Roosta MJ, Dabbaghmanesh MH, Omrani GR, Ayatollahi SMT, Bagheri Z, et al. Normative data and percentile curves of bone mineral density in healthy Iranian children aged 9-18 years. *Arch Osteoporos*. 2013;8:114.

233. Jeenduang N, Plyduang T, Horpet D. Association of 25-hydroxyvitamin D levels and metabolic syndrome in Thai postmenopausal women. *Diabetes Metab Syndr*. 2020;14(6):1585-90.

234. Jeenduang N, Sriprachan C, Plyduang T, Nuinoon M, Horpet D, Sangkaew B, et al. Vitamin D status and its associated factors in rural subjects in Nakhon Si Thammarat Province, Southern Thailand. *J Med Assoc Thai*. 2018;101(3):397-404.

235. Jelmila SN, Sulastri D, Lestari Y. Correlation between 25-Hydroxyvitamin D serum levels with telomere length in premenopausal Minangkabau ethnicity women. *Indian J Public Health Res Dev*. 2020;11(3):1150-60.

236. Jeon D-H, Yeom H, Yang J, Song JS, Lee HK, Kim HC. Are serum vitamin D levels associated with dry eye disease? Results from the study group for environmental eye disease. *J Prev Med Public Health*. 2017;50(6):369-76.

237. Jiajue R, Liu S, Pei Y, Qi X, Jiang Y, Wang Q, et al. Associations between osteocalcin, calciotropic hormones, and energy metabolism in a cohort of Chinese postmenopausal women: Peking vertebral fracture study. *Int J Endocrinol*. 2021;2021:5585018.

238. Jiang W, Wu D-B, Xiao G-B, Ding B, Chen E-Q. An epidemiology survey of vitamin D deficiency and its influencing factors. *Med Clin (Barc)*. 2020;154(1):7-12.

239. Johansson H, Odén A, Kanis J, McCloskey E, Lorentzon M, Ljunggren Ö, et al. Low serum vitamin D is associated with increased mortality in elderly men: MrOS Sweden. *Osteoporos Int*. 2012;23(3):991-9.

240. Jones P, Lucock M, Martin C, Thota R, Garg M, Yates Z, et al. Independent and interactive influences of environmental UVR, vitamin D levels, and folate variant MTHFD1-rs2236225 on homocysteine levels. *Nutrients*. 2020;12(5):1455.

241. Joukar F, Naghipour M, Hassanipour S, Salari A, Alizadeh A, Saeidi-Saedi H, et al. Association of serum levels of vitamin D with blood pressure status in Northern Iranian population: The PERSIAN Guilan Cohort Study (PGCS). *Int J Gen Med*. 2020;13:99-104.

242. Juwita F, Gumilang L, Risan NA, Dhamayanti M. The association of vitamin D and neurodevelopmental status among 2 years old infants. *Glob Pediatr Health*. 2021;8:2333794X211034075.

243. Kaddam IM, Al-Shaikh AM, Abaalkhail BA, Asseri KS, Al-Saleh YM, Al-Qarni AA, et al. Prevalence of vitamin D deficiency and its associated factors in three regions of Saudi Arabia. *Saudi Med J*. 2017;38(4):381-90.

244. Kandhro F, Dahot U, Naqvi SHA, Ujjan IU. Study of vitamin D deficiency and contributing factors in the population of Hyderabad, Pakistan. *Pakistan Journal of Pharmaceutical Sciences*. 2019;32(3):1063-8.

245. Kaneva AM, Potolitsyna NN, Bojko ER. Association of serum 25-hydroxyvitamin D with metabolic disturbances in adolescents. *Am J Hum Biol*. 2022;34(12):e23802.

246. Kapil U, Pandey RM, Goswami R, Sharma B, Sharma N, Ramakrishnan L, et al. Prevalence of vitamin D deficiency and associated risk factors among children residing at high altitude in Shimla district, Himachal Pradesh, India. *Indian J Endocrinol Metab*. 2017;21(1):178-83.

247. Kapil U, Pandey RM, Sharma B, Ramakrishnan L, Sharma N, Singh G, et al. Prevalence of vitamin D deficiency in children (6–18 years) residing in Kullu and Kangra districts of Himachal Pradesh, India. *Indian Journal of Pediatrics*. 2018;85(5):344-50.

248. Karagüzel G, Dilber B, Çan G, Ökten A, Değer O, Holick MF. Seasonal vitamin D status of healthy schoolchildren and predictors of low vitamin D status. *J Pediatr Gastroenterol Nutr*. 2014;58(5):654-60.

249. Karhapää P, Pihlajamäki J, Pörsti I, Kastarinen M, Mustonen J, Niemelä O, et al. Glomerular filtration rate and parathyroid hormone are associated with 1,25-dihydroxyvitamin D in men without chronic kidney disease. *J Intern Med*. 2012;271(6):573-80.

250. Karimi F, Dabbaghmanesh MH, Omrani GR. Association between serum uric acid and bone health in adolescents. *Osteoporos Int*. 2019;30(10):2057-64.

251. Karin Z, Gilic B, Supe Domic D, Sarac Z, Ercegovic K, Zenic N, et al. Vitamin D status and analysis of specific correlates in preschool children: A cross-sectional study in southern Croatia. *Int J Environ Res Public Health*. 2018;15(11):2503.

252. Karuppusami R, Antonisami B, Vasan SK, Gowri M, Selliah HY, Arulappan G, et al. Association of serum 25-Hydroxy vitamin D with total and regional adiposity and cardiometabolic traits. *PLoS One*. 2020;15(12):e0243850.

253. Kassem E, Eilat-Adar S, Sindiani M, Ben-Zaken S. Sex differences in vitamin D deficiency and anthropometric measurements in school-age children from rural areas in Israel. *Isr Med Assoc J*. 2020;11(22):696-9.

254. Kaykhaei MA, Hashemi M, Narouie B, Shikhzadeh A, Rashidi H, Moulaei N, et al. High prevalence of vitamin D deficiency in Zahedan, southeast Iran. *Ann Nutr Metab*. 2011;58(1):37-41.

255. Ke L, Mason RS, Mpofu E, Dibley M, Li Y, Brock KE. Vitamin D and parathyroid hormone status in a representative population living in Macau, China. *J Steroid Biochem Mol Biol*. 2015;148:261-8.

256. Ke L, Mason RS, Baur LA, Cowell CT, Liu X, Garnett SP, et al. Vitamin D levels in childhood and adolescence and cardiovascular risk factors in a cohort of healthy Australian children. *J Steroid Biochem Mol Biol*. 2018;177:270-7.

257. Kensara OA, Helal OF, El-Kafy EMA, Ghafouri KJ, Ghaith MM, Alsolami FJ, et al. The combined effect of vitamin D deficiency and hyperparathyroidism on postural stability among healthy adult males. *Pakistan Journal of Biological Sciences*. 2019;22(9):406-11.

258. Kensarah OA, Jazar AS, Azzeh FS. Hypovitaminosis D in healthy toddlers and preschool children from Western Saudi Arabia. *Int J Vitam Nutr Res*. 2015;85(1-2):50-60.

259. Khader YS, Batieha A, Jaddou H, Batieha Z, El-Khateeb M, Ajlouni K. Relationship between 25-hydroxyvitamin D and metabolic syndrome among Jordanian adults. *Nutrition Research and Practice*. 2011;5(2):132-9.

260. Khadilkar A, Kajale N, Oza C, Oke R, Gondhalekar K, Patwardhan V, et al. Vitamin D status and determinants in Indian children and adolescents: A multicentre study. *Sci Rep*. 2022;12(1):16790.

261. Khan AH, Fatima SS, Raheem A, Jafri L. Are serum leptin levels predicted by lipoproteins, vitamin D and body composition? *World J Diabetes*. 2019;10(4):260-8.

262. Khan AH, Iqbal R, Naureen G, Dar FJ, Ahmed FN. Prevalence of vitamin D deficiency and its correlates: Results of a community-based study conducted in Karachi, Pakistan. *Arch Osteoporos*. 2012;7:275-82.

263. Khan S, Pokharel B, Bhagat T, Joshi B, Sapkota S, Mishra B, et al. Prevalence of vitamin D insufficiency in school going children of eastern nepal: A cross-sectional study. *Int Med*. 2020;2(4):214.

264. Khayyatzadeh SS, Vatanparast H, Avan A, Bagherniya M, Bahrami A, Kiani MA, et al. Serum transaminase concentrations and the presence of irritable bowel syndrome are associated with serum 25-hydroxy vitamin D concentrations in adolescent girls who are overweight and obese. *Ann Nutr Metab*. 2017;71(3-4):234-41.

265. Khor GL, Chee WSS, Shariff ZM, Poh BK, Arumugam M, Rahman JA, et al. High prevalence of vitamin D insufficiency and its association with BMI-for-age among primary school children in Kuala Lumpur, Malaysia. *BMC Public Health*. 2011;11:95.

266. Khosravi-Boroujeni H, Sarrafzadegan N, Sadeghi M, Roohafza H, Ng S-K, Pourmogaddas A, et al. Prevalence and trends of vitamin D deficiency among Iranian adults: A longitudinal study from 2001-2013. *J Nutr Sci Vitaminol (Tokyo)*. 2017;63(5):284-90.

267. Khwanchuea R, Punsawad C. Associations between body composition, leptin, and vitamin D varied by the body fat percentage in adolescents. *Front Endocrinol (Lausanne)*. 2022;13:876231.

268. Kim G, Oh KW, Jang E-H, Kim M-K, Lim D-J, Kwon HS, et al. Relationship between vitamin D, parathyroid hormone, and bone mineral density in elderly Koreans. *J Korean Med Sci*. 2012;27(6):636-43.

269. Kim J, Lee Y, Won CW, Kye S, Shim J-S. Association of serum vitamin D with frailty in older Korean adults. *Maturitas*. 2020;134:15-20.

270. Kim TN, Park MS, Lim KI, Choi HY, Yang SJ, Yoo HJ, et al. Relationships between sarcopenic obesity and insulin resistance, inflammation, and vitamin D status: The Korean Sarcopenic Obesity Study. *Clinical Endocrinology*. 2013;78(4):525-32.

271. Kim Y, Chang Y, Ryu S, Cho IY, Kwon M-J, Sohn W, et al. Resolution of, and risk of incident non-alcoholic fatty liver disease with changes in serum 25-hydroxy vitamin D status. *J Clin Endocrinol Metab*. 2022;107(8):e3437-e47.

272. Kimlin MG, Lucas RM, Harrison SL, van Der Mei I, Armstrong BK, Whiteman DC, et al. The contributions of solar ultraviolet radiation exposure and other determinants to serum 25-hydroxyvitamin D concentrations in Australian adults: the AusD Study. *Am J Epidemiol*. 2014;179(7):864-74.

273. Kılınç S, Atay E, Ceran Ö, Atay Z. Evaluation of vitamin D status and its correlation with gonadal function in children at mini-puberty. *Clinical Endocrinology*. 2019;90(1):122-8.

274. Klenk J, Rapp K, Denkinger MD, Nagel G, Nikolaus T, Peter R, et al. Seasonality of vitamin D status in older people in Southern Germany: Implications for assessment. *Age Ageing*. 2013;42(3):404-8.

275. Koda S, Wada K, Yamakawa M, Nakashima Y, Hayashi M, Takeda N, et al. Associations of plasma 25-hydroxy vitamin D and dietary vitamin D intake with insulin eesistance in healthy Japanese women. *J Nutr Sci Vitaminol (Tokyo)*. 2023;69(1):46-52.

276. Kopiczko A, Łopuszańska-Dawid M, Gryko K. Bone mineral density in young adults: The influence of vitamin D status, biochemical indicators, physical activity and body composition. *Arch Osteoporos*. 2020;15(1):45.

277. Kouda K, Nakamura H, Fujita Y, Ohara K, Iki M. Vitamin D status and body fat measured by dual-energy X-ray absorptiometry in a general population of Japanese children. *Nutrition*. 2013;29(10):1204-8.

278. Kruavit A, Chailurkit L-o, Thakkinstian A, Sriphrapradang C, Rajatanavin R. Prevalence of vitamin D insufficiency and low bone mineral density in elderly Thai nursing home residents. *BMC Geriatr*. 2012;12:49.

279. Kull M, Kallikorm R, Lember M. Vitamin D as a possible independent predictor of bone mineral density in Estonian adults: A cross-sectional population-based study. *Internal Medicine Journal*. 2012;42(6):e89-e94.

280. Kuraoka S, Oda M, Mitsubuchi H, Nakamura K, Katoh T, Japan Environment And Children's Study Jecs Group. Impaired height growth associated with vitamin D deficiency in young children from the Japan environment and children's study. *Nutrients*. 2022;14(16):3325.

281. Kutlay S, Atli T, Aydogan I, Tutkak H, Nergizoglu G. The association of serum vitamin D levels with several cardiometabolic risk and aortic pulse wave velocity in elderly persons. *Eur Geriatr Med*. 2014;5(4):238-41.

282. Kuwabara A, Tsugawa N, Ao M, Ohta J, Tanaka K. Vitamin D deficiency as the risk of respiratory tract infections in the institutionalized elderly: A prospective 1-year cohort study. *Clin Nutr ESPEN*. 2020;40:309-13.

283. Laaksi A, Laaksi I, Pihlajamäki H, Vaara JP, Luukkaala T, Kyröläinen H. Associations of serum 25(OH)D levels with physical performance and anabolic hormones in young men. *Frontiers in Physiology*. 2023;14:1049503.

284. Laillou A, Wieringa F, Tran TN, Van PT, Le BM, Fortin S, et al. Hypovitaminosis D and mild hypocalcaemia are highly prevalent among young Vietnamese children and women and related to low dietary intake. *PLoS One*. 2013;8(5):e63979.

285. Laird E, O’Halloran AM, Carey D, Healy M, O’Connor D, Moore P, et al. The prevalence of vitamin D deficiency and the determinants of 25 (OH) D concentration in older Irish adults: Data from The Irish Longitudinal Study on Ageing (TILDA). *J Gerontol A Biol Sci Med Sci*. 2018;73(4):519-25.

286. Lam V, Albrecht MA, Takechi R, Prasopsang P, Lee YP, Foster JK, et al. Serum 25-hydroxyvitamin D is associated with reduced verbal episodic memory in healthy, middle-aged and older adults. *Eur J Nutr*. 2016;55(4):1503-13.

287. Landgrebe AV, Lund MAV, Lausten-Thomsen U, Frithioff-Bøjsøe C, Fonvig CE, Plesner JL, et al. Population-based pediatric reference values for serum parathyroid hormone, vitamin D, calcium, and phosphate in Danish/North-European white children and adolescents. *Clin Chim Acta*. 2021;523:483-90.

288. Larcombe L, Mookherjee N, Slater J, Slivinski C, Singer M, Whaley C, et al. Vitamin D in a Northern Canadian first nation population: Dietary intake, serum concentrations and functional gene polymorphisms. *PLoS One*. 2012;7(11):e49872.

289. Larijani B, Hossein-Nezhad A, Feizabad E, Maghbooli Z, Adibi H, Ramezani M, et al. Vitamin D deficiency, bone turnover markers and causative factors among adolescents: A cross-sectional study. *Journal of Diabetes & Metabolic Disorders*. 2016;15:46.

290. Larsen AU, Hopstock LA, Jorde R, Grimnes G. Associations of serum 25-hydroxyvitamin D and subjective sleep measures in an arctic population: Insights from the population-based Tromsø Study. *Sleep Med X*. 2022;4:100056.

291. Lategan R, Van den Berg VL, Ilich JZ, Walsh CM. Vitamin D status, hypertension and body mass index in an urban black community in Mangaung, South Africa. *Afr J Prim Health Care Fam Med*. 2016;8(1):1210.

292. Lee DM, Tajar A, O'Neill TW, O'Connor DB, Bartfai G, Boonen S, et al. Lower vitamin D levels are associated with depression among community-dwelling European men. *J Psychopharmacol*. 2011;25(10):1320-8.

293. Lee EY, Lee SJ, Kim KM, Yun YM, Song BM, Kim JE, et al. Association of metabolic syndrome and 25-hydroxyvitamin D with cognitive impairment among elderly Koreans. *Geriatr Gerontol Int*. 2017;17(7):1069-75.

294. Lee I, Park E, Cho J. Association of nonalcoholic fatty liver disease with serum vitamin D levels in combination of physical fitness in Korean older adults. *J Steroid Biochem Mol Biol*. 2020;198:105569.

295. Lee J, Won Woo H, Kim J, Shin M-H, Koh I, Youl Choi B, et al. Independent and interactive associations of season, dietary vitamin D, and vitamin D-related genetic variants with serum 25(OH)D in Korean adults aged 40 years or older. *Endocr J*. 2021;68(6):701-11.

296. Leirós M, Amenedo E, Rodríguez M, Pazo-Álvarez P, Franco L, Leis R, et al. Cognitive status and nutritional markers in a sample of institutionalized elderly people. *Front Aging Neurosci*. 2022;14:880405.

297. Leitão J, Carvalhana S, Silva AP, Velasco F, Medeiros I, Alves AC, et al. No evidence for lower levels of serum vitamin D in the presence of hepatic steatosis. A study on the portuguese general population. *Int J Med Sci*. 2018;15(14):1778-86.

298. Lemming EW, Sipinen JP, Nyberg G, Moraeus L, Lindroos AK. Vitamin D status and associations with diet, objectively measured physical activity patterns and background characteristics among adolescents in a representative national cross-sectional survey. *Public Health Nutr*. 2022;25(6):1427-37.

299. Leung RY, Cheung BM, Nguyen U-S, Kung AW, Tan KC, Cheung C-L. Optimal vitamin D status and its relationship with bone and mineral metabolism in Hong Kong Chinese. *Bone*. 2017;97:293-8.

300. Li X, Qu C, Wang Y, Mao Z, Wang C, Li W, et al. Associations of CYP24A1 copy number variation with vitamin D deficiency and insulin secretion. *Appl Physiol Nutr Metab*. 2019;44(12):1367-70.

301. Li C, Chen P, Duan X, Wang J, Shu B, Li X, et al. Bioavailable 25(OH)D but not total 25(OH)D is an independent determinant for bone mineral density in Chinese postmenopausal women. *EBioMedicine*. 2017;15:184-92.

302. Li M, Lv F, Zhang Z, Deng W, Li Y, Deng Z, et al. Establishment of a normal reference value of parathyroid hormone in a large healthy Chinese population and evaluation of its relation to bone turnover and bone mineral density. *Osteoporos Int*. 2016;27(5):1907-16.

303. Li L, Li K, Li J, Luo Y, Cheng Y, Jian M, et al. Ethnic, geographic, and seasonal differences of vitamin D status among adults in South-west China. *J Clin Lab Anal*. 2020;34(12):e23532.

304. Li D, Wei H, Xue H, Zhang J, Chen M, Gong Y, et al. Higher serum 25(OH)D level is associated with decreased risk of impairment of glucose homeostasis: Data from Southwest China. *BMC Endocr Disord*. 2018;18(1):25.

305. Li J, Ding W, Cao J, Sun L, Liu S, Zhang J, et al. Serum 25-hydroxyvitamin D and bone mineral density among children and adolescents in a Northwest Chinese city. *Bone*. 2018;116:28-34.

306. Li C-W, Yu K, Shyh-Chang N, Li G-X, Yu S-L, Liu H-J, et al. Sterol metabolism and protein metabolism are differentially correlated with sarcopenia in Asian Chinese men and women. *Cell Prolif*. 2021;54(4):e12989.

307. Li H, Huang T, Xiao P, Zhao X, Liu J, Cheng H, et al. Widespread vitamin D deficiency and its sex-specific association with adiposity in Chinese children and adolescents. *Nutrition*. 2020;71:110646.

308. Liaqat S, Ahmad J, Safdar R. Prevalence of vitamin D deficiency among infants and toddlers: Cross sectional study. *Indo Am J P Sci*. 2019;6(6):12199-204.

309. Lim S, Shin H, Kim MJ, Ahn HY, Kang SM, Yoon JW, et al. Vitamin D inadequacy is associated with significant coronary artery stenosis in a community-based elderly cohort: The Korean Longitudinal Study on Health and Aging. *J Clin Endocrinol Metab*. 2012;97(1):169-78.

310. Lima-Costa MF, Mambrini JVM, de Souza-Junior PRB, de Andrade FB, Peixoto SV, Vidigal CM, et al. Nationwide vitamin D status in older Brazilian adults and its determinants: The Brazilian Longitudinal Study of Aging (ELSI). *Sci Rep*. 2020;10(1):13521.

311. Lin L-Y, Smeeth L, Langan S, Warren-Gash C. Distribution of vitamin D status in the UK: A cross-sectional analysis of UK Biobank. *BMJ Open*. 2021;11(1):e038503.

312. Lingham G, Yazar S, Lucas RM, Walsh JP, Zhu K, Hunter M, et al. Low 25-hydroxyvitamin D concentration is not associated with refractive error in middle-aged and older Western Australian adults. *Transl Vis Sci Technol*. 2019;8(1):13.

313. Lingham G, Mackey DA, Zhu K, Lucas RM, Black LJ, Oddy WH, et al. Time spent outdoors through childhood and adolescence – assessed by 25-hydroxyvitamin D concentration – and risk of myopia at 20 years. *Acta Ophthalmologica*. 2021;99(6):679-87.

314. Liu J, Ma W, Wei L, Yang Y, Yang R, Shao F, et al. Adult serum 25(OH)D₃ in Gansu province, Northwest China: A cross-sectional study. *Asia Pac J Clin Nutr*. 2018;27(4):832-9.

315. Liu Y, Li X, Zhao A, Zheng W, Guo M, Xue Y, et al. High prevalence of insufficient vitamin D intake and serum 25-hydroxyvitamin D in Chinese school-age children: A cross-sectional study. *Nutrients*. 2018;10(7):822.

316. Liu L, Cao Z, Lu F, Liu Y, Lv Y, Qu Y, et al. Vitamin D deficiency and metabolic syndrome in elderly Chinese individuals: Evidence from CLHLS. *Nutr Metab (Lond)*. 2020;17:58.

317. Liu X, Brock KE, Brennan-Speranza TC, Flicker L, Golledge J, Hankey GJ, et al. Healthy lifestyles are associated with better vitamin D status in community-dwelling older men: The Health In Men Study (HIMS). *Clinical Endocrinology*. 2023;99(2):165-73.

318. Liu Y, Xia Y, Wu Q, Chang Q, Zhao Y. Associations between serum vitamins and serum uric acid in a population of Shenyang, China. *J Nutr Sci Vitaminol (Tokyo)*. 2021;67(2):77-83.

319. Lopes JB, Fernandes GH, Takayama L, Figueiredo CP, Pereira RMR. A predictive model of vitamin D insufficiency in older community people: From the São Paulo Aging & Health Study (SPAH). *Maturitas*. 2014;78(4):335-40.

320. Lourenço BH, Qi L, Willett WC, Cardoso MA. FTO genotype, vitamin D status, and weight gain during childhood. *Diabetes*. 2014;63(2):808-14.

321. Lu L, Sheng H, Li H, Gan W, Liu C, Zhu J, et al. Associations between common variants in GC and DHCR7/NADSYN1 and vitamin D concentration in Chinese Hans. *Hum Genet*. 2012;131(3):505-12.

322. Lu Y, Zheng Y, Wang N, Chen Y, Li Q, Han B, et al. The relationship between vitamin D and type 2 diabetes is intriguing: Glimpses from the Spect-China study. *Ann Nutr Metab*. 2017;71(3-4):195-202.

323. Luo S, Chen X, Hou L, Yue J, Liu X, Wang Y, et al. The relationship between sarcopenia and vitamin D levels in adults of different ethnicities: Findings from the West China Health and Aging Trend study. *J Nutr Health Aging*. 2021;25(7):909-13.

324. Lutsey PL, Parrinello CM, Misialek JR, Hoofnagle AN, Henderson CM, Laha TJ, et al. Short-term variability of vitamin D–Related biomarkers. *Clin Chem*. 2016;62(12):1647-53.

325. Luxwolda MF, Kuipers RS, Kema IP, van der Veer E, Dijck-Brouwer DAJ, Muskiet FAJ. Vitamin D status indicators in indigenous populations in East Africa. *Eur J Nutr*. 2013;52(3):1115-25.

326. Lwow F, Bohdanowicz-Pawlak A. Vitamin D and selected cytokine concentrations in postmenopausal women in relation to metabolic disorders and physical activity. *Exp Gerontol*. 2020;141:111107.

327. Macdonald HM, Mavroeidi A, Aucott LA, Diffey BL, Fraser WD, Ormerod AD, et al. Skin color change in Caucasian postmenopausal women predicts summer-winter change in 25-hydroxyvitamin D: Findings from the ANSAViD cohort study. *J Clin Endocrinol Metab*. 2011;96(6):1677-86.

328. MacDonell SO, Miller JC, Harper MJ, Reid MR, Haszard JJ, Gibson RS, et al. Multiple micronutrients, including zinc, selenium and iron, are positively associated with anemia in New Zealand aged care residents. *Nutrients*. 2021;13(4).

329. MacDonell SO, Miller JC, Harper MJ, Waters DL, Houghton LA. Vitamin D status and its predictors in New Zealand aged-care residents eligible for a government-funded universal vitamin D supplementation programme. *Public Health Nutr*. 2016;19(18):3349-60.

330. Maddah M, Sharami SH, Neyestani TR. Vitamin D insufficiency among postmenopausal women in urban and rural areas in Guilan, Northern Iran. *J Nutr Elder*. 2009;28(4):386-93.

331. Madsen KH, Rasmussen LB, Mejborn H, Andersen EW, Mølgaard C, Nissen J, et al. Vitamin D status and its determinants in children and adults among families in late summer in Denmark. *Br J Nutr*. 2014;112(5):776-84.

332. Majumdar V, Nagaraja D, Christopher R. Vitamin D status and metabolic syndrome in Asian Indians. *International Journal of Obesity*. 2011;35(8):1131-4.

333. Malacova E, Cheang PR, Dunlop E, Sherriff JL, Lucas RM, Daly RM, et al. Prevalence and predictors of vitamin D deficiency in a nationally representative sample of adults participating in the 2011-2013 Australian Health Survey. *Br J Nutr*. 2019;121(8):894-904.

334. Mallah EM, Hamad MF, Elmanaseer MA, Qinna NA, Idkaidek NM, Arafat TA, et al. Plasma concentrations of 25-hydroxyvitamin D among Jordanians: Effect of biological and habitual factors on vitamin D status. *BMC Clin Pathol*. 2011;11:8.

335. Manios Y, Moschonis G, Hulshof T, Bourhis A-S, Hull GLJ, Dowling KG, et al. Prevalence of vitamin D deficiency and insufficiency among schoolchildren in Greece: The role of sex, degree of urbanisation and seasonality. *Br J Nutr*. 2017;118(7):550-8.

336. Mansuri S, Badawi A, Kayaniyil S, Cole DE, Harris SB, Mamakeesick M, et al. Traditional foods and 25(OH)D concentrations in a subarctic First Nations community. *Int J Circumpolar Health*. 2016;75(1):31956.

337. Marasinghe E, Chackrewarthy S, Abeysena C, Rajindrajith S. Micronutrient status and its relationship with nutritional status in preschool children in urban Sri Lanka. *Asia Pac J Clin Nutr*. 2015;24(1):144-51.

338. Martini LA, Verly EJ, Marchioni DML, Fisberg RM. Prevalence and correlates of calcium and vitamin D status adequacy in adolescents, adults, and elderly from the Health Survey-São Paulo. *Nutrition*. 2013;29(6):845-50.

339. Marwaha RK, Tandon N, Garg MK, Kanwar R, Narang A, Sastry A, et al. Bone health in healthy Indian population aged 50 years and above. *Osteoporos Int*. 2011;22(11):2829-36.

340. Marzban M, Kalantarhormozi M, Mahmudpour M, Ostovar A, Keshmiri S, Darabi AH, et al. Prevalence of vitamin D deficiency and its associated risk factors among rural population of the Northern part of the Persian Gulf. *BMC Endocr Disord*. 2021;21(1):219.

341. Masoud MS, Yakout SM, Al-Attas OS, Alokail MS, Al-Daghri NM. The association between iron and vitamin D status in Arab adolescents. *Public Health Nutr*. 2020;23(7):1208-13.

342. Masson S, Agabiti N, Vago T, Miceli M, Mayer F, Letizia T, et al. The fibroblast growth factor-23 and vitamin D emerge as nontraditional risk factors and may affect cardiovascular risk. *J Intern Med*. 2015;277(3):318-30.

343. Mat S, Jaafar MH, Sockalingam S, Raja J, Kamaruzzaman SB, Chin A-V, et al. Vitamin D deficiency is associated with ethnicity and knee pain in a multi-ethnic South-East Asian nation: Results from Malaysian Elders Longitudinal Research (MELoR). *Int J Rheum Dis*. 2018;21(5):930-6.

344. Mata-Granados JM, Cuenca-Acebedo R, Luque de Castro MD, Quesada Gómez JM. Lower vitamin E serum levels are associated with osteoporosis in early postmenopausal women: A cross-sectional study. *J Bone Miner Metab*. 2013;31(4):455-60.

345. Mavroeidi A, Aucott L, Black AJ, Fraser WD, Reid DM, Macdonald HM. Seasonal variation in 25(OH)D at Aberdeen (57°N) and bone health indicators--could holidays in the sun and cod liver oil supplements alleviate deficiency? *PLoS One*. 2013;8(1):e53381.

346. Mayer O, Seidlerová J, Wohlfahrt P, Filipovský J, Cífková R, Černá V, et al. Synergistic effect of low K and D vitamin status on arterial stiffness in a general population. *J Nutr Biochem*. 2017;46:83-9.

347. Mba CM, Koulman A, Forouhi NG, Sharp SJ, Imamura F, Jones K, et al. Association between circulating 25-hydroxyvitamin D and cardiometabolic risk factors in adults in rural and urban settings. *Nutr Diabetes*. 2022;12(1):34.

348. McCullough ML, Wang Y, Hartman TJ, Hodge RA, Flanders WD, Stevens VL, et al. The Cancer Prevention Study-3 FFQ is a reliable and valid measure of nutrient intakes among racial/ethnic subgroups, compared with 24-hour recalls and biomarkers. *J Nutr*. 2021;151(3):636-48.

349. Meems LMG, de Borst MH, Postma DS, Vonk JM, Kremer HPH, Schuttelaar M-LA, et al. Low levels of vitamin D are associated with multimorbidity: Results from the LifeLines Cohort Study. *Ann Med*. 2015;47(6):474-81.

350. Mehboobali N, Iqbal SP, Iqbal MP. High prevalence of vitamin D deficiency and insufficiency in a low income peri-urban community in Karachi. *The Journal of the Pakistan Medical Association*. 2015;65(9):946-9.

351. Menant JC, Close JC, Delbaere K, Sturnieks DL, Trollor J, Sachdev PS, et al. Relationships between serum vitamin D levels, neuromuscular and neuropsychological function and falls in older men and women. *Osteoporos Int*. 2012;23(3):981-9.

352. Menezes CA, Magalhães LB, da Silva JT, da Silva Lago RMR, Gomes AN, Ladeia AMT, et al. Ultra-processed food consumption is related to higher trans fatty acids, sugar intake, and micronutrient-impaired status in schoolchildren of Bahia, Brazil. *Nutrients*. 2023;15(2):381.

353. Meng L, Man Q, Yuan L, Shen L, Li W, Guo G, et al. Serum 25-hydroxyvitamin D and elderly skeletal muscle mass and function in urban North China. *Asia Pac J Clin Nutr*. 2017;26(5):849-55.

354. Meo SA, Baghazal Aa, Al Sinan A, Al Dabeeb D, Al Muhaya H, Al N, et al. Association of serum 25-hydroxy-vitamin D with lung function and fractional exhaled nitric oxide. *Biomedical Research*. 2016;27(4):1140-4.

355. Meoli M, Muggli F, Lava SAG, Bianchetti MG, Agostoni C, Kocher C, et al. Vitamin D status in adolescents during COVID-19 pandemic: A cross-sectional comparative study. *Nutrients*. 2021;13(5):1467.

356. Merchant RA, van Dam RM, Tan LWL, Lim MY, Low JL, Morley JE. Vitamin D binding protein and vitamin D levels in multi-ethnic population. *J Nutr Health Aging*. 2018;22(9):1060-5.

357. Meshkibaf MH, Mousazadeh S, Maleknia M, Takhshid MA. Association of vitamin D deficiency with vitamin D binding protein (DBP) and CYP2R1 polymorphisms in Iranian population. *Meta Gene*. 2021;27:100824.

358. Michaëlsson K, Wolk A, Byberg L, Mitchell A, Mallmin H, Melhus H. The seasonal importance of serum 25-hydroxyvitamin D for bone mineral density in older women. *J Intern Med*. 2017;281(2):167-78.

359. Michalus I, Fijalkowski B, Lupinska A, Golec J, Chlebna-sokol D. Assessment of the supplementation of vitamin D in children aged 9-15 years from Lodz. *Przeglad Pediatryczny*. 2013;43(2):74-81.

360. Middelkoop K, Walker N, Stewart J, Delport C, Jolliffe DA, Nuttall J, et al. Prevalence and determinants of vitamin D deficiency in 1825 Cape Town primary schoolchildren: A cross-sectional study. *Nutrients*. 2022;14(6):1263.

361. Mielgo-Ayuso J, Valtueña J, Cuenca-García M, Gottrand F, Breidenassel C, Ferrari M, et al. Regular breakfast consumption is associated with higher blood vitamin status in adolescents: The HELENA (Healthy Lifestyle in Europe by Nutrition in Adolescence) Study. *Public Health Nutr*. 2017;20(8):1393-404.

362. Miettinen ME, Kinnunen L, Leiviskä J, Keinänen-Kiukaanniemi S, Korpi-Hyövälti E, Niskanen L, et al. Association of serum 25-hydroxyvitamin D with lifestyle factors and metabolic and cardiovascular disease markers: Population-Based cross-sectional study (FIN-D2D). *PLoS One*. 2014;9(7):e100235.

363. Milagres LC, Filgueiras MDS, Rocha NP, Suhett LG, de Albuquerque FM, Juvanhol LL, et al. Cutoff point estimation for serum vitamin D concentrations to predict cardiometabolic risk in Brazilian children. *Eur J Clin Nutr*. 2020;74(12):1698-706.

364. Milagres LC, Filgueiras MDS, Rocha NP, Juvanhol LL, Franceschini SdCC, Farias de Novaes J. Vitamin D is associated with the hypertriglyceridemic waist phenotype in Brazilian children. *Journal of Public Health*. 2021;43(4):e570-e7.

365. Mirza AA, Rathi H, Dakshinamurthy S, Goyal B, Saha S, Saxena V, et al. Assessment of vitamin D levels and other bone related biochemical markers in healthy adults in rural population of Uttarakhand, India. *Ind J Clin Biochem*. 2023;38(3):316-23.

366. Mogire RM, Morovat A, Muriuki JM, Mentzer AJ, Webb EL, Kimita W, et al. Prevalence and predictors of vitamin D deficiency in young African children. *BMC Med*. 2021;19(1):115.

367. Mohammad M, Alourfi Z, Haddad S. Relationship between vitamin D receptor gene FokI polymorphism and 25-hydroxyvitamin D levels in apparently healthy Syrians. *Meta Gene*. 2021;29:100945.

368. Moran JM, Lopez-Arza LG, Lavado-Garcia JM, Pedrera-Canal M, Rey-Sanchez P, Rodriguez-Velasco FJ, et al. Hormonal relationships to bone mass in elderly Spanish men as influenced by dietary calcium and vitamin D. *Nutrients*. 2013;5(12):4924-37.

369. Muhairi SJ, Mehairi AE, Khouri AA, Naqbi MM, Maskari FA, Kaabi JA, et al. Vitamin D deficiency among healthy adolescents in Al Ain, United Arab Emirates. *BMC Public Health*. 2013;13:33.

370. Mukhopadhyay P, Ghosh S, Bhattacharjee K, Chowdhury S. Inverse relationship between 25 hydroxy vitamin D and parathormone: Are there two inflection points? *Indian J Endocrinol Metab*. 2019;23(4):422-7.

371. Mukhopadhyay P, Ghosh S, Pandit K, Chatterjee P, Mukherjee PS, Chowdhury S. Pandemic of vitamin D deficiency: Cardiometabolic concern or skeletal biochemical abnormality? *Indian J Endocrinol Metab*. 2019;23(2):215-21.

372. Mustafa A, Shekhar C. Concentration levels of serum 25-Hydroxyvitamin-D and vitamin D deficiency among children and adolescents of India: A descriptive cross-sectional study. *BMC Pediatr*. 2021;21(1):334.

373. Mutt SJ, Jokelainen J, Sebert S, Auvinen J, Järvelin M-R, Keinänen-Kiukaanniemi S, et al. Vitamin D status and components of metabolic syndrome in older subjects from Northern Finland (latitude 65 North). *Nutrients*. 2019;11(6):1229.

374. Naganuma J, Koyama S, Arisaka O, Yoshihara S. Low serum 25-hydroxyvitamin D level is associated with obesity and atherogenesis in adolescent boys. *Ann Pediatr Endocrinol Metab*. 2022;27(1):30-6.

375. Nakamura K, Saito T, Kobayashi R, Oshiki R, Oyama M, Nishiwaki T, et al. C-reactive protein predicts incident fracture in community-dwelling elderly Japanese women: The Muramatsu study. *Osteoporos Int*. 2011;22(7):2145-50.

376. Nakamura K, Kitamura K, Watanabe Y, Saito T, Takahashi A, Kobayashi R, et al. Predictors of decline in vitamin D status in middle-aged and elderly individuals: A 5-year follow-up study. *Br J Nutr*. 2020;124(7):729-35.

377. Nakamura H, Tsujiguchi H, Hara A, Kambayashi Y, Miyagi S, Thu Nguyen TT, et al. Dietary calcium intake and hypertension: Importance of serum concentrations of 25-hydroxyvitamin D. *Nutrients*. 2019;11(4):911.

378. Nakamura K, Hui S-P, Ukawa S, Okada E, Nakagawa T, Okabe H, et al. Serum 25-hydroxyvitamin D₃ levels and poor sleep quality in a Japanese population: The DOSANCO Health Study. *Sleep Med*. 2019;57:135-40.

379. Nakaoka K, Noda S, Tanabe R, Yamada A, Tsugawa N, Hosoi T, et al. A high-fat diet in the presence of vitamin D deficiency status is associated with a negative influence on calcaneal quantitative ultrasound parameters in young adults: A cross-sectional study. *Nutr Res*. 2021;86:88-95.

380. Nakhaee S, Ali Yaghoubi M, Zarban A, Amirabadizadeh A, Faghihi V, Yoosef Javadmoosavi S, et al. Vitamin D deficiency and its associated risk factors in normal adult population of Birjand, Iran. *Clin Nutr ESPEN*. 2019;32:113-7.

381. Nälsén C, Becker W, Pearson M, Ridefelt P, Lindroos AK, Kotova N, et al. Vitamin D status in children and adults in Sweden: Dietary intake and 25-hydroxyvitamin D concentrations in children aged 10–12 years and adults aged 18–80 years. *Journal of Nutritional Science*. 2020;9:e47.

382. Narchi H, Kochiyil J, Hamad SA, Yasin J, Laleye L, Dhaheri AA. Hypovitaminosis D in adolescent females – An analytical cohort study in the United Arab Emirates. *Paediatr Int Child Health*. 2015;35(1):36-43.

383. Navarrete-Reyes AP, García-Muñoz I, García-Lara JMA, Torres-Carrillo NM, Amieva H, Avila-Funes JA. 25-OH-Vitamin D is not associated with cognitive performance among Mexican community-dwelling older persons. *J Frailty Aging*. 2015;4(2):74-9.

384. Neyestani TR, Hajifaraji M, Omidvar N, Eshraghian MR, Shariatzadeh N, Kalayi A, et al. High prevalence of vitamin D deficiency in school-age children in Tehran, 2008: A red alert. *Public Health Nutr*. 2012;15(2):324-30.

385. Nguyen P, Pham L, Kien N, Nguyen D, Nguyen H, Lai N, et al. Vitamin D and bone mineral density status, and their correlation with bone turnover markers in healthy children aged 6-14 in Vietnam. *Curr Pediatr Res*. 2020;24(3):204-9.

386. Nguyen HTT, von Schoultz B, Nguyen TV, Dzung DN, Duc PTM, Thuy VT, et al. Vitamin D deficiency in Northern Vietnam: Prevalence, risk factors and associations with bone mineral density. *Bone*. 2012;51(6):1029-34.

387. Ní Chaoimh C, McCarthy EK, Hourihane JOB, Kenny LC, Irvine AD, Murray DM, et al. Low vitamin D deficiency in Irish toddlers despite northerly latitude and a high prevalence of inadequate intakes. *Eur J Nutr*. 2018;57(2):783-94.

388. Niafar M, Bahrami A, Aliasgharzadeh A, Aghamohammadzadeh N, Najafipour F, Mobasseri M. Vitamin D status in healthy postmenopausal Iranian women. *Journal of Research in Medical Sciences*. 2009;14(3):171-7.

389. Nichols EK, Khatib IMD, Aburto NJ, Serdula MK, Scanlon KS, Wirth JP, et al. Vitamin D status and associated factors of deficiency among Jordanian children of preschool age. *Eur J Clin Nutr*. 2015;69(1):90-5.

390. Nichols EK, Khatib IMD, Aburto NJ, Sullivan KM, Scanlon KS, Wirth JP, et al. Vitamin D status and determinants of deficiency among non-pregnant Jordanian women of reproductive age. *Eur J Clin Nutr*. 2012;66(6):751-6.

391. Nielsen NO, Bjerregaard P, Rønn PF, Friis H, Andersen S, Melbye M, et al. Associations between vitamin D status and type 2 diabetes measures among Inuit in Greenland may be affected by other factors. *PLoS One*. 2016;11(4):e0152763.

392. Niimi R, Chiba K, Okazaki N, Yonekura A, Tomita M, Osaki M. Relationships between QUS and HR-pQCT, DXA, and bone turnover markers. *J Bone Miner Metab*. 2022;40(5):790-800.

393. Nikooyeh B, Abdollahi Z, Hajifaraji M, Alavi-majd H, Salehi F, Yarparvar AH, et al. Vitamin D status and cardiometabolic risk factors across latitudinal gradient in Iranian adults: National food and nutrition surveillance. *Nutrition and Health*. 2017;23(2):87-94.

394. Nikooyeh B, Abdollahi Z, Hajifaraji M, Alavi-Majd H, Salehi F, Yarparvar AH, et al. Vitamin D status, latitude and their associations with some health parameters in children: National food and nutrition surveillance. *Journal of Tropical Pediatrics*. 2017;63(1):57-64.

395. Nurbazlin M, Chee WSS, Rokiah P, Tan ATB, Chew YY, Nusaibah ARS, et al. Effects of sun exposure on 25(OH) vitamin D concentration in urban and rural women in Malaysia. *Asia Pac J Clin Nutr*. 2013;22(3):391-9.

396. Ó Breasail M, Pearse C, Zengin A, Jarjou L, Cooper C, Ebeling PR, et al. Longitudinal change in bone density, geometry, and estimated bone strength in older men and women from the gambia: Findings from the gambian bone and muscle aging study (GamBAS). *Journal of Bone and Mineral Research*. 2023;38(1):48-58.

397. O'Brien DM, Thummel KE, Bulkow LR, Wang Z, Corbin B, Klejka J, et al. Declines in traditional marine food intake and vitamin D levels from the 1960s to present in young Alaska Native women. *Public Health Nutr*. 2017;20(10):1738-45.

398. Öberg J, Jorde R, Almås B, Emaus N, Grimnes G. Vitamin D deficiency and lifestyle risk factors in a Norwegian adolescent population. *Scand J Public Health*. 2014;42(7):593-602.

399. Okan F, Okan S, Zincir H. Effect of sunlight exposure on vitamin D status of individuals living in a nursing home and their own homes. *J Clin Densitom*. 2020;23(1):21-8.

400. Oliveira MA, Faerstein E, Koury JC, Pereira-Manfro WF, Milagres LG, Neto JFN, et al. Vitamin D is directly associated with favorable glycemic, lipid, and inflammatory profiles in individuals with at least one component of metabolic syndrome irrespective of total adiposity: Pró-Saúde Study, Brazil. *Nutr Res*. 2021;96:1-8.

401. Olsen KS, Aksnes L, Frøyland L, Lund E, Rylander C. Vitamin D status and PUFA ratios in a national representative cross-section of healthy, middle-aged Norwegian women – The Norwegian Women and Cancer Post-Genome Cohort. *Scand J Public Health*. 2014;42(8):814-20.

402. Orces CH. Vitamin D status among older adults residing in the Littoral and Andes Mountains in Ecuador. *Scientific World Journal*. 2015;2015:545297.

403. Oshiro CE, Hillier TA, Edmonds G, Peterson M, Hill PL, Hampson S. Vitamin D deficiency and insufficiency in Hawaii: Levels and sources of serum vitamin D in older adults. *American Journal of Human Biology* 2022;34(3):e23636.

404. Oudshoorn C, Hartholt KA, van Leeuwen JPTM, Colin EM, van der Velde N, van der Cammen TJM. Better knowledge on vitamin D and calcium in older people is associated with a higher serum vitamin D level and a higher daily dietary calcium intake. *Health Education Journal*. 2012;71(4):474-82.

405. Oussedik-Lehtihet S, Haouichat C, Hammoumraoui N, Ducros E, Gouhier-Kodas C, Lancrenon S, et al. Hypovitaminosis D and its associated factors in North Algerian postmenopausal women: Results of a cross-sectional study. *J Nutr Metab*. 2017;2017:9032141.

406. Overby NC, Johannesen E, Jensen G, Skjaevesland A-K, Haugen M. Test-retest reliability and validity of a web-based food-frequency questionnaire for adolescents aged 13-14 to be used in the Norwegian Mother and Child Cohort Study (MoBa). *Food Nutr Res*. 2014;58.

407. Paes-Silva RP, Tomiya MTO, Maio R, De Castro CMMB, Arruda IKGd, Diniz ADS. Prevalence and factors associated with fat-soluble vitamin deficiency in adolescents. *Nutr Hosp*. 2018;35(5):1153-62.

408. Pan T, Dasgupta A, Paul B, Bandyopadhyay L, Augustine ATV, Suman S. Bone health and its association with vitamin D and other covariates: A community-based study among women in a rural area of West Bengal. *Indian J Public Health*. 2020;64(2):135-40.

409. Panahi N, Fahimfar N, Roshani S, Arjmand B, Gharibzadeh S, Shafiee G, et al. Association of amino acid metabolites with osteoporosis, a metabolomic approach: Bushehr elderly health program. *Metabolomics*. 2022;18(8):63.

410. Pang X, Yang Z, Wang J, Duan Y, Zhao L, Yu D, et al. Relationship between serum 25OH-vitamin D₂ Level and vitamin D status of children aged 3–5 years in China. *Nutrients*. 2021;13(11):4135.

411. Pankiv V, Pankiv I. Association of vitamin D status with body mass index in adolescents in Ukraine. *Rom J Diabetes Nutr Metab Dis*. 2018;25(4):377-81.

412. Pannu PK, Zhao Y, Soares MJ, Piers LS, Ansari Z. The associations of vitamin D status and dietary calcium with the metabolic syndrome: An analysis of the Victorian Health Monitor survey. *Public Health Nutr*. 2017;20(10):1785-96.

413. Pantovic A, Zec M, Zekovic M, Obrenovic R, Stankovic S, Glibetic M. Vitamin D Is inversely related to obesity: Cross-sectional study in a small cohort of serbian adults. *Journal of the American College of Nutrition*. 2019;38(5):405-14.

414. Park HY, Kim JH, Bae S, Choi YY, Park JY, Hong Y-C. Interaction effect of serum 25-hydroxyvitamin D levels and CYP1A1, CYP1B1 polymorphisms on blood pressure in an elderly population. *J Hypertens*. 2015;33(1):69-76.

415. Park J-H, Hong IY, Chung JW, Choi HS. Vitamin D status in South Korean population: Seven-year trend from the KNHANES. *Medicine (Baltimore)*. 2018;97(26):e11032.

416. Passi-Solar Á, Margozzini P, Cortinez-O’Ryan A, Muñoz JC, Mindell JS. Nutritional and metabolic benefits associated with active and public transport: Results from the Chilean National Health Survey, ENS 2016–2017. *J Transp Health*. 2020;17:100819.

417. Patel P, Mughal MZ, Patel P, Yagnik B, Kajale N, Mandlik R, et al. Dietary calcium intake influences the relationship between serum 25-hydroxyvitamin D₃ (25OHD) concentration and parathyroid hormone (PTH) concentration. *Arch Dis Child*. 2016;101(4):316-9.

418. Patel PA, Patel PP, Mughal Z, Padidela R, Patel AD, Patwardhan V, et al. Interrelationship between serum 25-hydroxyvitamin D(3) concentration and lipid profiles in premenopausal Indian women. *Indian J Endocrinol Metab*. 2017;21(1):96-101.

419. Patel L, Vecchia CL, Alicandro G. Serum vitamin D and cardiometabolic risk factors in the UK population. *J Hum Nutr Diet*. 2023;36(3):1019-30.

420. Patriota P, Rezzi S, Guessous I, Marques-Vidal P. Association between anthropometric markers of adiposity, adipokines and vitamin D levels. *Sci Rep*. 2022;12(1):15435.

421. Pazaitou-Panayiotou K, Papapetrou PD, Chrisoulidou A, Konstantinidou S, Doumala E, Georgiou E, et al. Height, whole body surface area, gender, working outdoors, and sunbathing in previous summer are important determinants of serum 25-hydroxyvitamin D levels. *Exp Clin Endocrinol Diabetes*. 2012;120(1):14-22.

422. Peng H, Li H, Li C, Chao X, Zhang Q, Zhang Y. Association between vitamin D insufficiency and elevated serum uric acid among middle-aged and elderly Chinese Han women. *PLoS One*. 2013;8(4):e61159.

423. Pérez-Bravo F, Duarte L, Arredondo-Olguín M, Iñiguez G, Castillo-Valenzuela O. Vitamin D status and obesity in children from Chile. *Eur J Clin Nutr*. 2022;76(6):899-901.

424. Peters BSE, Verly Jr E, Marchioni DML, Fisberg M, Martini LA. The influence of breakfast and dairy products on dietary calcium and vitamin D intake in postpubertal adolescents and young adults. *J Hum Nutr Diet*. 2012;25(1):69-74.

425. Petrenya N, Lamberg-Allardt C, Melhus M, Broderstad AR, Brustad M. Vitamin D status in a multi-ethnic population of Northern Norway: The SAMINOR 2 Clinical Survey. *Public Health Nutr*. 2020;23(7):1186-200.

426. Petry N, Al-Maamary SA, Woodruff BA, Alghannami S, Al-Shammakhi SM, Al-Ghammari IK, et al. National prevalence of micronutrient deficiencies, anaemia, genetic blood disorders and over-and undernutrition in Omani women of reproductive age and preschool children. *Sultan Qaboos Univ Med J*. 2020;20(2):e151-e64.

427. Pilz S, Dobnig H, Tomaschitz A, Kienreich K, Meinitzer A, Friedl C, et al. Low 25-hydroxyvitamin D is associated with increased mortality in female nursing home residents. *J Clin Endocrinol Metab*. 2012;97(4):E653-7.

428. Poh BK, Rojroongwasinkul N, Nguyen BKL, Sandjaja, Ruzita AT, Yamborisut U, et al. 25-hydroxy-vitamin D demography and the risk of vitamin D insufficiency in the South East Asian Nutrition Surveys (SEANUTS). *Asia Pac J Clin Nutr*. 2016;25(3):538-48.

429. Poopedi MA, Norris SA, Pettifor JM. Is vitamin D status associated with non-communicable disease risk in children? A cohort study. *South Afr J Clin Nutr*. 2022;35(3):88-93.

430. Pouraram H, Djazayery A, Mohammad K, Parsaeian M, Abdollahi Z, Dorosty Motlagh A, et al. Second national integrated micronutrient survey in Iran: Study design and preliminary findings. *Arch Iran Med*. 2018;21(4):137-44.

431. Pulungan A, Andarie AA, Soesanti F, Yassien MR, de Bruin C, Wijaya A, et al. Anthropometric, biochemical and hormonal profiles of the partially admixed pygmoid group in Rampasasa (Flores, Indonesia). *J Pediatr Endocrinol Metab*. 2021;34(5):547-57.

432. Pusparini P, Merijanti LT, Sudharma NI. Increased matrix metalloproteinase-9 in male elderly with low 25-hydroxy-vitamin D. *Universa Medicina*. 2016;35(3):171-80.

433. Qiao Z, Shi L-X, Peng N-C, Xu S-J, Zhang M, Li H, et al. Serum 25 (OH) D level and parathyroid hormone in Chinese adult population: A cross-sectional study in Guiyang urban community from southeast of China. *Int J Endocrinol*. 2013;2013:150461.

434. Quah SW, Abdul Majid H, Al-Sadat N, Yahya A, Su TT, Jalaludin MY. Risk factors of vitamin D deficiency among 15-year-old adolescents participating in the Malaysian Health and Adolescents Longitudinal Research Team Study (MyHeARTs). *PLoS One*. 2018;13(7):e0200736.

435. Rabenberg M, Scheidt-Nave C, Busch MA, Thamm M, Rieckmann N, Durazo-Arvizu RA, et al. Implications of standardization of serum 25-hydroxyvitamin D data for the evaluation of vitamin D status in Germany, including a temporal analysis. *BMC Public Health*. 2018;18(1):845.

436. Rad MZ, Neyestani TR, Nikooyeh B, Shariatzadeh N, Kalayi A, Khalaji N, et al. Competitive protein-binding assay-based enzyme-immunoassay method, compared to high-pressure liquid chromatography, has a very lower diagnostic value to detect vitamin D deficiency in 9–12 years children. *Int J Prev Med*. 2015;6:67.

437. Rafraf M, Hasanabad SK, Jafarabadi MA. Vitamin D status and its relationship with metabolic syndrome risk factors among adolescent girls in Boukan, Iran. *Public Health Nutr*. 2014;17(4):803-9.

438. Rahmadhani R, Zaharan NL, Mohamed Z, Moy FM, Jalaludin MY. The associations between VDR BsmI polymorphisms and risk of vitamin D deficiency, obesity and insulin resistance in adolescents residing in a tropical country. *PLoS One*. 2017;12(6):e0178695.

439. Raina K, Verma H, Bhatia A. Prevalence of vitamin D deficiency in Jammu region. *JK Science*. 2014;16(1):21.

440. Ramankutty P, de Klerk NH, Miller M, Fenech M, O'Callaghan N, Armstrong BK, et al. Ultraviolet radiation exposure and serum vitamin D levels in young children. *Journal of Paediatrics and Child Health*. 2014;50(9):713-20.

441. Raposo L, Martins S, Ferreira D, Guimarães JT, Santos AC. Vitamin D, parathyroid hormone and metabolic syndrome - The PORMETS study. *BMC Endocr Disord*. 2017;17(1):71.

442. Rastad H, Mahdavi Gorabi A, Qorbani M, Seif E, Asayesh H, Motlagh ME, et al. Prevalence and determinants of vitamin D deficiency in Iranian children and adolescents: The CASPIAN-V study. *Journal of Diabetes and Metabolic Disorders*. 2021;20(1):383-9.

443. Raszewski G, Bojar I, Łukawski K, Bakalczuk G, Owoc A, Wdowiak A. 25-hydroxyvitamin D status and its impact on cognitive functions in postmenopausal woman. *Advances in Hygiene and Experimental Medicine*. 2019;73:588-97.

444. Rathnayake N, Alwis G, Lenora J, Lekamwasam S. Factors associated with measures of sarcopenia in pre and postmenopausal women. *BMC Women’s Health*. 2021;21:5.

445. Rathod A, Bonny O, Guessous I, Suter PM, Conen D, Erne P, et al. Association of urinary calcium excretion with serum calcium and vitamin D levels. *Clin J Am Soc Nephrol*. 2015;10(3):452-62.

446. Raulio S, Erlund I, Männistö S, Sarlio-Lähteenkorva S, Sundvall J, Tapanainen H, et al. Successful nutrition policy: Improvement of vitamin D intake and status in Finnish adults over the last decade. *Eur J Public Health*. 2017;27(2):268-73.

447. Rautenbach PH, Nienaber-Rousseau C, de Lange-Loots Z, Kruger IM, Pieters M. Associations between 25-hydroxyvitamin D and total and γ' fibrinogen and plasma clot properties and gene interactions in a group of healthy black South African women. *Front Cardiovasc Med*. 2022;9:868542.

448. Rehman G, Ahmad H, Ddin A, Rind HJ, Kashif S, Saleem A, et al. Demographic differences and trends of vitamin D levels among the teenaged girls in Balochistan. *Cureus*. 2020;12(12):e12335.

449. Rezaei OM, Sharifi F, Moodi M, Zarban A, Tahergorabi R, Tahergorabi Z. The prevalence and determinants of vitamin D status among older adults: Data from a longitudinal aging study. *Int J Prev Med*. 2023;14:27.

450. Riaz H, Finlayson AE, Bashir S, Hussain S, Mahmood S, Malik F, et al. Prevalence of vitamin D deficiency in Pakistan and implications for the future. *Expert Rev Clin Pharmacol*. 2016;9(2):329-38.

451. Rips L, Toom A, Kuik R, Varblane A, Mölder H, Kull M, et al. Severe deficiency of vitamin D has no negative effect on physical performance during military training. *J Sports Med Phys Fitness*. 2023;63(2):329-38.

452. Riverin B, Dewailly E, Côté S, Johnson-Down L, Morin S, Dodin S. Prevalence of vitamin D insufficiency among healthy school-age Cree children. *Paediatr Child Health*. 2014;19(3):e15-e9.

453. Riverin B, Dewailly E, Côté S, Johnson-Down L, Morin S, Dodin S. Prevalence of vitamin D insufficiency and associated factors among Canadian Cree: A cross-sectional study. *Can J Public Health*. 2013;104(4):e291-e7.

454. Robinson SL, Ramirez-Zea M, Roman AV, Villamor E, Group NMCMSSN. Correlates and family aggregation of vitamin D concentrations in school-aged children and their parents in nine Mesoamerican countries. *Public Health Nutr*. 2017;20(15):2754-65.

455. Rodríguez-Rodríguez E, Aparicio A, Andrés P, Ortega RM. Moderate vitamin D deficiency and inflammation related markers in overweight/obese schoolchildren. *Int J Vitam Nutr Res*. 2014;84(1-2):98-107.

456. Rodríguez-Rodríguez E, Ortega RM, González-Rodríguez LG, López-Sobaler AM, VALORNUT UCMRG. Vitamin D deficiency is an independent predictor of elevated triglycerides in Spanish school children. *Eur J Nutr*. 2011;50(5):373-8.

457. Rosendahl J, Fogelholm M, Pelkonen A, Mäkelä MJ, Mäkitie O, Erkkola M. A history of cow's milk allergy is associated with lower vitamin D status in schoolchildren. *Horm Res Paediatr*. 2017;88(3-4):244-50.

458. Ruelas YF, Equihua MDT, Solís NAJ, Rodríguez LMB, Enciso ID, Ramírez CAS. Vitamin D status and its relation to insulin resistance in a Mexican pediatric population. *J Pediatr Endocrinol Metab*. 2020;33(4):481-6.

459. Rufus-Membere P, Holloway-Kew KL, Diez-Perez A, Kotowicz MA, Pasco JA. Associations between bone impact microindentation and clinical risk factors for fracture. *Endocrinology*. 2019;160(9):2143-50.

460. Saberi-Karimian M, Ghazizadeh H, Zanganeh Baygi M, Minaie M, Sadeghi F, Pouraram H, et al. The national health program for vitamin D supplementation in a developing country. *Clin Nutr ESPEN*. 2023;54:52-9.

461. Sacheck J, Goodman E, Chui K, Chomitz V, Must A, Economos C. Vitamin D deficiency, adiposity, and cardiometabolic risk in urban schoolchildren. *J Pediatr*. 2011;159(6):945-50.

462. Sadiq M, Sarwar MS, Rehman HU. A cross-sectional study to determine the rate of shortage of vitamin D especially in the low standard urban community. *Indo Am J P Sci*. 2018;5(12):17145-50.

463. Saki F, Dabbaghmanesh MH, Omrani GR, Bakhshayeshkaram M. Vitamin D deficiency and its associated risk factors in children and adolescents in southern Iran. *Public Health Nutr*. 2017;20(10):1851-6.

464. Salem-Sokhn E, Salami A, Fawaz M, Eid AH, El Shamieh S. Helicobacter pylori interacts with serum vitamin D to influence hypertension. *Curr Aging Sci*. 2021;14(1):26-31.

465. Sales CH, de Mello Fontanelli M, Rogero MM, Sarti FM, Fisberg RM. Dietary inadequacies overestimate the blood deficiencies of magnesium, zinc, and vitamins A, C, E, and D among residents of Sao Paulo. *Clin Nutr ESPEN*. 2023;53:196-205.

466. Samefors M, Östgren CJ, Mölstad S, Lannering C, Midlöv P, Tengblad A. Vitamin D deficiency in elderly people in Swedish nursing homes is associated with increased mortality. *European Journal of Endocrinology*. 2014;170(5):667-75.

467. Santos BR, Costa NC, Silva TR, Oppermann K, Magalhães JA, Casanova G, et al. Prevalence of vitamin D deficiency in women from southern Brazil and association with vitamin D-binding protein levels and GC-DBP gene polymorphisms. *PLoS One*. 2019;14(12):e0226215.

468. Santos A, Amaral TF, Guerra RS, Sousa AS, Álvares L, Moreira P, et al. Vitamin D status and associated factors among Portuguese older adults: Results from the Nutrition UP 65 cross-sectional study. *BMJ Open*. 2017;7(6):e016123.

469. Santos Araújo EPD, Queiroz DJM, Neves JPR, Lacerda LMd, Gonçalves MDCR, Carvalho ATd. Prevalence of hypovitaminosis D and associated factors in adolescent students of a capital of Northeastern Brazil. *Nutr Hosp*. 2017;34(5):1416-23.

470. Sanwalka N, Khadilkar A, Chiplonkar S, Khatod K, Phadke N, Khadilkar V. Vitamin D receptor gene polymorphisms and bone mass indices in post-menarchal Indian adolescent girls. *J Bone Miner Metab*. 2013;31(1):108-15.

471. Sari DK, Sari LM, Laksmi LI, Farhat. The moderate correlation between 25(OH)D serum and saliva in healthy people with low vitamin D intake. *Int J Gen Med*. 2021;14:841-50.

472. Sarma D, Saikia UK, Baro A. Vitamin D status of school children in and around Guwahati. *Indian J Endocrinol Metab*. 2019;23(1):81-5.

473. Schramm S, Lahner H, Jöckel K-H, Erbel R, Führer D, Moebus S. Impact of season and different vitamin D thresholds on prevalence of vitamin D deficiency in epidemiological cohorts—A note of caution. *Endocrine*. 2017;56(3):658-66.

474. Seo JA, Cho H, Eun CR, Yoo HJ, Kim SG, Choi KM, et al. Association between visceral obesity and sarcopenia and vitamin D deficiency in older Koreans: The Ansan geriatric study. *J Am Geriatr Soc*. 2012;60(4):700-6.

475. Seo JA, Eun CR, Cho H, Lee SK, Yoo HJ, Kim SG, et al. Low vitamin D status is associated with nonalcoholic fatty liver disease independent of visceral obesity in Korean adults. *PLoS One*. 2013;8(10):e75197.

476. Shah TH, Hassan M, Siddiqui TS. Subclinical rickets. *Pak J Med Sci*. 2014;30(4):854-7.

477. Shaheen SO, Jameson KA, Robinson SM, Boucher BJ, Syddall HE, Sayer AA, et al. Relationship of vitamin D status to adult lung function and COPD. *Thorax*. 2011;66(8):692-8.

478. Shanyhin A, Babienko V, Vatan M, Rozhnova A, Strakhov Y. Hygienic assessment of the prevalence of vitamin D deficiency states associated with dyslipidemia in the adult population of Southern Ukraine. *Georgian Med News*. 2022(332):93-8.

479. Sharma N, Cooper R, Kuh D, Shah I. Associations of statin use with motor performance and myalgia may be modified by 25-hydroxyvitamin D: Findings from a British birth cohort. *Sci Rep*. 2017;7(1):6578.

480. Sharma N, Sharma B, Singh G, Gupta A, Sharma R, Kapil U. Vitamin D status in cold trans-Himalayan deserts at altitude of 4000 meter and above in India. *Indian J Community Health*. 2018;30(4):400-2.

481. Sheen YH, Lee E, Kang M-J, Yu H-S, Ahn K, Kim KW, et al. Interaction between 25-hydroxyvitamin D and variants at 17q12-21 on respiratory infections. *Pediatr Pulmonol*. 2016;51(9):958-67.

482. Shen J, Fu S, Song Y. Relationship of Fibroblast Growth Factor 23 (FGF-23) serum levels with low bone mass in postmenopausal women. *Journal of Cellular Biochemistry*. 2017;118(12):4454-9.

483. Sherchand O, Baranwal JK, Gelal B. Epidemiology and determinants of vitamin D deficiency in Eastern Nepal: A community-based, cross-sectional study. *Int J Endocrinol*. 2022;2022:1063163.

484. Sherief LM, Ali A, Gaballa A, Abdellatif GM, Kamal NM, Afify MR, et al. Vitamin D status and healthy Egyptian adolescents: Where do we stand? *Medicine (Baltimore)*. 2021;100(29):e26661.

485. Shetty S, Kapoor N, Naik D, Asha HS, Prabu S, Thomas N, et al. Osteoporosis in healthy South Indian males and the influence of life style factors and vitamin D status on bone mineral density. *Journal of Osteoporosis*. 2014;2014:723238.

486. Shi J, He L, Yu D, Ju L, Guo Q, Piao W, et al. Prevalence and correlates of metabolic syndrome and its components in chinese children and adolescents aged 7-17: The China national nutrition and health survey of children and lactating mothers from 2016-2017. *Nutrients*. 2022;14(16):3348.

487. Shin YH, Ha EK, Kim JH, Yon DK, Lee SW, Sim HJ, et al. Serum vitamin D level is associated with smell dysfunction independently of aeroallergen sensitization, nasal obstruction, and the presence of allergic rhinitis in children. *Pediatr Allergy Immunol*. 2021;32(1):116-23.

488. Shinkov A, Borissova AM, Dakovska L, Vlahov J, Kassabova L, Svinarov D. Winter 25-hydroxyvitamin D levels in young urban adults are affected by smoking, body mass index and educational level. *Eur J Clin Nutr*. 2015;69(3):355-60.

489. Shridhar K, Kinra S, Gupta R, Khandelwal S, D P, Cox SE, et al. Serum calcium concentrations, chronic inflammation and glucose metabolism: A cross-sectional analysis in the Andhra Pradesh Children and Parents Study (APCaPS). *Curr Dev Nutr*. 2019;3(3):nzy085.

490. Silva CC, Gavinha S, Manso MC, Rodrigues R, Martins S, Guimarães JT, et al. Serum levels of vitamin D and dental caries in 7-year-old children in Porto Metropolitan area. *Nutrients*. 2021;13(1):166.

491. Singh A, Baalasubramanian S, Kalaivani M, Kapoor R, Bhagwat K, Ghosh-Jerath S. Standardisation and application of a novel multiplex assay for estimating micronutrient status and inflammatory markers in women of Sauria Paharia and Santhal tribes of Jharkhand. *Br J Nutr*. 2022;128(12):2464-79.

492. Singh S, Jalan D, Bhardwaj P, Sharma P, Elhence A. Cross sectional study of vitamin D levels in Western Rajasthan and meta-analysis for estimation of vitamin D levels. *Ind J Clin Biochem*. 2022:1-7.

493. Smith G, Wimalawansa SJ, Laillou A, Sophonneary P, Un S, Hong R, et al. High prevalence of vitamin D deficiency in cambodian women: A common deficiency in a sunny country. *Nutrients*. 2016;8(5):290.

494. Soininen S, Eloranta A-M, Lindi V, Venäläinen T, Zaproudina N, Mahonen A, et al. Determinants of serum 25-hydroxyvitamin D concentration in Finnish children: The Physical Activity and Nutrition in Children (PANIC) study. *Br J Nutr*. 2016;115(6):1080-91.

495. Sonderman JS, Munro HM, Blot WJ, Signorello LB. Reproducibility of serum 25-hydroxyvitamin D and vitamin D-binding protein levels over time in a prospective cohort study of black and white adults. *Am J Epidemiol*. 2012;176(7):615-21.

496. Song BM, Kim HC, Choi DP, Oh SM, Suh I. Association between serum 25-hydroxyvitamin D level and insulin resistance in a rural population. *Yonsei Med J*. 2014;55(4):1036-41.

497. Song H-R, Kweon S-S, Choi J-S, Rhee J-A, Lee Y-H, Nam H-S, et al. High prevalence of vitamin D deficiency in adults aged 50 years and older in Gwangju, Korea: The Dong-gu Study. *J Korean Med Sci*. 2014;29(1):149-52.

498. Soontrapa S, Soontrapa S, Chaikitpinyo S. Prevalence of vitamin D insufficiency among elderly males living in rural Khon Kaen province, Northeast Thailand. *J Med Assoc Thai*. 2015;98 Suppl 8:S21-S5.

499. Souberbielle J-C, Massart C, Brailly-Tabard S, Cavalier E, Chanson P. Prevalence and determinants of vitamin D deficiency in healthy French adults: The VARIETE study. *Endocrine*. 2016;53(2):543-50.

500. Sousa SES, Sales MC, Araújo JRT, Sena-Evangelista KCM, Lima KC, Pedrosa LFC. High prevalence of hypovitaminosis D in institutionalized elderly individuals is associated with summer in a region with high ultraviolet radiation levels. *Nutrients*. 2019;11(7):1516.

501. Srimani S, Saha I, Chaudhuri D. Prevalence and association of metabolic syndrome and vitamin D deficiency among postmenopausal women in a rural block of West Bengal, India. *PLoS One*. 2017;12(11):e0188331.

502. Standahl Olsen K, Rylander C, Brustad M, Aksnes L, Lund E. Plasma 25 hydroxyvitamin D level and blood gene expression profiles: A cross-sectional study of the Norwegian Women and Cancer Post-genome Cohort. *Eur J Clin Nutr*. 2013;67(7):773-8.

503. Stephan Y, Sutin AR, Luchetti M, Aschwanden D, Terracciano A. The mediating role of biomarkers in the association between subjective aging and episodic memory. *The Journals of Gerontology Series B, Psychological Sciences and Social Sciences*. 2023;78(2):242-52.

504. Su Y, Leung J, Lee J, Ho K-f, Kwok T. The effect of physical activity on dose-relationship between serum 25-hydroxyvitamin D and cardiovascular health events in older adults. *Nutr Metab Cardiovasc Dis*. 2020;30(4):656-65.

505. Suárez-Calleja C, Aza-Morera J, Iglesias-Cabo T, Tardón A. Vitamin D, pregnancy and caries in children in the INMA-Asturias birth cohort. *BMC Pediatr*. 2021;21(1):380.

506. Sulimani RA, Mohammed AG, Alfadda AA, Alshehri SN, Al-Othman AM, Al-Daghri NM, et al. Vitamin D deficiency and biochemical variations among urban Saudi adolescent girls according to season. *Saudi Med J*. 2016;37(9):1002-8.

507. Summerhays E, Eliasson M, Lundqvist R, Söderberg S, Zeller T, Oskarsson V. Time trends of vitamin D concentrations in Northern Sweden between 1986 and 2014: A population-based cross-sectional study. *Eur J Nutr*. 2020;59(7):3037-44.

508. Sun X, Cao Z-B, Tanisawa K, Ito T, Oshima S, Higuchi M. The relationship between serum 25-hydroxyvitamin D concentration, cardiorespiratory fitness, and insulin resistance in Japanese men. *Nutrients*. 2014;7(1):91-102.

509. Sundarakumar JS, Shahul Hameed SK, Team SS, Ravindranath V. Burden of vitamin D, vitamin B12 and folic acid deficiencies in an aging, rural Indian community. *Front Public Health*. 2021;9:707036.

510. Sung M, Jee HM, Kim JH, Ha EK, Shin YH, Kim JH, et al. Serum vitamin D level mitigates fractional exhaled nitric oxide linked to bisphenol-A in school-aged children. *Eur Rev Med Pharmacol Sci*. 2022;26(5):1640-7.

511. Surve S, Begum S, Chauhan S, Khatkhatay MI, Joshi B. Determinants of vitamin D deficiency among under-five children in urban slums of Mumbai, India. *Indian Pediatr*. 2021;58(9):888-9.

512. Suryanarayana P, Arlappa N, Sai Santhosh V, Balakrishna N, Lakshmi Rajkumar P, Prasad U, et al. Prevalence of vitamin D deficiency and its associated factors among the urban elderly population in Hyderabad metropolitan city, South India. *Annals of Human Biology*. 2018;45(2):133-9.

513. Szabó B, Tabák ÁG, Toldy E, Szekeres L, Szili B, Bakos B, et al. The role of serum total and free 25-hydroxyvitamin D and PTH values in defining vitamin D status at the end of winter: A representative survey. *J Bone Miner Metab*. 2017;35(1):83-90.

514. Szili B, Szabó B, Horváth P, Bakos B, Kirschner G, Kósa JP, et al. Impact of genetic influence on serum total- and free 25-hydroxyvitamin-D in humans. *J Steroid Biochem Mol Biol*. 2018;183:62-7.

515. Szternel L, Krintus M, Bergmann K, Derezinski T, Sypniewska G. Association between fasting glucose concentration, lipid profile and 25(OH)D status in children aged 9-11. *Nutrients*. 2018;10(10):1359.

516. Talaei A, Yadegari N, Rafee M, Rezvanfar MR, Moini A. Prevalence and cut-off point of vitamin D deficiency among secondary students of Arak, Iran in 2010. *Indian J Endocrinol Metab*. 2012;16(5):786-90.

517. Tamer G, Mesci B, Tamer I, Kilic D, Arik S. Is vitamin D deficiency an independent risk factor for obesity and abdominal obesity in women? *Endokrynol Pol*. 2012;63(3):196-201.

518. Tandon VR, Sharma S, Mahajan S, Raina K, Mahajan A, Khajuria V, et al. Prevalence of vitamin D deficiency among Indian menopausal women and its correlation with diabetes: A first Indian cross sectional data. *J Midlife Health*. 2014;5(3):121-5.

519. Teng J, Li J, Yang T, Cui J, Xia X, Chen G, et al. Long-term exposure to air pollution and lung function among children in China: Association and effect modification. *Front Public Health*. 2022;10:988242.

520. Terán G, Cuna W, Brañez F, Persson KEM, Rottenberg ME, Nylén S, et al. Differences in nutritional and health status in school children from the highlands and lowlands of Bolivia. *The American Journal of Tropical Medicine and Hygiene*. 2018;98(1):326-33.

521. Terock J, Hannemann A, Weihs A, Janowitz D, Grabe HJ. Alexithymia is associated with reduced vitamin D levels, but not polymorphisms of the vitamin D binding-protein gene. *Psychiatr Genet*. 2021;31(4):126-34.

522. Thompson M, Aitken D, Balogun S, Cicuttini F, Jones G. Population vitamin D stores are increasing in Tasmania, and this is associated with less BMD loss over 10 years. *J Clin Endocrinol Metab*. 2021;106(8):e2995-e3004.

523. Thorisdottir B, Gunnarsdottir I, Steingrimsdottir L, Palsson GI, Birgisdottir BE, Thorsdottir I. Vitamin D intake and status in 6-year-old Icelandic children followed up from infancy. *Nutrients*. 2016;8(2):75.

524. Thornton KA, Marín C, Mora-Plazas M, Villamor E. Vitamin D deficiency associated with increased incidence of gastrointestinal and ear infections in school-age children. *Pediatr Infect Dis J*. 2013;32(6):585-93.

525. Tian L, Yang R, Wei L, Liu J, Yang Y, Shao F, et al. Prevalence of osteoporosis and related lifestyle and metabolic factors of postmenopausal women and elderly men: A cross-sectional study in Gansu province, Northwestern of China. *Medicine (Baltimore)*. 2017;96(43):e8294.

526. Tokida R, Uehara M, Nakano M, Suzuki T, Sakai N, Ikegami S, et al. Reference values for bone metabolism in a Japanese cohort survey randomly sampled from a basic elderly resident registry. *Sci Rep*. 2021;11(1):7822.

527. Tolppanen A-M, Fraser A, Fraser WD, Lawlor DA. Risk factors for variation in 25-hydroxyvitamin D₃ and D₂ concentrations and vitamin D deficiency in children. *J Clin Endocrinol Metab*. 2012;97(4):1202-10.

528. Trummer O, Schwetz V, Walter-Finell D, Lerchbaum E, Renner W, Gugatschka M, et al. Allelic determinants of vitamin D insufficiency, bone mineral density, and bone fractures. *J Clin Endocrinol Metab*. 2012;97(7):E1234-E40.

529. Tse SM, Weiler H, Kovesi T. Food insecurity, vitamin D insufficiency and respiratory infections among Inuit children. *Int J Circumpolar Health*. 2016;75:29954.

530. Tsugawa N, Kuwabara A, Ogasawara H, Nishino M, Nakagawa K, Kamao M, et al. Vitamin D status in Japanese young women in 2016-2017 and 2020: Seasonal variation and the effect of lifestyle including changes caused by the COVID-19 pandemic. *J Nutr Sci Vitaminol (Tokyo)*. 2022;68(3):172-80.

531. Tuffaha M, El Bcheraoui C, Daoud F, Al Hussaini HA, Alamri F, Al Saeedi M, et al. Deficiencies under plenty of sun: Vitamin D status among adults in the kingdom of Saudi Arabia, 2013. *N Am J Med Sci*. 2015;7(10):467-75.

532. Tung KTS, Wong RS, Tsang HW, Chan BNK, Wong SY, So H-K, et al. An assessment of risk factors for insufficient levels of vitamin D during early infancy. *Nutrients*. 2021;13(4):1068.

533. Vahid F, Hoge A, Hébert JR, Bohn T. Association of diet quality indices with serum and metabolic biomarkers in participants of the ORISCAV-LUX-2 study. *Eur J Clin Nutr*. 2023;62(5):2063-85.

534. Vajdi M, Nikniaz L, Pour Asl AM, Abbasalizad Farhangi M. Lifestyle patterns and their nutritional, socio-demographic and psychological determinants in a community-based study: A mixed approach of latent class and factor analyses. *PLoS One*. 2020;15(7):e0236242.

535. VanSickle JS, Srivastava T, Garg U, Rezaiekhaligh MH, Alon US. Comparing directly measured versus mathematically calculated free serum 25-hydroxy vitamin D level in children. *J Bone Miner Metab*. 2020;38(2):271-4.

536. Vázquez-Lorente H, Herrera-Quintana L, Molina-López J, Gamarra-Morales Y, López-González B, Planells E. Relationship between body composition and biochemical parameters with antioxidant status in a healthy cohort of postmenopausal women. *Metabolites*. 2022;12(8):746.

537. Verhoeven V, Vanpuyenbroeck K, Lopez-Hartmann M, Wens J, Remmen R. Walk on the sunny side of life — Epidemiology of hypovitaminosis D and mental health in elderly nursing home residents. *J Nutr Health Aging*. 2012;16(4):417-20.

538. Vignali E, Macchia E, Cetani F, Reggiardo G, Cianferotti L, Saponaro F, et al. Development of an algorithm to predict serum vitamin D levels using a simple questionnaire based on sunlight exposure. *Endocrine*. 2017;55(1):85-92.

539. Vioque J, Garcia-de-la-Hera M, Gonzalez-Palacios S, Torres-Collado L, Notario-Barandiaran L, Oncina-Canovas A, et al. Reproducibility and validity of a short food frequency questionnaire for dietary assessment in children aged 7–9 years in Spain. *Nutrients*. 2019;11(4):933.

540. Voortman T, van den Hooven EH, Heijboer AC, Hofman A, Jaddoe VWV, Franco OH. Vitamin D deficiency in school-age children is associated with sociodemographic and lifestyle factors. *J Nutr*. 2015;145(4):791-8.

541. Wakayo T, Belachew T, Vatanparast H, Whiting SJ. Vitamin D deficiency and its predictors in a country with thirteen months of sunshine: The case of school children in central Ethiopia. *PLoS One*. 2015;10(3):e0120963.

542. Wang D, Lin H, Xia M, Aleteng Q, Li X, Ma H, et al. Vitamin D levels are inversely associated with liver fat content and risk of non-alcoholic fatty liver disease in a Chinese middle-aged and elderly population: The Shanghai Changfeng Study. *PLoS One*. 2016;11(6):e0157515.

543. Wang Y, Wang Y-J, Zhan J-K, Tang Z-Y, Huang W, Tan P, et al. Vitamin D binding protein affects the correlation of 25 (OH) D and frailty in the older men. *Int J Endocrinol*. 2014;2014:543783.

544. Wang Y, Gao X, Lv J, Zeng Y, Li Q, Wang L, et al. Gut microbiome signature are correlated with bone mineral density alterations in the Chinese elders. *Frontiers in Cellular and Infection Microbiology*. 2022;12:827575.

545. Wang J, Chen L, Zhang Y, Li C-g, Zhang H, Wang Q, et al. Association between serum vitamin B6 concentration and risk of osteoporosis in the middle-aged and older people in China: A cross-sectional study. *BMJ Open*. 2019;9(7):e028129.

546. Wang T, Sun H, Ge H, Liu X, Yu F, Han H, et al. Association between vitamin D and risk of cardiovascular disease in Chinese rural population. *PLoS One*. 2019;14(5):e0217311.

547. Wang Y, Han H, Wang J, Shen F, Yu F, Wang L, et al. Polymorphisms in CYP2R1 gene associated with serum vitamin D levels and status in a Chinese rural population. *Biomed Environ Sci*. 2019;32(7):550-3.

548. Wang X, Liu L, Li P, Ma J, Jiang R, Wang R, et al. Reference and influential factors of serum bone markers in Chinese adolescents. *Sci Rep*. 2017;7(1):17340.

549. Wang J, Wang H, Chang S, Zhao L, Fu P, Yu W, et al. The influence of malnutrition and micronutrient status on anemic risk in children under 3 years old in poor areas in China. *PLoS One*. 2015;10(10):e0140840.

550. Weiler HA, Sarafin K, Martineau C, Daoust JL, Esslinger K, Greene-Finestone LS, et al. Vitamin D status of people 3 to 79 years of age from the Canadian Health Measures Survey 2012-2019. *J Nutr*. 2023;153(4):1150-61.

551. Weldegiorgis TZ, Hidru TH, Yang X-L, Xia Y-L, Ma L, Li H-H. Association between serum 25-hydroxyvitamin D concentrations and metabolic syndrome in the middle-aged and elderly Chinese population in Dalian, Northeast China: A cross-sectional study. *Journal of Diabetes Investigation*. 2020;11(1):184-91.

552. Wolters M, Intemann T, Russo P, Moreno LA, Molnár D, Veidebaum T, et al. 25-Hydroxyvitamin D reference percentiles and the role of their determinants among European children and adolescents. *Eur J Clin Nutr*. 2022;76(4):564-73.

553. Wyskida M, Owczarek A, Szybalska A, Brzozowska A, Szczerbowska I, Wieczorowska-Tobis K, et al. Socio-economic determinants of vitamin D deficiency in the older Polish population: Results from the PolSenior study. *Public Health Nutr*. 2018;21(11):1995-2003.

554. Xia Z, Man Q, Li L, Song P, Jia S, Song S, et al. Vitamin D receptor gene polymorphisms modify the association of serum 25-hydroxyvitamin D levels with handgrip strength in the elderly in Northern China. *Nutrition*. 2019;57:202-7.

555. Xiao P, Dong H, Li H, Yan Y, Cheng H, Liu J, et al. Adequate 25-hydroxyvitamin D levels are inversely associated with various cardiometabolic risk factors in Chinese children, especially obese children. *BMJ Open Diabetes Res Care*. 2020;8(1):e000846.

556. Xiao P, Cheng H, Li H, Zhao X, Hou D, Xie X, et al. Vitamin D trajectories and cardiometabolic risk factors during childhood: A large population-based prospective cohort study. *Front Cardiovasc Med*. 2022;9:836376.

557. Xie Z, Xia W, Zhang Z, Wu W, Lu C, Tao S, et al. Prevalence of vitamin D inadequacy among Chinese postmenopausal women: A nationwide, multicenter, cross-sectional study. *Front Endocrinol (Lausanne)*. 2019;9:782.

558. Xu Y, Gao C, He J, Gu W, Yi C, Chen B, et al. Sclerostin and its associations with bone metabolism markers and sex hormones in healthy community-dwelling elderly individuals and adolescents. *Front Cell Dev Biol*. 2020;8:57.

559. Yan D-D, Wang J, Hou X-H, Bao Y-Q, Zhang Z-L, Hu C, et al. Association of serum uric acid levels with osteoporosis and bone turnover markers in a Chinese population. *Acta Pharmacol Sin*. 2018;39(4):626-32.

560. Yan X, Zhang N, Cheng S, Wang Z, Qin Y. Gender differences in vitamin D status in China. *Med Sci Monit*. 2019;25:7094-9.

561. Yang C, Liu X, Li J, Yan N, Dang Y, Chang Z, et al. Association of serum vitamin D and estradiol levels with metabolic syndrome in rural women of Northwest China: A cross-sectional study. *Metab Syndr Relat Disord*. 2022;20(3):182-9.

562. Yang H-K, Choi J, Kim WK, Lee S-Y, Park YM, Han MY, et al. The association between hypovitaminosis D and pediatric allergic diseases: A Korean nationwide population-based study. *Allergy Asthma Proc*. 2016;37(4):64-9.

563. Yao Y, Fu S, Li N, Hu F, Zhang H, Zhu Q, et al. Sex, residence and fish intake predict vitamin D status in Chinese centenarians. *J Nutr Health Aging*. 2019;23(2):165-71.

564. Yao T-C, Tu Y-L, Chang S-W, Tsai H-J, Gu P-W, Ning H-C, et al. Suboptimal vitamin D status in a population-based study of Asian children: Prevalence and relation to allergic diseases and atopy. *PLoS One*. 2014;9(6):e99105.

565. Yeap SS, Thambiah SC, Suppiah S, Md-Said S, Appannah G, Samsudin IN, et al. Asymptomatic morphometric vertebral fractures and its associated factors: A cross-sectional study among adults in a selected urban area in Selangor, Malaysia. *PLoS One*. 2021;16(7):e0255069.

566. Yoshihara A, Iwasaki M, Miyazaki H, Nakamura K. Association between low renal function and tooth loss over 5 years. *Gerodontology*. 2014;31(2):111-6.

567. Yoshimura N, Muraki S, Oka H, Morita M, Yamada H, Tanaka S, et al. Profiles of vitamin D insufficiency and deficiency in Japanese men and women: Association with biological, environmental, and nutritional factors and coexisting disorders: The ROAD study. *Osteoporos Int*. 2013;24(11):2775-87.

568. Younes N, El Hajj M-A, Bizdikian AJ, Gannagé-Yared M-H. An epidemiological evaluation of fractures and its determinants among Lebanese schoolchildren: A cross-sectional study. *Arch Osteoporos*. 2019;14(1):9.

569. Young MF, Ou J, Duong C, Luo H, Beyh YS, Meng J, et al. Assessment of Vitamin D status and association with inflammation: Biomarkers Reflecting Inflammation and Nutritional Determinants of Anemia (BRINDA) project. *Am J Clin Nutr*. 2023;117(1):175-81.

570. Yousef FM. Associations factors affecting on osteoporosis in postmenopausal women in Saudi Arabian, Jeddah. *International Journal of Pharmaceutical Research & Allied Sciences*. 2017;6(2):204-12.

571. Yuan C, Wang J, Zhang W, Yi H, Shu B, Li C, et al. Effects of obesity with reduced 25(OH)D levels on bone health in elderly Chinese people: A nationwide cross-sectional study. *Frontiers in Immunology*. 2023;14:1162175.

572. Yunara S, Widajanti N, Ichwani J. Association of 25-hydroxyvitamin D concentration and frailty degree in elderly community in Surabaya. *International Journal of Pharmaceutical Research*. 2020;12(4):1534-40.

573. Zamoiski RD, Guallar E, García-Vargas GG, Rothenberg SJ, Resnick C, Andrade MR, et al. Association of arsenic and metals with concentrations of 25-hydroxyvitamin D and 1,25-dihydroxyvitamin D among adolescents in Torreón, Mexico. *Environ Health Perspect*. 2014;122(11):1233-8.

574. Zelzer S, Hofer E, Meinitzer A, Fritz-Petrin E, Simstich S, Goessler W, et al. Association of vitamin D metabolites with cognitive function and brain atrophy in elderly individuals - The Austrian stroke prevention study. *Aging (Albany NY)*. 2021;13(7):9455-67.

575. Zhang D, Cheng C, Wang Y, Xue Y, Liu Y, Li W, et al. Serum 25-Hydroxyvitamin D concentrations and cardiometabolic biomarkers in Chinese rural population. *Horm Metab Res*. 2021;53(2):105-11.

576. Zhang X, Chen M, Duan R, Xue H, Luo J, Lv X, et al. The Nutrition and Health in Southwest China (NHSC) study: Design, implementation, and major findings. *Eur J Clin Nutr*. 2021;75(2):299-306.

577. Zhang FF, Al Hooti S, Al Zenki S, Alomirah H, Jamil KM, Rao A, et al. Vitamin D deficiency is associated with high prevalence of diabetes in Kuwaiti adults: Results from a national survey. *BMC Public Health*. 2016;16(1):100.

578. Zhang H-q, Teng J-h, Li Y, Li X-x, He Y-h, He X, et al. Vitamin D status and its association with adiposity and oxidative stress in schoolchildren. *Nutrition*. 2014;30(9):1040-4.

579. Zhao N, Zhen D, Zhao Z, Fu S, Guan C, Liu L, et al. 25-Hydroxyvitamin D and incidence of type 2 diabetes from a Chinese cohort study. *J Nutr Sci Vitaminol (Tokyo)*. 2022;68(1):8-15.

580. Zhou SJ, Skeaff M, Makrides M, Gibson R. Vitamin D status and its predictors among pre-school children in Adelaide. *Journal of Paediatrics and Child Health*. 2015;51(6):614-9.

581. Zhou W, Deng Y, Zhang C, Dai H, Guan L, Luo X, et al. Chlorpyrifos residue level and ADHD among children aged 1-6 years in rural China: A cross-sectional study. *Frontiers in Pediatrics*. 2022;10:952559.

582. Zhu W, Heil DP. Associations of vitamin D status with markers of metabolic health: A community-based study in Shanghai, China. *Diabetes Metab Syndr*. 2018;12(5):727-32.

583. Zhuang Z, Yu C, Guo Y, Bian Z, Yang L, Millwood IY, et al. Metabolic signatures of genetically elevated vitamin D among Chinese: Observational and Mendelian randomization study. *J Clin Endocrinol Metab*. 2021;106(8):e3249-e60.

584. Zou Y, Liu Z, Li H, Hou La, Pang J, Liu X, et al. Evaluation of bone metabolism-associated biomarkers in Tibet, China. *J Clin Lab Anal*. 2021;35(12):e24068.

585. Zumaraga MP, Concepcion MA, Duante C, Rodriguez M. Next generation sequencing of 502 lifestyle and nutrition related genetic polymorphisms reveals independent loci for low serum 25-hydroxyvitamin d levels among adult respondents of the 2013 Philippine national nutrition survey. *J ASEAN Fed Endocr Soc*. 2021;36(1):56-63.

586. Zupo R, Moroni A, Castellana F, Gasparri C, Catino F, Lampignano L, et al. A machine-learning approach to target clinical and biological features associated with sarcopenia: Findings from Northern and Southern Italian aging populations. *Metabolites*. 2023;13(4):565.

587. Hoy D, Brooks P, Woolf A, Blyth F, March L, Bain C, et al. Assessing risk of bias in prevalence studies: modification of an existing tool and evidence of interrater agreement. *J Clin Epidemiol*. 2012;65:934-9.

*586 included studies plus one reference to the study quality assessment tool that was used

# Supplementary Table 3: Study quality scores ^a^

|  |  | **Item ^b^** | | | | | | | | | | |  |
| --- | --- | --- | --- | --- | --- | --- | --- | --- | --- | --- | --- | --- | --- |
| **Author-year** | **Title** | **1** | **2** | **3** | **4** | **5** | **6** | **7** | **8** | **9** | **10** | **Total** | |
| Abdeen-2015 | Predicted Efficacy of The Palestinian Wheat Flour Fortification Programme: Complementary Analysis of Biochemical and Dietary Data | 0 | 1 | 1 | 0 | 1 | 1 | 1 | 1 | 1 | 1 | 8 | |
| Abidin-2021 | Total Vs. Bioavailable: Determining A Better 25(OH)D Index in Association with Bone Density and Muscle Mass in Postmenopausal Women | 0 | 0 | 0 | 0 | 1 | 1 | 1 | 1 | 1 | 1 | 6 | |
| Abrahao-2021 | Serum 25-Hydroxyvitamin D Concentration and its Association with Glucose Intolerance in an Indigenous Population | 0 | 0 | 0 | 0 | 1 | 1 | 1 | 1 | 1 | 1 | 6 | |
| Abu Shady-2016 | Predictors of Serum 25-Hydroxyvitamin D Concentrations Among a Sample of Egyptian Schoolchildren | 0 | 0 | 0 | 0 | 1 | 1 | 1 | 1 | 1 | 1 | 6 | |
| Acherjya-2020 | Hypovitaminosis D and its Relationship with Diabetes Mellitus Among the Postmenopausal Women in Jashore, Bangladesh | 0 | 0 | 0 | 0 | 1 | 1 | 1 | 1 | 1 | 1 | 6 | |
| Afkhami-Ardekani-2019 | Prevalence and Predictors of Vitamin D Insufficiency in Adult Population of Yazd - The Sun Province in Center of Iran | 0 | 1 | 1 | 0 | 1 | 1 | 1 | 1 | 1 | 1 | 8 | |
| Aggarwal-2021 | Metabolic Bone Profile of Healthy Adult North Indian Population from Chandigarh Urban Bone Epidemiological Study (Cubes) | 0 | 0 | 1 | 0 | 1 | 1 | 1 | 1 | 1 | 1 | 7 | |
| Al Hafidh-2020 | Vitamin D Status and Mitochondrial Function in Children | 0 | 1 | 1 | 1 | 1 | 1 | 1 | 1 | 1 | 1 | 9 | |
| Al-Dabhani-2017 | Prevalence of Vitamin D Deficiency and Association with Metabolic Syndrome in a Qatari Population | 0 | 0 | 0 | 0 | 1 | 1 | 1 | 1 | 1 | 1 | 6 | |
| Al-Daghri-2016 | Calculated Adiposity and Lipid Indices in Healthy Arab Children as Influenced by Vitamin D Status | 0 | 0 | 0 | 0 | 1 | 1 | 1 | 1 | 1 | 1 | 6 | |
| Al-Daghri-2022 | Establishing the Prevalence of Osteomalacia In Arab Adolescents Using Nutritional Biomarkers of Bone Health | 0 | 0 | 0 | 0 | 1 | 1 | 1 | 1 | 1 | 1 | 6 | |
| Al-Eisa-2016 | Correlation Between Vitamin D Levels and Muscle Fatigue Risk Factors Based on Physical Activity in Healthy Older Adults | 0 | 0 | 0 | 0 | 1 | 1 | 1 | 1 | 1 | 1 | 6 | |
| Al-Raddadi-2018 | Prevalence of Lifestyle Practices that Might Affect Bone Health in Relation to Vitamin D Status Among Female Saudi Adolescents | 0 | 1 | 1 | 0 | 1 | 1 | 1 | 1 | 1 | 1 | 8 | |
| Al-Saleh-2015 | Vitamin D Status in Saudi School Children Based on Knowledge | 0 | 1 | 1 | 0 | 1 | 1 | 1 | 1 | 1 | 1 | 8 | |
| Al-Sumaih-2021 | The Use of Finite Mixture Models to Examine the Serum 25(OH)D Levels Among Saudis | 1 | 1 | 1 | 0 | 1 | 1 | 1 | 1 | 1 | 1 | 9 | |
| Al-Taiar-2018 | Vitamin D Status Among Adolescents in Kuwait: A Cross-Sectional Study | 1 | 1 | 1 | 1 | 1 | 1 | 1 | 1 | 1 | 1 | 10 | |
| Alanouti-2022 | Associations Between Serum 25-Hydroxyvitamin D, Body Mass Index and Body Fat Composition Among Emirati Population: Results from the UAE Healthy Future Study | 0 | 0 | 0 | 0 | 1 | 1 | 1 | 1 | 1 | 1 | 6 | |
| Alghadir-2017 | Mechanical Factors and Vitamin D Deficiency in Schoolchildren with Low Back Pain: Biochemical and Cross-Sectional Survey Analysis | 0 | 0 | 1 | 0 | 1 | 1 | 1 | 1 | 1 | 1 | 7 | |
| Ali-2020 | Vitamin D Concentration and Focal Brain Atrophy in Older Adults: A Voxel-Based Morphometric Study | 0 | 0 | 0 | 0 | 1 | 1 | 1 | 1 | 1 | 1 | 6 | |
| Alonso-2019 | Calcidiol Levels and Muscle Function Maintenance, Functional Capacity and Bone Mineral Bone Density in Non-Selected Spanish Population | 0 | 1 | 1 | 0 | 1 | 1 | 1 | 1 | 1 | 1 | 8 | |
| Alyahya-2014 | Risk Factors of Low Vitamin D Status in Adolescent Females in Kuwait: Implications for High Peak Bone Mass Attainment | 0 | 0 | 0 | 0 | 1 | 1 | 1 | 1 | 1 | 1 | 6 | |
| Amaliya-2015 | Java Project on Periodontal Diseases: Periodontal Bone Loss in Relation to Environmental and Systemic Conditions | 0 | 0 | 0 | 0 | 1 | 1 | 1 | 1 | 1 | 1 | 6 | |
| Amanzholkyzy-2018 | Ethnic Manifestations of Gene Polymorphisms of Vitamin D Receptor (VDR) In Adolescents of Western Kazakhstan Region | 0 | 0 | 0 | 0 | 1 | 1 | 1 | 1 | 1 | 1 | 6 | |
| Andersen-2013 | Vitamin D Status In Greenland Is Influenced By Diet And Season: Indicators of Dermal 25-hydroxy Vitamin D Production North of the Arctic Circle | 0 | 1 | 1 | 1 | 1 | 1 | 1 | 1 | 1 | 1 | 9 | |
| Andersen-2013 | Seasonal Changes in Vitamin D Status Among Danish Adolescent Girls and Elderly Women: The Influence of Sun Exposure and Vitamin D Intake | 0 | 1 | 1 | 0 | 1 | 1 | 1 | 1 | 1 | 1 | 8 | |
| Andersen-2018 | Serum 25-hydroxyvitamin D, Calcium and Parathyroid Hormone Levels in Native and European Populations in Greenland | 0 | 1 | 1 | 1 | 1 | 1 | 1 | 1 | 1 | 1 | 9 | |
| Ando-2018 | Serum 25-hydroxyvitamin D Levels Showed Strong Seasonality but Lacked Association with Vitamin D Intake in 3-Year-Old Japanese Children | 0 | 0 | 0 | 0 | 1 | 1 | 1 | 1 | 1 | 1 | 6 | |
| Angeles-Agdeppa-2021 | Vitamin D Status and Usual Nutrient Intake of Filipino Children Aged 6-12 Years in Selected Areas in The Philippines: A 2018 National Nutrition Survey | 0 | 1 | 1 | 0 | 1 | 1 | 1 | 1 | 1 | 1 | 8 | |
| Antczak-Domagala-2019 | Evaluation of The Relationship Between Vitamin D Concentration and Cognitive Performance in Community Dwelling Elderly People | 0 | 0 | 0 | 0 | 1 | 1 | 0 | 0 | 1 | 1 | 4 | |
| Ara-2023 | Factors Affecting the Micronutrient Status of Adolescent Girls Living in Complex Agro-Aquatic Ecological Zones of Bangladesh | 0 | 0 | 0 | 0 | 1 | 1 | 1 | 1 | 1 | 1 | 6 | |
| Arabi-2011 | PTH Level but not 25 (OH) Vitamin D Level Predicts Bone Loss Rates in the Elderly | 0 | 1 | 1 | 0 | 1 | 1 | 1 | 1 | 1 | 1 | 8 | |
| Arabi-2021 | Vitamin D Deficiency in Lebanese Adults: Prevalence and Predictors from a Cross-Sectional Community-Based Study | 0 | 1 | 1 | 0 | 1 | 1 | 1 | 1 | 1 | 1 | 8 | |
| Arazi-2019 | 25-hydroxyvitamin D Levels and Its Relation to Muscle Strength, Maximal Oxygen Consumption, And Body Mass Index in Young and Middle Adulthood Women | 0 | 0 | 0 | 1 | 1 | 1 | 1 | 1 | 1 | 1 | 7 | |
| Asakura-2020 | Vitamin D Status in Japanese Adults: Relationship of Serum 25-hydroxyvitamin D with Simultaneously Measured Dietary Vitamin D Intake and Ultraviolet Ray Exposure | 0 | 0 | 0 | 0 | 1 | 1 | 1 | 1 | 1 | 1 | 6 | |
| Asante-2023 | Vitamin D Status Over Time and Cognitive Function in Norwegian Older Adults: A Prospective Cohort of The Hunt Study | 0 | 0 | 1 | 0 | 1 | 1 | 1 | 1 | 1 | 1 | 7 | |
| Ashraf-2012 | Associations of C-Reactive Protein to Indices of Vascular Health and The Influence of Serum 25(OH)D Status in Healthy Adults | 0 | 0 | 0 | 0 | 1 | 1 | 1 | 1 | 1 | 1 | 6 | |
| Aspelund-2019 | Effect of Genetically Low 25-hydroxyvitamin D on Mortality Risk: Mendelian Randomization Analysis in 3 Large European Cohorts | 1 | 0 | 1 | 0 | 1 | 1 | 1 | 1 | 1 | 1 | 8 | |
| Azmathullah-2016 | Study of Vitamin D Levels in Postmenopausal Women | 0 | 1 | 0 | 1 | 1 | 1 | 1 | 1 | 1 | 1 | 8 | |
| Bacon-2016 | Vitamin D Status of Maori and Non-Maori Octogenarians in New Zealand: A Cohort Study (LiLACS NZ) | 0 | 1 | 1 | 0 | 1 | 1 | 1 | 1 | 1 | 1 | 8 | |
| Baek-2021 | Association of Phthalate Exposure and Airway Dysfunction with Mediation by Serum Periostin | 0 | 0 | 0 | 0 | 1 | 1 | 0 | 0 | 1 | 1 | 4 | |
| Bano-2018 | Influence of Serum 25-hydroxyvitamin D Levels, Fat-Free Mass, and Fat Mass on Bone Density, Geometry and Strength, In Healthy Young and Elderly Adults | 0 | 0 | 0 | 0 | 1 | 1 | 1 | 1 | 0 | 1 | 5 | |
| Bansal-2014 | Serum Parathyroid Hormone and 25-Hydroxyvitamin D Concentrations and Risk of Incident Heart Failure: The Multi-Ethnic Study of Atherosclerosis | 0 | 1 | 0 | 1 | 1 | 1 | 1 | 1 | 1 | 1 | 8 | |
| Basinska-Lewandowska-2021 | Effect of Summer Sunshine Exposure on Vitamin D Status in Young and Middle Age Poles: Is 30 ng/mL Vitamin D Cut-Off Really Suitable for the Polish Population? | 0 | 0 | 0 | 0 | 1 | 1 | 1 | 1 | 1 | 0 | 5 | |
| Batieha-2011 | Vitamin D Status in Jordan: Dress Style and Gender Discrepancies | 1 | 1 | 1 | 0 | 1 | 1 | 1 | 1 | 1 | 1 | 9 | |
| Beer-2020 | Prevalence and Correlates of Vitamin D Deficiency in a Tropical Setting: Results from a Nationally Representative Survey | 1 | 1 | 1 | 0 | 1 | 1 | 1 | 1 | 1 | 1 | 9 | |
| Bennouar-2022 | Reconsidering Vitamin D Optimal Values Based on Parathyroid Hormone Levels in a North Algerian Cohort: Stratification by Gender and Season | 0 | 0 | 0 | 0 | 1 | 1 | 1 | 1 | 1 | 1 | 6 | |
| Beydoun-2021 | Vitamin D Status and its Longitudinal Association with Changes in Patterns of Sleep Among Middle-Aged Urban Adults | 0 | 1 | 1 | 0 | 1 | 1 | 1 | 1 | 1 | 1 | 8 | |
| Bhat-2018 | High Prevalence of Osteoporosis and Morphometric Vertebral Fractures in Indian Males Aged 60 Years and Above: Should Age for Screening be Lowered? | 0 | 1 | 0 | 0 | 1 | 1 | 1 | 1 | 1 | 1 | 7 | |
| Bhatt-2014 | Vitamin D Insufficiency is Associated with Abdominal Obesity in Urban Asian Indians Without Diabetes in North India | 0 | 0 | 0 | 0 | 1 | 1 | 1 | 1 | 1 | 1 | 6 | |
| Bhattacharjee-2019 | A Study to Compare the Metabolic Health (Anthropologic and Biochemical) Between Scheduled Tribe and Non-Scheduled Tribe Population in Underdeveloped Parts in the District of Birbhum, West Bengal: A Population Based Observational Study | 0 | 0 | 0 | 0 | 1 | 0 | 0 | 1 | 1 | 1 | 4 | |
| Biben-2017 | Vitamin D Status and its Impact on Body Composition in Elderly Community-Dwelling Individuals in Bandung And Sumedang, West Java Province, Indonesia | 0 | 1 | 1 | 0 | 1 | 1 | 1 | 1 | 1 | 1 | 8 | |
| Bilinski-2021 | Low Serum 25-hydroxyvitamin D Level Does Not Adversely Affect Bone Turnover in Prepubertal Children | 0 | 0 | 0 | 0 | 1 | 1 | 1 | 1 | 1 | 1 | 6 | |
| Binobead-2019 | Prevalence of Vitamin D Deficiency and the Effect of Anthropometric and Lifestyle Factors on the vitamin D Statuses of Healthy Women Residing in Riyadh | 0 | 0 | 0 | 0 | 1 | 1 | 1 | 1 | 1 | 1 | 6 | |
| Binu-2019 | Bone Health After Fifth Decade in Rural Ambulatory South Indian Postmenopausal Women | 0 | 0 | 0 | 0 | 1 | 1 | 0 | 0 | 1 | 1 | 4 | |
| Blomberg-2017 | Low Maternal Prenatal 25-Hydroxyvitamin D Blood Levels are Associated with Childhood Atopic Dermatitis | 0 | 1 | 1 | 0 | 1 | 1 | 1 | 1 | 1 | 1 | 8 | |
| Bogazzi-2011 | Vitamin D Status May Contribute to Serum Insulin-Like Growth Factor I Concentrations in Healthy Subjects | 0 | 0 | 0 | 0 | 1 | 1 | 1 | 1 | 1 | 1 | 6 | |
| Bojar-2020 | Depression, Metabolic Syndrome, Serum TSH, And Vitamin D Concentrations in Rural and Urban Postmenopausal Women | 0 | 0 | 0 | 0 | 1 | 1 | 1 | 1 | 1 | 1 | 6 | |
| Bonakdaran-2016 | Association Between Serum 25-hydroxyvitamin D Concentrations and Prevalence of Metabolic Syndrome | 0 | 1 | 1 | 0 | 1 | 1 | 1 | 1 | 1 | 0 | 7 | |
| Borissova-2013 | Vitamin D Status in Bulgaria–Winter Data | 0 | 0 | 0 | 0 | 1 | 1 | 1 | 1 | 1 | 1 | 6 | |
| Boucher-Berry-2012 | Vitamin D, Osteocalcin, and Risk for Adiposity as Comorbidities in Middle School Children | 0 | 0 | 0 | 0 | 1 | 1 | 1 | 1 | 1 | 1 | 6 | |
| Bowman-2012 | Nutrient Biomarker Patterns, Cognitive Function, And MRI Measures of Brain Aging | 0 | 1 | 0 | 0 | 1 | 1 | 1 | 1 | 1 | 1 | 7 | |
| Braithwaite-2015 | Vitamin D Binding Protein Genotype is Associated with Plasma 25OHD Concentration in West African Children | 0 | 1 | 1 | 0 | 1 | 1 | 1 | 1 | 1 | 1 | 8 | |
| Buchebner-2019 | Association Between Vitamin D, Frailty, and Progression of Frailty in Community-Dwelling Older Women | 0 | 1 | 1 | 0 | 1 | 1 | 1 | 1 | 1 | 1 | 8 | |
| Buchmann-2021 | Vitamin D Insufficiency is Associated with Metabolic Syndrome Independent of Insulin Resistance and Obesity in Young Adults - The Berlin Aging Study II | 0 | 1 | 1 | 0 | 1 | 1 | 1 | 1 | 1 | 1 | 8 | |
| Cabral-2018 | Relationship Between Dietary Vitamin D and Serum 25-Hydroxyvitamin D Levels in Portuguese Adolescents | 0 | 1 | 1 | 0 | 1 | 1 | 1 | 1 | 1 | 1 | 8 | |
| Cairncross-2016 | The Relationship Between Vitamin D Status and Allergic Diseases in New Zealand Preschool Children | 0 | 0 | 0 | 0 | 1 | 1 | 1 | 1 | 1 | 1 | 6 | |
| Cantio-2022 | Vitamin D Status in Pregnancy and Childhood Associates with Intelligence Quotient at Age 7 Years: An Odense Child Cohort Study | 0 | 1 | 0 | 0 | 1 | 1 | 1 | 1 | 1 | 1 | 7 | |
| Capuano-2021 | Epidemiologic Data of Vitamin D Deficiency and Its Implication in Cardio-Cerebrovascular Risk in A Southern Italian Population | 0 | 1 | 1 | 0 | 1 | 1 | 1 | 1 | 1 | 1 | 8 | |
| Carrelli-2011 | Vitamin D Deficiency Is Associated with Subclinical Carotid Atherosclerosis: The Northern Manhattan Study | 0 | 0 | 0 | 0 | 1 | 1 | 1 | 1 | 1 | 1 | 6 | |
| Carrillo-Vega-2017 | Vitamin D Deficiency in Older Adults and Its Associated Factors: A Cross-Sectional Analysis of The Mexican Health and Aging Study | 1 | 1 | 1 | 0 | 1 | 1 | 1 | 1 | 1 | 1 | 9 | |
| Casey-2019 | Factors Associated with Serum 25-Hydroxyvitamin D Concentrations in Older People in Europe: The EUREYE Study | 0 | 1 | 1 | 0 | 1 | 1 | 1 | 1 | 1 | 1 | 8 | |
| Cashman-2013 | Vitamin D Status of Irish Adults: Findings from the National Adult Nutrition Survey | 1 | 1 | 1 | 0 | 1 | 1 | 1 | 1 | 1 | 1 | 9 | |
| Cashman-2022 | Adequacy of Calcium and Vitamin D Nutritional Status in a Nationally Representative Sample of Irish Teenagers Aged 13-18 Years | 1 | 1 | 1 | 0 | 1 | 1 | 1 | 1 | 1 | 1 | 9 | |
| Chailurkit-2012 | The Association between Vitamin D Status and Type 2 Diabetes in a Thai Population, a Cross-Sectional Study | 1 | 1 | 1 | 1 | 1 | 1 | 1 | 1 | 1 | 1 | 10 | |
| Chan-Na | Not all Elderly People Benefit from Vitamin D Supplementation with Respect to Physical Function: Results from the Osteoporotic Fractures in Men Study, Hong Kong | 0 | 1 | 1 | 0 | 1 | 1 | 1 | 1 | 1 | 1 | 8 | |
| Chaudhry-2018 | Risk Factors for Vitamin A and D Deficiencies Among Children Under-Five in the State of Palestine | 0 | 0 | 0 | 0 | 1 | 1 | 0 | 1 | 1 | 1 | 5 | |
| Checkley-2015 | 25-hydroxy Vitamin D Levels are Associated with Childhood Asthma in a Population-Based Study in Peru | 0 | 1 | 1 | 1 | 1 | 1 | 1 | 1 | 1 | 1 | 9 | |
| Chen-2015 | The Epidemic Investigation of Serum 25-Hydroxy Vitamin D Levels in the Adults in Qujing Area of Yunnan Province in China | 0 | 0 | 0 | 0 | 1 | 1 | 1 | 1 | 1 | 1 | 6 | |
| Chen-2016 | The Associations Between Helicobacter Pylori Infection, Serum Vitamin D, and Metabolic Syndrome: A Community-Based Study | 0 | 0 | 0 | 0 | 1 | 1 | 1 | 1 | 1 | 1 | 6 | |
| Chen-2017 | Vitamin D Status Among the Elderly Chinese Population: A Cross-Sectional Analysis of the 2010-2013 China National Nutrition and Health Survey (CNNHS) | 1 | 1 | 1 | 0 | 1 | 1 | 1 | 1 | 1 | 1 | 9 | |
| Chen-2018 | Associations Between Vitamin D Deficiency, Musculoskeletal Health, and Cardiometabolic Risk Among Community-Living People in Taiwan: Age and Sex-Specific Relationship | 0 | 1 | 1 | 0 | 1 | 1 | 0 | 1 | 1 | 1 | 7 | |
| Chen-2019 | Specific Higher Levels of Serum Uric Acid Might Have a Protective Effect on Bone Mineral Density Within a Chinese Population Over 60 Years Old: A Cross-Sectional Study from Northeast China | 0 | 0 | 0 | 0 | 1 | 1 | 1 | 1 | 1 | 1 | 6 | |
| Cheong-2020 | Nutritional Biomarkers and Associated Factors in Community-Dwelling Older Adults: Findings from The Shield Study | 0 | 0 | 0 | 0 | 1 | 1 | 1 | 1 | 1 | 1 | 6 | |
| Chiang-2017 | 25-hydroxyvitamin D and Blood Pressure: A Plateau Effect in Adults with African Ancestry Living at Different Latitudes | 0 | 1 | 1 | 0 | 1 | 1 | 1 | 1 | 1 | 1 | 8 | |
| Chlebna-Sokol-2019 | Evidence of A Significant Vitamin D Deficiency Among 9-13-Year-Old Polish Children: Results of a Multicentre Study | 0 | 0 | 0 | 0 | 1 | 1 | 1 | 1 | 1 | 1 | 6 | |
| Choi-2014 | Serum 25-Hydroxyvitamin D and Insulin Resistance in Apparently Healthy Adolescents | 0 | 0 | 0 | 1 | 1 | 1 | 1 | 1 | 1 | 1 | 7 | |
| Chua-2020 | Associations of Serum 25-hydroxyvitamin D with Adiposity and At-Risk Lipid Profile Differ for Indigenous (Orang Asli) Male and Female Adults of Peninsular Malaysia | 0 | 1 | 0 | 0 | 1 | 1 | 1 | 1 | 1 | 1 | 7 | |
| Chuang-2016 | Circulating 25-hydroxyvitamin D And Physical Performance in Older Adults: A Nationwide Study in Taiwan | 0 | 1 | 1 | 0 | 1 | 1 | 1 | 1 | 1 | 1 | 8 | |
| Chuc-2019 | Nutritional Status of Children Aged 12 To 36 Months in a Rural District of Hungyen Province, Vietnam | 0 | 1 | 1 | 0 | 1 | 1 | 0 | 0 | 1 | 1 | 6 | |
| Cirillo-2022 | Correlates of Calcidiol Deficiency in Adults-Cross-Sectional, Observational, Population-Based Study | 0 | 1 | 1 | 0 | 1 | 1 | 1 | 1 | 1 | 1 | 8 | |
| Clark-2021 | Vitamin D Concentration and its Association with Parathyroid Hormone in Children and Adolescents | 0 | 0 | 0 | 0 | 1 | 1 | 1 | 1 | 1 | 1 | 6 | |
| Cobayashi-2015 | 25-hydroxyvitamin D3 Levels, BSMI Polymorphism and Insulin Resistance in Brazilian Amazonian Children | 0 | 0 | 0 | 0 | 1 | 1 | 1 | 1 | 1 | 1 | 6 | |
| Conzade-2017 | Prevalence and Predictors of Subclinical Micronutrient Deficiency in German Older Adults: Results from The Population-Based Kora-Age Study | 0 | 0 | 1 | 0 | 1 | 1 | 1 | 1 | 1 | 1 | 7 | |
| Courraud-2020 | Dietary Habits, Metabolic Health and Vitamin D Status in Greenlandic Children | 0 | 1 | 1 | 0 | 1 | 1 | 1 | 1 | 1 | 1 | 8 | |
| Cremers-2011 | Maternal and Child's Vitamin D Supplement Use and Vitamin D Level in Relation to Childhood Lung Function: The Koala Birth Cohort Study | 0 | 0 | 0 | 0 | 1 | 1 | 1 | 1 | 1 | 1 | 6 | |
| Croll-2021 | Associations of Vitamin D Deficiency with MRI Markers of Brain Health in a Community Sample | 0 | 1 | 0 | 1 | 1 | 1 | 1 | 1 | 1 | 1 | 8 | |
| Da-Hye-2017 | Are Serum Vitamin D Levels Associated with Dry Eye Disease? Results from the Study Group for Environmental Eye Disease | 0 | 0 | 0 | 0 | 1 | 1 | 1 | 1 | 1 | 1 | 6 | |
| Dalgard-2011 | Vitamin D Status in Relation to Glucose Metabolism and Type 2 Diabetes in Septuagenarians | 1 | 1 | 1 | 0 | 1 | 1 | 1 | 1 | 1 | 1 | 9 | |
| Das-2022 | Sunlight, Dietary Habits, Genetic Polymorphisms and Vitamin D Deficiency in Urban and Rural Infants of Bangladesh | 0 | 0 | 0 | 0 | 1 | 1 | 1 | 1 | 1 | 1 | 6 | |
| Davarzani-2021 | The Interaction of Aging with Serum 25(Oh)D and 1,25(OH)2 D Status on Muscle Strength | 0 | 0 | 0 | 0 | 1 | 1 | 1 | 1 | 1 | 1 | 6 | |
| De koning-2017 | The Relationship Between Serum 25(OH)D Levels and Anxiety Symptoms in Older Persons: Results from the Longitudinal Aging Study Amsterdam | 0 | 0 | 1 | 0 | 1 | 1 | 1 | 1 | 1 | 1 | 7 | |
| de Menezes-Junior-2023 | Influence of Sunlight on the Association Between 25-hydroxyvitamin D Levels and Sleep Quality in Brazilian Adults: A Population-Based Study | 0 | 0 | 1 | 0 | 1 | 1 | 1 | 1 | 1 | 1 | 7 | |
| de Oliveira-2020 | Prevalence and Factors Associated with Hypovitaminosis D in Adolescents from A Sunny Country: Findings from The ERICA Survey | 0 | 0 | 1 | 0 | 1 | 1 | 1 | 1 | 1 | 1 | 7 | |
| Delshad-2019 | Wintertime Vitamin D Status and its Related Risk Factors Among Children Living in Auckland, New Zealand | 0 | 0 | 0 | 0 | 1 | 1 | 1 | 1 | 1 | 1 | 6 | |
| Deng-2022 | The Interaction Between Vitamin D and Diets on Serum Lipids in Chinese Han Adolescents | 0 | 0 | 0 | 0 | 1 | 1 | 1 | 1 | 1 | 1 | 6 | |
| Denova-gutierrez-2019 | Low Serum Vitamin D Concentrations Are Associated with Insulin Resistance in Mexican Children and Adolescents | 0 | 0 | 0 | 0 | 1 | 1 | 1 | 1 | 1 | 1 | 6 | |
| Dhore-2013 | Vitamin D Status of Apparently Healthy Early Adolescents in Amravati City of Maharashtra, India | 0 | 0 | 0 | 0 | 1 | 1 | 1 | 1 | 1 | 1 | 6 | |
| Diederichsen-2017 | CT-Detected Growth of Coronary Artery Calcification in Asymptomatic Middle-Aged Subjects and Association with 15 Biomarkers | 0 | 1 | 1 | 0 | 1 | 1 | 1 | 1 | 1 | 1 | 8 | |
| Diekmann-2013 | Vitamin D Status and Physical Function in Nursing Home Residents: A 1-Year Observational Study | 0 | 1 | 0 | 0 | 1 | 1 | 1 | 1 | 1 | 1 | 7 | |
| Dimakopoulos-2019 | Association of Serum Vitamin D Status with Dietary Intake and Sun Exposure in Adults | 0 | 1 | 1 | 0 | 1 | 1 | 1 | 1 | 1 | 1 | 8 | |
| Divanoglou-2021 | Association of Vitamin D Receptor Gene Polymorphisms with Serum Vitamin D Levels in a Greek Rural Population (Velestino Study) | 0 | 1 | 1 | 0 | 1 | 1 | 1 | 1 | 1 | 1 | 8 | |
| Djennane-2014 | Vitamin D Status of Schoolchildren in Northern Algeria, Seasonal Variations and Determinants of Vitamin D Deficiency | 0 | 0 | 0 | 0 | 1 | 1 | 1 | 1 | 1 | 1 | 6 | |
| Dogan-Sander-2021 | Inflammation and the Association of Vitamin D and Depressive Symptomatology | 0 | 1 | 1 | 0 | 1 | 1 | 1 | 1 | 1 | 1 | 8 | |
| Dong-2014 | The Micronutrient Status of Children Aged 24-60 Months Living in Rural Disaster Areas One Year After the Wenchuan Earthquake | 0 | 1 | 1 | 0 | 1 | 1 | 1 | 1 | 1 | 1 | 8 | |
| Drali-2021 | Vitamin D Status in Preschool Children in Algeria | 0 | 0 | 0 | 0 | 1 | 1 | 1 | 1 | 1 | 1 | 6 | |
| Duarte-2020 | Prevalence of Vitamin D Deficiency and Its Predictors in the Portuguese Population: A Nationwide Population-Based Study | 1 | 1 | 1 | 0 | 1 | 1 | 1 | 1 | 1 | 1 | 9 | |
| Ebrahimi-2014 | Prevalence of Vitamin D Deficiency Among Iranian Adolescents | 0 | 1 | 1 | 0 | 1 | 1 | 1 | 1 | 1 | 1 | 8 | |
| Egeland-2011 | Food Insecurity and Nutrition Transition Combine to Affect Nutrient Intakes in Canadian Arctic Communities | 0 | 1 | 1 | 0 | 1 | 1 | 1 | 1 | 1 | 1 | 8 | |
| El Badawy-2015 | Vitamin D, Parathormone and Associated Minerals Among Students in Zagazig District, Sharkia Governorate, Egypt | 0 | 1 | 1 | 1 | 1 | 1 | 1 | 1 | 1 | 1 | 9 | |
| El Maataoui-2016 | Vitamin D Status in Healthy Moroccan Men and Women Aged 50 Years and Older: A Cross-Sectional Study | 0 | 0 | 0 | 0 | 1 | 1 | 1 | 1 | 1 | 1 | 6 | |
| El-Khateeb-2019 | Vitamin D Deficiency and Associated Factors in Jordan | 1 | 1 | 1 | 0 | 1 | 1 | 1 | 1 | 1 | 1 | 9 | |
| Ellul-2020 | Plasma Metabolomic Profiles Associated with Infant Food Allergy with Further Consideration of Other Early Life Factors | 0 | 1 | 0 | 0 | 1 | 1 | 1 | 1 | 1 | 1 | 7 | |
| Emmerson-2018 | Vitamin D Status of White Pregnant Women and Infants at Birth and 4 Months in North West England: A Cohort Study | 0 | 0 | 0 | 0 | 1 | 1 | 1 | 1 | 1 | 1 | 6 | |
| Erasmus-2022 | Vitamin D, Vitamin D-Binding Proteins, And VDR Polymorphisms in Individuals with Hyperglycaemia | 0 | 0 | 1 | 0 | 1 | 0 | 1 | 1 | 1 | 1 | 6 | |
| Erdonmez-2011 | No Relationship Between Vitamin D Status and Insulin Resistance in A Group of High School Students | 0 | 0 | 0 | 1 | 1 | 1 | 1 | 1 | 1 | 1 | 7 | |
| Ewendt-2023 | Association Between Vitamin D Status and Eryptosis-Results from the German National Cohort Study | 0 | 0 | 1 | 0 | 1 | 1 | 1 | 1 | 1 | 1 | 7 | |
| Fabian-2012 | Vitamin Status in Elderly People in Relation to the Use of Nutritional Supplements | 0 | 0 | 0 | 0 | 1 | 1 | 1 | 1 | 1 | 1 | 6 | |
| Farber-2021 | Evaluation of Hemorheological Parameters as Biomarkers of Calcium Metabolism and Insulin Resistance in Postmenopausal Women | 0 | 0 | 0 | 0 | 1 | 1 | 1 | 1 | 1 | 1 | 6 | |
| Fassula-2021 | Moderator Role of Vitamin D Concentrations on The Association Between Metabolic Syndrome and C-Reactive Protein Among Adults | 0 | 1 | 1 | 0 | 1 | 1 | 1 | 1 | 1 | 1 | 8 | |
| Feehan-2022 | Vitamin D and Bone Health of Older Adults Within Care Homes: An Observational Study | 0 | 1 | 0 | 0 | 1 | 1 | 1 | 1 | 1 | 1 | 7 | |
| Flores Ruelas-2020 | Vitamin D Status and its Relation to Insulin Resistance in a Mexican Pediatric Population | 0 | 0 | 0 | 0 | 1 | 1 | 1 | 1 | 0 | 1 | 5 | |
| Flores-2021 | Vitamin D Status in Mexican Children 1 To 11 Years of Age: An Update from the ENSANUT 2018-19 | 1 | 1 | 1 | 1 | 1 | 1 | 1 | 1 | 1 | 1 | 10 | |
| Forney-2017 | Emerging Relationships Between Vitamin D Status, Physical Activity Habits, and Immune Indices in College-Aged Females | 0 | 0 | 0 | 0 | 1 | 1 | 1 | 1 | 1 | 1 | 6 | |
| Fox-2023 | 25-hydroxyvitamin D Level is Associated with Greater Grip Strength Across Adult Life Span: A Population-Based Cohort Study | 0 | 0 | 1 | 0 | 1 | 1 | 1 | 1 | 1 | 1 | 7 | |
| Galeazzi-2023 | Vitamin D Status in Healthy Italian School-Age Children: A Single-Center Cross-Sectional Study | 0 | 1 | 0 | 0 | 1 | 1 | 1 | 1 | 1 | 1 | 7 | |
| Ganie-2022 | Vitamin D Status Among Kashmiri Tribal Population: A Cross-Sectional Community-Based Study | 0 | 1 | 1 | 0 | 1 | 1 | 1 | 1 | 1 | 1 | 8 | |
| Gannage-Yared-2018 | Relationship Between 25 Hydroxyvitamin D and Lipid Profile in Lebanese School Children | 1 | 1 | 1 | 0 | 1 | 1 | 1 | 1 | 1 | 1 | 9 | |
| Gao-2017 | The Levels of Bone Turnover Markers 25(OH)D and PTH And Their Relationship with Bone Mineral Density in Postmenopausal Women in A Suburban District in China | 0 | 0 | 1 | 0 | 1 | 1 | 1 | 1 | 1 | 1 | 7 | |
| Garcia-Dorta-2021 | Baseline Levels of Vitamin D in A Healthy Population from A Region with High Solar Irradiation | 0 | 1 | 1 | 0 | 1 | 1 | 1 | 1 | 1 | 1 | 8 | |
| Garg-2014 | The Relationship Between Serum 25-hydroxy Vitamin D, Parathormone And Bone Mineral Density in Indian Population | 0 | 0 | 0 | 0 | 1 | 1 | 1 | 1 | 1 | 1 | 6 | |
| Garg-2018 | Sun Exposure and Vitamin D in Rural India: A Cross-Sectional Study | 0 | 1 | 1 | 1 | 1 | 1 | 1 | 1 | 1 | 1 | 9 | |
| Ge-2017 | The Association Between Serum 25-hydroxyvitamin D3 Concentration and Serum Lipids in The Rural Population of China | 0 | 0 | 0 | 0 | 1 | 1 | 1 | 1 | 1 | 1 | 6 | |
| Gebreegziabher-2013 | Vitamin D Insufficiency in a Sunshine-Sufficient Area: Southern Ethiopia | 0 | 0 | 0 | 0 | 1 | 1 | 1 | 1 | 1 | 1 | 6 | |
| Ghobadi-2019 | Association of Vitamin D Status and Metabolic Syndrome Components in Iranian Children | 0 | 1 | 1 | 0 | 1 | 1 | 1 | 1 | 1 | 1 | 8 | |
| Giallauria-2012 | Arterial Stiffness and Vitamin D Levels: The Baltimore Longitudinal Study of Aging | 0 | 1 | 0 | 1 | 1 | 0 | 1 | 1 | 1 | 1 | 7 | |
| Gill-2014 | Vitamin D Levels in an Australian Population | 0 | 1 | 1 | 0 | 1 | 1 | 1 | 1 | 1 | 1 | 8 | |
| Gonzalez-Molero-2011 | Vitamin D Deficiency in Spain: A Population-Based Cohort Study | 0 | 0 | 0 | 0 | 1 | 1 | 1 | 1 | 1 | 1 | 6 | |
| Gonzalez-Molero-2012 | Vitamin D and Incidence of Diabetes: A Prospective Cohort Study | 0 | 0 | 1 | 0 | 1 | 1 | 1 | 1 | 1 | 1 | 7 | |
| Goodwill-2018 | Vitamin D Status is Associated with Executive Function a Decade Later: Data from the Women's Healthy Ageing Project | 0 | 1 | 1 | 0 | 1 | 1 | 1 | 1 | 1 | 1 | 8 | |
| Goswami-2021 | Hypovitaminosis D, Dyslipidemia, and Thyroid Dysfunction Among Adolescents and Their Associations with Blood Pressure in a Northeastern City of India | 0 | 1 | 1 | 0 | 1 | 0 | 0 | 0 | 1 | 0 | 4 | |
| Grant-2009 | Vitamin D Deficiency in Early Childhood: Prevalent in The Sunny South Pacific | 0 | 1 | 1 | 0 | 1 | 1 | 1 | 1 | 1 | 1 | 8 | |
| Greene-Finestone-2011 | 25-hydroxyvitamin D in Canadian Adults: Biological, Environmental, and Behavioral Correlates | 1 | 1 | 1 | 0 | 1 | 1 | 1 | 1 | 1 | 1 | 9 | |
| Grineva-2013 | Vitamin D Deficiency is A Risk Factor for Obesity and Diabetes Type 2 in Women at Late Reproductive Age | 0 | 0 | 0 | 0 | 1 | 1 | 1 | 1 | 1 | 1 | 6 | |
| Guan-2020 | Correlation of Serum Vitamin D with Lipid Profiles in Middle-Aged and Elderly Chinese Individuals | 0 | 1 | 1 | 0 | 1 | 1 | 1 | 1 | 1 | 1 | 8 | |
| Gudmundsdottir-2020 | Serum 25-hydroxyvitamin D Concentrations in 16-Year-Old Icelandic Adolescent and its Association with Bone Mineral Density | 0 | 0 | 0 | 0 | 1 | 1 | 1 | 1 | 1 | 1 | 6 | |
| Ha-2013 | Serum Vitamin D, Physical Activity, and Metabolic Risk Factors in Korean Children | 0 | 0 | 0 | 0 | 1 | 1 | 1 | 1 | 1 | 1 | 6 | |
| Habibesadat-2014 | Prevalence of Vitamin D Deficiency and its Related Factors in Children and Adolescents Living in North Khorasan, Iran | 0 | 1 | 1 | 0 | 1 | 1 | 1 | 1 | 1 | 1 | 8 | |
| Hacker-Thompson-2012 | Validation of a Dietary Vitamin D Questionnaire Using Multiple Diet Records and the Block 98 Health Habits and History Questionnaire in Healthy Postmenopausal Women in Northern California | 0 | 0 | 0 | 0 | 1 | 1 | 1 | 1 | 1 | 1 | 6 | |
| Hanks-2012 | Calcium Homeostasis may Influence Resting Energy Expenditure with Effects Most Apparent in Early Pubertal Girls | 0 | 0 | 0 | 0 | 1 | 1 | 1 | 1 | 1 | 1 | 6 | |
| Hansen-2015 | Association of 25-hydroxyvitamin D Status and Genetic Variation in the Vitamin D Metabolic Pathway with FEV1 In the Framingham Heart Study | 0 | 1 | 0 | 1 | 1 | 1 | 1 | 1 | 1 | 1 | 8 | |
| Hao-2014 | Additional Role of Serum 25-hydroxyvitamin D-3 Levels in Atherosclerosis in Chinese Middle-Aged and Elderly Men | 0 | 1 | 0 | 1 | 1 | 0 | 1 | 1 | 1 | 1 | 7 | |
| Hasan-2017 | Association of Vitamin D Receptor Gene Polymorphisms with Metabolic Syndrome and its Components Among Adult Arabs From the United Arab Emirates | 0 | 0 | 0 | 0 | 1 | 1 | 1 | 1 | 1 | 1 | 6 | |
| Haslam-2014 | Vitamin D Status is Associated with Grip Strength in Centenarians | 0 | 1 | 1 | 0 | 1 | 1 | 1 | 1 | 1 | 1 | 8 | |
| Hata-2023 | Osteoporosis and Sarcopenia are Associated With Each Other and Reduced IGF1 Levels Are A Risk for Both Diseases in the Very Old Elderly | 0 | 1 | 0 | 0 | 1 | 0 | 1 | 1 | 1 | 1 | 6 | |
| Hauksson-2016 | Vitamin D Status of Icelandic Children and Its Influence on Bone Accrual | 0 | 1 | 1 | 0 | 1 | 1 | 1 | 1 | 1 | 1 | 8 | |
| Hekimoglu-2023 | Comparison of Cord Blood and 6-Month-Old Vitamin D Levels of Healthy Term Infants Supplemented With 400 IU/Day Dose of Vitamin D | 0 | 0 | 0 | 0 | 1 | 1 | 1 | 1 | 1 | 1 | 6 | |
| Hekimsoy-2010 | Vitamin D Status Among Adults in the Aegean Region of Turkey | 0 | 1 | 1 | 0 | 1 | 1 | 1 | 1 | 1 | 1 | 8 | |
| Henning-2023 | Patterns of Dietary Blood Markers are Related to Frailty Status in The Frailomic Validation Phase | 0 | 1 | 1 | 0 | 1 | 1 | 1 | 0 | 0 | 1 | 6 | |
| Herrador-2014 | Micronutrient Deficiencies and Related Factors in School-Aged Children in Ethiopia: A Cross-Sectional Study in Libo Kemkem and Fogera Districts, Amhara Regional State | 0 | 1 | 1 | 0 | 1 | 1 | 1 | 1 | 1 | 1 | 8 | |
| Hien-2012 | Vitamin D Status of Pregnant and Non-Pregnant Women of Reproductive Age Living in Hanoi City and The Hai Duong Province of Vietnam | 0 | 1 | 1 | 0 | 1 | 1 | 1 | 1 | 1 | 1 | 8 | |
| Hill-2016 | Serum 25-Hydroxyvitamin D Concentration and its Determinants in the Very Old: The Newcastle 85+ Study | 1 | 1 | 0 | 0 | 1 | 1 | 1 | 1 | 1 | 1 | 8 | |
| Hirani-2013 | Vitamin D Status Among Older Community Dwelling Men Living in a Sunny Country and Associations with Lifestyle Factors: The Concord Health and Ageing in Men Project, Sydney, Australia | 0 | 1 | 1 | 0 | 1 | 1 | 1 | 1 | 1 | 1 | 8 | |
| Hirschler-2014 | Association Between Vitamin D and Apo B Concentrations in Argentinean Indian Children | 0 | 1 | 1 | 0 | 1 | 1 | 1 | 1 | 1 | 1 | 8 | |
| Hirschler-2019 | Vitamin D Levels and Cardiometabolic Markers in Indigenous Argentinean Children Living at Different Altitudes | 0 | 1 | 1 | 1 | 1 | 1 | 1 | 1 | 1 | 1 | 9 | |
| Hoevenaar-Blom-2019 | Prevalence and Determinants of Vitamin D Deficiency in Infants and Toddlers in The Netherlands: A Pilot Study | 0 | 0 | 0 | 0 | 1 | 1 | 1 | 1 | 1 | 1 | 6 | |
| Hoge-2015 | Vitamin D Deficiency is Common Among Adults in Wallonia (Belgium, 51 Degrees 30 ' North): Findings from The Nutrition, Environment and Cardio-Vascular Health Study | 0 | 1 | 1 | 0 | 1 | 1 | 1 | 1 | 1 | 1 | 8 | |
| Horton-French-2021 | Prevalence and Predictors of Vitamin D Deficiency in a Nationally Representative Sample of Australian Adolescents and Young Adults | 1 | 1 | 1 | 0 | 1 | 1 | 1 | 1 | 1 | 1 | 9 | |
| Houghton-2019 | Micronutrient Status Differs Among Maasai And Kamba Preschoolers in a Supplementary Feeding Programme in Kenya | 0 | 0 | 0 | 0 | 1 | 1 | 1 | 1 | 1 | 1 | 6 | |
| Hribar-2020 | Nutrihealth Study: Seasonal Variation in Vitamin D Status Among the Slovenian Adult and Elderly Population | 0 | 1 | 1 | 0 | 1 | 1 | 1 | 1 | 1 | 1 | 8 | |
| Hu-2021 | Threshold for Relationship Between Vitamin D and Parathyroid Hormone in Chinese Women of Childbearing Age | 1 | 1 | 1 | 0 | 1 | 1 | 1 | 1 | 1 | 1 | 9 | |
| Hu-2022 | The Modification of Individual Factors on Association Between Serum 25(OH)D and Incident Type 2 Diabetes: Results from a Prospective Cohort Study | 0 | 0 | 1 | 0 | 1 | 1 | 1 | 1 | 1 | 1 | 7 | |
| Hu-2022 | Vitamin D Status for Chinese Children and Adolescents in CNNHS 2016-2017 | 0 | 1 | 1 | 1 | 1 | 1 | 1 | 1 | 1 | 1 | 9 | |
| Huang-2013 | Lipoprotein Lipase Links Vitamin D, Insulin Resistance, And Type 2 Diabetes: A Cross-Sectional Epidemiological Study | 1 | 1 | 1 | 0 | 1 | 1 | 1 | 1 | 1 | 1 | 9 | |
| Huang-2019 | Vitamin D and Cause-Specific Vascular Disease and Mortality: A Mendelian Randomisation Study Involving 99,012 Chinese and 106,911 European Adults | 0 | 1 | 0 | 1 | 1 | 0 | 1 | 1 | 1 | 1 | 7 | |
| Huang-2022 | Correlation Analysis Between Vitamin A, D, And E Status with Altitude, Seasonal Variation, And Other Factors, Among Children Aged 0-6 Years in a Chinese Population Living in the Tibetan Plateau of Ganzi Prefecture | 0 | 0 | 0 | 0 | 1 | 1 | 1 | 1 | 1 | 1 | 6 | |
| Hurskainen-2012 | Association of Serum 25-hydroxyvitamin D with Type 2 Diabetes and Markers of Insulin Resistance in a General Older Population in Finland | 0 | 1 | 1 | 1 | 1 | 1 | 1 | 1 | 1 | 1 | 9 | |
| Husemoen-2016 | Serum 25-hydroxyvitamin D And Self-Reported Mental Health Status in Adult Danes | 0 | 0 | 1 | 0 | 1 | 1 | 1 | 1 | 1 | 1 | 7 | |
| Hussain-2020 | Levels of Vitamin D and Vitamin D Pathway Gene Polymorphisms in Adults: Results Based on Rural Agriculture Workers in Punjab Province of Pakistan | 0 | 1 | 0 | 0 | 1 | 0 | 1 | 1 | 0 | 1 | 5 | |
| Huta-Osiecka-2017 | Serum 25-hydroxyvitamin D Concentrations and Selected Diet Components in Postmenopausal Women | 0 | 0 | 0 | 0 | 1 | 1 | 1 | 1 | 1 | 1 | 6 | |
| Hutchings-2022 | Vitamin D Status in Armenian Women: A Stratified Cross-Sectional Cluster Analysis | 1 | 1 | 1 | 0 | 1 | 1 | 1 | 1 | 1 | 1 | 9 | |
| Ikonen-2021 | The Determinants and Longitudinal Changes in Vitamin D Status in Middle-Age: A Northern Finland Birth Cohort 1966 Study | 0 | 1 | 1 | 1 | 1 | 1 | 1 | 1 | 1 | 1 | 9 | |
| Imran-2019 | Low 25-hydroxyvitamin D Level Increases Carotid Intima-Media Thickness in Elderly Women | 0 | 0 | 1 | 0 | 1 | 1 | 1 | 1 | 1 | 1 | 7 | |
| Jain-2011 | Vitamin D Deficiency in Healthy Breastfed Term Infants at 3 Months & Their Mothers in India: Seasonal Variation & Determinants | 0 | 0 | 0 | 0 | 1 | 1 | 1 | 1 | 1 | 1 | 6 | |
| Jakab-2017 | Standardizing 25-hydroxyvitamin D Data from The Hunmen Cohort | 0 | 0 | 0 | 0 | 1 | 1 | 1 | 1 | 1 | 1 | 6 | |
| Jamka-2021 | Assessment of The Effect of Estradiol on Biochemical Bone Turnover Markers Among Postmenopausal Women | 0 | 0 | 0 | 0 | 1 | 1 | 1 | 1 | 1 | 1 | 6 | |
| Jang-2013 | Association Between Serum Vitamin D and Metabolic Risk Factors in Korean Schoolgirls | 0 | 0 | 0 | 0 | 1 | 1 | 1 | 1 | 1 | 1 | 6 | |
| Janssen-2013 | Vitamin D and Muscle Function: Is There a Threshold in the Relation? | 0 | 0 | 0 | 0 | 1 | 1 | 1 | 1 | 0 | 1 | 5 | |
| Jayatissa-2019 | Vitamin D Deficiency Among Children Aged 10-18 Years in Sri Lanka | 1 | 1 | 1 | 0 | 1 | 1 | 1 | 1 | 1 | 1 | 9 | |
| Jeddi-2013 | Normative Data and Percentile Curves of Bone Mineral Density in Healthy Iranian Children Aged 9-18 Years | 0 | 1 | 1 | 1 | 1 | 1 | 1 | 1 | 1 | 1 | 9 | |
| Jeenduang-2018 | Vitamin D Status and Its Associated Factors in Rural Subjects in Nakhon Si Thammarat Province, Southern Thailand | 0 | 0 | 0 | 0 | 1 | 1 | 1 | 1 | 1 | 1 | 6 | |
| Jelmila-2020 | Correlation Between 25-hydroxyvitamin D Serum Levels with Telomere Length in Premenopausal Minangkabau Ethnicity Women | 0 | 1 | 1 | 0 | 1 | 1 | 1 | 1 | 1 | 1 | 8 | |
| Jiajue-2021 | Associations Between Osteocalcin, Calciotropic Hormones, and Energy Metabolism in a Cohort of Chinese Postmenopausal Women: Peking Vertebral Fracture Study | 0 | 1 | 1 | 0 | 1 | 1 | 1 | 1 | 1 | 1 | 8 | |
| Jiang-2020 | An Epidemiology Survey of Vitamin D Deficiency and Its Influencing Factors | 0 | 1 | 0 | 0 | 1 | 1 | 1 | 1 | 1 | 1 | 7 | |
| Jones-2020 | Independent and Interactive Influences of Environmental UVR, Vitamin D Levels, And Folate Variant MTHFD-Rs2236225 On Homocysteine Levels | 0 | 0 | 0 | 0 | 1 | 1 | 1 | 1 | 1 | 1 | 6 | |
| Joukar-2020 | Association of Serum Levels of Vitamin D with Blood Pressure Status in Northern Iranian Population: The Persian Guilan Cohort Study (PGCS) | 0 | 1 | 1 | 0 | 1 | 1 | 1 | 1 | 1 | 1 | 8 | |
| Kandhro-2019 | Study of Vitamin D Deficiency and Contributing Factors in the Population of Hyderabad, Pakistan | 0 | 0 | 1 | 0 | 1 | 1 | 1 | 1 | 1 | 1 | 7 | |
| Kaneva-2022 | Association of Serum 25-hydroxyvitamin D with Metabolic Disturbances in Adolescents | 0 | 0 | 0 | 0 | 1 | 1 | 1 | 1 | 0 | 1 | 5 | |
| Kapil-2017 | Prevalence of Vitamin D Deficiency and Associated Risk Factors Among Children Residing at High Altitude in Shimla District, Himachal Pradesh, India | 0 | 1 | 1 | 0 | 1 | 1 | 1 | 1 | 1 | 1 | 8 | |
| Karaguzel-2014 | Seasonal Vitamin D Status of Healthy Schoolchildren and Predictors of Low Vitamin D Status | 0 | 0 | 0 | 0 | 1 | 1 | 1 | 1 | 1 | 1 | 6 | |
| Karhapaa-2012 | Glomerular Filtration Rate and Parathyroid Hormone are Associated with 1,25-Dihydroxyvitamin D in Men Without Chronic Kidney Disease | 0 | 1 | 1 | 0 | 1 | 1 | 0 | 0 | 1 | 1 | 6 | |
| Karimi-2019 | Association Between Serum Uric Acid and Bone Health in Adolescents | 0 | 0 | 1 | 0 | 1 | 1 | 1 | 1 | 1 | 1 | 7 | |
| Karin-2018 | Vitamin D Status and Analysis of Specific Correlates in Preschool Children: A Cross-Sectional Study in Southern Croatia | 0 | 1 | 0 | 1 | 1 | 1 | 1 | 1 | 1 | 1 | 8 | |
| Karuppusami-2020 | Association of Serum 25-Hydroxy Vitamin D with Total and Regional Adiposity and Cardiometabolic Traits | 0 | 1 | 1 | 0 | 1 | 1 | 1 | 1 | 1 | 1 | 8 | |
| Kassem-2020 | Sex Differences in Vitamin D Deficiency and Anthropometric Measurements in School-Age Children from Rural Areas in Israel | 0 | 0 | 0 | 0 | 1 | 1 | 1 | 1 | 1 | 1 | 6 | |
| Kaykhaei-2011 | High Prevalence of Vitamin D Deficiency in Zahedan, Southeast Iran | 0 | 0 | 0 | 0 | 1 | 1 | 1 | 1 | 1 | 1 | 6 | |
| Ke-2015 | Vitamin D and Parathyroid Hormone Status in A Representative Population Living in Macau, China | 0 | 1 | 1 | 0 | 1 | 1 | 1 | 1 | 1 | 1 | 8 | |
| Ke-2018 | Vitamin D Levels in Childhood and Adolescence and Cardiovascular Risk Factors in a Cohort of Healthy Australian Children | 0 | 0 | 0 | 0 | 1 | 1 | 1 | 1 | 1 | 1 | 6 | |
| Kensara-2019 | The Combined Effect of Vitamin D Deficiency and Hyperparathyroidism on Postural Stability Among Healthy Adult Males | 0 | 0 | 0 | 0 | 1 | 1 | 1 | 1 | 1 | 1 | 6 | |
| Kensarah-2015 | Hypovitaminosis D in Healthy Toddlers and Preschool Children from Western Saudi Arabia | 0 | 1 | 1 | 0 | 1 | 1 | 1 | 1 | 1 | 1 | 8 | |
| Khadilkar-2022 | Vitamin D Status and Determinants in Indian Children and Adolescents: A Multicentre Study | 0 | 1 | 1 | 0 | 1 | 1 | 1 | 1 | 1 | 1 | 8 | |
| Khan-2012 | Prevalence of Vitamin D Deficiency and its Correlates: Results of a Community-Based Study Conducted in Karachi, Pakistan | 0 | 1 | 1 | 0 | 1 | 1 | 1 | 1 | 1 | 1 | 8 | |
| Khan-2019 | Are Serum Leptin Levels Predicted by Lipoproteins, Vitamin D and Body Composition? | 0 | 0 | 0 | 0 | 1 | 1 | 1 | 1 | 1 | 1 | 6 | |
| Khan-2020 | Prevalence of Vitamin D Insufficiency in School Going Children of Eastern Nepal: A Cross-Sectional Study | 0 | 0 | 1 | 0 | 1 | 1 | 1 | 1 | 1 | 1 | 7 | |
| Khayyatzadeh-2018 | Serum Transaminase Concentrations and the Presence of Irritable Bowel Syndrome Are Associated with Serum 25-hydroxy Vitamin D Concentrations in Adolescent Girls Who Are Overweight and Obese | 0 | 1 | 1 | 1 | 1 | 1 | 1 | 1 | 1 | 1 | 9 | |
| Khosravi-boroujeni-2017 | Prevalence and Trends of Vitamin D Deficiency Among Iranian Adults: A Longitudinal Study from 2001-2013 | 0 | 0 | 0 | 0 | 1 | 1 | 1 | 1 | 1 | 1 | 6 | |
| Khwanchuea-2022 | Associations Between Body Composition, Leptin, and Vitamin D Varied by the Body Fat Percentage in Adolescents | 0 | 0 | 0 | 0 | 1 | 0 | 1 | 1 | 1 | 1 | 5 | |
| Kilinc-2019 | Evaluation of Vitamin D Status and its Correlation with Gonadal Function in Children at Mini-Puberty | 0 | 0 | 0 | 0 | 1 | 1 | 1 | 1 | 1 | 1 | 6 | |
| Kim-2012 | Relationship Between Vitamin D, Parathyroid Hormone, and Bone Mineral Density in Elderly Koreans | 0 | 1 | 1 | 0 | 1 | 1 | 1 | 1 | 1 | 1 | 8 | |
| Kim-2013 | Relationships Between Sarcopenic Obesity and Insulin Resistance, Inflammation, and Vitamin D Status: The Korean Sarcopenic Obesity Study | 0 | 0 | 0 | 0 | 1 | 1 | 1 | 1 | 1 | 1 | 6 | |
| Kim-2020 | Association of Serum Vitamin D with Frailty in Older Korean Adults | 1 | 1 | 1 | 1 | 1 | 1 | 1 | 1 | 1 | 1 | 10 | |
| Kimlin-2014 | The Contributions of Solar Ultraviolet Radiation Exposure and Other Determinants to Serum 25-Hydroxyvitamin D Concentrations in Australian Adults: The AusD Study | 1 | 1 | 1 | 0 | 1 | 1 | 1 | 1 | 1 | 1 | 9 | |
| Klenk-2013 | Seasonality of Vitamin D Status in Older People in Southern Germany: Implications for Assessment | 0 | 0 | 1 | 0 | 1 | 1 | 1 | 1 | 1 | 1 | 7 | |
| Koda-2023 | Associations of Plasma 25-hydroxy Vitamin D and Dietary Vitamin D Intake with Insulin Resistance in Healthy Japanese Women | 0 | 0 | 0 | 0 | 1 | 1 | 1 | 1 | 0 | 1 | 5 | |
| Kouda-2013 | Vitamin D Status and Body Fat Measured by Dual-Energy X-Ray Absorptiometry in A General Population of Japanese Children | 0 | 0 | 0 | 0 | 1 | 1 | 1 | 1 | 1 | 1 | 6 | |
| Kruavit-2012 | Prevalence of Vitamin D Insufficiency and Low Bone Mineral Density in Elderly Thai Nursing Home Residents | 0 | 0 | 0 | 0 | 1 | 1 | 1 | 1 | 1 | 1 | 6 | |
| Kull-2012 | Vitamin D as a Possible Independent Predictor of Bone Mineral Density in Estonian Adults: A Cross-Sectional Population-Based Study | 0 | 1 | 1 | 0 | 1 | 1 | 1 | 1 | 0 | 1 | 7 | |
| Kutlay-2014 | The Association of Serum Vitamin D Levels with Several Cardiometabolic Risk and Aortic Pulse Wave Velocity in Elderly Persons | 0 | 1 | 1 | 0 | 1 | 1 | 1 | 1 | 1 | 1 | 8 | |
| Kuwabara-2020 | Vitamin D Deficiency as the Risk of Respiratory Tract Infections in the Institutionalized Elderly: A Prospective 1-Year Cohort Study | 0 | 0 | 0 | 0 | 1 | 1 | 1 | 1 | 1 | 1 | 6 | |
| Laaksi-2023 | Associations of Serum 25(Oh)D Levels with Physical Performance and Anabolic Hormones in Young Men | 0 | 0 | 0 | 0 | 1 | 1 | 1 | 1 | 0 | 1 | 5 | |
| Laillou-2013 | Hypovitaminosis D and Mild Hypocalcaemia are Highly Prevalent Among Young Vietnamese Children and Women and Related to Low Dietary Intake | 1 | 0 | 1 | 0 | 1 | 1 | 1 | 1 | 1 | 1 | 8 | |
| Laird-2018 | The Prevalence of Vitamin D Deficiency and The Determinants Of 25(OH)D Concentration in Older Irish Adults: Data from The Irish Longitudinal Study on Ageing (TILDA) | 1 | 1 | 1 | 1 | 1 | 1 | 1 | 1 | 1 | 1 | 10 | |
| Lam-2016 | Serum 25-hydroxyvitamin D is Associated with Reduced Verbal Episodic Memory in Healthy, Middle-Aged and Older Adults | 0 | 0 | 0 | 0 | 1 | 1 | 1 | 1 | 1 | 1 | 6 | |
| Larcombe-2012 | Vitamin D in a Northern Canadian First Nation Population: Dietary Intake, Serum Concentrations and Functional Gene Polymorphisms | 0 | 0 | 0 | 0 | 1 | 1 | 1 | 1 | 1 | 1 | 6 | |
| Larijani-2016 | Vitamin D Deficiency, Bone Turnover Markers and Causative Factors Among Adolescents: A Cross-Sectional Study | 0 | 1 | 1 | 0 | 1 | 1 | 1 | 1 | 1 | 1 | 8 | |
| Larsen-2022 | Associations of Serum 25-Hydroxyvitamin D and Subjective Sleep Measures in an Arctic Population: Insights from the Population-Based Tromso Study | 0 | 0 | 1 | 0 | 1 | 1 | 1 | 1 | 1 | 1 | 7 | |
| Lategan-2016 | Vitamin D Status, Hypertension and Body Mass Index in an Urban Black Community in Mangaung, South Africa | 0 | 0 | 1 | 0 | 1 | 1 | 1 | 1 | 1 | 1 | 7 | |
| Lee-2011 | Lower Vitamin D Levels are Associated with Depression Among Community-Dwelling European Men | 0 | 1 | 1 | 0 | 1 | 1 | 1 | 1 | 1 | 1 | 8 | |
| Lee-2017 | Association of Metabolic Syndrome and 25-hydroxyvitamin D with Cognitive Impairment Among Elderly Koreans | 0 | 0 | 0 | 0 | 1 | 1 | 1 | 1 | 1 | 1 | 6 | |
| Lee-2020 | Association of Nonalcoholic Fatty Liver Disease with Serum Vitamin D Levels in Combination of Physical Fitness in Korean Older Adults | 0 | 0 | 0 | 0 | 1 | 1 | 1 | 1 | 1 | 1 | 6 | |
| Lee-2021 | Independent and Interactive Associations of Season, Dietary Vitamin D, And Vitamin D-Related Genetic Variants with Serum 25(OH)D in Korean Adults Aged 40 Years or Older | 0 | 0 | 0 | 0 | 1 | 1 | 1 | 1 | 1 | 1 | 6 | |
| Leiros-2022 | Cognitive Status and Nutritional Markers in a Sample of Institutionalized Elderly People | 0 | 0 | 0 | 0 | 1 | 1 | 1 | 1 | 1 | 1 | 6 | |
| Leitao-2018 | No Evidence for Lower Levels of Serum Vitamin D in The Presence of Hepatic Steatosis. A Study on the Portuguese General Population | 1 | 0 | 1 | 0 | 1 | 1 | 1 | 1 | 1 | 1 | 8 | |
| Li-2016 | Establishment of a Normal Reference Value of Parathyroid Hormone in a Large Healthy Chinese Population and Evaluation of its Relation to Bone Turnover and Bone Mineral Density | 0 | 0 | 1 | 0 | 1 | 1 | 1 | 1 | 1 | 1 | 7 | |
| Li-2017 | Bioavailable 25(OH)D but Not Total 25(OH)D Is an Independent Determinant for Bone Mineral Density in Chinese Postmenopausal Women | 0 | 1 | 0 | 1 | 1 | 1 | 1 | 1 | 1 | 1 | 8 | |
| Li-2018 | Higher Serum 25(OH)D Level Is Associated with Decreased Risk of Impairment of Glucose Homeostasis: Data from Southwest China | 0 | 1 | 1 | 0 | 1 | 1 | 1 | 1 | 1 | 1 | 8 | |
| Li-2018 | Serum 25-hydroxyvitamin D and Bone Mineral Density Among Children and Adolescents in a Northwest Chinese City | 0 | 1 | 1 | 0 | 1 | 1 | 1 | 1 | 1 | 1 | 8 | |
| Li-2020 | Ethnic, Geographic, and Seasonal Differences of Vitamin D Status Among Adults in South-West China | 0 | 0 | 0 | 0 | 1 | 1 | 1 | 1 | 1 | 1 | 6 | |
| Li-2020 | Widespread Vitamin D Deficiency and its Sex-Specific Association with Adiposity in Chinese Children and Adolescents | 1 | 1 | 1 | 0 | 1 | 1 | 1 | 1 | 1 | 1 | 9 | |
| Li-2021 | Sterol Metabolism and Protein Metabolism are Differentially Correlated with Sarcopenia in Asian Chinese Men and Women | 0 | 1 | 0 | 1 | 1 | 1 | 1 | 1 | 1 | 1 | 8 | |
| Lim-2012 | Vitamin D Inadequacy is Associated with Significant Coronary Artery Stenosis in a Community-Based Elderly Cohort: The Korean Longitudinal Study on Health and Aging | 0 | 1 | 1 | 0 | 1 | 1 | 1 | 1 | 1 | 1 | 8 | |
| Lingham-2021 | Time Spent Outdoors Through Childhood and Adolescence - Assessed by 25-Hydroxyvitamin D Concentration - and Risk of Myopia at 20 Years | 0 | 1 | 1 | 1 | 1 | 1 | 1 | 1 | 1 | 1 | 9 | |
| Liu-2018 | Adult Serum 25(OH)D3 In Gansu Province, Northwest China: A Cross-Sectional Study | 0 | 0 | 0 | 0 | 1 | 1 | 1 | 1 | 1 | 1 | 6 | |
| Liu-2018 | High Prevalence of Insufficient Vitamin D Intake and Serum 25-hydroxyvitamin D in Chinese School-Age Children: A Cross-Sectional Study | 0 | 1 | 1 | 1 | 1 | 1 | 1 | 1 | 1 | 1 | 9 | |
| Liu-2023 | Healthy Lifestyles are Associated with Better Vitamin D Status in Community-Dwelling Older Men: The Health in Men Study (Hims) | 0 | 0 | 0 | 0 | 1 | 1 | 1 | 1 | 0 | 1 | 5 | |
| Lopes-2014 | A Predictive Model of Vitamin D Insufficiency in Older Community People: From the Sao Paulo Aging & Health Study (SPAH) | 0 | 0 | 0 | 0 | 1 | 1 | 1 | 1 | 1 | 1 | 6 | |
| Lu-2012 | Associations Between Common Variants in GC And DHCR7/NADSYN1 and Vitamin D Concentration in Chinese Hans | 0 | 1 | 1 | 0 | 1 | 1 | 1 | 1 | 1 | 1 | 8 | |
| Luo-2021 | The Relationship Between Sarcopenia and Vitamin D Levels in Adults of Different Ethnicities: Findings from the West China Health and Aging Trend Study | 0 | 1 | 1 | 0 | 1 | 1 | 0 | 1 | 1 | 1 | 7 | |
| Lutsey-2016 | Short-Term Variability of Vitamin D-Related Biomarkers | 0 | 1 | 1 | 0 | 1 | 1 | 1 | 1 | 1 | 1 | 8 | |
| Luxwolda-2013 | Vitamin D Status Indicators in Indigenous Populations in East Africa | 0 | 0 | 0 | 0 | 1 | 1 | 1 | 1 | 1 | 1 | 6 | |
| Lwow-2020 | Vitamin D and Selected Cytokine Concentrations in Postmenopausal Women in Relation to Metabolic Disorders and Physical Activity | 0 | 0 | 0 | 0 | 1 | 1 | 1 | 1 | 1 | 1 | 6 | |
| Macdonald-2011 | Skin Color Change in Caucasian Postmenopausal Women Predicts Summer-Winter Change in 25-hydroxyvitamin D: Findings from the ANSAVID Cohort Study | 0 | 1 | 0 | 0 | 1 | 1 | 1 | 1 | 1 | 1 | 7 | |
| Macdonell-2016 | Vitamin D Status and its Predictors in New Zealand Aged-Care Residents Eligible for A Government-Funded Universal Vitamin D Supplementation Programme | 0 | 0 | 0 | 0 | 1 | 1 | 1 | 1 | 1 | 1 | 6 | |
| Macdonell-2021 | Multiple Micronutrients, Including Zinc, Selenium and Iron, are Positively Associated with Anemia in New Zealand Aged Care Residents | 0 | 0 | 0 | 0 | 1 | 1 | 1 | 1 | 1 | 1 | 6 | |
| Maddah-2009 | Vitamin D Insufficiency Among Postmenopausal Women in Urban and Rural Areas in Guilan, Northern Iran | 0 | 1 | 1 | 0 | 1 | 1 | 1 | 1 | 1 | 1 | 8 | |
| Madsen-2014 | Vitamin D Status and its Determinants in Children and Adults Among Families in Late Summer in Denmark | 0 | 1 | 1 | 0 | 1 | 1 | 1 | 1 | 1 | 1 | 8 | |
| Majumdar-2011 | Vitamin D Status and Metabolic Syndrome in Asian Indians | 0 | 0 | 0 | 0 | 1 | 1 | 1 | 1 | 1 | 1 | 6 | |
| Mallah-2011 | Plasma Concentrations Of 25-hydroxyvitamin D Among Jordanians: Effect of Biological and Habitual Factors on Vitamin D Status | 0 | 0 | 0 | 0 | 1 | 1 | 1 | 1 | 1 | 1 | 6 | |
| Manios-2017 | Prevalence of Vitamin D Deficiency and Insufficiency Among Schoolchildren in Greece: The Role of Sex, Degree of Urbanisation and Seasonality | 1 | 1 | 1 | 0 | 1 | 1 | 1 | 1 | 1 | 1 | 9 | |
| Mansuri-2016 | Traditional Foods and 25(OH)D Concentrations in a Subarctic First Nations Community | 0 | 1 | 0 | 0 | 1 | 1 | 1 | 1 | 1 | 1 | 7 | |
| Marasinghe-2015 | Micronutrient Status and its Relationship with Nutritional Status in Preschool Children in Urban Sri Lanka | 0 | 1 | 1 | 0 | 1 | 1 | 1 | 1 | 1 | 1 | 8 | |
| Martini-2013 | Prevalence and Correlates of Calcium and Vitamin D Status Adequacy in Adolescents, Adults, And Elderly from the Health Survey-Sao Paulo | 0 | 0 | 1 | 0 | 1 | 1 | 1 | 1 | 1 | 1 | 7 | |
| Marwaha-2011 | Bone Health in Healthy Indian Population Aged 50 Years and Above | 0 | 0 | 0 | 0 | 1 | 1 | 1 | 1 | 1 | 1 | 6 | |
| Masoud-2020 | The Association Between Iron and Vitamin D Status in Arab Adolescents | 0 | 1 | 1 | 0 | 1 | 1 | 1 | 1 | 1 | 1 | 8 | |
| Mat-2018 | Vitamin D Deficiency is Associated with Ethnicity and Knee Pain in a Multi-Ethnic South-East Asian Nation: Results from Malaysian Elders Longitudinal Research (Melor) | 0 | 1 | 1 | 0 | 1 | 1 | 0 | 1 | 1 | 1 | 7 | |
| Mata-Granados-2013 | Lower Vitamin E Serum Levels are Associated with Osteoporosis in Early Postmenopausal Women: A Cross-Sectional Study | 0 | 0 | 0 | 1 | 1 | 1 | 1 | 1 | 1 | 1 | 7 | |
| Mavroeidi-2013 | Seasonal Variation in 25(OH)D at Aberdeen (57degrees) And Bone Health Indicators- Could Holidays in the Sun and Cod Liver Oil Supplements Alleviate Deficiency? | 0 | 0 | 1 | 0 | 1 | 1 | 1 | 1 | 1 | 1 | 7 | |
| Mayer-2017 | Synergistic Effect of Low K and D Vitamin Status on Arterial Stiffness in a General Population | 0 | 1 | 1 | 1 | 1 | 1 | 1 | 1 | 1 | 1 | 9 | |
| Mba-2022 | Association Between Circulating 25-Hydroxyvitamin D and Cardiometabolic Risk Factors in Adults in Rural and Urban Settings | 0 | 1 | 1 | 0 | 1 | 1 | 1 | 1 | 1 | 1 | 8 | |
| Mccullough-2021 | The Cancer Prevention Study-3 FFQ is a Reliable and Valid Measure of Nutrient Intakes Among Racial/Ethnic Subgroups, Compared with 24-Hour Recalls and Biomarkers | 0 | 0 | 0 | 0 | 1 | 1 | 1 | 1 | 1 | 1 | 6 | |
| Meems-2015 | Low Levels of Vitamin D Are Associated with Multimorbidity: Results from the Lifelines Cohort Study | 0 | 0 | 0 | 0 | 1 | 1 | 1 | 1 | 1 | 1 | 6 | |
| Mehboobali-2015 | High Prevalence of Vitamin D Deficiency and Insufficiency in A Low-Income Peri-Urban Community in Karachi | 0 | 0 | 0 | 0 | 1 | 1 | 1 | 1 | 1 | 1 | 6 | |
| Menant-2011 | Relationships Between Serum Vitamin D Levels, Neuromuscular and Neuropsychological Function and Falls in Older Men and Women | 0 | 1 | 1 | 0 | 1 | 1 | 1 | 1 | 1 | 1 | 8 | |
| Meng-2017 | Serum 25-hydroxyvitamin D and Elderly Skeletal Muscle Mass and Function in Urban North China | 0 | 0 | 0 | 0 | 1 | 1 | 1 | 1 | 1 | 1 | 6 | |
| Meo-2016 | Association of Serum 25-hydroxy-Vitamin D with Lung Function and Fractional Exhaled Nitric Oxide | 0 | 0 | 0 | 0 | 1 | 1 | 1 | 1 | 1 | 1 | 6 | |
| Meoli-2021 | Vitamin D Status in Adolescents During COVID-19 Pandemic: A Cross-Sectional Comparative Study | 0 | 1 | 1 | 0 | 1 | 1 | 1 | 1 | 1 | 1 | 8 | |
| Merchant-2018 | Vitamin D Binding Protein and Vitamin D Levels in Multi-Ethnic Population | 0 | 1 | 1 | 0 | 1 | 1 | 1 | 1 | 1 | 1 | 8 | |
| Michalus-2013 | Assessment of The Supplementation of Vitamin D in Children Aged 9-15 Years from Lodz | 0 | 0 | 0 | 0 | 1 | 1 | 1 | 1 | 1 | 1 | 6 | |
| Middelkoop-2022 | Prevalence and Determinants of Vitamin D Deficiency in 1825 Cape Town Primary Schoolchildren: A Cross-Sectional Study | 0 | 1 | 0 | 1 | 1 | 1 | 1 | 1 | 1 | 1 | 8 | |
| Mielgo-Ayuso-2017 | Regular Breakfast Consumption Is Associated with Higher Blood Vitamin Status in Adolescents: The HELENA (Healthy Lifestyle in Europe By Nutrition in Adolescence) Study | 0 | 1 | 1 | 1 | 1 | 1 | 1 | 1 | 1 | 1 | 9 | |
| Miettinen-2014 | Association of Serum 25-hydroxyvitamin D with Lifestyle Factors and Metabolic and Cardiovascular Disease Markers: Population-Based Cross-Sectional Study (FIN-D2D) | 0 | 1 | 1 | 1 | 1 | 1 | 1 | 1 | 1 | 1 | 9 | |
| Milagres-2020 | Cutoff Point Estimation for Serum Vitamin D Concentrations to Predict Cardiometabolic Risk in Brazilian Children | 0 | 1 | 1 | 0 | 1 | 1 | 1 | 1 | 1 | 1 | 8 | |
| Mirza-2022 | Assessment of Vitamin D Levels and Other Bone Related Biochemical Markers in Healthy Adults in Rural Population of Uttarakhand, India | 0 | 0 | 0 | 0 | 1 | 1 | 1 | 1 | 1 | 1 | 6 | |
| Mogire-2021 | Prevalence and Predictors of Vitamin D Deficiency in Young African Children | 0 | 0 | 0 | 0 | 1 | 1 | 1 | 1 | 1 | 1 | 6 | |
| Moran-2013 | Hormonal Relationships to Bone Mass in Elderly Spanish Men as Influenced by Dietary Calcium and Vitamin D | 0 | 0 | 0 | 0 | 1 | 1 | 1 | 1 | 1 | 1 | 6 | |
| Muhairi-2013 | Vitamin D Deficiency Among Healthy Adolescents in Al Ain, United Arab Emirates | 0 | 1 | 1 | 0 | 1 | 1 | 1 | 1 | 1 | 1 | 8 | |
| Mukhopadhyay-2019 | Inverse Relationship Between 25 Hydroxy Vitamin D and Parathormone: are There Two Inflection Points? | 0 | 0 | 0 | 0 | 1 | 1 | 1 | 1 | 1 | 1 | 6 | |
| Mustafa-2021 | Concentration Levels of Serum 25-hydroxyvitamin-D and Vitamin D Deficiency Among Children and Adolescents of India: A Descriptive Cross-Sectional Study | 1 | 1 | 1 | 0 | 1 | 1 | 1 | 1 | 1 | 1 | 9 | |
| Mutt-2019 | Vitamin D Status and Components of Metabolic Syndrome in Older Subjects from Northern Finland (Latitude 65degreenorth) | 0 | 1 | 0 | 0 | 1 | 1 | 1 | 1 | 1 | 1 | 7 | |
| Naganuma-2022 | Low Serum 25-hydroxyvitamin D Level is Associated with Obesity and Atherogenesis in Adolescent Boys | 0 | 0 | 0 | 0 | 1 | 1 | 1 | 1 | 1 | 1 | 6 | |
| Nakamura-2011 | C-Reactive Protein Predicts Incident Fracture in Community-Dwelling Elderly Japanese Women: The Muramatsu Study | 0 | 1 | 1 | 0 | 1 | 1 | 1 | 1 | 1 | 1 | 8 | |
| Nakamura-2019 | Serum 25-Hydroxyvitamin D3 Levels and Poor Sleep Quality in a Japanese Population: The DOSANCO Health Study | 0 | 0 | 0 | 1 | 1 | 1 | 0 | 1 | 1 | 1 | 6 | |
| Nakamura-2020 | Predictors of Decline in Vitamin D Status in Middle-Aged and Elderly Individuals: A 5-Year Follow-Up Study | 0 | 0 | 1 | 1 | 1 | 1 | 1 | 1 | 1 | 1 | 8 | |
| Nakaoka-2021 | A High-Fat Diet in the Presence of Vitamin D Deficiency Status Is Associated with a Negative Influence on Calcaneal Quantitative Ultrasound Parameters in Young Adults: A Cross-Sectional Study | 0 | 0 | 0 | 0 | 1 | 1 | 1 | 1 | 1 | 1 | 6 | |
| Nakhaee-2019 | Vitamin D Deficiency and Its Associated Risk Factors in Normal Adult Population of Birjand, Iran | 0 | 0 | 1 | 0 | 1 | 1 | 1 | 1 | 0 | 1 | 6 | |
| Nalsen-2020 | Vitamin D Status in Children and Adults in Sweden: Dietary Intake And 25-hydroxyvitamin D Concentrations in Children Aged 10 12 Years and Adults Aged 18-80 Years | 1 | 1 | 0 | 0 | 1 | 1 | 1 | 1 | 1 | 1 | 8 | |
| Narchi-2015 | Hypovitaminosis D in Adolescent Females–An Analytical Cohort Study in the United Arab Emirates | 0 | 1 | 1 | 0 | 1 | 1 | 1 | 1 | 1 | 1 | 8 | |
| Navarrete-Reyes-2015 | 25-OH-Vitamin D is Not Associated with Cognitive Performance Among Mexican Community-Dwelling Older Persons | 0 | 1 | 1 | 1 | 1 | 1 | 1 | 1 | 1 | 1 | 9 | |
| Neyestani-2012 | High Prevalence of Vitamin D Deficiency in School-Age Children in Tehran, 2008: A Red Alert | 0 | 1 | 1 | 1 | 1 | 1 | 1 | 1 | 1 | 1 | 9 | |
| Nguyen-2012 | Vitamin D Deficiency in Northern Vietnam: Prevalence, Risk Factors and Associations with Bone Mineral Density | 0 | 1 | 1 | 1 | 1 | 1 | 1 | 1 | 1 | 1 | 9 | |
| Nguyen-2020 | Vitamin D and Bone Mineral Density Status, and Their Correlation with Bone Turnover Markers in Healthy Children Aged 6-14 in Vietnam | 0 | 1 | 1 | 0 | 1 | 1 | 1 | 1 | 1 | 1 | 8 | |
| Ni chaoimh-2018 | Low Vitamin D Deficiency in Irish Toddlers Despite Northerly Latitude and a High Prevalence of Inadequate Intakes | 0 | 1 | 1 | 0 | 1 | 1 | 1 | 1 | 1 | 1 | 8 | |
| Niafar-2009 | Vitamin D Status in Healthy Postmenopausal Iranian Women | 0 | 1 | 1 | 0 | 1 | 1 | 1 | 1 | 1 | 1 | 8 | |
| Nichols-2012 | Vitamin D Status and Determinants of Deficiency Among Non-Pregnant Jordanian Women of Reproductive Age | 1 | 1 | 1 | 0 | 1 | 1 | 1 | 1 | 1 | 1 | 9 | |
| Nichols-2015 | Vitamin D Status and Associated Factors of Deficiency Among Jordanian Children of Preschool Age | 1 | 1 | 1 | 0 | 1 | 1 | 1 | 1 | 1 | 1 | 9 | |
| Nielsen-2016 | Associations Between Vitamin D Status and Type 2 Diabetes Measures Among Inuit in Greenland may be Affected by Other Factors | 1 | 1 | 1 | 0 | 1 | 1 | 1 | 1 | 1 | 1 | 9 | |
| Niimi-2022 | Relationships Between QUS And HR-pQCT, DXA, and Bone Turnover Markers | 0 | 0 | 0 | 0 | 1 | 1 | 1 | 1 | 1 | 1 | 6 | |
| Nikooyeh-2017 | Vitamin D Status and Cardiometabolic Risk Factors Across Latitudinal Gradient in Iranian Adults: National Food and Nutrition Surveillance | 1 | 1 | 1 | 1 | 1 | 1 | 1 | 1 | 1 | 1 | 10 | |
| Nikooyeh-2017 | Vitamin D Status, Latitude and their Associations with some Health Parameters in Children: National Food and Nutrition Surveillance | 1 | 1 | 1 | 0 | 1 | 1 | 1 | 1 | 1 | 1 | 9 | |
| Nurbazlin-2013 | Effects of Sun Exposure on 25(OH) Vitamin D Concentration in Urban and Rural Women in Malaysia | 0 | 0 | 0 | 0 | 1 | 1 | 1 | 1 | 1 | 1 | 6 | |
| O Breasail-2023 | Longitudinal Change in Bone Density, Geometry, and Estimated Bone Strength in Older Men and Women from The Gambia: Findings from the Gambian Bone and Muscle Aging Study (GamBAS) | 0 | 0 | 0 | 0 | 1 | 1 | 1 | 0 | 0 | 1 | 4 | |
| O'brien-2017 | Declines in Traditional Marine Food Intake and Vitamin D Levels from the 1960s to Present in Young Alaska Native Women | 0 | 1 | 1 | 0 | 1 | 1 | 1 | 1 | 1 | 1 | 8 | |
| Oberg-2014 | Vitamin D Deficiency and Lifestyle Risk Factors in a Norwegian Adolescent Population | 0 | 1 | 1 | 1 | 1 | 1 | 1 | 1 | 1 | 1 | 9 | |
| Oden Akman-2011 | Frequency of Vitamin D Insufficiency in Healthy Children Between 1 and 16 Years of Age in Turkey | 0 | 1 | 0 | 0 | 1 | 1 | 1 | 1 | 1 | 1 | 7 | |
| Okan-2020 | Effect of Sunlight Exposure on Vitamin D Status of Individuals Living in a Nursing Home and their Own Homes | 0 | 0 | 0 | 0 | 1 | 1 | 1 | 1 | 1 | 1 | 6 | |
| Orces-2015 | Vitamin D Status Among Older Adults Residing in the Littoral and Andes Mountains in Ecuador | 0 | 1 | 1 | 0 | 1 | 1 | 1 | 1 | 1 | 1 | 8 | |
| Oshiro-2022 | Vitamin D Deficiency and Insufficiency in Hawaii: Levels and Sources of Serum Vitamin D in Older Adults | 0 | 1 | 1 | 0 | 1 | 1 | 1 | 1 | 1 | 1 | 8 | |
| Oudshoorn-2012 | Better Knowledge on Vitamin D and Calcium in Older People is Associated with a Higher Serum Vitamin D Level and a Higher Daily Dietary Calcium Intake | 0 | 0 | 0 | 0 | 1 | 1 | 1 | 1 | 1 | 1 | 6 | |
| Oussedik-Lehtihet-2017 | Hypovitaminosis D and its Associated Factors in North Algerian Postmenopausal Women: Results of a Cross-Sectional Study | 0 | 0 | 1 | 0 | 1 | 1 | 1 | 1 | 1 | 1 | 7 | |
| Overby-2014 | Test-Retest Reliability and Validity of a Web-Based Food-Frequency Questionnaire for Adolescents Aged 13-14 to be Used in the Norwegian Mother and Child Cohort Study (MoBa) | 0 | 0 | 0 | 0 | 1 | 1 | 1 | 1 | 1 | 1 | 6 | |
| Paes-Silva-2018 | Prevalence and Factors Associated with Fat-Soluble Vitamin Deficiency in Adolescents | 0 | 1 | 1 | 0 | 1 | 1 | 1 | 1 | 1 | 1 | 8 | |
| Pan-2020 | Bone Health and its Association with Vitamin D and Other Covariates: A Community-Based Study Among Women in a Rural Area of West Bengal | 0 | 1 | 1 | 0 | 1 | 1 | 1 | 1 | 1 | 1 | 8 | |
| Panahi-2022 | Association of Amino Acid Metabolites with Osteoporosis, a Metabolomic Approach: Bushehr Elderly Health Program | 0 | 1 | 1 | 0 | 1 | 1 | 1 | 1 | 1 | 1 | 8 | |
| Pang-2021 | Relationship Between Serum 25OH-Vitamin D2 Level and Vitamin D Status of Children Aged 3-5 Years in China | 1 | 1 | 1 | 1 | 1 | 1 | 1 | 1 | 1 | 1 | 10 | |
| Pankiv-2020 | Association of Vitamin D Status with Body Mass Index in Adolescents in Ukraine | 0 | 0 | 0 | 0 | 1 | 1 | 1 | 1 | 1 | 0 | 5 | |
| Pannu-2017 | The Associations of Vitamin D Status and Dietary Calcium with the Metabolic Syndrome: An Analysis of the Victorian Health Monitor Survey | 0 | 1 | 1 | 0 | 1 | 1 | 1 | 1 | 1 | 1 | 8 | |
| Pantovic-2019 | Vitamin D is Inversely Related to Obesity: Cross-Sectional Study in a Small Cohort of Serbian Adults | 0 | 0 | 0 | 0 | 1 | 1 | 1 | 1 | 1 | 1 | 6 | |
| Park-2015 | Interaction Effect of Serum 25-hydroxyvitamin D levels And CYP1A1, CYP1B1polymorphisms on Blood Pressure in an Elderly Population | 0 | 0 | 0 | 0 | 1 | 1 | 1 | 1 | 1 | 1 | 6 | |
| Park-2018 | Vitamin D Status in South Korean Population: Seven-Year Trend from the KNHANES | 1 | 1 | 1 | 1 | 1 | 1 | 1 | 1 | 1 | 1 | 10 | |
| Passi-Solar-2020 | Nutritional and Metabolic Benefits Associated with Active and Public Transport: Results from the Chilean National Health Survey, ENS 2016-2017 | 1 | 1 | 1 | 0 | 1 | 1 | 1 | 1 | 1 | 1 | 9 | |
| Patel-2016 | Dietary Calcium Intake Influences the Relationship Between Serum 25-hydroxyvitamin D3 (25OHD) Concentration and Parathyroid Hormone (PTH) Concentration | 0 | 1 | 1 | 0 | 1 | 1 | 1 | 1 | 1 | 1 | 8 | |
| Patriota-2022 | Association Between Anthropometric Markers of Adiposity, Adipokines and Vitamin D Levels | 0 | 0 | 0 | 0 | 1 | 1 | 1 | 1 | 0 | 1 | 5 | |
| Pazaitou-Panayiotou-2012 | Height, Whole Body Surface Area, Gender, Working Outdoors, and Sunbathing in Previous Summer are Important Determinants of Serum 25-hydroxyvitamin D Levels | 0 | 0 | 0 | 0 | 1 | 1 | 1 | 1 | 1 | 1 | 6 | |
| Peng-2013 | Association Between Vitamin D Insufficiency and Elevated Serum Uric Acid Among Middle-Aged and Elderly Chinese Han Women | 0 | 1 | 1 | 1 | 1 | 1 | 1 | 1 | 1 | 1 | 9 | |
| Perez-Bravo-2022 | Vitamin D Status and Obesity in Children from Chile | 0 | 0 | 0 | 0 | 1 | 1 | 1 | 1 | 1 | 1 | 6 | |
| Peters-2012 | The Influence of Breakfast and Dairy Products on Dietary Calcium and Vitamin D Intake in Postpubertal Adolescents and Young Adults | 0 | 0 | 0 | 0 | 1 | 1 | 1 | 1 | 1 | 1 | 6 | |
| Petrenya-2020 | Vitamin D Status in a Multi-Ethnic Population of Northern Norway: The Saminor 2 Clinical Survey | 0 | 1 | 1 | 0 | 1 | 1 | 1 | 1 | 1 | 1 | 8 | |
| Pilz-2012 | Low 25-Hydroxyvitamin D is Associated with Increased Mortality in Female Nursing Home Residents | 0 | 0 | 0 | 0 | 1 | 1 | 1 | 1 | 1 | 1 | 6 | |
| Poh-2016 | 25-Hydroxy-Vitamin D Demography and the Risk of Vitamin D Insufficiency in the South East Asian Nutrition Surveys (SEANUTS) | 1 | 1 | 1 | 0 | 1 | 1 | 1 | 1 | 1 | 1 | 9 | |
| Poopedi-2022 | Is Vitamin D Status Associated with Non-Communicable Disease Risk in Children? A Cohort Study | 0 | 0 | 0 | 0 | 1 | 1 | 1 | 1 | 1 | 1 | 6 | |
| Pulungan-2021 | Anthropometric, Biochemical and Hormonal Profiles of the Partially Admixed Pygmoid Group in Rampasasa (Flores, Indonesia) | 0 | 1 | 1 | 0 | 1 | 1 | 1 | 1 | 1 | 1 | 8 | |
| Pusparini-2016 | Increased Matrix Metalloproteinase-9 In Male Elderly with Low 25-Hydroxy-Vitamin D | 0 | 0 | 1 | 0 | 1 | 1 | 1 | 1 | 1 | 1 | 7 | |
| Qiao-2013 | Serum 25(OH)D Level and Parathyroid Hormone in Chinese Adult Population: A Cross-Sectional Study in Guiyang Urban Community from Southeast of China | 0 | 1 | 1 | 1 | 1 | 1 | 1 | 1 | 1 | 1 | 9 | |
| Quah-2018 | Risk Factors of Vitamin D Deficiency Among 15-Year-Old Adolescents Participating in the Malaysian Health and Adolescents Longitudinal Research Team Study (MyHeARTs) | 0 | 1 | 1 | 1 | 1 | 1 | 1 | 1 | 1 | 1 | 9 | |
| Rabenberg-2018 | Implications of Standardization of Serum 25-hydroxyvitamin D Data for The Evaluation of Vitamin D Status in Germany, Including a Temporal Analysis | 1 | 1 | 1 | 0 | 1 | 1 | 1 | 1 | 0 | 1 | 8 | |
| Rad-2015 | Competitive Protein-Binding Assay-Based Enzyme-Immunoassay Method, Compared to High-Pressure Liquid Chromatography, Has a Very Lower Diagnostic Value to Detect Vitamin D Deficiency In 9-12 Years Children | 0 | 0 | 0 | 0 | 1 | 1 | 1 | 1 | 1 | 1 | 6 | |
| Rafraf-2014 | Vitamin D Status and its Relationship with Metabolic Syndrome Risk Factors Among Adolescent Girls in Boukan, Iran | 0 | 1 | 1 | 1 | 1 | 1 | 1 | 1 | 1 | 1 | 9 | |
| Rahmadhani-2017 | The Associations Between VDR BSMI Polymorphisms and Risk of Vitamin D Deficiency, Obesity and Insulin Resistance in Adolescents Residing in a Tropical Country | 0 | 1 | 1 | 0 | 1 | 1 | 1 | 1 | 1 | 1 | 8 | |
| Ramankutty-2014 | Ultraviolet Radiation Exposure and Serum Vitamin D Levels in Young Children | 0 | 0 | 0 | 0 | 1 | 1 | 1 | 1 | 1 | 1 | 6 | |
| Raposo-2017 | Vitamin D, Parathyroid Hormone and Metabolic Syndrome - The PORMETS Study | 1 | 1 | 1 | 0 | 1 | 1 | 1 | 1 | 1 | 1 | 9 | |
| Raszewski-2019 | 25-hydroxyvitamin D Status and Its Impact on Cognitive Functions in Postmenopausal Woman | 0 | 0 | 0 | 1 | 1 | 1 | 1 | 1 | 1 | 1 | 7 | |
| Rathnayake-2021 | Factors Associated with Measures of Sarcopenia in Pre And Postmenopausal Women | 0 | 0 | 0 | 0 | 1 | 1 | 1 | 1 | 1 | 1 | 6 | |
| Rathod-2015 | Association of Urinary Calcium Excretion with Serum Calcium and Vitamin D Levels | 0 | 0 | 0 | 0 | 1 | 1 | 1 | 1 | 1 | 1 | 6 | |
| Raulio-2017 | Successful Nutrition Policy: Improvement of Vitamin D Intake and Status in Finnish Adults Over the Last Decade | 1 | 1 | 1 | 0 | 1 | 1 | 1 | 1 | 1 | 1 | 9 | |
| Rautenbach-2022 | Associations Between 25-hydroxyvitamin D And Total and Gamma' Fibrinogen and Plasma Clot Properties and Gene Interactions in a Group of Healthy Black South African Women | 0 | 0 | 1 | 0 | 1 | 1 | 1 | 1 | 1 | 1 | 7 | |
| Rehman-2020 | Demographic Differences and Trends of Vitamin D Levels Among the Teenaged Girls in Balochistan | 0 | 1 | 1 | 0 | 1 | 1 | 1 | 1 | 1 | 1 | 8 | |
| Rezaei-2023 | The Prevalence and Determinants of Vitamin D Status Among Older Adults: Data from a Longitudinal Aging Study | 0 | 0 | 1 | 0 | 1 | 1 | 1 | 1 | 1 | 1 | 7 | |
| Rips-2023 | Severe Deficiency of Vitamin D Has No Negative Effect on Physical Performance During Military Training | 0 | 0 | 0 | 0 | 1 | 1 | 1 | 1 | 1 | 1 | 6 | |
| Riverin-2014 | Prevalence of Vitamin D Insufficiency Among Healthy School-Age Cree Children | 0 | 1 | 1 | 0 | 1 | 1 | 1 | 1 | 1 | 1 | 8 | |
| Robinson-2017 | Correlates and Family Aggregation of Vitamin D Concentrations in School-Aged Children and Their Parents in Nine Mesoamerican Countries | 0 | 0 | 0 | 0 | 1 | 1 | 1 | 1 | 1 | 1 | 6 | |
| Rodriguez-Rodriguez-2011 | Vitamin D Deficiency is an Independent Predictor of Elevated Triglycerides in Spanish School Children | 0 | 1 | 1 | 0 | 1 | 1 | 1 | 1 | 1 | 1 | 8 | |
| Rosendahl-2017 | A History of Cow's Milk Allergy is Associated with Lower Vitamin D Status in Schoolchildren | 0 | 1 | 1 | 0 | 1 | 1 | 1 | 1 | 1 | 1 | 8 | |
| Rufus-Membere-2019 | Associations Between Bone Impact Microindentation and Clinical Risk Factors for Fracture | 0 | 1 | 1 | 0 | 1 | 1 | 1 | 1 | 1 | 1 | 8 | |
| Saberi-Karimian-2023 | The National Health Program for Vitamin D Supplementation in a Developing Country | 0 | 0 | 0 | 0 | 1 | 1 | 1 | 1 | 0 | 1 | 5 | |
| Sacheck-2011 | Vitamin D Deficiency, Adiposity, and Cardiometabolic Risk in Urban Schoolchildren | 0 | 0 | 0 | 0 | 1 | 1 | 1 | 1 | 1 | 1 | 6 | |
| Sadiq-2018 | A Cross-Sectional Study to Determine the Rate of Shortage of Vitamin D Especially in the Low Standard Urban Community | 0 | 0 | 0 | 0 | 1 | 1 | 1 | 1 | 1 | 1 | 6 | |
| Saki-2017 | Vitamin D Deficiency and its Associated Risk Factors in Children and Adolescents in Southern Iran | 0 | 1 | 1 | 0 | 1 | 1 | 1 | 1 | 1 | 1 | 8 | |
| Sales-2023 | Dietary Inadequacies Overestimate the Blood Deficiencies of Magnesium, Zinc, and Vitamins A, C, E, And D Among Residents of Sao Paulo | 0 | 0 | 1 | 0 | 1 | 1 | 1 | 1 | 1 | 1 | 7 | |
| Samefors-2014 | Vitamin D Deficiency in Elderly People in Swedish Nursing Homes is Associated with Increased Mortality | 0 | 0 | 0 | 0 | 1 | 1 | 1 | 1 | 1 | 1 | 6 | |
| Santos Araujo-2017 | Prevalence of Hypovitaminosis D and Associated Factors in Adolescent Students of a Capital of Northeastern Brazil | 0 | 1 | 1 | 0 | 1 | 1 | 1 | 1 | 1 | 1 | 8 | |
| Santos-2017 | Vitamin D Status and Associated Factors Among Portuguese Older Adults: Results from The Nutrition Up 65 Cross-Sectional Study | 1 | 1 | 1 | 0 | 1 | 1 | 1 | 1 | 1 | 1 | 9 | |
| Santos-2019 | Prevalence of Vitamin D Deficiency in Women from Southern Brazil and Association with Vitamin D-Binding Protein Levels and GC-DBP Gene Polymorphisms | 0 | 1 | 1 | 0 | 1 | 1 | 1 | 1 | 1 | 1 | 8 | |
| Sari-2021 | The Moderate Correlation Between 25(OH)D Serum and Saliva in Healthy People with Low Vitamin D Intake | 0 | 0 | 0 | 0 | 1 | 1 | 1 | 1 | 1 | 1 | 6 | |
| Sarma-2019 | Vitamin D Status of School Children in and Around Guwahati | 0 | 1 | 1 | 0 | 1 | 1 | 1 | 1 | 1 | 1 | 8 | |
| Schramm-2017 | Impact of Season and Different Vitamin D Thresholds on Prevalence of Vitamin D Deficiency in Epidemiological Cohorts-A Note of Caution | 0 | 1 | 1 | 0 | 1 | 1 | 1 | 1 | 1 | 1 | 8 | |
| Seo-2013 | Low Vitamin D Status is Associated with Nonalcoholic Fatty Liver Disease Independent of Visceral Obesity in Korean Adults | 0 | 1 | 1 | 0 | 1 | 1 | 1 | 1 | 1 | 1 | 8 | |
| Seo-na | Association Between Visceral Obesity and Sarcopenia and Vitamin D Deficiency in Older Koreans: The Ansan Geriatric Study | 0 | 1 | 1 | 0 | 1 | 1 | 1 | 1 | 1 | 1 | 8 | |
| Shah-2014 | Subclinical Rickets | 0 | 0 | 0 | 0 | 1 | 1 | 0 | 1 | 1 | 1 | 5 | |
| Shaheen-2011 | Relationship of Vitamin D Status to Adult Lung Function and COPD | 0 | 1 | 1 | 0 | 1 | 1 | 1 | 1 | 1 | 1 | 8 | |
| Shaikh-2016 | Prevalence of Vitamin D Deficiency and Calcium Homeostasis in Saudi Children | 0 | 0 | 0 | 0 | 1 | 1 | 1 | 1 | 1 | 1 | 6 | |
| Shanyhin-2022 | Hygienic Assessment of the Prevalence of Vitamin D Deficiency States Associated with Dyslipidemia in the Adult Population of Southern Ukraine | 0 | 0 | 0 | 0 | 1 | 1 | 0 | 1 | 1 | 1 | 5 | |
| Sharma-2018 | Vitamin D Status in Cold Trans-Himalayan Deserts at Altitude Of 4000 Meter and Above in India | 0 | 1 | 1 | 0 | 1 | 1 | 1 | 1 | 1 | 1 | 8 | |
| Sheen-2016 | Interaction Between 25-Hydroxyvitamin D and Variants At 17q12-21 on Respiratory Infections | 0 | 0 | 0 | 0 | 1 | 1 | 1 | 1 | 1 | 1 | 6 | |
| Shen-2017 | Relationship of Fibroblast Growth Factor 23 (FGF-23) Serum Levels with Low Bone Mass in Postmenopausal Women | 0 | 0 | 0 | 1 | 1 | 1 | 1 | 1 | 1 | 1 | 7 | |
| Sherief-2021 | Vitamin D Status and Healthy Egyptian Adolescents: Where Do We Stand? | 0 | 1 | 0 | 0 | 1 | 1 | 1 | 1 | 1 | 1 | 7 | |
| Shetty-2014 | Osteoporosis in Healthy South Indian Male and The Influence Of Life Style Factors and Vitamin D Status on Bone Mineral Density | 0 | 1 | 1 | 0 | 1 | 1 | 1 | 1 | 1 | 1 | 8 | |
| Shi-2022 | Prevalence and Correlates of Metabolic Syndrome and Its Components in Chinese Children and Adolescents Aged 7-17: The China National Nutrition and Health Survey of Children and Lactating Mothers From 2016-2017 | 1 | 1 | 1 | 1 | 1 | 1 | 1 | 1 | 1 | 1 | 10 | |
| Shridhar-2019 | Serum Calcium Concentrations, Chronic Inflammation and Glucose Metabolism: A Cross-Sectional Analysis in The Andhra Pradesh Children and Parents Study (APCAPS) | 0 | 0 | 1 | 0 | 1 | 1 | 1 | 1 | 1 | 1 | 7 | |
| Singh-2022 | Cross Sectional Study of Vitamin D Levels in Western Rajasthan And Meta-Analysis for Estimation of Vitamin D Levels | 0 | 0 | 0 | 0 | 1 | 1 | 1 | 1 | 1 | 1 | 6 | |
| Singh-2022 | Standardization and Application of a Novel Multiplex Assay for Estimating Micronutrient Status and Inflammatory Markers in Women of Sauria Paharia And Santhal Tribes of Jharkhand | 0 | 1 | 1 | 0 | 1 | 1 | 1 | 1 | 1 | 1 | 8 | |
| Smith-2016 | High Prevalence of Vitamin D Deficiency in Cambodian Women: A Common Deficiency in a Sunny Country | 1 | 1 | 1 | 0 | 1 | 1 | 1 | 1 | 1 | 1 | 9 | |
| Soininen-2016 | Determinants of Serum 25-hydroxyvitamin D Concentration in Finnish Children: The Physical Activity and Nutrition in Children (Panic) Study | 0 | 1 | 1 | 1 | 1 | 0 | 1 | 1 | 1 | 1 | 8 | |
| Sonderman-2012 | Reproducibility of Serum 25-hydroxyvitamin D and Vitamin D-Binding Protein Levels Over Time in a Prospective Cohort Study of Black and White Adults | 0 | 0 | 0 | 0 | 1 | 1 | 1 | 1 | 1 | 1 | 6 | |
| Song-2014 | Association Between Serum 25-hydroxyvitamin D Level and Insulin Resistance in a Rural Population | 0 | 0 | 0 | 0 | 1 | 1 | 1 | 1 | 1 | 1 | 6 | |
| Song-2014 | High Prevalence of Vitamin D Deficiency in Adults Aged 50 Years and Older in Gwangju, Korea: The Dong-Gu Study | 0 | 1 | 1 | 1 | 1 | 1 | 1 | 1 | 1 | 1 | 9 | |
| Soontrapa-2015 | Prevalence of Vitamin D Insufficiency Among Elderly Males Living in Rural Khon Kaen Province, Northeast Thailand | 0 | 0 | 0 | 0 | 1 | 1 | 1 | 1 | 1 | 1 | 6 | |
| Souberbielle-2016 | Prevalence and Determinants of Vitamin D Deficiency in Healthy French Adults: The Variete Study | 0 | 0 | 0 | 0 | 1 | 1 | 1 | 1 | 1 | 1 | 6 | |
| Sousa-2019 | High Prevalence of Hypovitaminosis D in Institutionalized Elderly Individuals is Associated with Summer in a Region with High Ultraviolet Radiation Levels | 0 | 0 | 0 | 1 | 1 | 1 | 1 | 1 | 1 | 1 | 7 | |
| Srimani-2017 | Prevalence and Association of Metabolic Syndrome and Vitamin D Deficiency Among Postmenopausal Women in a Rural Block of West Bengal, India | 0 | 1 | 1 | 0 | 1 | 1 | 1 | 1 | 1 | 1 | 8 | |
| Standahl Olsen-2013 | Plasma 25 Hydroxyvitamin D Level and Blood Gene Expression Profiles: A Cross-Sectional Study of the Norwegian Women and Cancer Post-Genome Cohort | 0 | 0 | 1 | 0 | 1 | 1 | 1 | 1 | 1 | 1 | 7 | |
| Stephan-2023 | The Mediating Role of Biomarkers in the Association Between Subjective Aging and Episodic Memory | 1 | 1 | 0 | 1 | 1 | 1 | 1 | 1 | 1 | 1 | 9 | |
| Su-2020 | The Effect of Physical Activity on Dose-Relationship Between Serum 25-hydroxyvitamin D and Cardiovascular Health Events in Older Adults | 1 | 0 | 1 | 0 | 1 | 1 | 1 | 1 | 1 | 1 | 8 | |
| Summerhays-2020 | Time Trends of Vitamin D Concentrations in Northern Sweden Between 1986 and 2014: A Population-Based Cross-Sectional Study | 1 | 1 | 1 | 0 | 1 | 1 | 1 | 0 | 1 | 1 | 8 | |
| Sun-2015 | Associations Between the Serum 25(Oh)D Concentration and Lipid Profiles in Japanese Men | 0 | 0 | 0 | 0 | 1 | 1 | 1 | 1 | 1 | 1 | 6 | |
| Sundarakumar-2021 | Burden of Vitamin D, Vitamin B12 And Folic Acid Deficiencies in an Aging, Rural Indian Community | 0 | 1 | 1 | 0 | 1 | 1 | 1 | 1 | 0 | 1 | 7 | |
| Sung-2022 | Serum Vitamin D Level Mitigates Fractional Exhaled Nitric Oxide Linked to Bisphenol-A in School-Aged Children | 0 | 0 | 0 | 0 | 1 | 1 | 1 | 1 | 1 | 1 | 6 | |
| Surve-2021 | Determinants of Vitamin D Deficiency Among Under-Five Children in Urban Slums of Mumbai, India | 0 | 0 | 0 | 0 | 1 | 1 | 1 | 1 | 1 | 1 | 6 | |
| Suryanarayana-2018 | Prevalence of Vitamin D Deficiency and its Associated Factors Among the Urban Elderly Population in Hyderabad Metropolitan City, South India | 0 | 1 | 1 | 1 | 1 | 1 | 1 | 1 | 1 | 1 | 9 | |
| Szabo-2017 | The Role of Serum Total and Free 25-Hydroxyvitamin D and PTH Values in Defining Vitamin D Status at the End of Winter: A Representative Survey | 1 | 1 | 1 | 0 | 1 | 1 | 1 | 1 | 1 | 1 | 9 | |
| Szili-2018 | Impact of Genetic Influence on Serum Total- And Free 25-Hydroxyvitamin-D in Humans | 1 | 1 | 1 | 0 | 1 | 1 | 1 | 1 | 1 | 1 | 9 | |
| Szternel-2018 | Association Between Fasting Glucose Concentration, Lipid Profile and 25(OH)D Status in Children Aged 9-11 | 0 | 0 | 0 | 0 | 1 | 1 | 1 | 1 | 1 | 1 | 6 | |
| Talaei-2012 | Prevalence and Cut-Off Point of Vitamin D Deficiency Among Secondary Students of Arak, Iran in 2010 | 0 | 1 | 1 | 0 | 1 | 1 | 1 | 1 | 1 | 1 | 8 | |
| Tamer-2012 | Is Vitamin D Deficiency an Independent Risk Factor for Obesity and Abdominal Obesity in Women? | 0 | 0 | 0 | 0 | 1 | 1 | 1 | 1 | 1 | 1 | 6 | |
| Teng-2022 | Long-Term Exposure to Air Pollution and Lung Function Among Children in China: Association and Effect Modification | 0 | 0 | 1 | 0 | 1 | 1 | 1 | 1 | 1 | 1 | 7 | |
| Teran-2018 | Differences in Nutritional and Health Status in School Children from the Highlands and Lowlands of Bolivia | 0 | 0 | 0 | 0 | 1 | 1 | 1 | 1 | 1 | 1 | 6 | |
| Terock-2021 | Alexithymia Is Associated with Reduced Vitamin D Levels, but not Polymorphisms of the Vitamin D Binding-Protein Gene | 0 | 1 | 1 | 0 | 1 | 1 | 1 | 1 | 1 | 1 | 8 | |
| Thompson-2021 | Population Vitamin D Stores Are Increasing in Tasmania, and this is Associated with Less BMD Loss Over 10 Years | 0 | 1 | 1 | 0 | 1 | 1 | 1 | 1 | 1 | 1 | 8 | |
| Thorisdottir-2016 | Vitamin D Intake and Status in 6-Year-Old Icelandic Children Followed Up from Infancy | 0 | 1 | 1 | 0 | 1 | 1 | 1 | 1 | 1 | 1 | 8 | |
| Thornton-2013 | Vitamin D Deficiency Associated with Increased Incidence of Gastrointestinal and Ear Infections in School-Age Children | 0 | 1 | 1 | 1 | 1 | 1 | 1 | 1 | 1 | 1 | 9 | |
| Tian-2017 | Prevalence of Osteoporosis and Related Lifestyle and Metabolic Factors of Postmenopausal Women and Elderly Men | 0 | 1 | 1 | 0 | 1 | 1 | 1 | 1 | 1 | 1 | 8 | |
| Tokida-2021 | Reference Values for Bone Metabolism in a Japanese Cohort Survey Randomly Sampled from a Basic Elderly Resident Registry | 0 | 1 | 1 | 0 | 1 | 1 | 1 | 1 | 1 | 1 | 8 | |
| Tolppanen-2012 | Risk Factors for Variation in 25-hydroxyvitamin D3 and D-2 Concentrations and Vitamin D Deficiency in Children | 0 | 1 | 0 | 1 | 1 | 1 | 1 | 1 | 1 | 1 | 8 | |
| Trummer-2012 | Allelic Determinants of Vitamin D Insufficiency, Bone Mineral Density, and Bone Fractures | 0 | 0 | 0 | 0 | 1 | 1 | 1 | 1 | 1 | 1 | 6 | |
| Tse-2016 | Food Insecurity, Vitamin D Insufficiency and Respiratory Infections Among Inuit Children | 0 | 0 | 0 | 0 | 1 | 1 | 1 | 1 | 1 | 1 | 6 | |
| Tsugawa-2022 | Vitamin D Status in Japanese Young Women in 2016-2017 And 2020: Seasonal Variation and the Effect of Lifestyle Including Changes Caused by the COVID-19 Pandemic | 0 | 0 | 0 | 0 | 1 | 1 | 1 | 1 | 1 | 1 | 6 | |
| Tsujiguchi-2019 | Dietary Calcium Intake and Hypertension: Importance of Serum Concentrations Of 25-hydroxyvitamin D | 0 | 1 | 1 | 0 | 1 | 1 | 1 | 1 | 1 | 1 | 8 | |
| Tuffaha-2015 | Deficiencies Under Plenty of Sun: Vitamin D Status Among Adults in The Kingdom of Saudi Arabia, 2013 | 1 | 1 | 1 | 0 | 1 | 1 | 0 | 0 | 1 | 1 | 7 | |
| Tung-2021 | An Assessment of Risk Factors for Insufficient Levels of Vitamin D During Early Infancy | 0 | 0 | 0 | 1 | 1 | 1 | 1 | 1 | 1 | 1 | 7 | |
| Vahid-2023 | Association of Diet Quality Indices with Serum and Metabolic Biomarkers in Participants of the ORISCAV-LUX-2 Study | 0 | 0 | 0 | 0 | 1 | 1 | 0 | 0 | 1 | 1 | 4 | |
| Vajdi-2020 | Lifestyle Patterns and their Nutritional, Sociodemographic and Psychological Determinants in a Community-Based Study: A Mixed Approach of Latent Class and Factor Analyses | 0 | 1 | 1 | 0 | 1 | 1 | 1 | 1 | 1 | 1 | 8 | |
| Vansickle-2020 | Comparing Directly Measured Versus Mathematically Calculated Free Serum 25-hydroxy Vitamin D Level in Children | 0 | 0 | 0 | 0 | 1 | 1 | 1 | 1 | 1 | 1 | 6 | |
| Vazquez-Lorente-2023 | Relationship Between Body Composition and Biochemical Parameters with Antioxidant Status in a Healthy Cohort of Postmenopausal Women | 0 | 0 | 0 | 0 | 1 | 0 | 1 | 1 | 1 | 1 | 5 | |
| Verhoeven-2011 | Walk on The Sunny Side of Life - Epidemiology of Hypovitaminosis D and Mental Health in Elderly Nursing Home Residents | 0 | 1 | 0 | 0 | 1 | 1 | 0 | 1 | 1 | 1 | 6 | |
| Vignali-2017 | Development of an Algorithm to Predict Serum Vitamin D Levels Using a Simple Questionnaire Based on Sunlight Exposure | 0 | 1 | 1 | 0 | 1 | 1 | 1 | 1 | 1 | 1 | 8 | |
| Vioque-2019 | Reproducibility and Validity of a Short Food Frequency Questionnaire for Dietary Assessment in Children Aged 7-9 Years in Spain | 0 | 0 | 0 | 0 | 1 | 0 | 1 | 1 | 1 | 1 | 5 | |
| Vissing Landgrebe-2021 | Population-Based Pediatric Reference Values for Serum Parathyroid Hormone, Vitamin D, Calcium, and Phosphate in Danish/North-European White Children and Adolescents | 0 | 1 | 0 | 0 | 1 | 1 | 1 | 1 | 1 | 1 | 7 | |
| Wakayo-2015 | Vitamin D Deficiency and its Predictors in a Country with Thirteen Months of Sunshine: The Case of School Children in Central Ethiopia | 0 | 1 | 1 | 0 | 1 | 1 | 1 | 1 | 1 | 1 | 8 | |
| Wang-2014 | Vitamin D Binding Protein Affects the Correlation Of 25(Oh)D and Frailty in the Older Men | 0 | 0 | 0 | 0 | 1 | 1 | 1 | 1 | 1 | 1 | 6 | |
| Wang-2015 | The Influence of Malnutrition and Micronutrient Status on Anemic Risk in Children Under 3 Years Old in Poor Areas in China | 0 | 1 | 1 | 0 | 1 | 1 | 1 | 1 | 1 | 1 | 8 | |
| Wang-2017 | Reference and Influential Factors of Serum Bone Markers in Chinese Adolescents | 0 | 0 | 1 | 0 | 1 | 1 | 1 | 1 | 1 | 1 | 7 | |
| Wang-2019 | Association Between Serum Vitamin B-6 Concentration and Risk of Osteoporosis in The Middle-Aged and Older People in China: A Cross-Sectional Study | 0 | 0 | 0 | 0 | 1 | 1 | 1 | 1 | 1 | 1 | 6 | |
| Wang-2019 | Association Between Vitamin D and Risk of Cardiovascular Disease in Chinese Rural Population | 0 | 0 | 0 | 0 | 1 | 1 | 1 | 1 | 1 | 1 | 6 | |
| Wang-2019 | Polymorphisms in Cyp2r1 Gene Associated with Serum Vitamin D Levels and Status in a Chinese Rural Population | 0 | 0 | 0 | 0 | 1 | 1 | 1 | 1 | 1 | 1 | 6 | |
| Wang-2022 | Gut Microbiome Signature Are Correlated with Bone Mineral Density Alterations in the Chinese Elders | 0 | 0 | 0 | 0 | 1 | 1 | 1 | 1 | 1 | 1 | 6 | |
| Warensjo Lemming-2022 | Vitamin D Status and Associations with Diet, Objectively Measured Physical Activity Patterns and Background Characteristics Among Adolescents in a Representative National Cross-Sectional Survey | 1 | 1 | 1 | 0 | 1 | 1 | 1 | 1 | 1 | 1 | 9 | |
| Weiler-2023 | Vitamin D Status of People 3 To 79 Years of Age from the Canadian Health Measures Survey 2012 To 2019 | 1 | 1 | 1 | 0 | 1 | 1 | 1 | 1 | 1 | 1 | 9 | |
| Weldegiorgis-2020 | Association Between Serum 25-hydroxyvitamin D Concentrations and Metabolic Syndrome in The Middle-Aged and Elderly Chinese Population in Dalian, Northeast China: A Cross-Sectional Study | 0 | 0 | 0 | 0 | 1 | 1 | 1 | 1 | 1 | 1 | 6 | |
| Wolters-2022 | 25-hydroxyvitamin D Reference Percentiles and the Role of their Determinants Among European Children and Adolescents | 1 | 1 | 1 | 1 | 1 | 1 | 1 | 1 | 1 | 1 | 10 | |
| Wyskida-2018 | Socio-Economic Determinants of Vitamin D Deficiency in the Older Polish Population: Results from the PolSenior Study | 1 | 1 | 1 | 1 | 1 | 1 | 1 | 1 | 1 | 1 | 10 | |
| Xiao-2020 | Adequate 25-hydroxyvitamin D Levels Are Inversely Associated with Various Cardiometabolic Risk Factors in Chinese Children, Especially Obese Children | 1 | 1 | 1 | 1 | 1 | 1 | 1 | 1 | 1 | 1 | 10 | |
| Xiao-2022 | Vitamin D Trajectories and Cardiometabolic Risk Factors During Childhood: A Large Population-Based Prospective Cohort Study | 0 | 1 | 1 | 1 | 1 | 1 | 1 | 1 | 1 | 1 | 9 | |
| Xie-2019 | Prevalence of Vitamin D Inadequacy Among Chinese Postmenopausal Women: A Nationwide, Multicenter, Cross-Sectional Study | 1 | 1 | 1 | 1 | 1 | 1 | 1 | 1 | 1 | 1 | 10 | |
| Xu-2020 | Sclerostin and its Associations with Bone Metabolism Markers and Sex Hormones in Healthy Community-Dwelling Elderly Individuals and Adolescents | 0 | 0 | 0 | 0 | 1 | 1 | 1 | 1 | 1 | 1 | 6 | |
| Yan-2018 | Association of Serum Uric Acid Levels with Osteoporosis and Bone Turnover Markers in a Chinese Population | 0 | 0 | 0 | 0 | 1 | 1 | 1 | 1 | 1 | 1 | 6 | |
| Yan-2019 | Gender Differences in Vitamin D Status in China | 0 | 0 | 1 | 0 | 1 | 1 | 1 | 1 | 1 | 1 | 7 | |
| Yang-2022 | Association of Serum Vitamin D and Estradiol Levels with Metabolic Syndrome in Rural Women of Northwest China: A Cross-Sectional Study | 0 | 1 | 1 | 0 | 1 | 1 | 1 | 1 | 1 | 1 | 8 | |
| Yao-2014 | Suboptimal Vitamin D Status in a Population-Based Study of Asian Children: Prevalence and Relation to Allergic Diseases and Atopy | 0 | 1 | 1 | 1 | 1 | 1 | 1 | 1 | 1 | 1 | 9 | |
| Yao-2019 | Sex, Residence and Fish Intake Predict Vitamin D Status in Chinese Centenarians | 0 | 0 | 0 | 0 | 1 | 1 | 1 | 1 | 1 | 1 | 6 | |
| Yeap-2021 | Asymptomatic Morphometric Vertebral Fractures and its Associated Factors: A Cross-Sectional Study Among Adults in a Selected Urban Area in Selangor, Malaysia | 0 | 0 | 0 | 1 | 1 | 1 | 1 | 1 | 1 | 1 | 7 | |
| Yoshihara-2014 | Association Between Low Renal Function and Tooth Loss Over 5 Years | 0 | 1 | 0 | 0 | 1 | 1 | 0 | 1 | 1 | 1 | 6 | |
| Yoshimura-2013 | Profiles of Vitamin D Insufficiency and Deficiency in Japanese Men and Women: Association with Biological, Environmental, And Nutritional Factors and Coexisting Disorders: The Road Study | 0 | 0 | 0 | 0 | 1 | 1 | 1 | 1 | 1 | 1 | 6 | |
| Younes-2019 | An Epidemiological Evaluation of Fractures and its Determinants Among Lebanese Schoolchildren: A Cross-Sectional Study | 0 | 1 | 0 | 0 | 1 | 0 | 1 | 1 | 1 | 1 | 6 | |
| Young-2023 | Assessment of Vitamin D Status and Association with Inflammation: Biomarkers Reflecting Inflammation and Nutritional Determinants of Anemia (BRINDA) Project | 1 | 1 | 1 | 0 | 1 | 1 | 1 | 1 | 1 | 1 | 9 | |
| Yousef-2017 | Associations Factors Affecting on Osteoporosis in Postmenopausal Women in Saudi Arabian, Jeddah | 0 | 0 | 0 | 0 | 1 | 1 | 0 | 1 | 1 | 1 | 5 | |
| Yuan-2023 | Effects of Obesity with Reduced 25(Oh)D Levels on Bone Health in Elderly Chinese People: A Nationwide Cross-Sectional Study | 0 | 0 | 0 | 0 | 1 | 0 | 1 | 1 | 0 | 1 | 4 | |
| Yunara-2020 | Association Of 25-Hydroxyvitamin D Concentration and Frailty Degree in Elderly Community in Surabaya | 0 | 1 | 1 | 0 | 1 | 1 | 1 | 1 | 1 | 1 | 8 | |
| Zamoiski-2014 | Association of Arsenic and Metals with Concentrations Of 25-Hydroxyvitamin D And 1,25-dihydroxyvitamin D Among Adolescents in Torreon, Mexico | 0 | 1 | 1 | 0 | 1 | 1 | 1 | 1 | 1 | 1 | 8 | |
| Zelzer-2021 | Association of Vitamin D Metabolites with Cognitive Function and Brain Atrophy in Elderly Individuals - The Austrian Stroke Prevention Study | 0 | 1 | 1 | 0 | 1 | 1 | 1 | 1 | 1 | 1 | 8 | |
| Zhang-2014 | Vitamin D Status and its Association with Adiposity and Oxidative Stress in Schoolchildren | 0 | 1 | 1 | 0 | 1 | 1 | 1 | 1 | 1 | 1 | 8 | |
| Zhang-2016 | Vitamin D Deficiency is Associated with High Prevalence of Diabetes in Kuwaiti Adults: Results from a National Survey | 1 | 1 | 1 | 0 | 1 | 1 | 1 | 1 | 1 | 1 | 9 | |
| Zhang-2021 | Serum 25-hydroxyvitamin D Concentrations and Cardiometabolic Biomarkers in Chinese Rural Population | 0 | 1 | 1 | 0 | 1 | 1 | 1 | 1 | 1 | 1 | 8 | |
| Zhang-2021 | The Nutrition and Health in Southwest China (NHSC) Study: Design, Implementation, And Major Findings | 0 | 1 | 1 | 1 | 1 | 1 | 0 | 1 | 1 | 1 | 8 | |
| Zhao-2022 | 25-hydroxyvitamin D and Incidence of Type 2 Diabetes from A Chinese Cohort Study | 0 | 1 | 1 | 0 | 1 | 1 | 1 | 1 | 1 | 1 | 8 | |
| Zheng-2018 | The Relationship Between Vitamin D and Type 2 Diabetes Is Intriguing: Glimpses from The SPECT-China Study | 0 | 1 | 1 | 0 | 1 | 1 | 1 | 1 | 1 | 1 | 8 | |
| Zhou-2015 | Vitamin D Status and its Predictors Among Pre-School Children in Adelaide | 0 | 1 | 1 | 0 | 1 | 1 | 1 | 1 | 1 | 1 | 8 | |
| Zhou-2022 | Chlorpyrifos Residue Level and ADHD Among Children Aged 1-6 Years in Rural China: A Cross-Sectional Study | 0 | 0 | 0 | 0 | 1 | 1 | 1 | 1 | 1 | 0 | 5 | |
| Zhu-2018 | Associations of Vitamin D Status with Markers of Metabolic Health: A Community-Based Study in Shanghai, China | 0 | 1 | 1 | 0 | 1 | 1 | 1 | 1 | 1 | 1 | 8 | |
| Zhuang-2021 | Metabolic Signatures of Genetically Elevated Vitamin D Among Chinese: Observational and Mendelian Randomization Study | 0 | 0 | 0 | 0 | 1 | 1 | 1 | 1 | 1 | 1 | 6 | |
| Zou-2021 | Evaluation of Bone Metabolism-Associated Biomarkers in Tibet, China | 0 | 1 | 1 | 0 | 1 | 1 | 1 | 1 | 1 | 1 | 7 | |
| Zupo-2023 | A Machine-Learning Approach to Target Clinical and Biological Features Associated with Sarcopenia: Findings from Northern and Southern Italian Aging Populations | 0 | 0 | 0 | 0 | 1 | 1 | 1 | 1 | 0 | 1 | 5 | |

^a^ Evaluated using a tool developed by Hoy and colleagues ^587^

^b^ Items: 1) Was the study’s target population **a close representation** of the national population in relation to relevant variables, e.g., age, sex, occupation? 2) Was the sampling frame a **true or close representation** of the target population? 3) Was some form of **random selection** used to select the sample, OR, was a census undertaken? 4) Was the likelihood of **non-response bias minimal**? 5) Were data collected **directly from the subjects** as opposed to a proxy? 6) Was an acceptable case definition used in the study? 7) Was the study instrument that measured the parameter of interest shown to have **reliability and validity (if necessary)**? 8) Was the **same mode of data collection** used for all subjects 9) Was the **length of the shortest prevalence period** for the parameter of interest appropriate? 10) Were the **numerator(s) and denominator(s)** for the parameter of interest appropriate? ^587^

# Supplementary Table 4: Pooled mean circulating 25-hydroxyvitamin D concentration by country

| Country | n (studies) | n  (participants) | Mean 25(OH)D (nmol/L) | Lower 95% CI | Upper 95% CI |
| --- | --- | --- | --- | --- | --- |
| Afghanistan | 1 | 1,706 | 25.77 | 9.21 | 42.34 |
| Algeria | 4 | 2,646 | 47.27 | 34.79 | 59.75 |
| Argentina | 2 | 530 | 45.60 | 27.37 | 63.82 |
| Armenia | 1 | 1,206 | 49.92 | 48.79 | 51.05 |
| Australia | 18 | 18,021 | 65.90 | 58.29 | 73.51 |
| Austria | 4 | 2,029 | 46.25 | 24.48 | 68.02 |
| Bangladesh | 3 | 638 | 53.27 | 46.22 | 60.31 |
| Belgium | 2 | 1,286 | 46.28 | 31.94 | 60.62 |
| Bolivia | 1 | 203 | 68.72 | 68.28 | 69.16 |
| Brazil | 15 | 8,414 | 63.01 | 56.44 | 69.57 |
| Bulgaria | 1 | 2,016 | 38.75 | 38.00 | 39.50 |
| Cambodia | 2 | 2,070 | 74.65 | 60.15 | 89.14 |
| Cameroon | 1 | 586 | 51.70 | 50.69 | 52.71 |
| Canada | 7 | 26,497 | 54.47 | 40.08 | 68.86 |
| Chile | 2 | 7,247 | 42.13 | 28.96 | 55.31 |
| China | 67 | 758,955 | 51.67 | 47.93 | 55.40 |
| Colombia | 2 | 39,486 | 69.37 | 61.94 | 76.81 |
| Croatia | 1 | 260 | 46.55 | 44.10 | 49.00 |
| Czech Republic | 1 | 1,023 | 52.60 | 51.54 | 53.66 |
| Denmark | 7 | 33,468 | 54.76 | 45.73 | 63.80 |
| Ecuador | 1 | 2,374 | 64.43 | 63.38 | 65.48 |
| Egypt | 4 | 1,572 | 53.50 | 20.30 | 86.70 |
| Estonia | 2 | 338 | 37.96 | 26.20 | 49.72 |
| Ethiopia | 3 | 1,003 | 58.09 | 34.95 | 81.23 |
| Faroe Islands | 1 | 668 | 47.40 | 45.43 | 49.37 |
| Finland | 9 | 12,305 | 62.79 | 57.15 | 68.44 |
| France | 2 | 945 | 62.86 | 55.19 | 70.54 |
| Gambia | 2 | 623 | 63.07 | 56.24 | 69.90 |
| Germany | 10 | 43,100 | 52.05 | 44.56 | 59.55 |
| Ghana | 1 | 479 | 75.88 | 74.34 | 77.42 |
| Greece | 4 | 4,024 | 48.84 | 43.98 | 53.69 |
| Greenland | 3 | 3,589 | 51.18 | 39.93 | 62.44 |
| Hungary | 3 | 1,332 | 42.06 | 9.68 | 74.44 |
| Iceland | 4 | 11,082 | 51.07 | 44.20 | 57.94 |
| India | 39 | 73,229 | 51.70 | 43.72 | 59.69 |
| Indonesia | 9 | 1,124 | 45.08 | 35.22 | 54.94 |
| Iran | 33 | 27,541 | 48.39 | 40.54 | 56.25 |
| Iraq | 1 | 300 | 45.19 | 43.55 | 46.84 |
| Ireland | 4 | 7,475 | 55.37 | 48.00 | 62.74 |
| Israel | 1 | 116 | 27.96 | 26.18 | 29.73 |
| Italy | 7 | 4,138 | 52.54 | 42.79 | 62.29 |
| Jamaica | 1 | 448 | 72.13 | 70.47 | 73.80 |
| Japan | 24 | 23,128 | 49.62 | 44.76 | 54.49 |
| Jordan | 5 | 11,791 | 52.61 | 23.40 | 81.83 |
| Kazakhstan | 1 | 110 | 51.17 | 48.14 | 54.20 |
| Kenya | 1 | 500 | 85.72 | 85.43 | 86.00 |
| Korea South | 20 | 29,734 | 49.59 | 41.72 | 57.47 |
| Kuwait | 3 | 2,608 | 28.32 | 21.56 | 35.07 |
| Lebanon | 5 | 3,553 | 44.40 | 39.67 | 49.12 |
| Luxembourg | 1 | 1,404 | 61.40 | 59.52 | 63.28 |
| Malaysia | 8 | 5,048 | 58.74 | 49.13 | 68.34 |
| Mexico | 7 | 7,697 | 64.92 | 56.86 | 72.98 |
| Morocco | 1 | 254 | 50.84 | 48.23 | 53.45 |
| Nepal | 1 | 181 | 66.57 | 61.54 | 71.60 |
| Netherlands | 7 | 13,597 | 61.84 | 53.32 | 70.36 |
| New Zealand | 6 | 3,332 | 68.98 | 58.85 | 79.10 |
| Nine Mesoamerican Countries ^a^ | 1 | 223 | 79.50 | 77.12 | 81.88 |
| Norway | 6 | 26,820 | 51.46 | 41.73 | 61.18 |
| Pakistan | 9 | 25,851 | 45.23 | 38.29 | 52.17 |
| Palestine | 2 | 319 | 59.56 | 36.09 | 83.04 |
| Peru | 1 | 1,134 | 63.65 | 62.36 | 64.94 |
| Philippines | 1 | 789 | 70.24 | 69.00 | 71.47 |
| Poland | 12 | 6,089 | 52.72 | 48.81 | 56.62 |
| Portugal | 6 | 6,445 | 47.91 | 37.41 | 58.42 |
| Qatar | 1 | 1,205 | 46.25 | 44.91 | 47.60 |
| Russia | 2 | 410 | 43.40 | 24.83 | 61.98 |
| Saudi Arabia | 19 | 28,215 | 39.08 | 31.06 | 47.10 |
| Serbia | 1 | 170 | 47.99 | 45.19 | 50.78 |
| Seychelles | 1 | 438 | 72.63 | 70.83 | 74.43 |
| Singapore | 2 | 695 | 70.98 | 61.24 | 80.72 |
| Slovenia | 1 | 280 | 48.58 | 47.83 | 49.32 |
| South Africa | 6 | 4,085 | 68.06 | 55.59 | 80.53 |
| Spain | 14 | 4,191 | 57.56 | 48.24 | 66.87 |
| Sri Lanka | 3 | 3,215 | 41.80 | 18.31 | 65.29 |
| Sweden | 5 | 6,948 | 56.12 | 44.54 | 67.70 |
| Switzerland | 3 | 8,513 | 63.72 | 49.13 | 78.31 |
| Taiwan | 4 | 9,940 | 60.41 | 51.82 | 69.01 |
| Tanzania | 1 | 88 | 106.80 | 100.87 | 112.73 |
| Thailand | 7 | 5,065 | 77.93 | 57.96 | 97.91 |
| Turkey | 9 | 3,831 | 59.98 | 41.51 | 78.45 |
| United Kingdom ^b^ | 7 | 12,338 | 50.56 | 38.98 | 62.15 |
| United States of America | 21 | 16,375 | 65.87 | 58.20 | 73.55 |
| Ukraine | 2 | 1,232 | 62.12 | 53.41 | 70.83 |
| United Arab Emirates | 4 | 1,205 | 39.21 | 21.00 | 57.42 |
| Vietnam | 6 | 3,588 | 57.13 | 41.46 | 72.79 |

25(OH)D, 25-hydroxyvitamin D; CI, confidence interval

^a^ Guatemala, El Salvador, the Dominican Republic, Honduras, Nicaragua, Costa Rica, Panama, Belize, and Mexico

^b^ England, Scotland, Wales and Northern Ireland

# Supplementary Table 5: Pooled mean circulating 25-hydroxyvitamin D concentration by latitude, season, use of a certified assay/standardised data, study quality and country income classification

| Characteristics | n (*publications*) | n *(data points)* | n *(countries)* | n *(participants)* | Mean 25(OH)D (nmol/L) | 95% CI | *I^2^ (%)* |
| --- | --- | --- | --- | --- | --- | --- | --- |
| Latitude |  |  |  |  |  |  |  |
| Low (<40^o^) | 393 | 415 | 55 | 1,174,090 | 53.65 | 52.26; 55.03 | 100.0 |
| High (≥40^o^) | 137 | 141 | 32 | 238,191 | 54.57 | 52.14; 57.00 | 100.0 |
| Season |  |  |  |  |  |  |  |
| Winter/Spring | 68 | 69 | 36 | 37,798 | 48.78 | 46.25; 51.31 | 99.9 |
| Summer/Autumn | 65 | 65 | 35 | 34,937 | 57.30 | 55.51; 59.10 | 99.8 |
| Use of a certified assay or harmonised data^a^ | | |  |  |  |  |  |
| Yes | 68 | 72 | 34 | 720,697 | 52.93 | 49.24; 56.62 | 100.0 |
| No | 461 | 478 | 82 | 691,584 | 54.05 | 52.55; 55.77 | 100.0 |
| Study quality^b^ |  |  |  |  |  |  |  |
| Low | 30 | 30 | 21 | 23,328 | 54.99 | 50.97; 59.00 | 99.8 |
| Moderate | 419 | 436 | 78 | 1,055,514 | 54.21 | 52.77; 55.66 | 100.0 |
| High | 82 | 90 | 40 | 335,439 | 51.98 | 47.73; 56.24 | 100.0 |
| Country classification by income^d^ | | |  |  |  |  |  |
| High-income | 258 | 264 | 41 | 387,812 | 53.47 | 52.07; 54.88 | 100.0 |
| Upper-middle-income | 139 | 142 | 19 | 842,403 | 56.22 | 53.07; 59.37 | 100.0 |
| Lower-middle-income | 122 | 134 | 20 | 160,731 | 52.06 | 48.02; 56.10 | 100.0 |
| Low-income | 6 | 7 | 3 | 3,332 | 50.28 | 32.41; 68.15 | 99.9 |

25(OH)D, 25-hydroxyvitamin D; CI, confidence interval

^a^ Use of an assay that was certified to the reference measurement procedures (RMPs) developed under the Vitamin D Standardization Program (VDSP), or data that were retrospectively harmonised to a certified assay ^b^ Evaluated using a tool developed by Hoy and colleagues^587^: a low (>8), moderate (6–8), or high (≤5) risk of bias on the basis of the overall score; ^c^ For longitudinal cohort studies, the most recently collected data were extracted; ^d^ The World Bank classification by income

# Supplementary Table 6: Prevalence of circulating 25-hydroxyvitamin D concentration <30, <50 and <75 nmol/L by region and sex

|  | | | n (*publications*) | | n  (*data points*) | | | n *(countries)* | | | n  *(participants)* | | *n (participants)* | | | | Prevalence (%) | 95% CI | | | | *I^2^ (%)* | |  |
| --- | --- | --- | --- | --- | --- | --- | --- | --- | --- | --- | --- | --- | --- | --- | --- | --- | --- | --- | --- | --- | --- | --- | --- | --- |
| *<30 nmol/L* | | | | | | | | | | | | | | | | | | | | | | | |  |
| All regions | | | 76 | | 89 | | | 78 | | | 365,326 | | 58,834 | | | | 18.41 | 16.40; 20.43 | | | | 99.8 | |  |
| Male | | | 33 | | 35 | | | 22 | | | 85,091 | | 12,680 | | | | 14.43 | 11.52, 17.33 | | | | 99.6 | |  |
| Female | | | 32 | | 33 | | | 23 | | | 89,632 | | 18,656 | | | | 21.19 | 17.21; 25.18 | | | | 99.7 | |  |
| Africa | | | 7 | | 8 | | | 6 | | | 5,070 | | 160 | | | | 3.58 | 2.25; 4.90 | | | | 95.2 | |  |
| Male | | | 1 | | 1 | | | 1 | | | 220 | | 5 | | | | 2.20 | 0.26; 4.14 | | | | - | |  |
| Female | | | 1 | | 1 | | | 1 | | | 330 | | 24 | | | | 7.30 | 4.49; 10.11 | | | | - | |  |
| Asia | | | 27 | | 36 | | | 17 | | | 204,029 | | 47,376 | | | | 27.34 | 23.95; 31.73 | | | | 99.7 | |  |
| Male | | | 15 | | 15 | | | 10 | | | 53,307 | | 9,330 | | | | 18.16 | 14.81; 21.52 | | | | 99.2 | |  |
| Female | | | 17 | | 17 | | | 12 | | | 57,643 | | 15,090 | | | | 25.94 | 19.29; 32.59 | | | | 99.7 | |  |
| Europe | | | 26 | | 28 | | | 18 | | | 77,507 | | 7,378 | | | | 14.24 | 12.05; 16.43 | | | | 99.5 | |  |
| Male | | | 13 | | 15 | | | 9 | | | 15,549 | | 2,037 | | | | 13.75 | 9.24; 18.25 | | | | 99.3 | |  |
| Female | | | 11 | | 12 | | | 8 | | | 16,798 | | 2,570 | | | | 19.85 | 14.30; 25.40 | | | | 99.3 | |  |
| North America | | | 9 | | 9 | | | 11 | | | 27,655 | | 2,333 | | | | 20.05 | 12.96; 27.13 | | | | 99.8 | |  |
| Male | | | 1 | | 1 | | | 1 | | | 10,925 | | 1,038 | | | | 9.50 | 8.95; 10.05 | | | | - | |  |
| Female | | | 1 | | 1 | | | 1 | | | 10,845 | | 792 | | | | 7.30 | 6.81; 7.79 | | | | - | |  |
| South America | | | 2 | | 3 | | | 2 | | | 41,275 | | 1,087 | | | | 2.61 | 1.67; 3.55 | | | | 94.9 | |  |
| Male | | | 0 | | 0 | | | - | | | 0 | | 0 | | | | - | - | | | | - | |  |
| Female | | | 0 | | 0 | | | - | | | 0 | | 0 | | | | - | - | | | | - | |  |
| Oceania | | | 5 | | 5 | | | 1 | | | 9,790 | | 499 | | | | 5.27 | 3.43; 7.11 | | | | 93.6 | |  |
| Male | | | 3 | | 3 | | | 1 | | | 5,090 | | 745 | | | | 4.16 | 1.38; 6.95 | | | | 96.6 | |  |
| Female | | | 2 | | 2 | | | 1 | | | 4,016 | | 179 | | | | 4.24 | 2.87; 5.61 | | | | 77.6 | |  |
| *<50 nmol/L* | | | | | | | | | | | | | | | | | | | | | | | |  |
| All regions | 361 | | | 384 | | | 88 | | | 1,449,197 | | | | | 763,636 | | 47.36 | | | 44.66; 50.05 | | 99.9 | | |
| Male | 185 | | | 192 | | | 57 | | | 495,004 | | | | | 261,262 | | 45.40 | | | 41.80; 49.00 | | 99.9 | | |
| Female | 174 | | | 181 | | | 55 | | | 601,960 | | | | | 343,876 | | 53.34 | | | 49.66; 57.02 | | 99.9 | | |
| Africa | 17 | | | 18 | | | 8 | | | 10,788 | | | | | 2,402 | | 29.60 | | | 15.89; 43.30 | | 99.8 | | |
| Male | 8 | | | 9 | | | 5 | | | 1,940 | | | | | 629 | | 32.88 | | | 11.84; 53.93 | | 99.7 | | |
| Female | 6 | | | 7 | | | 4 | | | 1,825 | | | | | 559 | | 32.24 | | | 12.28; 52.19 | | 99.5 | | |
| Asia | 188 | | | 203 | | | 31 | | | 648,433 | | | | | 402,033 | | 55.37 | | | 51.88; 58.86 | | 99.9 | | |
| Male | 106 | | | 107 | | | 21 | | | 191,948 | | | | | 116,095 | | 52.00 | | | 46.76; 57.24 | | 99.9 | | |
| Female | 105 | | | 106 | | | 22 | | | 272,114 | | | | | 179,993 | | 62.96 | | | 57.17; 66.75 | | 99.9 | | |
| Europe | 92 | | | 97 | | | 31 | | | 619,471 | | | | | 318,218 | | 46.65 | | | 42.83; 50.47 | | 99.8 | | |
| Male | 43 | | | 47 | | | 21 | | | 248,070 | | | | | 132,997 | | 44.33 | | | 36.59; 51.07 | | 99.8 | | |
| Female | 36 | | | 40 | | | 20 | | | 275,578 | | | | | 149,830 | | 50.39 | | | 44.47; 56.31 | | 99.7 | | |
| North America | 26 | | | 26 | | | 11 | | | 92,328 | | | | | 22,517 | | 32.59 | | | 23.09; 42.10 | | 99.9 | | |
| Male | 10 | | | 11 | | | 4 | | | 40,142 | | | | | 15,804 | | 19.82 | | | 15.23; 24.41 | | 99.4 | | |
| Female | 12 | | | 13 | | | 4 | | | 42,713 | | | | | 10,739 | | 26.53 | | | 22.36; 30.70 | | 98.6 | | |
| South America | 20 | | | 21 | | | 5 | | | 53,801 | | | | | 13,250 | | 28.46 | | | 20.59; 36.33 | | 99.8 | | |
| Male | 8 | | | 8 | | | 4 | | | 3,285 | | | | | 742 | | 33.22 | | | 18.22; 48.22 | | 99.2 | | |
| Female | 8 | | | 8 | | | 3 | | | 4,140 | | | | | 1,229 | | 33.84 | | | 19.77; 47.91 | | 99.1 | | |
| Oceania | 18 | | | 19 | | | 2 | | | 24,376 | | | | | 5,217 | | 23.50 | | | 18.03; 28.96 | | 99.1 | | |
| Male | 10 | | | 10 | | | 2 | | | 9.619 | | | | | 4,828 | | 25.24 | | | 17.74; 32.74 | | 98.9 | | |
| Female | 7 | | | 7 | | | 2 | | | 5,590 | | | | | 1,526 | | 30.14 | | | 20.61; 39.67 | | 98.2 | | |
| *<75 nmol/L* | | | | | | | | | | | | | | | | | | | | | | |  |  |
| All regions | | 241 | | | | 250 | | | 79 | | | 559,665 | | 447,878 | | 74.73 | | | 73.05; 76.42 | | 99.8 | |  |  |
| Male | | 114 | | | | 117 | | | 42 | | | 124,674 | | 91,147 | | 72.81 | | | 69.23; 76.39 | | 99.8 | |  |  |
| Female | | 106 | | | | 109 | | | 37 | | | 144,797 | | 114,040 | | 79.79 | | | 77.13; 82.44 | | 99.7 | |  |  |
| Africa | | 12 | | | | 13 | | | 7 | | | 9,381 | | 5,170 | | 66.37 | | | 52.86; 79.89 | | 99.7 | |  |  |
| Male | | 2 | | | | 2 | | | 2 | | | 455 | | 443 | | 97.46 | | | 96.02; 98.91 | | - | |  |  |
| Female | | 1 | | | | 1 | | | 1 | | | 266 | | 250 | | 94.00 | | | 91.15; 96.85 | | - | |  |  |
| Asia | | 129 | | | | 134 | | | 25 | | | 331,724 | | 289,917 | | 78.24 | | | 76.31; 80.17 | | 99.8 | |  |  |
| Male | | 72 | | | | 72 | | | 19 | | | 56,035 | | 43,798 | | 74.03 | | | 68.80; 79.26 | | 99.8 | |  |  |
| Female | | 73 | | | | 73 | | | 20 | | | 82,273 | | 68,216 | | 81.67 | | | 78.87; 84.48 | | 99.7 | |  |  |
| Europe | | 55 | | | | 57 | | | 28 | | | 99,523 | | 76,156 | | 77.05 | | | 74.03; 80.07 | | 99.5 | |  |  |
| Male | | 20 | | | | 22 | | | 13 | | | 17,987 | | 13,694 | | 72.01 | | | 66.00; 78.03 | | 99.2 | |  |  |
| Female | | 14 | | | | 16 | | | 8 | | | 13,112 | | 11,066 | | 78.77 | | | 73.99; 83.54 | | 98.9 | |  |  |
| North America | | 18 | | | | 18 | | | 11 | | | 84,386 | | 55,348 | | 62.72 | | | 56.51; 68.92 | | 99.2 | |  |  |
| Male | | 7 | | | | 8 | | | 3 | | | 39,072 | | 25,935 | | 70.51 | | | 68.04; 72.98 | | 96.8 | |  |  |
| Female | | 8 | | | | 9 | | | 3 | | | 41,586 | | 27,035 | | 72.97 | | | 67.42; 78.52 | | 99.0 | |  |  |
| South America | | 17 | | | | 17 | | | 6 | | | 13,337 | | 8,363 | | 62.80 | | | 50.97; 74.64 | | 99.8 | |  |  |
| Male | | 6 | | | | 6 | | | 3 | | | 2,043 | | 1,055 | | 56.13 | | | 47.10; 65.16 | | 92.0 | |  |  |
| Female | | 6 | | | | 6 | | | 3 | | | 2,626 | | 1,833 | | 72.81 | | | 66.88; 78.74 | | 87.2 | |  |  |
| Oceania | | 10 | | | | 11 | | | 2 | | | 21,314 | | 12,924 | | 68.93 | | | 59.71; 78.15 | | 99.5 | |  |  |
| Male | | 7 | | | | 7 | | | 2 | | | 9,082 | | 6,222 | | 70.86 | | | 61.86; 79.85 | | 99.3 | |  |  |
| Female | | 4 | | | | 4 | | | 2 | | | 4,934 | | 3,386 | | 73,47 | | | 58.24; 88.71 | | 99.3 | |  |  |

25(OH)D, 25-hydroxyvitamin D; CI, confidence interval

# Supplementary Table 7: Prevalence of circulating 25-hydroxyvitamin D concentration <30, <50 and <75 nmol/L by region and adults and children

|  | n  (*studies*) | n  (*data points*) | *n*  *(countries)* | n  *(participants)* | *n*  *(participants)* | Prevalence  (%) | 95% CI | *I^2^ (%)* |
| --- | --- | --- | --- | --- | --- | --- | --- | --- |
| *<30 nmol/L* | | | | | | | | |
| All regions |  |  |  |  |  |  |  |  |
| Adults | 20 | 20 | 21 | 66,710 | 5,391 | 13.60 | 11.45; 15.76 | 99.4 |
| Children | 12 | 12 | 12 | 128,133 | 20,363 | 12.18 | 6.17; 18.19 | 99.9 |
| Africa |  |  |  |  |  |  |  |  |
| Adults | 0 | -- | -- | -- | -- | -- | -- | - |
| Children | 0 | -- | -- | -- | -- | -- | -- | - |
| Asia |  |  |  |  |  |  |  |  |
| Adults | 7 | 7 | 6 | 9,814 | 1,225 | 17.02 | 11.18; 22.86 | 99.2 |
| Children | 5 | 5 | 2 | 83,528 | 18,841 | 20.84 | 15.49; 26.18 | 99.4 |
| Europe |  |  |  |  |  |  |  |  |
| Adults | 9 | 9 | 12 | 29,259 | 2290 | 15.43 | 11.64; 19.22 | 99.6 |
| Children | 4 | 4 | 3 | 3,546 | 219 | 6.74 | 3.27; 10.21 | 95.4 |
| North America |  |  |  |  |  |  |  |  |
| Adults | 1 | 1 | 1 | 13,774 | 1,223 | 7.97 | 7.52, 8.42 | -- |
| Children | 1 | 1 | 1 | 8,997 | 510 | 3.61 | 3.22, 3.98 | -- |
| South America |  |  |  |  |  |  |  |  |
| Adults | 1 | 1 | 1 | 7,170 | 265 | 3.70 | 3.26, 4.14 | -- |
| Children | 1 | 1 | 1 | 31,841 | 784 | 2.40 | 2.23, 2.57 | -- |
| Oceania |  |  |  |  |  |  |  |  |
| Adults | 2 | 2 | 1 | 6,693 | 388 | 6.88 | 2.15; 11.61 | 97.5 |
| Children | 1 | 1 | 1 | 221 | 9 | 4.46 | 1.45, 7.46 | -- |

*<50 nmol/L*

| All regions |  |  |  |  |  |  |  |  |
| --- | --- | --- | --- | --- | --- | --- | --- | --- |
| Adults | 78 | 78 | 43 | 784,495 | 425,640 | 47.10 | 41.96, 52.24 | 99.9 |
| Children | 44 | 45 | 24 | 174,954 | 84,811 | 46.37 | 37.71, 55.04 | 99.9 |
| Africa |  |  |  |  |  |  |  |  |
| Adults | 2 | 2 | 2 | 790 | 385 | 42.80 | -35.31, 120.90 | 99.9 |
| Children | 3 | 3 | 3 | 2,569 | 752 | 45.69 | -13.07, 104.45 | 99.9 |
| Asia |  |  |  |  |  |  |  |  |
| Adults | 34 | 34 | 15 | 206,378 | 136.944 | 53.15 | 42.45, 63.85 | 100.0 |
| Children | 22 | 22 | 7 | 98,645 | 65,718 | 62.40 | 55.12, 69.67 | 99.8 |
| Europe |  |  |  |  |  |  |  |  |
| Adults | 27 | 27 | 19 | 510,623 | 271,413 | 49.24 | 41.45, 57.03 | 99,9 |
| Children | 10 | 11 | 8 | 9,165 | 3,991 | 35.45 | 24.05, 46.85 | 99.3 |
| North America |  |  |  |  |  |  |  |  |
| Adults | 6 | 6 | 3 | 48,401 | 12206 | 24.27 | 17.46, 31.08 | 98.6 |
| Children | 1 | 1 | 1 | 27,224 | 5,111 | 15.32 | 14.90, 15.73 | - |
| South America |  |  |  |  | , |  |  |  |
| Adults | 4 | 4 | 2 | 8,201 | 2,198 | 41.08 | 12.55, 69.62 | 99.7 |
| Children | 4 | 4 | 3 | 34,602 | 8,293 | 17.42 | 11.15, 23.68 | 98.3 |
| Oceania |  |  |  |  |  |  |  |  |
| Adults | 5 | 5 | 2 | 10,102 | 2,494 | 27.91 | 19.35, 36.46 | 98.8 |
| Children | 4 | 4 | 2 | 2,749 | 946 | 25.38 | 7.22, 43.53 | 99.3 |
| *<75 nmol/L* | | | | | | | | |
| All regions |  |  |  |  |  |  |  |  |
| Adults | 51 | 51 | 32 | 168,680 | 122,258 | 75.63 | 70.69, 80.58 | 99.9 |
| Children | 22 | 22 | 17 | 49,990 | 36,387 | 78.09 | 71.73, 84.45 | 99.8 |
| Africa |  |  |  |  |  |  |  |  |
| Adults | 1 | 1 | 1 | 451 | 429 | 95.56 | 93.67, 97.46 | -- |
| Children | 1 | 1 | 1 | 572 | 566 | 97.78 | 96.02, 99.54 | -- |
| Asia |  |  |  |  |  |  |  |  |
| Adults | 26 | 26 | 12 | 68,664 | 53,586 | 76.73 | 69.97, 83.48 | 99,9 |
| Children | 8 | 8 | 5 | 8,715 | 6,942 | 83.85 | 74.94, 92.77 | 99.6 |
| Europe |  |  |  |  |  |  |  |  |
| Adults | 14 | 14 | 15 | 41,866 | 31,103 | 78.82 | 72.41, 85.24 | 99.7 |
| Children | 5 | 5 | 5 | 7,970 | 6,813 | 78.33 | 68.50, 88.16 | 99.5 |
| North America |  |  |  |  |  |  |  |  |
| Adults | 5 | 5 | 2 | 47,601 | 30,340 | 61.15 | 57.23, 65.07 | 90.6 |
| Children | 1 | 1 | 1 | 27,224 | 18,088 | 67.32 | 66.77, 67.87 | -- |
| South America |  |  |  |  |  |  |  |  |
| Adults | 1 | 1 | 1 | 288 | 259 | 90.52 | 82.27, 98.77 | -- |
| Children | 4 | 4 | 3 | 2,981 | 1,996 | 66.65 | 51.60, 81.71 | 98.9 |
| Oceania |  |  |  |  |  |  |  |  |
| Adults | 4 | 4 | 1 | 9,810 | 6,541 | 69.25 | 59.90, 78.60 | 98.9 |
| Children | 3 | 3 | 2 | 2,528 | 1,982 | 74.64 | 57.03, 92.25 | 99.0 |

25(OH)D, 25-hydroxyvitamin D; CI, confidence interval

# Supplementary Table 8: Pooled prevalence according to commonly reported thresholds by country

| Country | Publications | Participants | Prevalence | 95% CI | |
| --- | --- | --- | --- | --- | --- |
| *<30 nmol/L* | | | | |  |
| Afghanistan | 1 | 2405 | 43.93 | -0.63; 88.50 | |
| Algeria | 1 | 435 | 8.10 | 5.54; 10.66 | |
| Argentina | 0 |  |  | 0.00; 0.00 | |
| Armenia | 1 | 1206 | 12.93 | 11.05; 14.81 | |
| Australia | 5 | 9790 | 5.34 | 3.05; 7.64 | |
| Austria | 0 |  |  | 0.00; 0.00 | |
| Bangladesh | 0 |  |  | 0.00; 0.00 | |
| Belgium | 0 |  |  | 0.00; 0.00 | |
| Brazil | 1 | 2264 | 1.70 | 1.17; 2.23 | |
| Bulgaria | 0 |  |  | 0.00; 0.00 | |
| Cambodia | 1 | 961 | 12.36 | -4.30; 29.02 | |
| Cameroon | 1 | 586 | 4.40 | 2.32; 6.48 | |
| Canada | 4 | 23211 | 25.00 | -11.54; 61.54 | |
| Chile | 0 |  |  | 0.00; 0.00 | |
| China | 8 | 153680 | 22.56 | 15.89; 29.23 | |
| Colombia | 1 | 39011 | 3.03 | 1.76; 4.31 | |
| Croatia | 0 |  |  | 0.00; 0.00 | |
| Denmark | 0 |  |  | 0.00; 0.00 | |
| Ecuador | 0 |  |  | 0.00; 0.00 | |
| Egypt | 1 | 550 | 5.30 | 2.88; 7.72 | |
| Estonia | 0 |  |  | 0.00; 0.00 | |
| Ethiopia | 1 | 202 | 14.80 | 9.90; 19.70 | |
| Faroe Islands | 0 |  |  | 0.00; 0.00 | |
| Finland | 2 | 7701 | 1.54 | -0.33; 3.40 | |
| France | 1 | 892 | 9.89 | 7.93; 11.85 | |
| Germany | 3 | 21660 | 13.81 | 10.69; 16.93 | |
| Greece | 2 | 3437 | 16.94 | -6.19; 40.07 | |
| Greenland | 0 |  |  | 0.00; 0.00 | |
| Hungary | 1 | 882 | 30.00 | 26.51; 33.49 | |
| Iceland | 1 | 139 | 6.00 | 2.05; 9.95 | |
| India | 4 | 5188 | 47.21 | 29.66; 64.77 | |
| Indonesia | 0 |  |  | 0.00; 0.00 | |
| Iran | 3 | 11239 | 18.21 | 8.03; 28.38 | |
| Iraq | 1 | 300 | 59.30 | 53.74; 64.86 | |
| Ireland | 3 | 2119 | 10.10 | -0.76; 20.96 | |
| Italy | 0 |  |  | 0.00; 0.00 | |
| Japan | 0 |  |  | 0.00; 0.00 | |
| Jordan | 2 | 2947 | 40.06 | 0.37; 79.75 | |
| Kenya | 0 |  |  | 0.00; 0.00 | |
| Korea South | 0 |  |  | 0.00; 0.00 | |
| Kuwait | 1 | 960 | 36.25 | 33.21; 39.29 | |
| Lebanon | 1 | 466 | 39.10 | 34.67; 43.53 | |
| Malaysia | 1 | 400 | 12.00 | 8.82; 15.19 | |
| Nine Mesoamerican Countries |  |  |  | 0.00; 0.00 | |
| Mexico | 1 | 275 | 0.00 | 0.00; 0.00 | |
| Mongolia | 0 |  |  | 0.00; 0.00 | |
| Morocco | 0 |  |  | 0.00; 0.00 | |
| Nepal | 0 |  |  | 0.00; 0.00 | |
| Netherlands | 1 | 2716 | 12.40 | 11.16; 13.64 | |
| New Zealand | 0 |  |  | 0.00; 0.00 | |
| Norway | 2 | 24903 | 3.20 | 0.66; 5.75 | |
| Oman | 1 | 2553 | 13.40 | 7.91; 18.89 | |
| Pakistan | 1 | 15330 | 28.40 | 8.02; 48.78 | |
| Palestine | 0 |  |  | 0.00; 0.00 | |
| Peru | 0 |  |  | 0.00; 0.00 | |
| Philippines | 0 |  |  | 0.00; 0.00 | |
| Poland | 1 | 132 | 17.40 | 10.93; 23.87 | |
| Portugal | 2 | 2000 | 39.29 | 37.03; 41.56 | |
| Qatar | 0 |  |  | 0.00; 0.00 | |
| Russia | 1 | 90 | 34.40 | 24.59; 44.21 | |
| Saudi Arabia | 1 | 2226 | 19.40 | 17.15; 21.65 | |
| Singapore | 0 |  |  | 0.00; 0.00 | |
| Slovenia | 1 | 280 | 24.11 | 19.10; 29.12 | |
| South Africa | 2 | 1307 | 2.91 | -2.29; 8.10 | |
| Spain | 0 |  |  | 0.00; 0.00 | |
| Sri Lanka | 1 | 2525 | 13.20 | 11.88; 14.52 | |
| Sweden | 3 | 6576 | 5.63 | 3.25; 8.02 | |
| Switzerland | 0 |  |  | 0.00; 0.00 | |
| Syria | 0 |  |  | 0.00; 0.00 | |
| Taiwan | 0 |  |  | 0.00; 0.00 | |
| Tajikistan | 0 |  |  | 0.00; 0.00 | |
| Tanzania | 0 |  |  | 0.00; 0.00 | |
| Thailand | 0 |  |  | 0.00; 0.00 | |
| Turkey | 0 |  |  | 0.00; 0.00 | |
| UK | 2 | 3980 | 27.49 | 16.91; 38.08 | |
| USA | 3 | 3946 | 10.41 | -8.01; 28.83 | |
| Ukraine | 0 |  |  | 0.00; 0.00 | |
| United Arab Emirates | 0 |  |  | 0.00; 0.00 | |
| Vietnam | 2 | 1643 | 18.37 | 16.44; 20.29 | |
| *<50 nmol/L* | | | | |  |
| Afghanistan | 1 | 2405 | 68.55 |  |  |
| Algeria | 3 | 1787 | 53.71 | 20.87; 86.55 | |
| Argentina | 1 | 355 | 92.70 | 90.79; 94.61 | |
| Armenia | 0 |  |  | 0.00; 0.00 | |
| Australia | 14 | 21963 | 22.26 | 16.79; 27.73 | |
| Austria | 2 | 1537 | 65.77 |  |  |
| Bangladesh | 2 | 368 | 51.83 | 43.07; 60.60 | |
| Belgium | 2 | 1286 | 63.35 | 39.32; 87.38 | |
| Brazil | 14 | 10232 | 27.60 | 14.40; 40.79 | |
| Bulgaria | 1 | 2016 | 76.48 | 74.65; 78.32 | |
| Cambodia | 1 | 961 | 35.38 | -5.88; 76.64 | |
| Cameroon | 1 | 586 | 45.70 | 40.64; 50.76 | |
| Canada | 5 | 3481 | 45.39 | 14.83; 75.94 | |
| Chile | 0 |  |  | 0.00; 0.00 | |
| China | 43 | 247252 | 52.00 | 45.66; 58.35 | |
| Colombia | 2 | 39486 | 19.26 | 10.45; 28.06 | |
| Croatia | 1 | 260 | 58.00 | 52.00; 64.00 | |
| Denmark | 4 | 4660 | 36.40 | 12.14; 60.67 | |
| Ecuador | 2 | 2594 | 22.15 | 19.70; 24.61 | |
| Egypt | 4 | 1572 | 38.57 | 1.30; 75.85 | |
| Estonia | 1 | 65 | 92.10 | 85.54; 98.66 | |
| Ethiopia | 1 | 174 | 40.90 | 33.79; 48.02 | |
| Faroe Islands | 1 | 668 | 53.65 | 49.89; 57.41 | |
| Finland | 9 | 15663 | 27.96 | 14.33; 41.59 | |
| France | 1 | 892 | 34.66 | 31.54; 37.78 | |
| Germany | 7 | 31674 | 47.26 | 42.96; 51.57 | |
| Greece | 3 | 3926 | 55.19 | 45.44; 64.94 | |
| Greenland | 2 | 632 | 43.03 |  |  |
| Hungary | 1 | 882 | 71.00 | 67.55; 74.45 | |
| Iceland | 2 | 454 | 52.48 | 20.47; 84.49 | |
| India | 27 | 29244 | 62.09 | 51.95; 72.23 | |
| Indonesia | 5 | 699 | 56.62 | 43.53; 69.72 | |
| Iran | 21 | 55336 | 62.09 | 51.84; 72.33 | |
| Iraq | 1 | 300 | 75.30 | 70.42; 80.18 | |
| Ireland | 3 | 2119 | 39.96 | 24.16; 55.76 | |
| Italy | 4 | 4141 | 63.39 | 34.75; 92.03 | |
| Japan | 8 | 5973 | 45.18 | 26.38; 63.99 | |
| Jordan | 4 | 11491 | 57.09 | 19.50; 94.67 | |
| Kenya | 1 | 433 | 0.80 | -0.29; 1.89 | |
| Korea South | 13 | 201667 | 56.91 | 45.16; 68.65 | |
| Kuwait | 2 | 2376 | 82.18 | 80.35; 84.00 | |
| Lebanon | 3 | 1811 | 52.26 | 27.21; 77.31 | |
| Malaysia | 7 | 4923 | 41.62 | 23.52; 59.72 | |
| Mesoamerican Countries | 1 | 223 | 3.60 | 1.16; 6.05 | |
| Mexico | 8 | 9702 | 20.86 | 11.07; 30.65 | |
| Mongolia | 1 | 938 | 58.60 | 55.45; 61.75 | |
| Morocco | 0 |  |  | 0.00; 0.00 | |
| Nepal | 2 | 505 | 50.36 | 39.05; 61.67 | |
| Netherlands | 5 | 16651 | 30.70 | 25.04; 36.35 | |
| New Zealand | 4 | 2413 | 28.07 | 13.45; 42.70 | |
| Norway | 5 | 26820 | 46.99 | 27.78; 66.20 | |
| Oman | 1 | 2553 | 61.01 | 54.44; 67.57 | |
| Pakistan | 9 | 23766 | 55.06 | 39.46; 70.66 | |
| Palestine | 1 | 150 | 60.70 | 52.88; 68.52 | |
| Peru | 1 | 1134 | 14.77 | 12.93; 16.61 | |
| Philippines | 0 |  |  | 0.00; 0.00 | |
| Poland | 6 | 1760 | 42.82 | 30.93; 54.71 | |
| Portugal | 4 | 5881 | 62.49 | 39.00; 85.98 | |
| Qatar | 1 | 1205 | 64.40 | 61.70; 67.10 | |
| Russia | 2 | 410 | 74.51 |  |  |
| Saudi Arabia | 12 | 33879 | 86.63 | 79.42; 93.84 | |
| Singapore | 2 | 695 | 18.56 | 8.40; 28.72 | |
| Slovenia | 1 | 280 | 60.85 | 55.14; 66.56 | |
| South Africa | 5 | 4158 | 22.77 | 6.01; 39.53 | |
| Spain | 4 | 1499 | 31.92 | 5.38; 58.46 | |
| Sri Lanka | 2 | 2865 | 46.56 | 22.36; 70.77 | |
| Sweden | 5 | 9999 | 40.31 | 22.10; 58.51 | |
| Switzerland | 2 | 7220 | 34.38 | -11.59; 80.35 | |
| Syria | 1 | 197 | 59.40 | 52.54; 66.26 | |
| Taiwan | 3 | 4710 | 33.72 | 15.78; 51.66 | |
| Tajikistan | 1 | 1913 | 12.40 | 10.92; 13.88 | |
| Tanzania | 1 | 88 | 1.00 | -1.08; 3.08 | |
| Thailand | 2 | 1334 | 20.24 | -6.02; 46.49 | |
| Turkey | 9 | 3831 | 44.61 | 25.31; 63.91 | |
| UK | 9 | 468342 | 49.54 | 39.87; 59.21 | |
| USA | 12 | 78922 | 37.61 | 23.56; 51.67 | |
| Ukraine | 1 | 928 | 33.60 | 30.56; 36.64 | |
| United Arab Emirates | 3 | 1007 | 58.16 | 17.49; 98.83 | |
| Vietnam | 5 | 3298 | 42.98 | 26.41; 59.55 | |
| *<75 nmol/L* | | | | |  |
| Afghanistan | 0 |  |  |  | |
| Algeria | 2 | 1451 | 71.57 | 45.50; 97.64 | |
| Argentina | 1 | 355 | 99.60 |  |  |
| Armenia | 0 |  |  | 0.00; 0.00 | |
| Australia | 8 | 19478 | 66.55 | 59.24; 73.86 | |
| Austria | 1 | 1435 | 70.48 |  |  |
| Bangladesh | 1 | 152 | 93.42 | 89.48; 97.36 | |
| Belgium | 2 | 1286 | 89.14 | 79.71; 98.57 | |
| Brazil | 11 | 7338 | 57.84 | 45.92; 69.76 | |
| Bulgaria | 0 |  |  | 0.00; 0.00 | |
| Cambodia | 0 |  |  | 0.00; 0.00 | |
| Cameroon | 1 | 586 | 95.90 | 93.89; 97.92 | |
| Canada | 4 | 2366 | 59.86 | 43.26; 76.46 | |
| Chile | 2 | 1661 | 60.35 |  |  |
| China | 27 | 83024 | 81.38 | 74.68; 88.09 | |
| Colombia | 1 | 475 | 56.80 | 52.35; 61.26 | |
| Croatia | 1 | 260 | 87.00 | 82.91; 91.09 | |
| Denmark | 2 | 2343 | 58.29 | 50.00; 66.58 | |
| Ecuador | 1 | 2374 | 67.80 | 65.92; 69.68 | |
| Egypt | 3 | 1022 | 61.92 |  |  |
| Estonia | 1 | 65 |  | 0.00; 0.00 | |
| Ethiopia | 1 | 627 | 49.00 | 45.09; 52.91 | |
| Faroe Islands | 0 |  |  | 0.00; 0.00 | |
| Finland | 6 | 8065 | 77.55 | 69.10; 86.00 | |
| France | 1 | 892 | 80.26 | 77.65; 82.87 | |
| Germany | 1 | 1418 | 96.88 | 95.03; 98.72 | |
| Greece | 2 | 2451 | 83.77 |  |  |
| Greenland | 0 |  |  | 0.00; 0.00 | |
| Hungary | 2 | 1088 | 73.64 |  |  |
| Iceland | 1 | 139 | 88.00 | 82.60; 93.40 | |
| India | 21 | 20401 | 80.37 | 73.14; 87.61 | |
| Indonesia | 2 | 385 | 91.33 | 84.47; 98.20 | |
| Iran | 14 | 9503 | 77.48 | 67.20; 87.77 | |
| Iraq | 0 |  |  | 0.00; 0.00 | |
| Ireland | 1 | 741 | 71.50 | 68.25; 74.75 | |
| Italy | 3 | 1819 | 75.03 | 61.09; 88.96 | |
| Japan | 7 | 6463 | 83.23 | 70.77; 95.68 | |
| Jordan | 3 | 11778 | 46.27 | 2.94; 89.60 | |
| Kenya | 0 |  |  | 0.00; 0.00 | |
| Korea South | 11 | 162201 | 91.47 | 87.71; 95.23 | |
| Kuwait | 1 | 237 | 63.00 | 56.85; 69.15 | |
| Lebanon | 3 | 1811 | 78.12 |  |  |
| Malaysia | 2 | 1416 | 54.97 | -1.19; 111.12 | |
| Mesoamerican Countries | 1 | 223 | 44.40 | 37.88; 50.92 | |
| Mexico | 5 | 6988 | 72.26 | 58.05; 86.46 | |
| Mongolia | 1 | 938 | 85.50 | 83.25; 87.75 | |
| Morocco | 1 | 254 | 86.60 | 82.41; 90.79 | |
| Nepal | 1 | 324 | 86.10 | 82.33; 89.87 | |
| Netherlands | 2 | 4317 | 57.94 | 40.67; 75.20 | |
| New Zealand | 2 | 1836 | 79.58 |  |  |
| Norway | 3 | 25793 | 77.94 | 68.60; 87.29 | |
| Oman | 0 |  |  | 0.00; 0.00 | |
| Pakistan | 8 | 8436 | 78.92 | 65.81; 92.02 | |
| Palestine | 0 |  |  | 0.00; 0.00 | |
| Peru | 1 | 1134 | 85.58 | 83.72; 87.45 | |
| Philippines | 2 | 1622 | 58.29 | 53.79; 62.80 | |
| Poland | 5 | 4439 | 86.30 | 79.74; 92.87 | |
| Portugal | 4 | 5716 | 79.41 | 64.71; 94.11 | |
| Qatar | 1 | 1205 | 89.90 | 88.20; 91.60 | |
| Russia | 1 | 320 | 86.90 | 83.20; 90.60 | |
| Saudi Arabia | 6 | 8029 | 88.34 |  |  |
| Singapore | 2 | 695 | 63.62 | 40.88; 86.35 | |
| Slovenia | 1 | 280 | 83.92 | 79.62; 88.23 | |
| South Africa | 3 | 3451 | 68.01 | 49.11; 86.90 | |
| Spain | 4 | 1287 | 58.47 | 34.78; 82.16 | |
| Sri Lanka | 0 |  |  | 0.00; 0.00 | |
| Sweden | 3 | 8425 | 79.59 | 62.53; 96.66 | |
| Switzerland | 1 | 6485 | 88.21 | 87.42; 88.99 | |
| Syria | 1 | 197 | 82.75 | 77.47; 88.03 | |
| Taiwan | 3 | 4710 | 79.34 | 59.71; 98.97 | |
| Tajikistan | 0 |  |  | 0.00; 0.00 | |
| Tanzania | 0 |  |  | 0.00; 0.00 | |
| Thailand | 3 | 1674 | 62.90 | 44.79; 81.01 | |
| Turkey | 6 | 3074 | 49.59 | 31.33; 67.85 | |
| UK | 3 | 10827 | 82.84 | 74.93; 90.75 | |
| USA | 8 | 74809 | 60.07 | 43.38; 76.77 | |
| Ukraine | 1 | 928 | 66.60 | 63.57; 69.63 | |
| United Arab Emirates | 2 | 692 | 91.25 |  |  |
| Vietnam | 3 | 1976 | 76.70 | 57.06; 96.33 | |

25(OH)D, 25-hydroxyvitamin D; CI, confidence interval

^a^ Guatemala, El Salvador, the Dominican Republic, Honduras, Nicaragua, Costa Rica, Panama, Belize, and Mexico

^b^ England, Scotland, Wales and Northern Ireland

# Supplementary Table 9: Pooled prevalence estimates according to commonly reported thresholds by latitude, season, use of a certified assay/standardised data, study quality and country income classification

| Characteristics | | n (*publications*) | n  (*data points*) | | n  (*countries*) | n  *(participants)* | *n*  *(below cut-point)* | | Prevalence | 95% CI | | | *I^2^ (%)* | |
| --- | --- | --- | --- | --- | --- | --- | --- | --- | --- | --- | --- | --- | --- | --- |
| *<30 nmol/L* | | | | | | | | | | | | | | |
| Latitude | |  |  | |  |  |  | |  |  | | |  | |
| Low (<40^o^) | | 49 | 60 | | 37 | 268,839 | 50,058 | | 20.61 | 17.70; 23.51 | | | 99.9 | |
| High (≥40^o^) | | 27 | 29 | | 18 | 96,487 | 8,775 | | 13.90 | 11.71; 16.08 | | | 99.6 | |
| Season | |  |  | |  |  |  | |  |  | | |  | |
| Winter/Spring | | 16 | 17 | | 14 | 65,124 | 14,551 | | 16.20 | 10.28; 22.12 | | | 99.7 | |
| Summer/Autumn | | 16 | 17 | | 14 | 52,106 | 6,061 | | 7.48 | 4.34; 10.63 | | | 99.5 | |
| Use of a certified Assay/standardised data^a^ | |  |  | |  |  |  | |  |  | | |  | |
| Yes | | 22 | 24 | | 18 | 105,668 | 9,504 | | 11.56 | 9.26; 13.87 | | | 99.7 | |
| No | | 54 | 65 | | 47 | 259,658 | 49,329 | | 21.16 | 18.29; 24.04 | | | 99.9 | |
| Study quality^b^ | |  |  | |  |  |  | |  |  | | |  | |
| Low | | 2 | 2 | | 2 | 222 | 54 | | 25.48 | 8.84; 42.12 | | | 87.6 | |
| Moderate | | 44 | 48 | | 38 | 94,012 | 10,231 | | 15.32 | 13.35;17.30 | | | 99.5 | |
| High | | 31 | 39 | | 28 | 271,092 | 48,548 | | 21.33 | 17.86; 24.80 | | | 99.9 | |
| Income | |  |  | |  |  |  | |  |  | | |  | |
| High income | | 40 | 43 | | 23 | 120,103 | 11,114 | | 14.25 | 12.46; 16.05 | | | 99.5 | |
| Upper middle income | | 17 | 18 | | 9 | 198,533 | 35,617 | | 18.09 | 12.73; 23.46 | | | 99.9 | |
| Lower middle income | | 16 | 20 | | 12 | 33,483 | 7,615 | | 24.42 | 18.75; 30.09 | | | 99.4 | |
| Low income | | 2 | 4 | | 2 | 2,607 | 1,227 | | 36.66 | -7.05; 80.37 | | | 99.9 | |
| *<50 nmol/L* | | | | | | | | | | | | | | |
| Latitude |  | | |  |  |  |  | |  | | |  |  | |
| Low (<40^o^) | 269 | | | 287 | 57 | 841,405 | 452,180 | | 48.11 | | | 44.58, 51.64 | 99.9 | |
| High (≥40^o^) | 92 | | | 97 | 31 | 607,792 | 311,461 | | 45.11 | | | 41.00, 49.22 | 99.8 | |
| Season |  | | |  |  |  |  | |  | | |  |  | |
| Winter/Spring | 39 | | | 41 | 26 | 338,339 | 215,171 | | 51.83 | | | 44.64, 59.01 | 99.9 | |
| Summer/Autumn | 37 | | | 39 | 25 | 333,762 | 133,405 | | 35.11 | | | 28.68, 41.54 | 99.9 | |
| Use of a certified Assay/standardised data^a^ |  | | |  |  |  |  | |  | | |  |  | |
| Yes | 53 | | | 58 | 30 | 697,175 | 353,871 | | 42.98 | | | 37.32, 48.64 | 99.9 | |
| No | 308 | | | 326 | 84 | 752,022 | 409,770 | | 48.13 | | | 44.80, 51.45 | 99.9 | |
| Study quality^b^ |  | | |  |  |  |  | |  | | |  |  | |
| Low | 13 | | | 14 | 11 | 18,599 | 8,415 | | 49.45 | | | 32.04, 66.87 | 99.9 | |
| Moderate | 274 | | | 282 | 75 | 502,840 | 283,302 | | 47.08 | | | 43.52, 50.63 | 99.9 | |
| High | 75 | | | 88 | 45 | 927,758 | 471,924 | | 47.95 | | | 42.70, 53.20 | 100.0 | |
| Income |  | | |  |  |  |  | |  | | |  |  | |
| High income | 165 | | | 172 | 40 | 968,257 | 523,885 | | 46.67 | | | 42.75, 50.60 | 99.9 | |
| Upper middle income | 101 | | | 104 | 15 | 328,426 | 177,720 | | 42.60 | | | 37.45, 47.76 | 99.9 | |
| Lower middle income | 88 | | | 95 | 19 | 130,284 | 50,205 | | 54.12 | | | 47.18, 61.06 | 99.9 | |
| Low income | 2 | | | 4 | 2 | 2,579 | 1,800 | | 61.71 | | | 31.86, 91.55 | 99.7 | |
| *<75 nmol/L* | | | | | | | | | | | | | | |
| Latitude |  | | |  |  |  |  |  | | |  | | |  |
| Low (<40^o^) | 188 | | | 195 | 51 | 471,873 | 382,379 | 74.53 | | | 72.63, 76.43 | | | 99.8 |
| High (≥40^o^) | 53 | | | 55 | 28 | 87,792 | 65,498 | 75.55 | | | 72.39, 78.71 | | | 99.3 |
| Season |  | | |  |  |  |  |  | | |  | | |  |
| Winter/Spring | 19 | | | 19 | 17 | 56,050 | 40,826 | 73.94 | | | 66.37, 81.50 | | | 99.8 |
| Summer/Autumn | 20 | | | 20 | 17 | 63,905 | 39,670 | 65.58 | | | 60.24, 70.93 | | | 99.3 |
| Use of a certified Assay/standardised data^a^ |  | | |  |  |  |  |  | | |  | | |  |
| Yes | 29 | | | 31 | 24 | 147,169 | 104,134 | 77.09 | | | 71.80, 82.37 | | | 99.8 |
| No | 212 | | | 219 | 73 | 412,496 | 343,743 | 74.42 | | | 72.72, 76.12 | | | 99.8 |
| Study quality^b^ |  | | |  |  |  |  |  | | |  | | |  |
| Low | 12 | | | 12 | 11 | 12,629 | 9,824 | 72.78 | | | 64.19, 81.36 | | | 99.1 |
| Moderate | 185 | | | 190 | 71 | 379,436 | 321,800 | 75.58 | | | 74.12, 77.04 | | | 99.6 |
| High | 44 | | | 48 | 35 | 167,600 | 116,253 | 72.55 | | | 66.58, 78.52 | | | 99.9 |
| Income |  | | |  |  |  |  |  | | |  | | |  |
| High income | 107 | | | 110 | 38 | 373,953 | 304,970 | 76.64 | | | 74.22, 79.05 | | | 99.8 |
| Upper middle income | 62 | | | 64 | 13 | 112,008 | 90,547 | 71.67 | | | 68.57, 74.78 | | | 99.8 |
| Lower middle income | 66 | | | 68 | 16 | 61,963 | 43,911 | 75.82 | | | 70.63, 81.08 | | | 99.8 |
| Low income | 1 | | | 1 | 1 | 627 | 307 | 49.00 | | | 45.09, 52.91 | | | - |

CI, confidence interval

^a^Use of a 25-hydroxyvitamin D assay that was certified to the reference measurement procedures (RMPs) developed under the Vitamin D Standardization Program (VDSP), or data that have been retrospectively harmonised to a certified assay;

^b^Evaluated using a tool developed by Hoy and colleagues ^587^: a low (>8), moderate (6–8), or high (≤5)

risk of bias on the basis of the overall score

# Supplementary Table 10: Univariate meta-regression analysis mean circulating 25-hydroxyvitamin D concentration: Men

| Characteristics | Studies (*n*) | Coefficient | Lower 95% CI | Upper 95% CI | *P* |
| --- | --- | --- | --- | --- | --- |
| **Region** |  |  |  |  |  |
| Africa | 12 | 20.21 | 9.07 | 31.34 | **0.00** |
| Asia | 128 | ref. | ref. | ref. |  |
| Europe | 54 | 1.28 | -4.70 | 7.27 | 0.68 |
| North America | 13 | 8.85 | -1.97 | 19.66 | 0.11 |
| South America | 9 | 10.30 | -2.37 | 22.97 | 0.11 |
| Oceania | 12 | 15.29 | 4.17 | 26.41 | **0.01** |
| **Latitude** |  |  |  |  |  |
| Low | 173 | ref. |  |  |  |
| High | 55 | -0.36 | -6.30 | 5.57 | 0.90 |
| **Certified assay** |  |  |  |  |  |
| Yes | 34 | ref. | ref. | ref. |  |
| No | 194 | 3.29 | -3.78 | 10.37 | 0.36 |
| **Study design** |  |  |  |  |  |
| Cohort | 41 | ref. | ref. | ref. |  |
| Cross-sectional | 187 | -0.94 | -7.51 | 5.64 | 0.78 |
| **Population** |  |  |  |  |  |
| Adults | 134 | ref. | ref. | ref. | ref. |
| Children | 53 | 3.06 | -3.10 | 9.21 | 0.33 |
| Mixed | 41 | -5.48 | -12.23 | 1.27 | 0.11 |
| **Study quality** |  |  |  |  |  |
| Low | 39 | -5.20 | -17.82 | 7.43 | 0.82 |
| Moderate risk | 177 | -1.33 | -12.75 | 10.09 | 0.82 |
| High | 12 | ref. | ref. | ref. |  |
| **Income** |  |  |  |  |  |
| High income | 102 | ref. | ref. | ref. | ref. |
| Upper middle income | 65 | 2.21 | -3.89 | 8.31 | 0.48 |
| Lower middle income | 53 | 1.77 | -4.74 | 8.28 | 0.59 |
| Low income | 2 | 6.77 | -20.64 | 34.19 | 0.63 |

25(OH)D, 25-hydroxyvitamin D; CI, confidence interval

# Supplementary Table 11: Multi-variate meta-regression analysis of mean circulating 25-hydroxyvitamin D concentration: Men

| Characteristics* | Studies (*n*) | Coefficient | Lower 95% CI | Upper 95% CI | *P* |
| --- | --- | --- | --- | --- | --- |
| **Region** |  |  |  |  |  |
| Africa | 10 | 20.91 | 6.51 | 35.32 | **0.00** |
| Asia | 128 | ref. | ref. | ref. | ref. |
| Europe | 51 | 4.82 | -7.89 | 17.53 | 0.46 |
| North America | 12 | 10.02 | -3.48 | 23.52 | 0.15 |
| South America | 9 | 10.56 | -2.82 | 23.94 | 0.12 |
| Oceania | 12 | 17.91 | 4.80 | 31.01 | **0.01** |
| **Latitude** |  |  |  |  |  |
| Low | 167 | ref. |  |  |  |
| High | 55 | 0.17 | -11.39 | 11.72 | 0.98 |
| **Certified assay** |  |  |  |  |  |
| Yes | 34 | ref. | ref. | ref. | ref. |
| No | 188 | 2.88 | -5.47 | 11.24 | 0.50 |
| **Study design** |  |  |  |  |  |
| Cohort | 40 | ref. | ref. | ref. |  |
| Cross-sectional | 182 | -0.62 | -7.79 | 6.54 | 0.87 |
| **Population** |  |  |  |  |  |
| Adults | 133 | ref. | ref. | ref. | ref. |
| Children | 49 | 1.32 | -5.20 | 7.83 | 0.69 |
| Mixed | 40 | -4.38 | -11.72 | 2.95 | 0.24 |
| **Study quality** |  |  |  |  |  |
| Low | 37 | -0.25 | -13.87 | 13.38 | 0.97 |
| Moderate risk | 173 | 0.96 | -11.22 | 13.14 | 0.88 |
| High | 12 | ref. | ref. | ref. | ref. |
| **Income** |  |  |  |  |  |
| High income | 102 | ref. | ref. | ref. | ref. |
| Upper middle income | 65 | 4.08 | 0.31 | -3.76 | 11.92 |
| Lower middle income | 53 | 3.97 | 0.35 | -4.35 | 12.30 |
| Low income | 2 | -5.01 | 0.76 | -36.75 | 26.72 |
| * Mutually adjusted |  |  |  |  |  |

25(OH)D, 25-hydroxyvitamin D; CI, confidence interval

# Supplementary Table 12: Univariate meta-regression analysis mean circulating 25-hydroxyvitamin D concentration: Women

| **Characteristics** | **Studies (*n*)** | **Coefficient** | **Lower 95% CI** | **Upper 95% CI** | **P** |
| --- | --- | --- | --- | --- | --- |
| **Region** |  |  |  |  |  |
| Africa | 13 | 19.26 | 9.40 | 29.12 | **0.00** |
| Asia | 162 | ref. | ref. | ref. |  |
| Europe | 62 | 6.78 | 4.44 | 1.67 | **0.01** |
| North America | 15 | 21.16 | 16.26 | 11.87 | **0.00** |
| South America | 11 | 13.27 | 8.34 | 2.64 | **0.01** |
| Oceania | 10 | 23.02 | 17.59 | 11.88 | **0.00** |
| **Latitude** |  |  |  |  |  |
| Low | 210 | ref. | ref. | ref. |  |
| High | 63 | 4.89 | -0.38 | 10.16 | 0.07 |
| **Certified assay** |  |  |  |  |  |
| Yes | 37 | ref. | ref. | ref. |  |
| No | 236 | 2.36 | -4.14 | 8.87 | 0.48 |
| **Study design** |  |  |  |  |  |
| Cohort | 52 | ref. | ref. | ref. |  |
| Cross-sectional | 221 | -2.94 | -8.61 | 2.73 | 0.31 |
| **Population** |  |  |  |  |  |
| Adults | 166 | ref. | ref. | ref. |  |
| Children | 96 | -4.89 | -9.58 | -0.20 | **0.04** |
| Mixed | 11 | -6.64 | -18.02 | 4.74 | 0.25 |
| **Study quality** |  |  |  |  |  |
| Low | 43 | -10.96 | -22.60 | 0.68 | 0.07 |
| Moderate risk | 217 | -6.68 | -17.19 | 3.83 | 0.21 |
| High | 13 | ref. | ref. | ref. |  |
| **Income** |  |  |  |  |  |
| High income | 122 | ref. | ref. | ref. |  |
| Upper middle income | 77 | -0.35 | -5.72 | 0.90 | 0.84 |
| Lower middle income | 68 | -1.50 | -7.09 | 0.60 | 0.28 |
| Low income | 2 | 7.89 | -18.39 | 0.56 | 0.27 |

25(OH)D, 25-hydroxyvitamin D; CI, confidence interval

# Supplementary Table 13: Multi-variate meta-regression analysis mean circulating 25-hydroxyvitamin D concentration: Women

| Characteristics* | Studies (*n*) | Coefficient | Lower 95% CI | Upper 95% CI | *P* |
| --- | --- | --- | --- | --- | --- |
| **Region** |  |  |  |  |  |
| Africa | 12 | 17.02 | 5.47 | 28.57 | **0.00** |
| Asia | 162 | ref. | ref. | ref. |  |
| Europe | 60 | 8.94 | -1.63 | 19.52 | 0.10 |
| North America | 14 | 24.53 | 13.78 | 35.27 | **0.00** |
| South America | 11 | 14.85 | 3.72 | 25.98 | **0.01** |
| Oceania | 10 | 26.47 | 14.04 | 38.90 | **0.00** |
| **Latitude** |  |  |  |  |  |
| Low | 206 | ref. |  |  |  |
| High | 63 | 0.43 | -9.30 | 10.15 | 0.93 |
| **Certified assay** |  |  |  |  |  |
| Yes | 37 | ref. | ref. | ref. |  |
| No | 232 | 2.41 | -4.48 | 9.30 | 0.49 |
| **Study design** |  |  |  |  |  |
| Cohort | 51 | ref. | ref. | ref. |  |
| Cross-sectional | 218 | -0.64 | -6.40 | 5.13 | 0.83 |
| **Population** |  |  |  |  |  |
| Adults | 166 | ref. | ref. | ref. | ref. |
| Children | 92 | -5.64 | -10.33 | -0.94 | **0.02** |
| Mixed | 11 | -8.29 | -19.66 | 3.08 | 0.15 |
| **Study quality** |  |  |  |  |  |
| Low | 41 | -4.40 | -5.32 | 10.75 | 0.51 |
| Moderate risk | 215 | -2.75 | -16.02 | 7.22 | 0.46 |
| High | 13 | ref. | ref. | ref. |  |
| **Income** |  |  |  |  |  |
| High income | 122 | ref. | ref. | ref. | ref. |
| Upper middle income | 77 | 4.43 | -1.84 | 10.69 | 0.17 |
| Lower middle income | 68 | 5.47 | -1.12 | 12.06 | 0.10 |
| Low income | 2 | 0.58 | -27.36 | 28.52 | 0.97 |
| * Mutually adjusted |  |  |  |  |  |

25(OH)D, 25-hydroxyvitamin D; CI, confidence interval

# Supplementary Figure 1: Publication bias

**A** Egger’s funnel plot


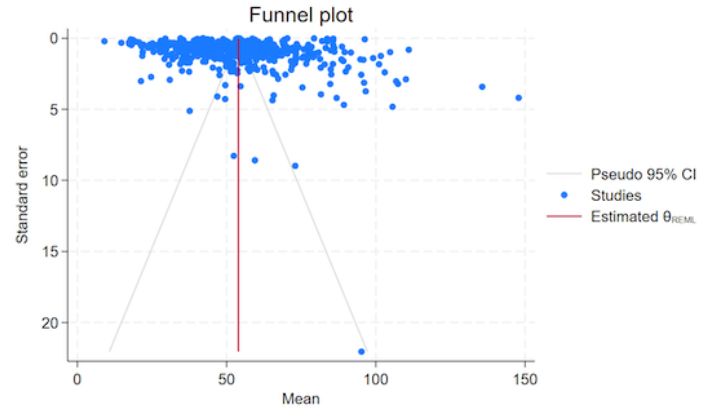


# Supplementary Figure 2: World map of pooled mean 25-hydroxyvitamin D concentration (nmol/L)


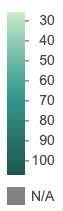

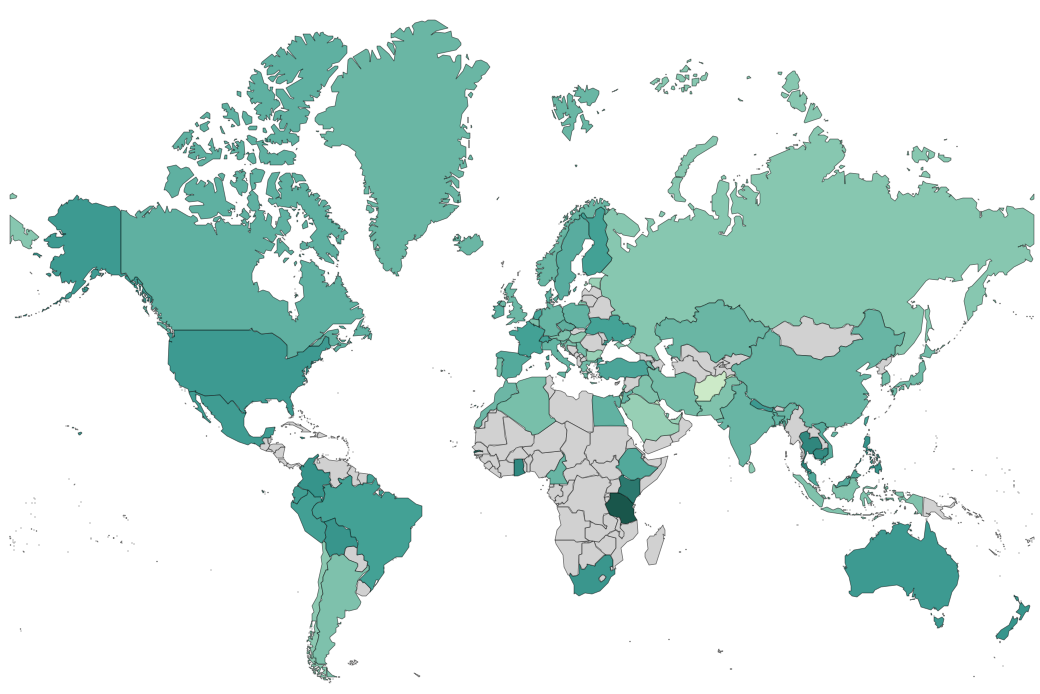


(N/A: data not available)

# Supplementary Figure 3: Forest plot for overall pooled mean circulating 25-hydroxyvitamin D concentration


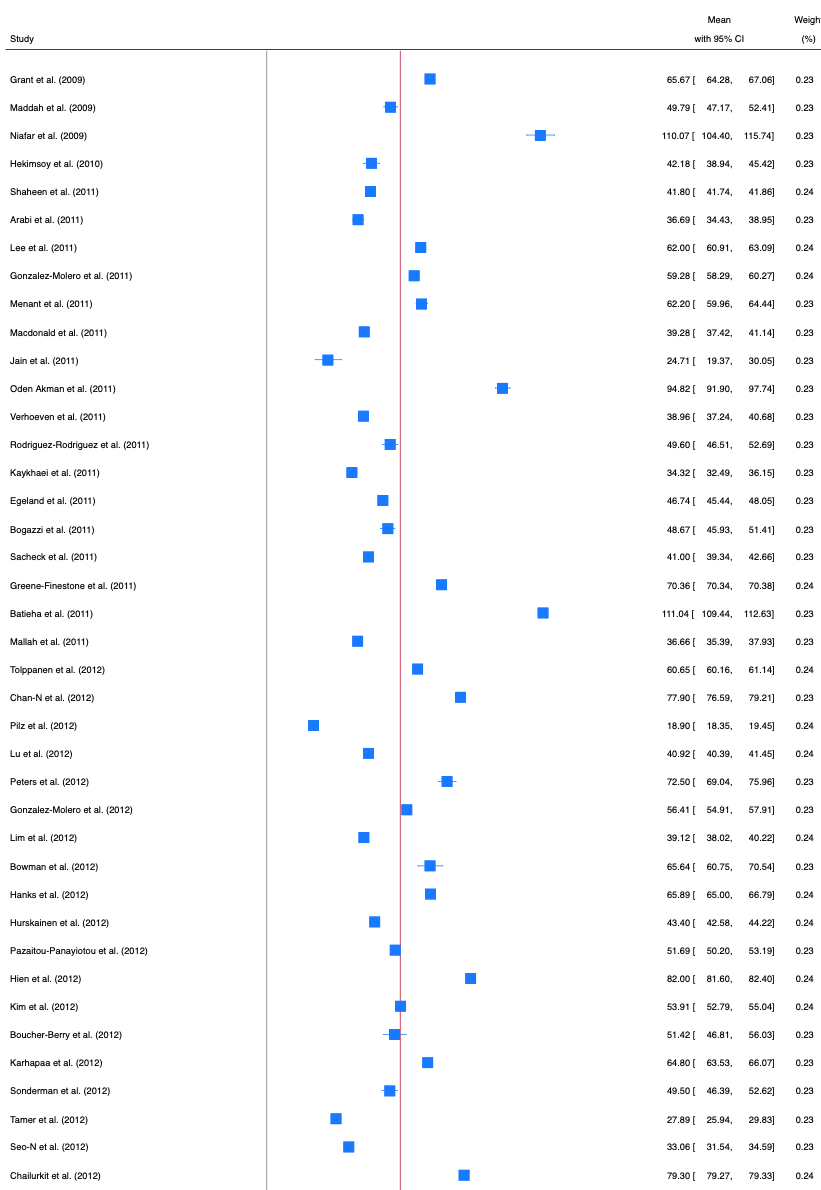


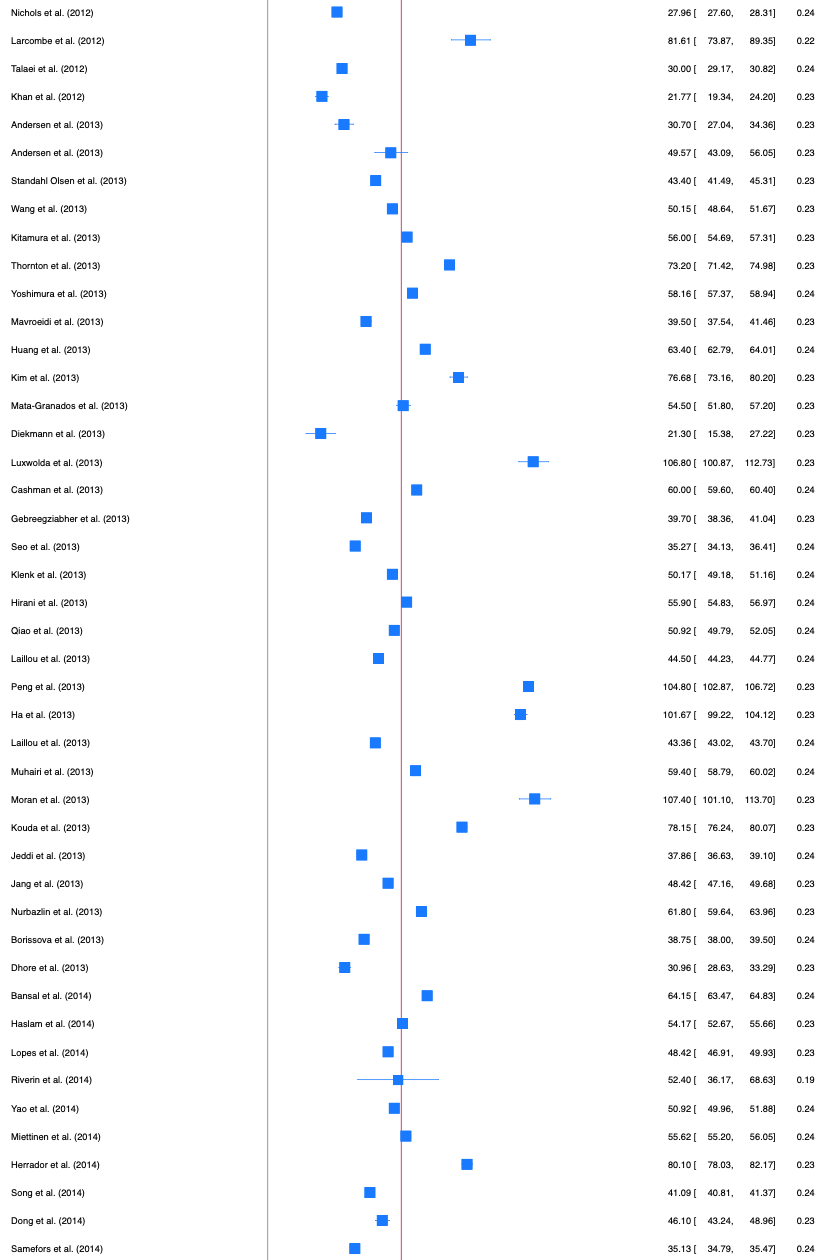


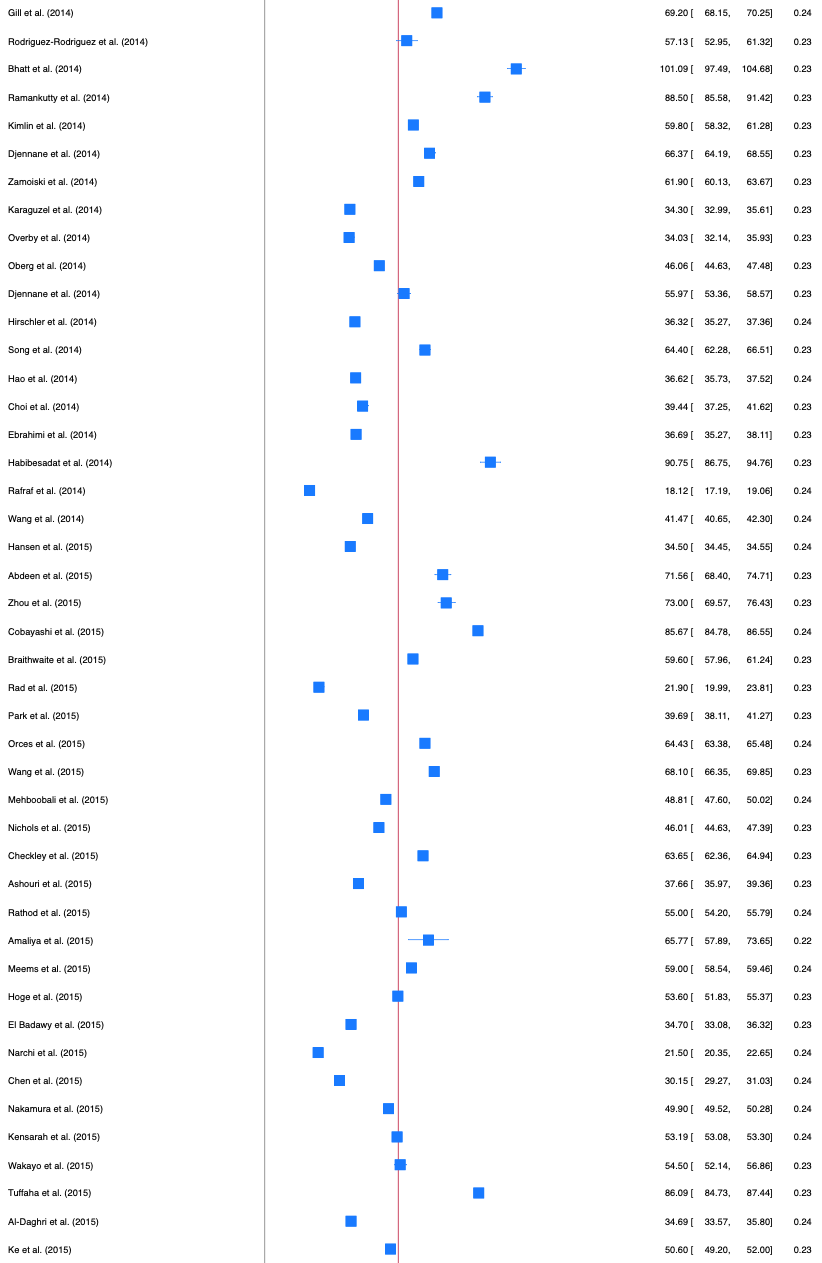


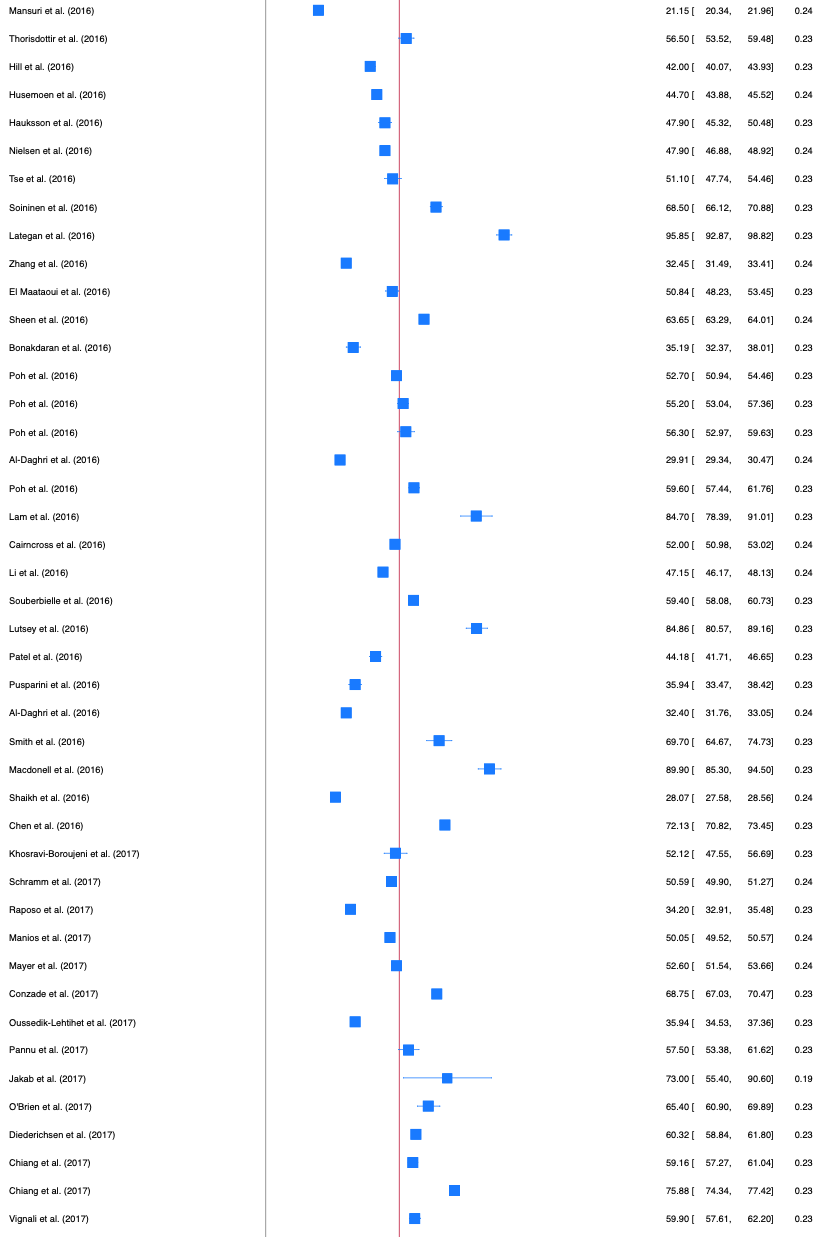


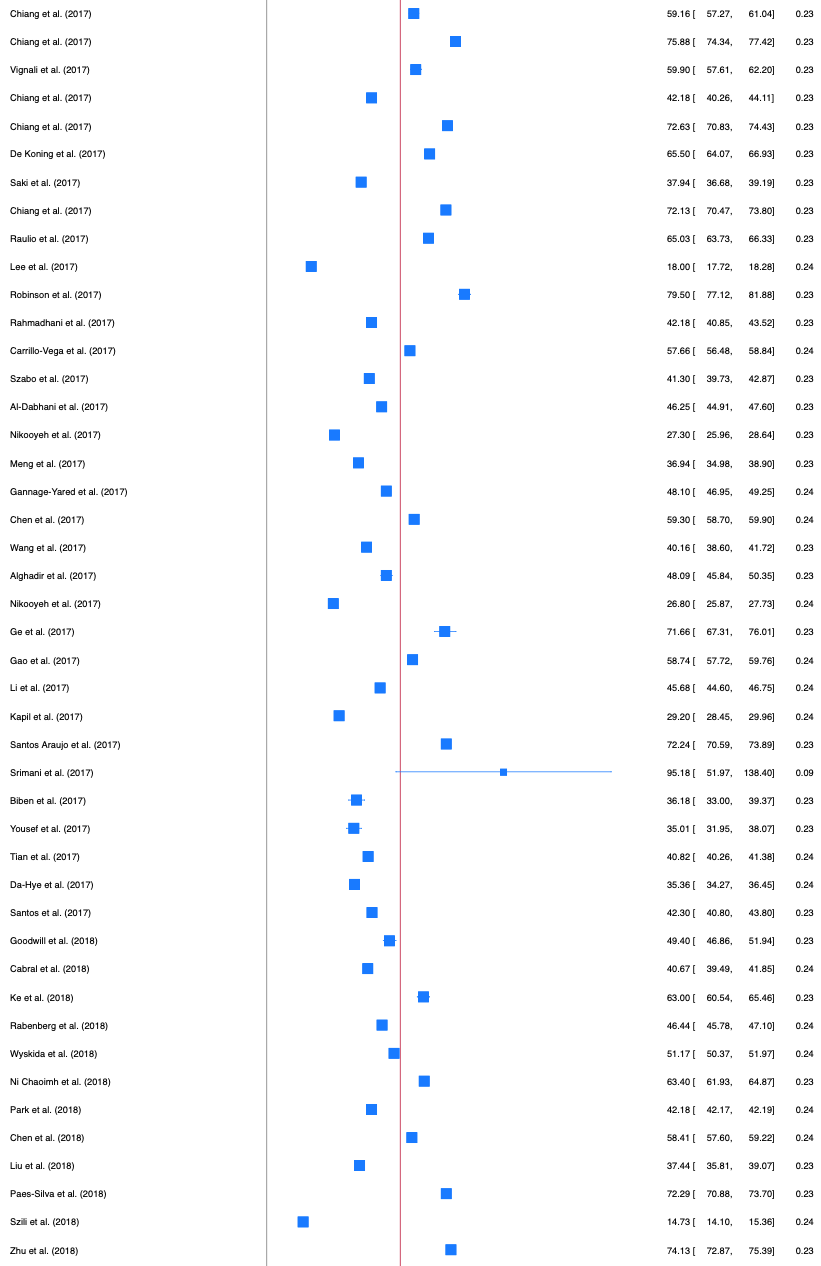


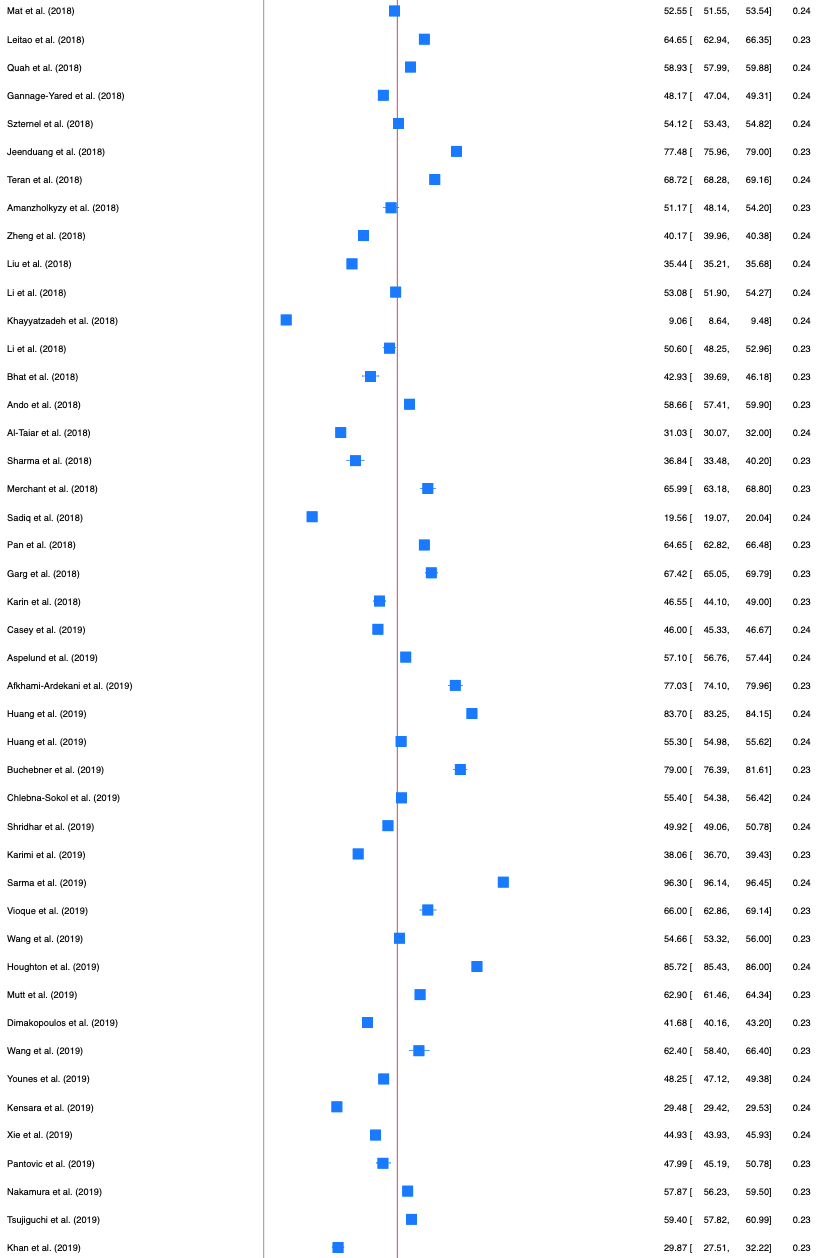


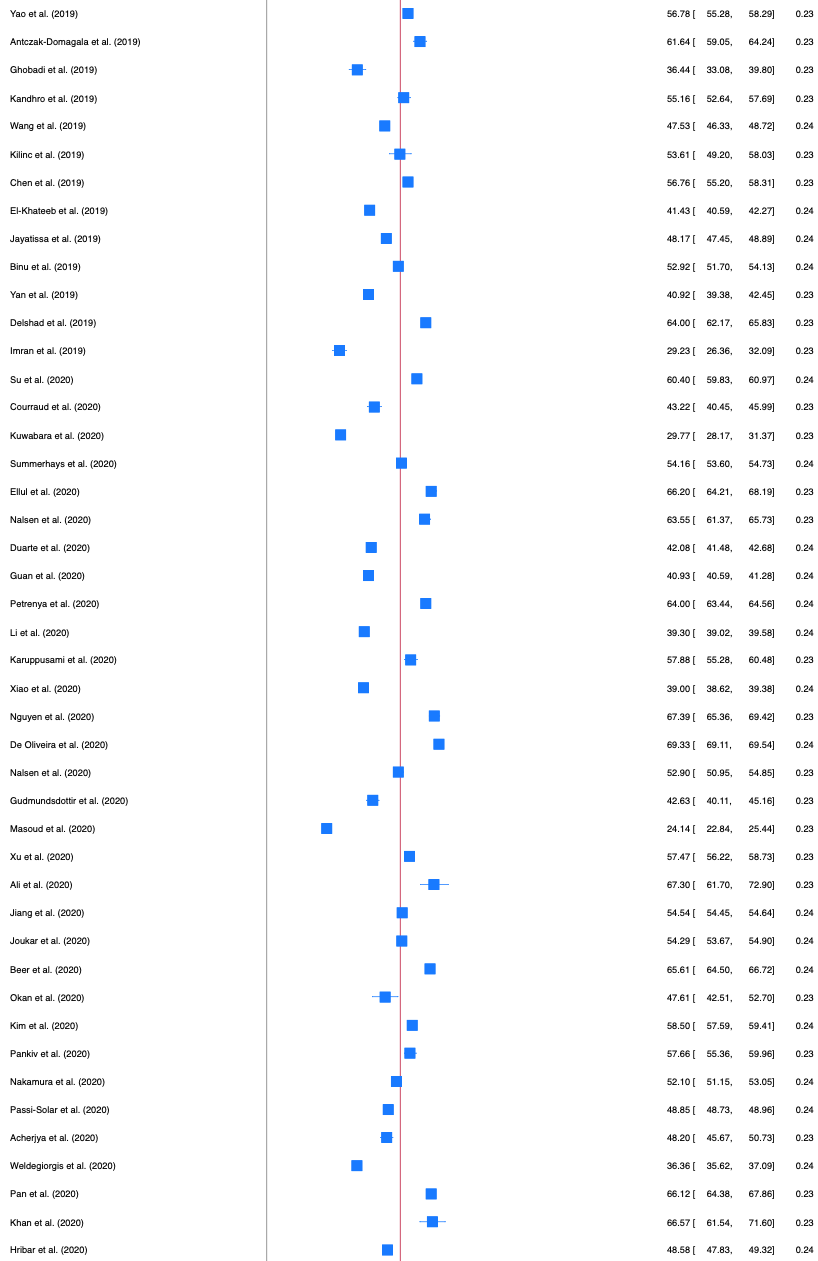


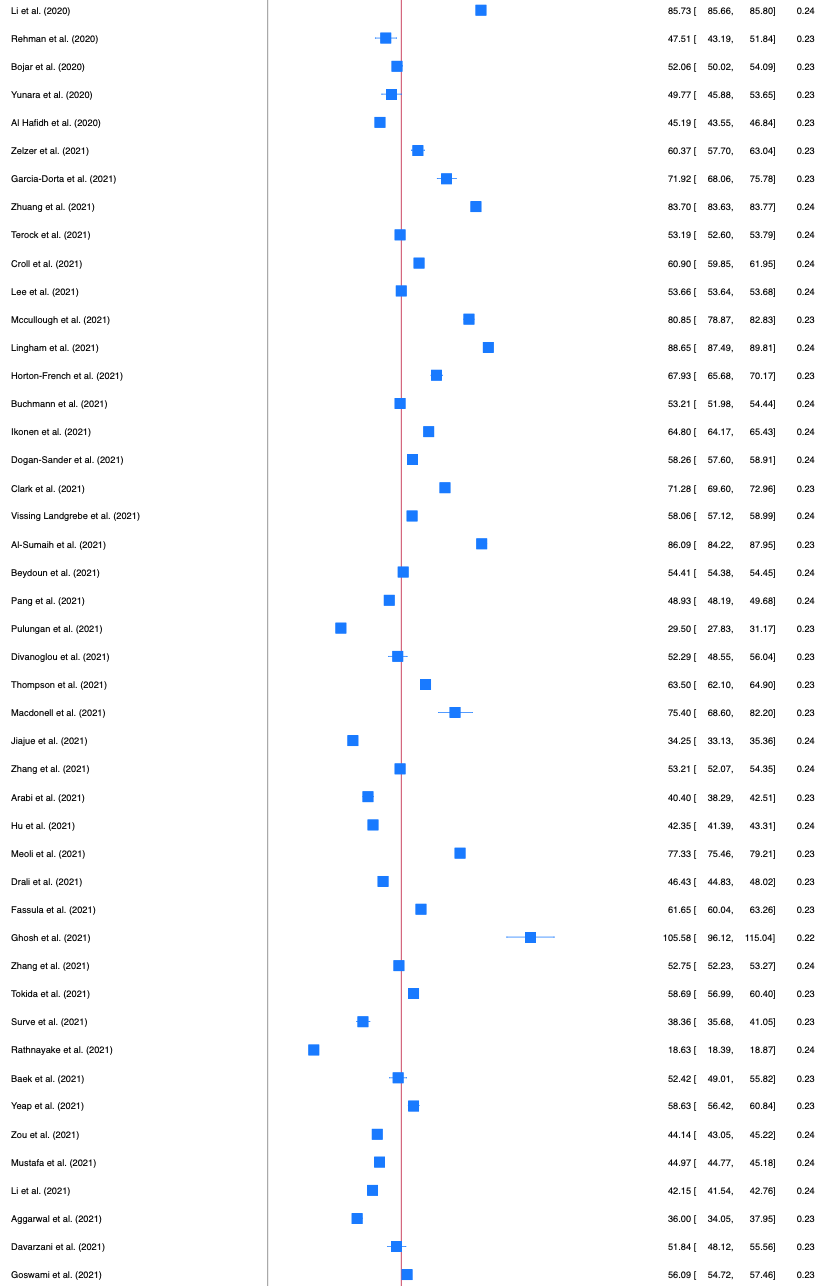


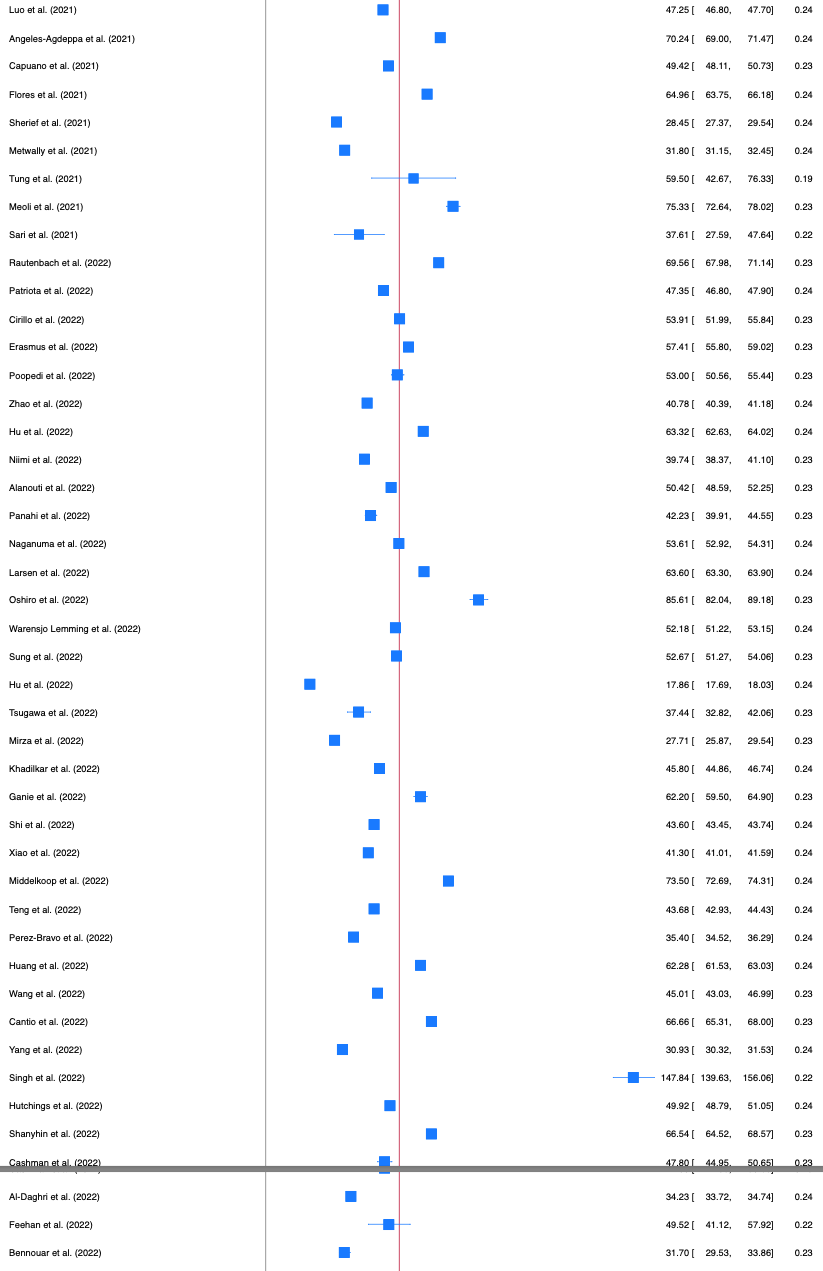


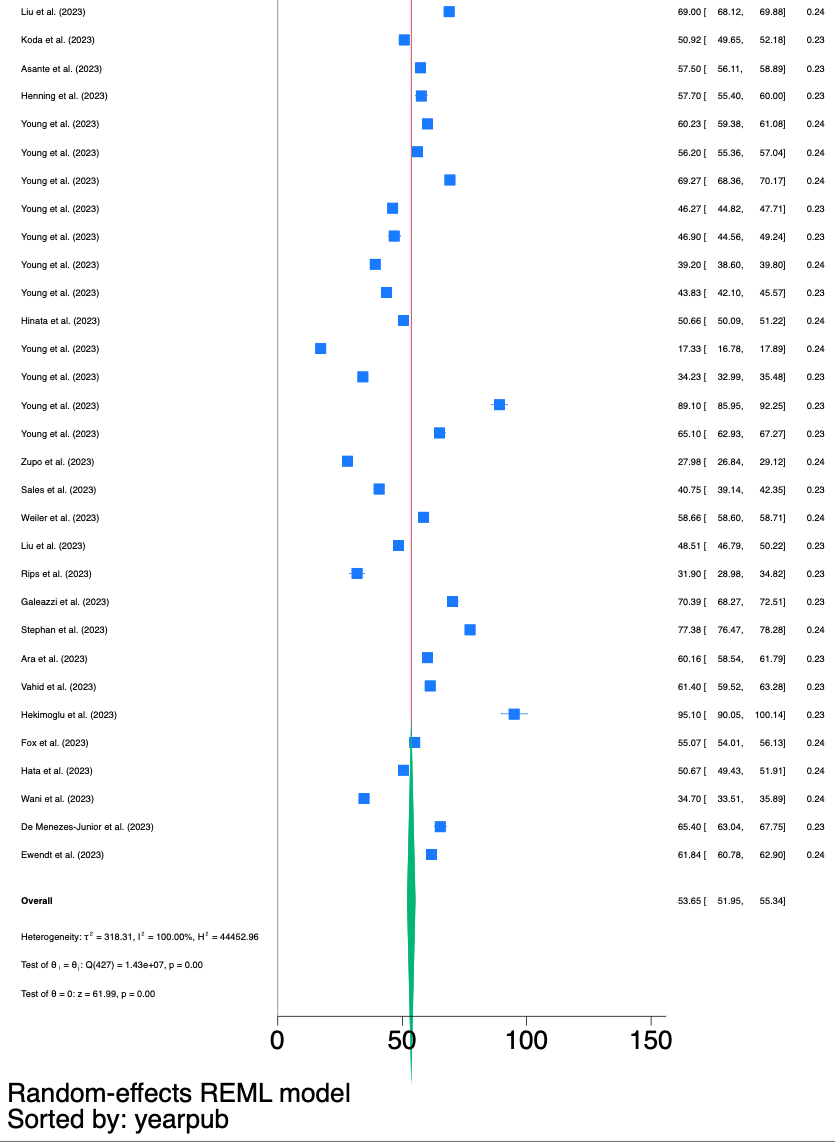


# Supplementary Figure 4a: Forest plot for pooled mean circulating 25-hydroxyvitamin D concentration by country – Africa


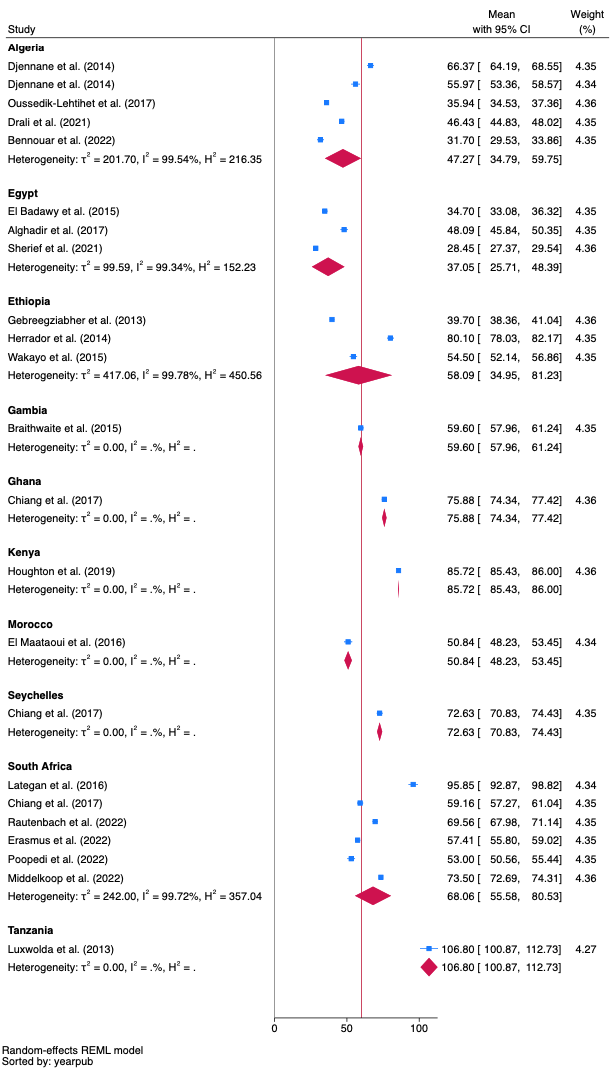


# Supplementary Figure 4b: Forest plot for pooled mean circulating 25-hydroxyvitamin D concentration by country – Asia


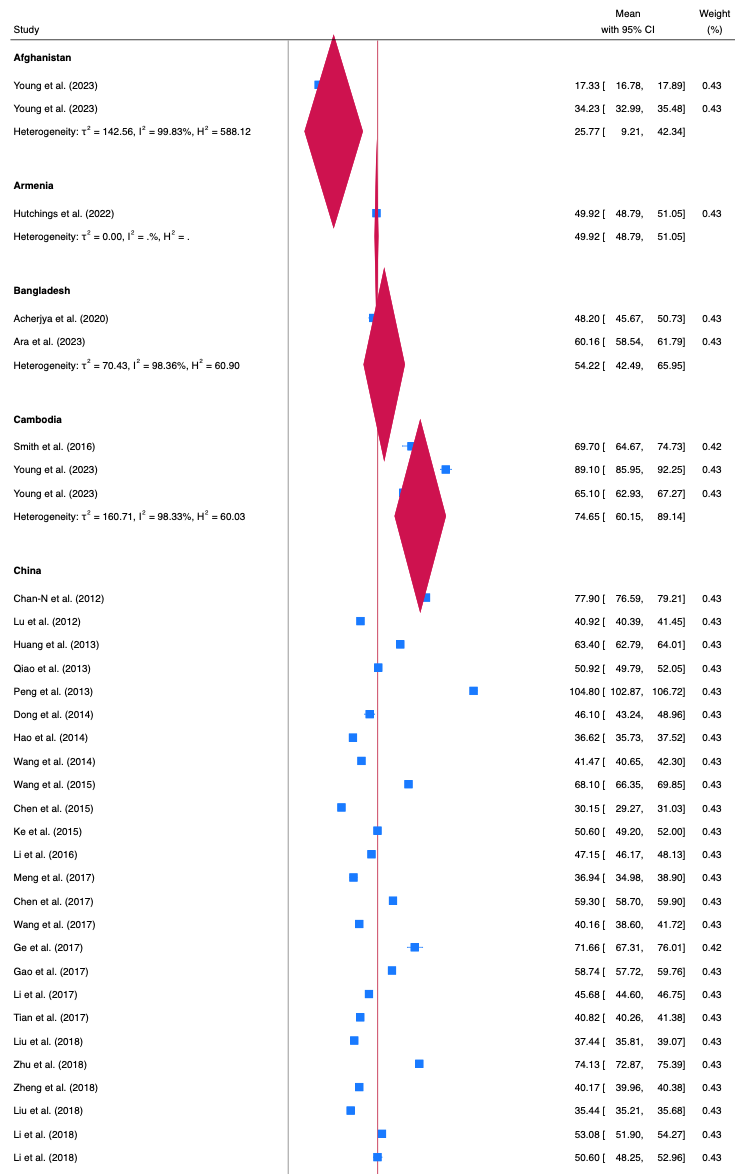


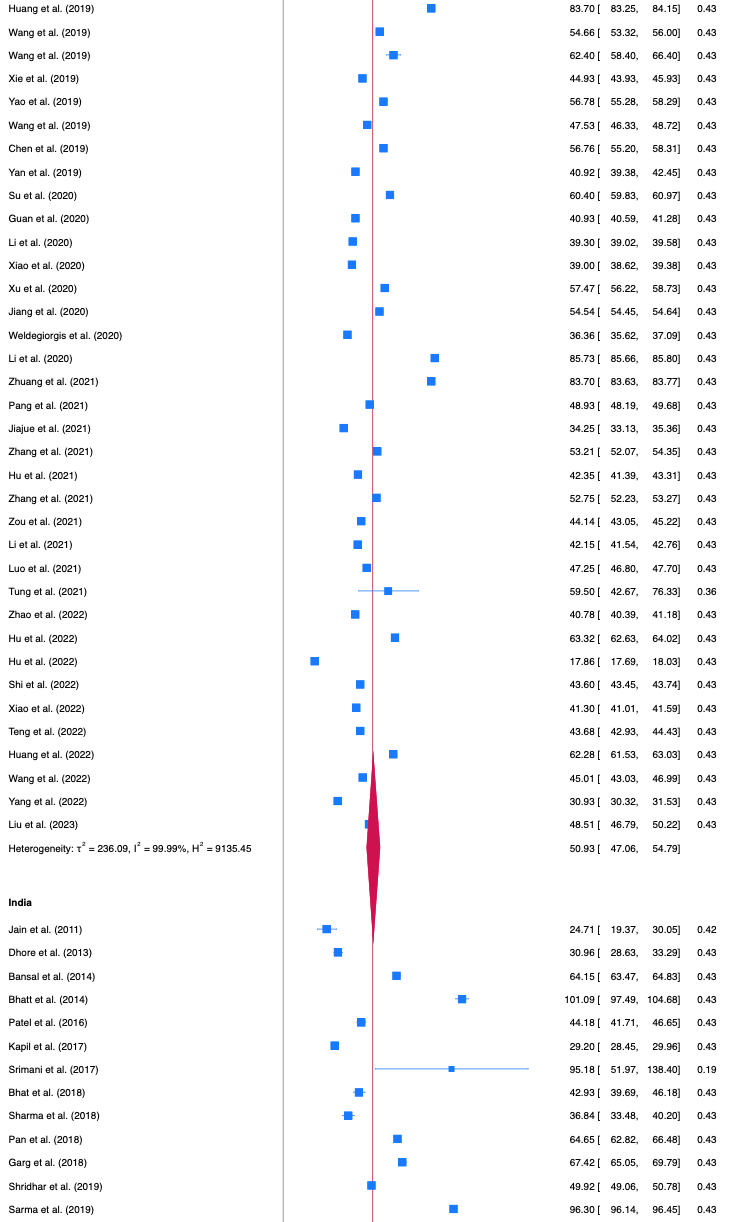


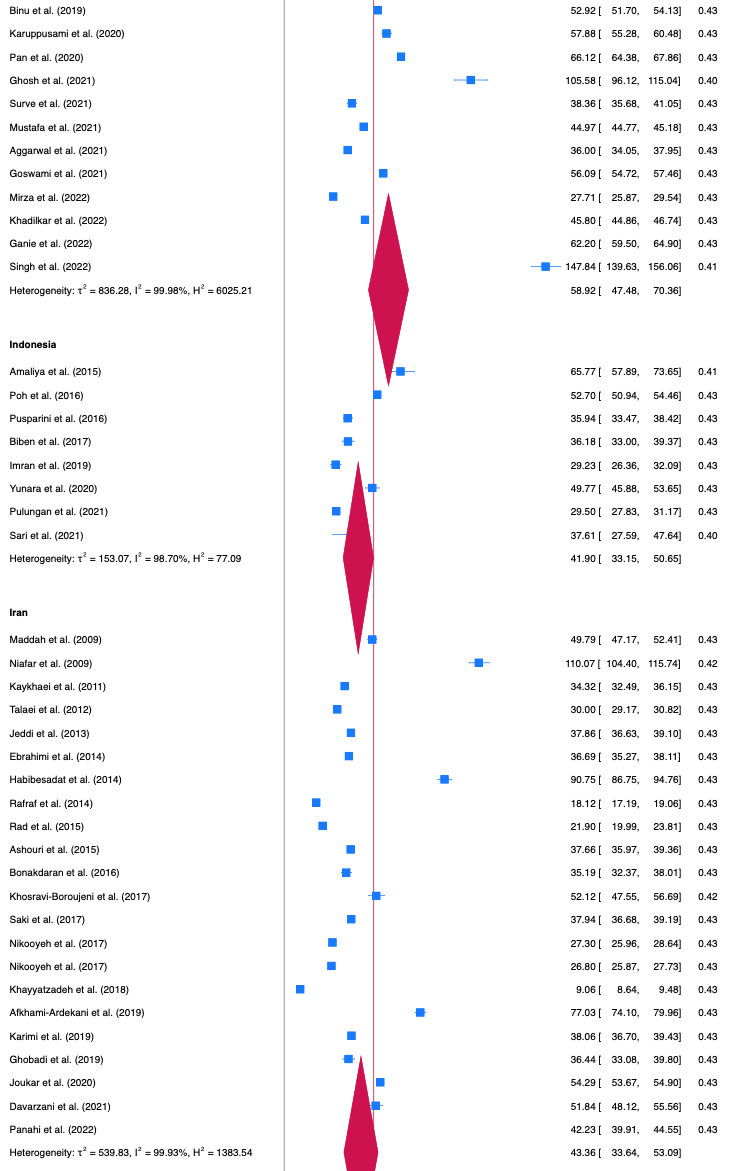


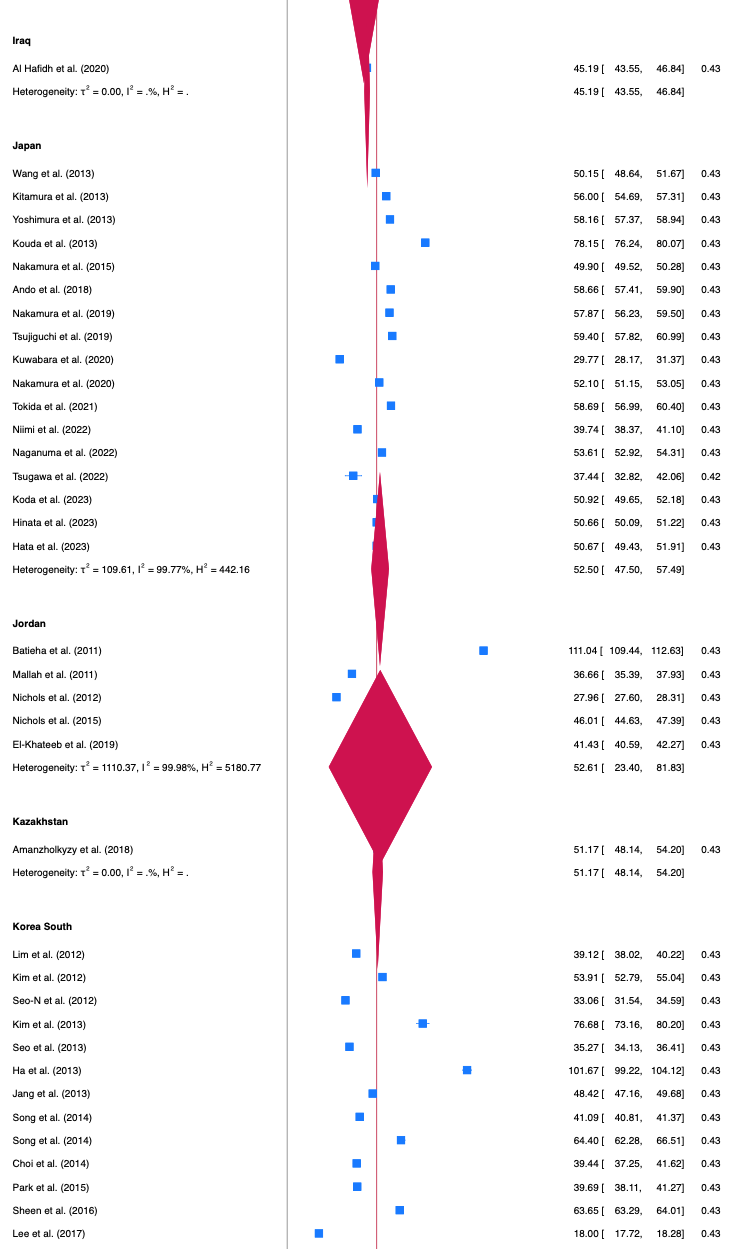


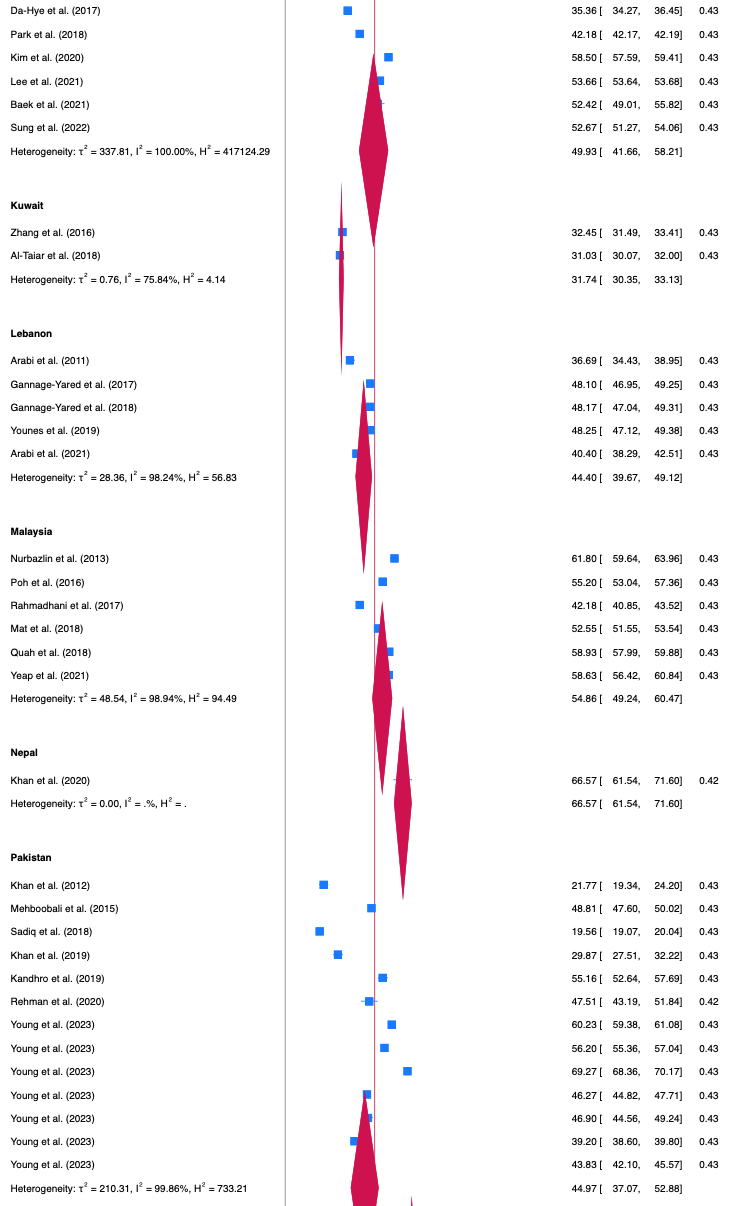


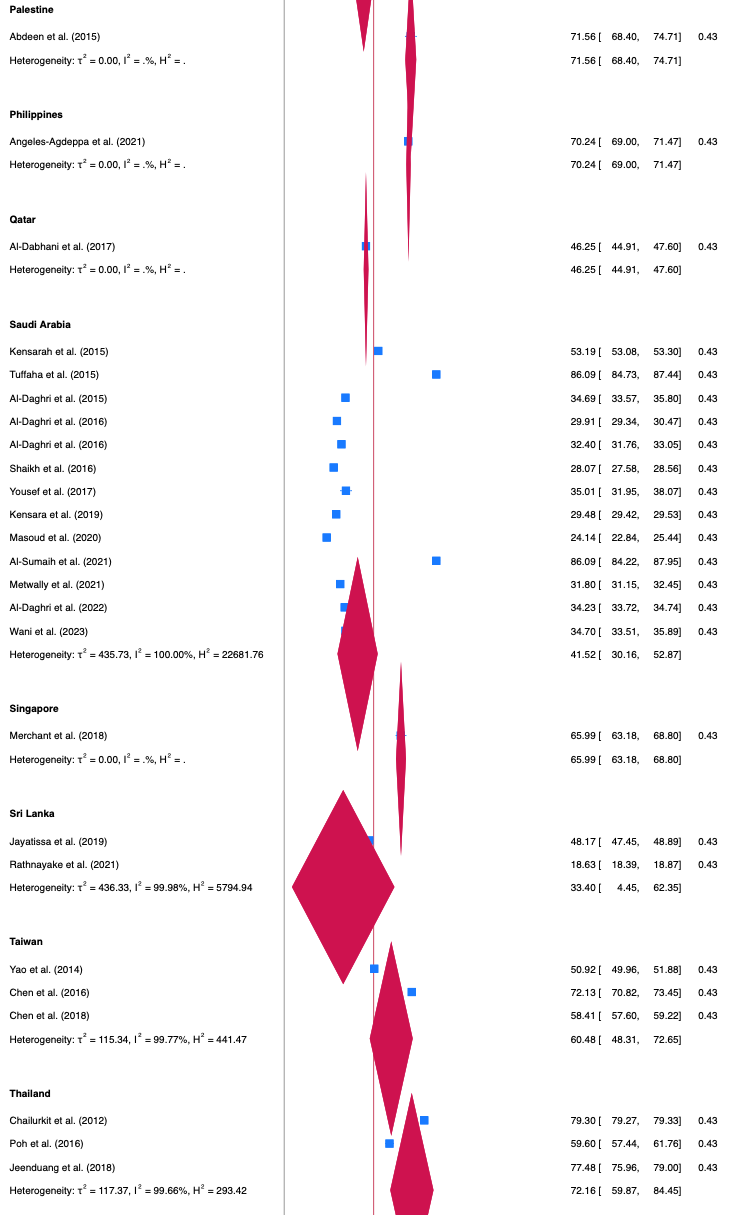


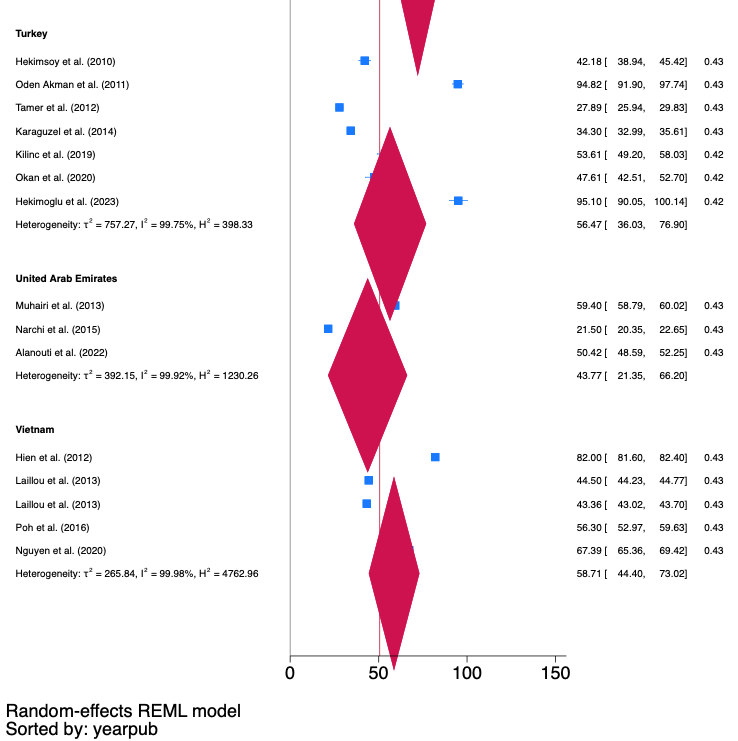


# Supplementary Figure 4c: Forest plot for pooled mean circulating 25-hydroxyvitamin D concentration by country – Europe


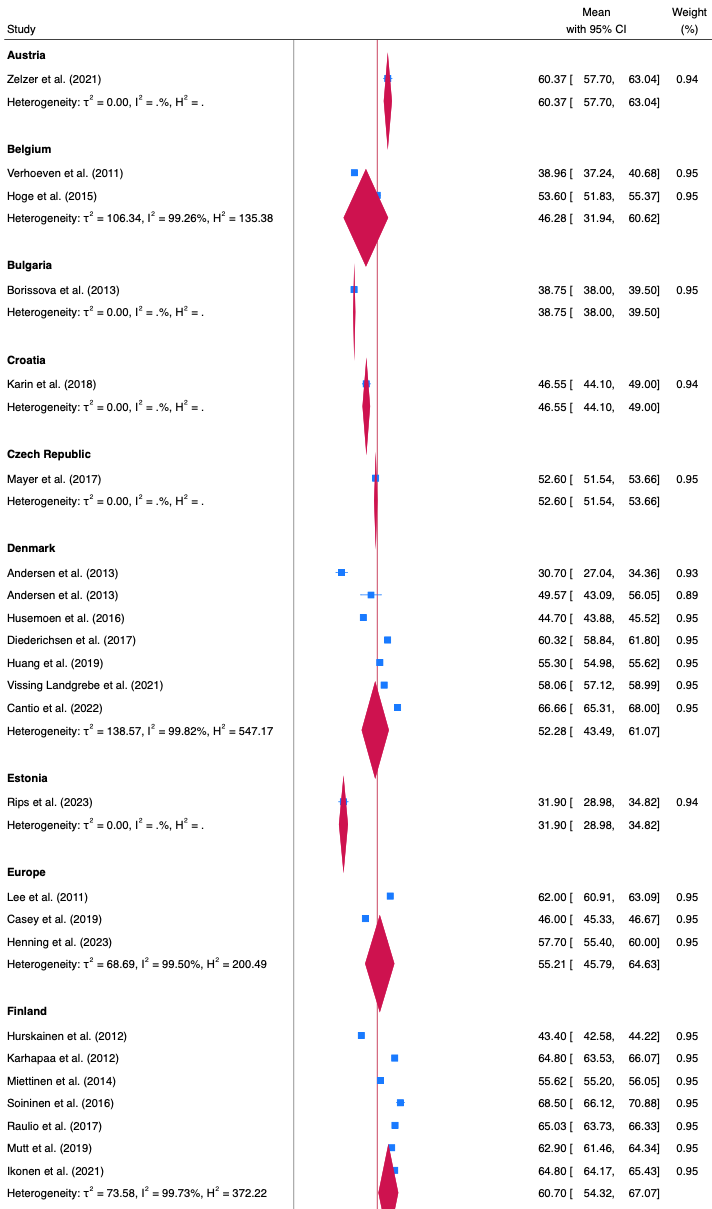


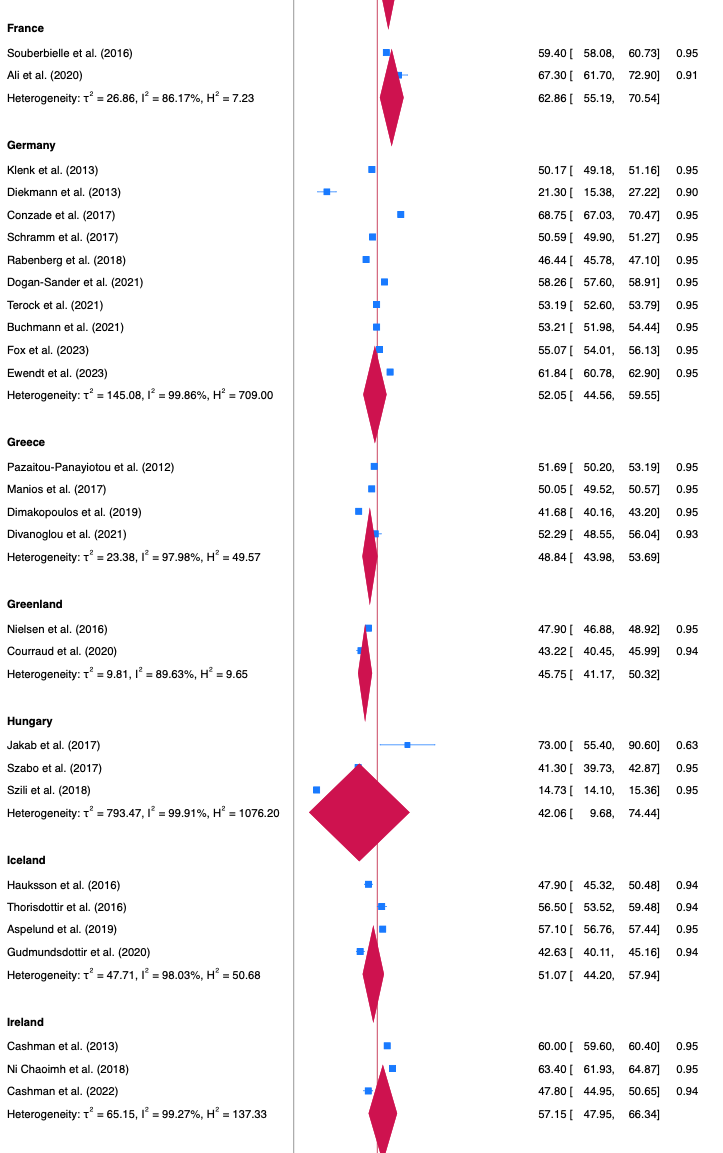


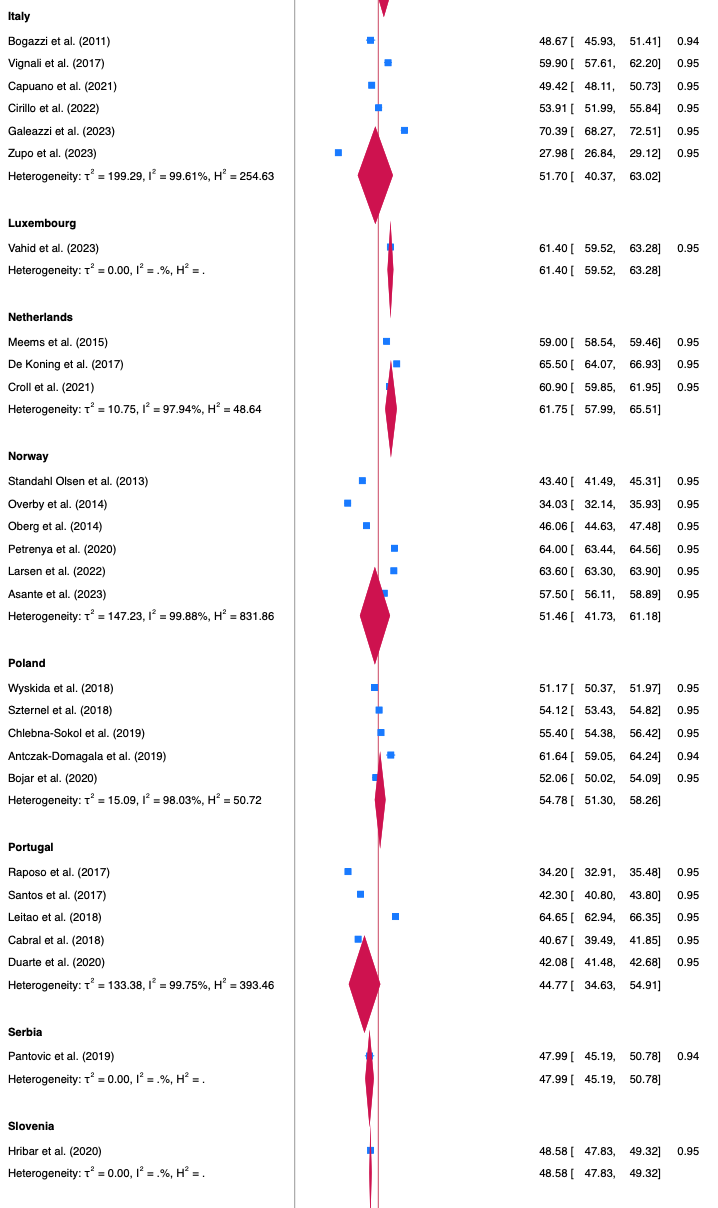


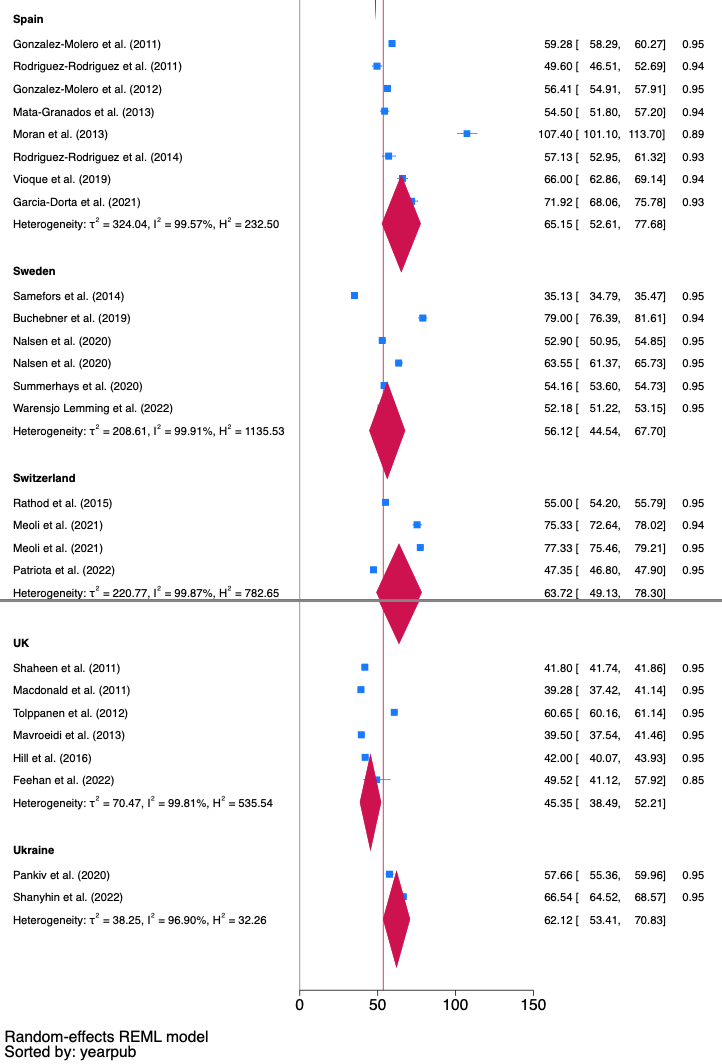


# Supplementary Figure 4d: Forest plot for pooled mean circulating 25-hydroxyvitamin D concentration by country – North America


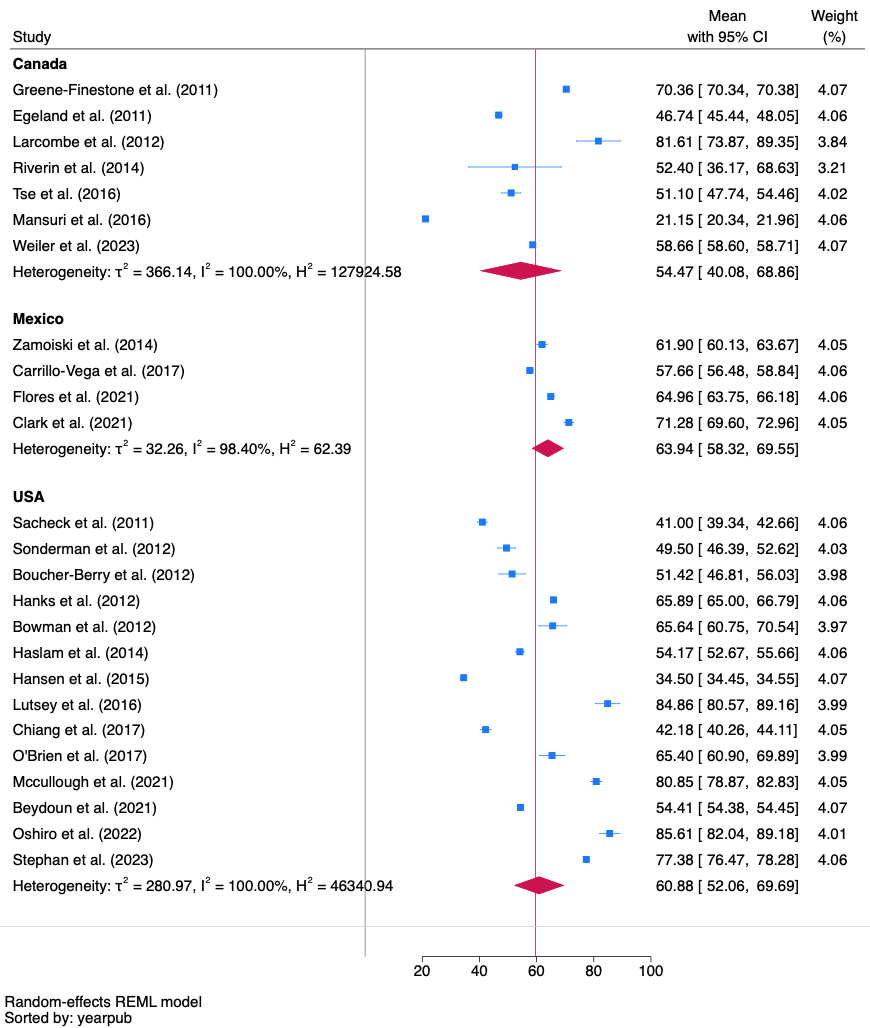


# Supplementary Figure 4e: Forest plot for pooled mean circulating 25-hydroxyvitamin D concentration by country – South America

# Supplementary Figure 4f: Forest plot for pooled mean circulating 25-hydroxyvitamin D concentration by country - Oceania

# Supplementary Figure 5: Forest plot for pooled mean circulating 25-hydroxyvitamin D concentration by high (≥40^o^) and low (<40^o^) latitude


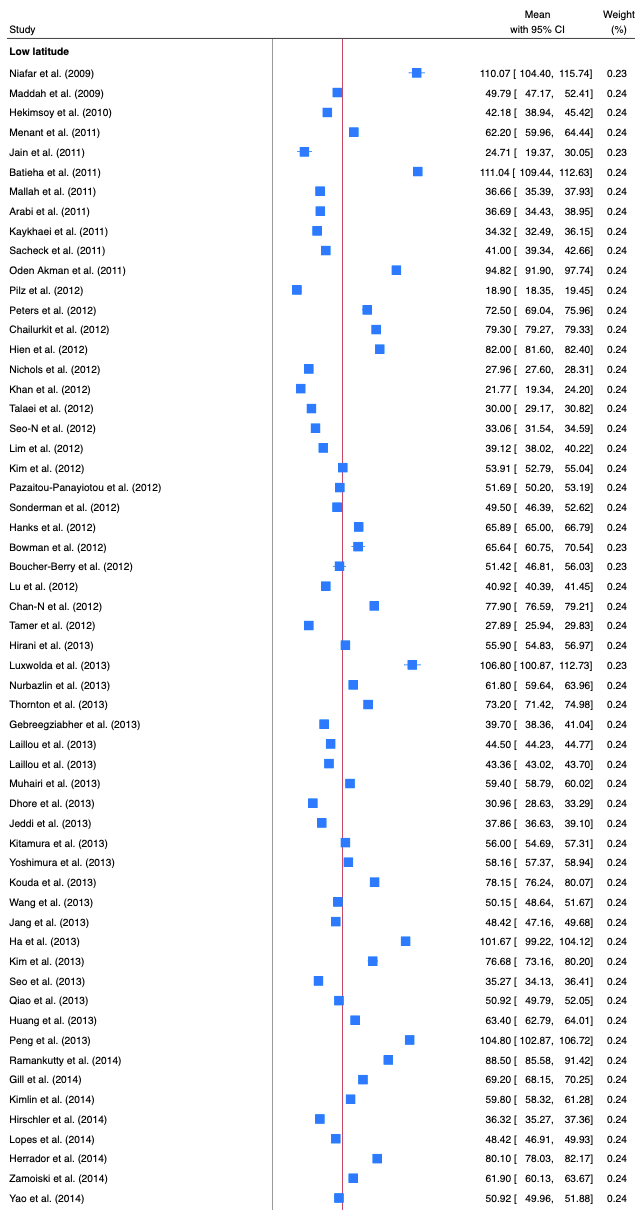


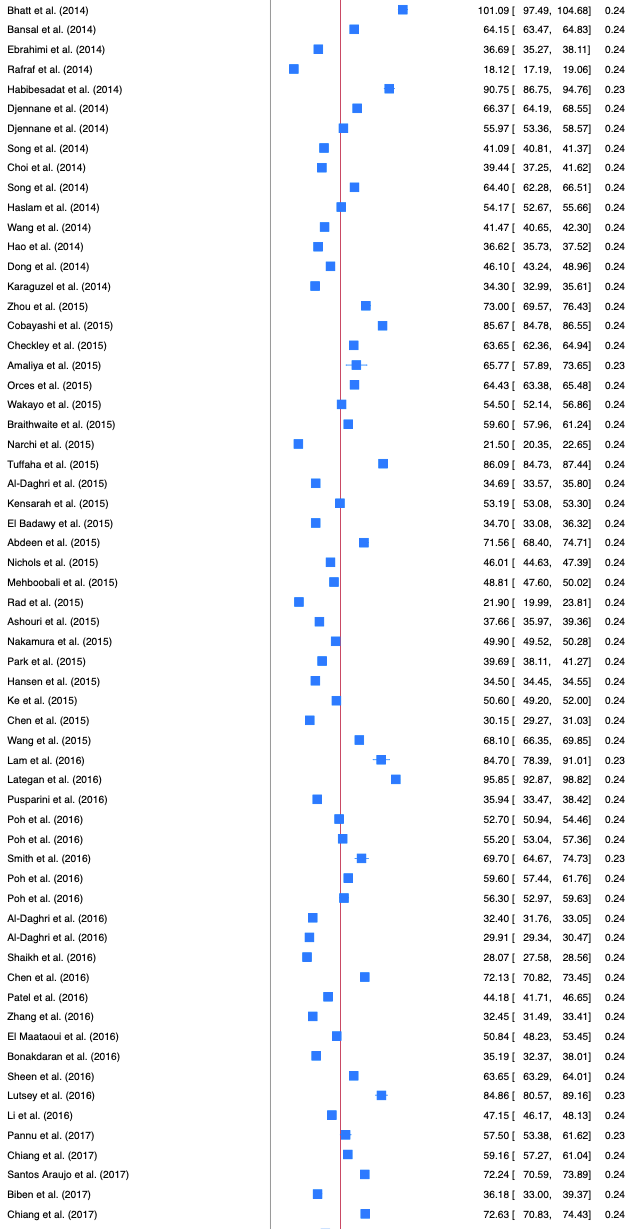


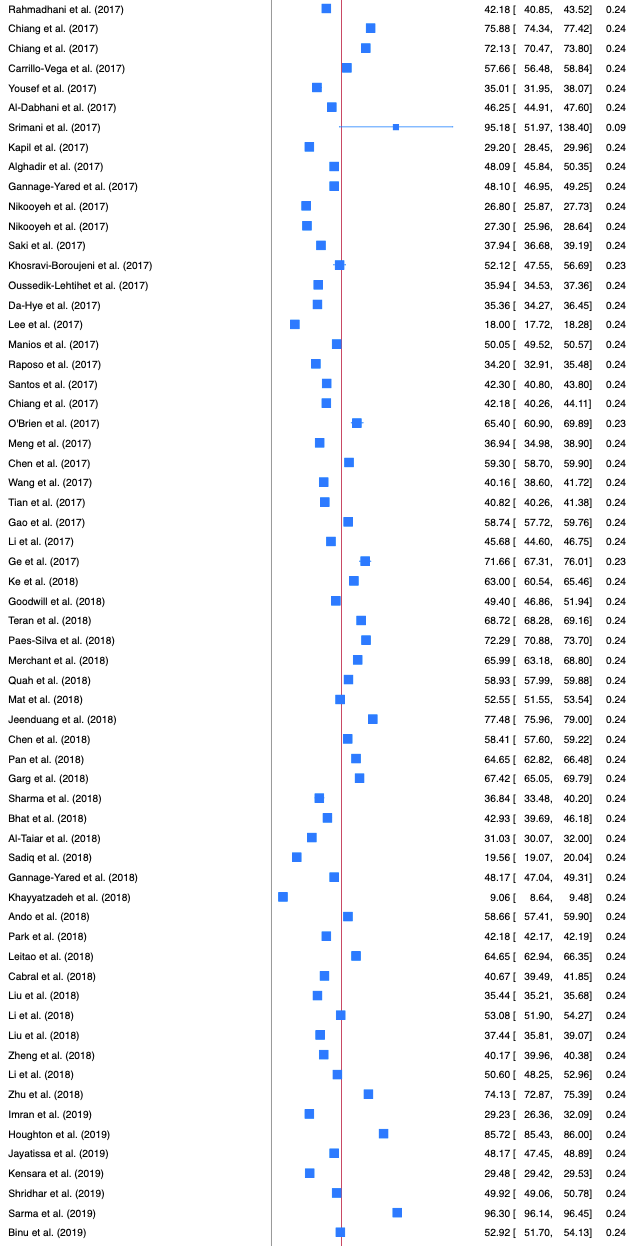


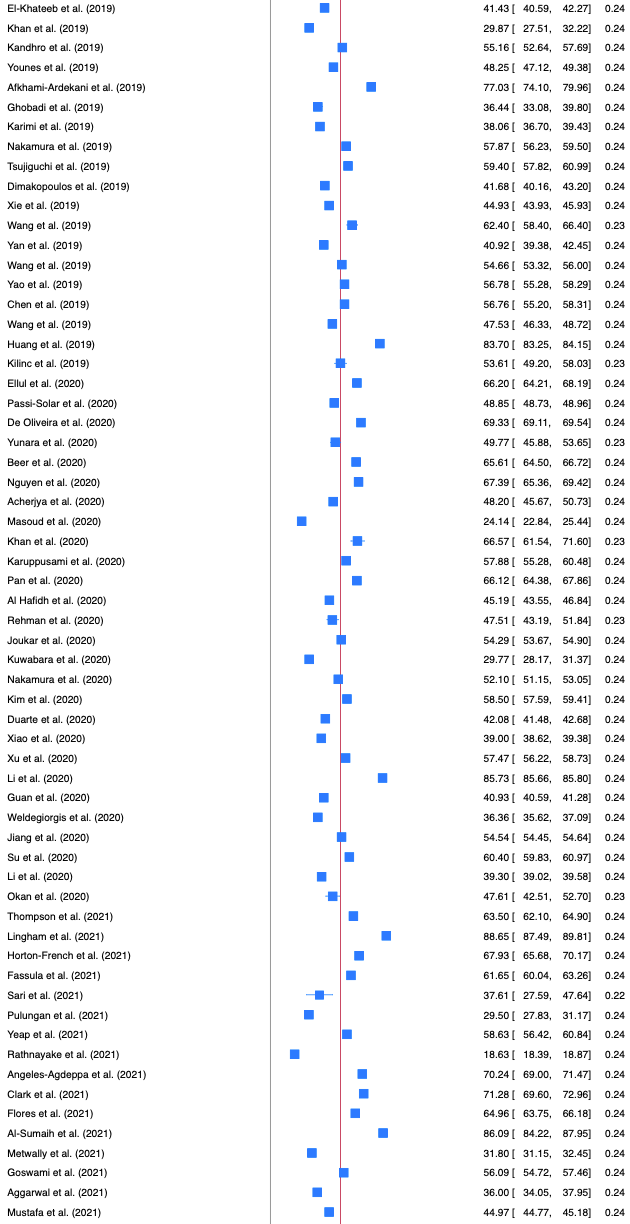

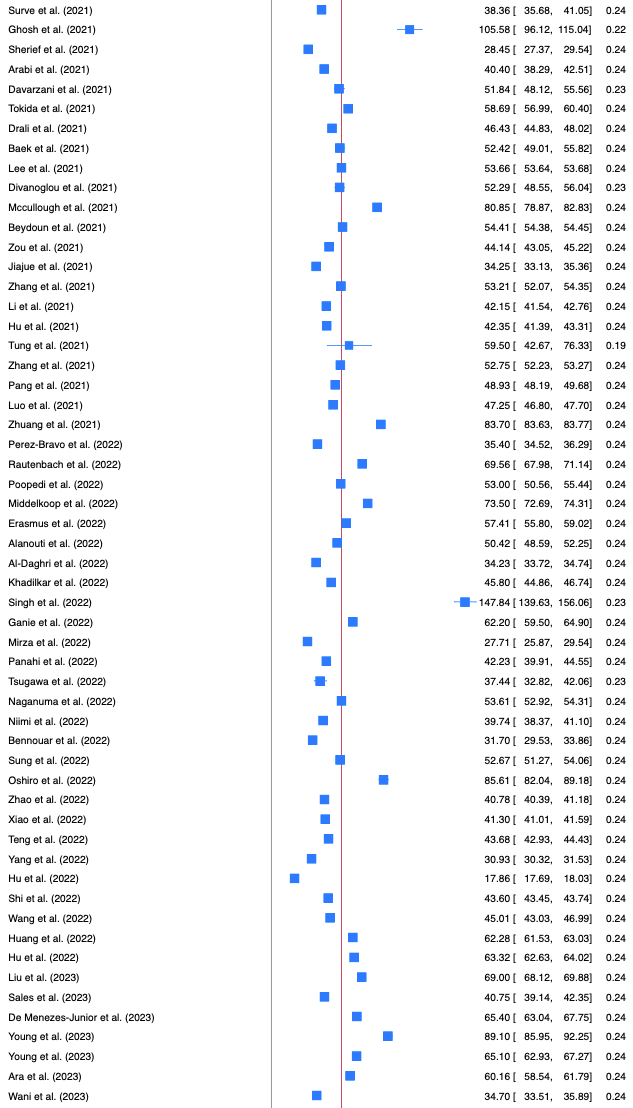

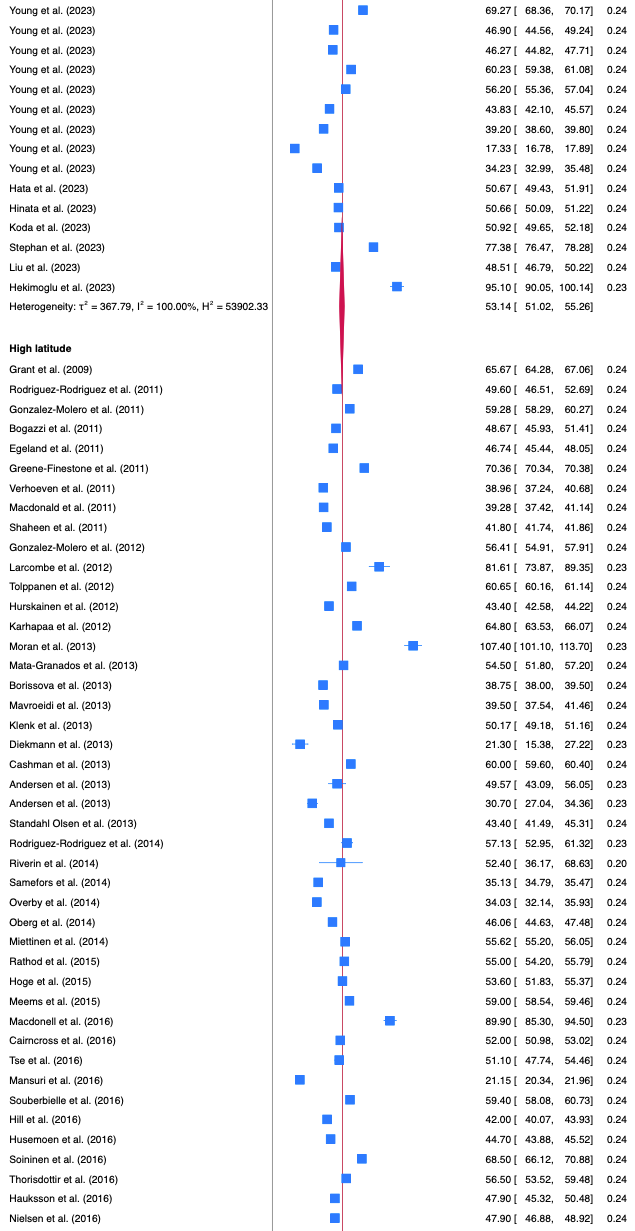

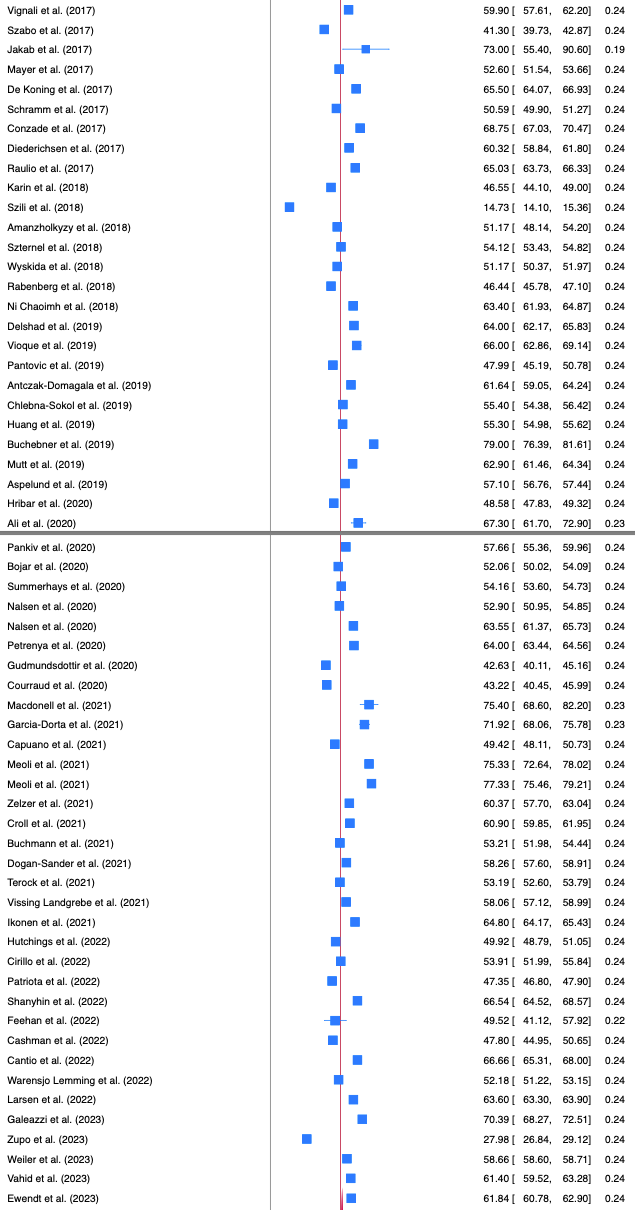

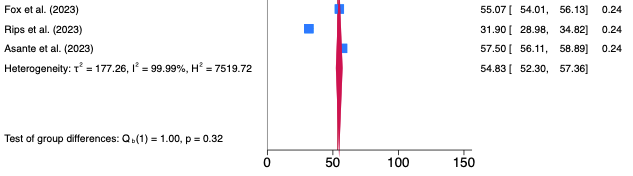


# Supplementary Figure 6a: Bubble plot of circulating 25-hydroxyvitamin D concentration by latitude in all participants


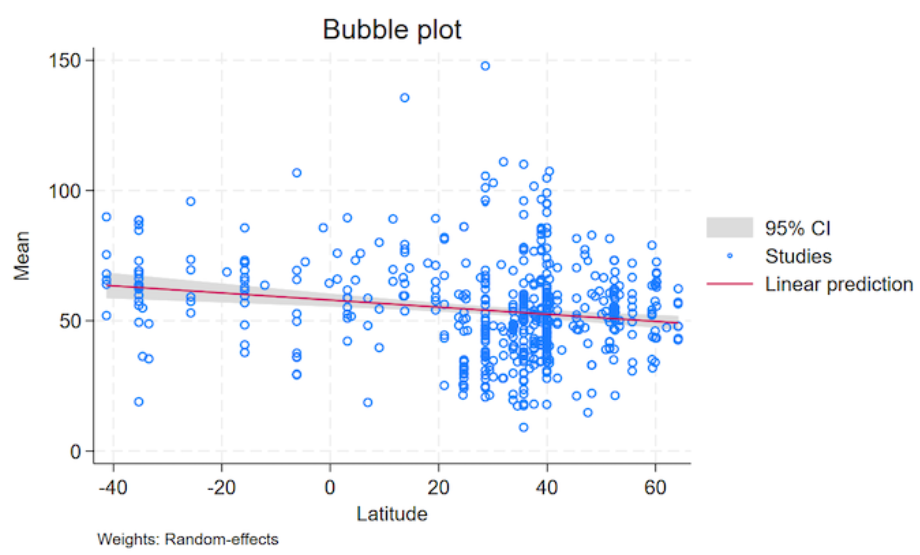


25(OH)D (nmol/L)

25(OH)D, 25-hydroxyvitamin D; CI, confidence interval


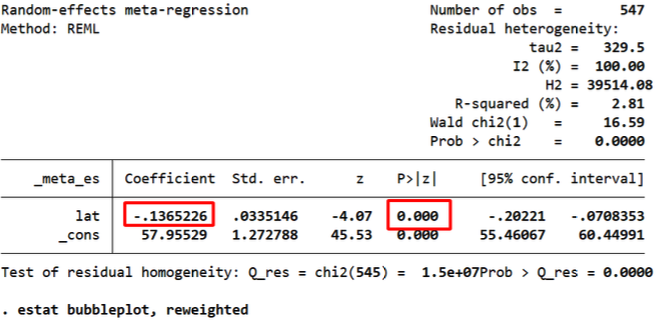


# Supplementary Figure 6b: Bubble plot of circulating 25-hydroxyvitamin D concentration by latitude in men


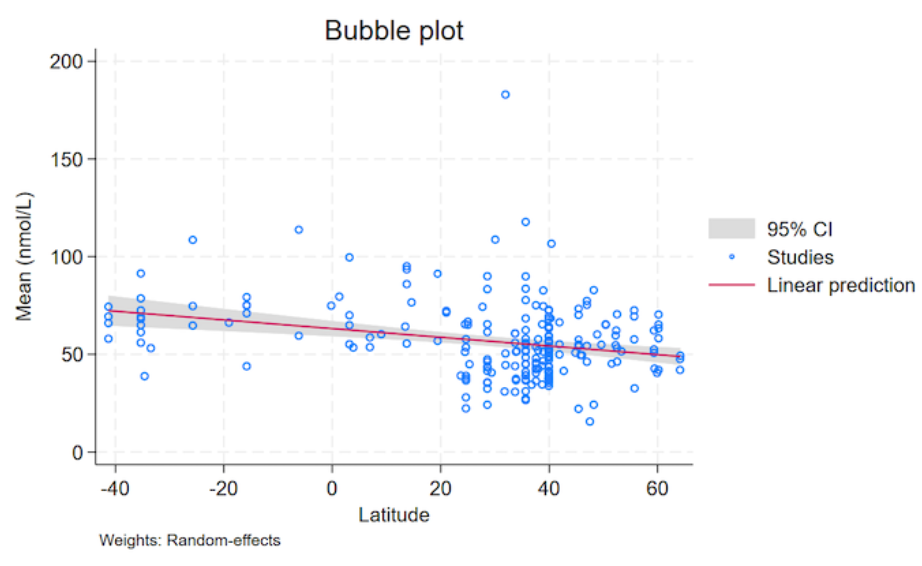


25(OH)D (nmol/L)

25(OH)D, 25-hydroxyvitamin D; CI, confidence interval

^
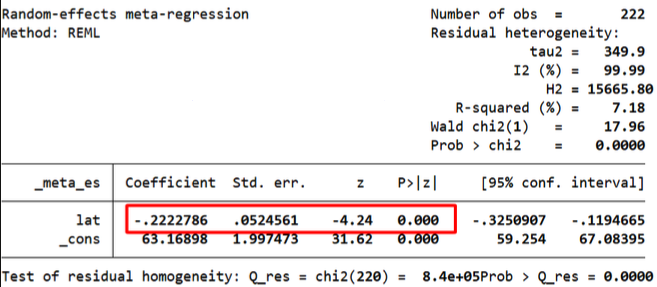
^

# Supplementary Figure 6c: Bubble plot of circulating 25-hydroxyvitamin D concentration by latitude in women


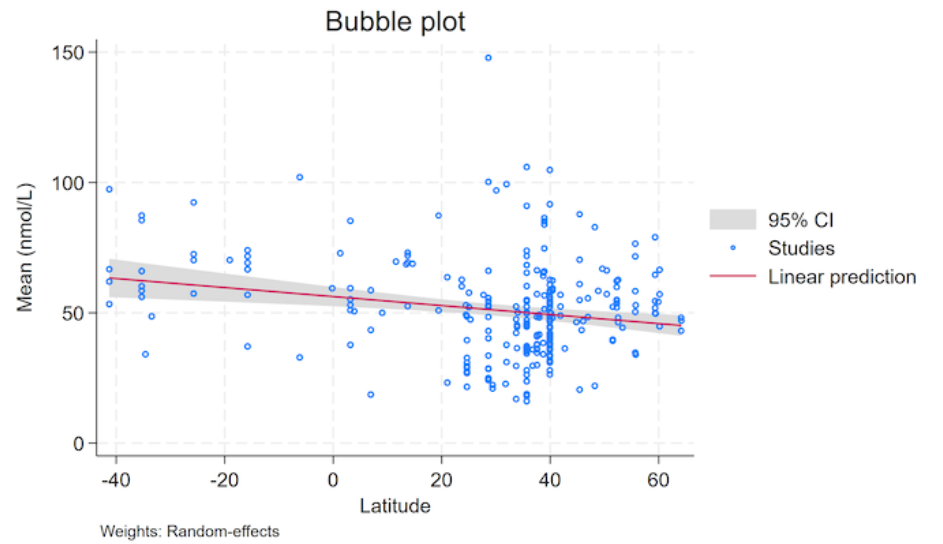


25(OH)D (nmol/L)

25(OH)D, 25-hydroxyvitamin D; CI, confidence interval


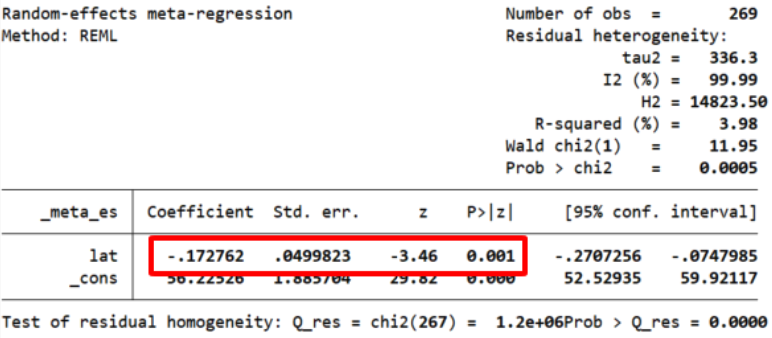


# Supplementary Figure 7: Forest plot for pooled mean circulating 25-hydroxyvitamin D concentration in men


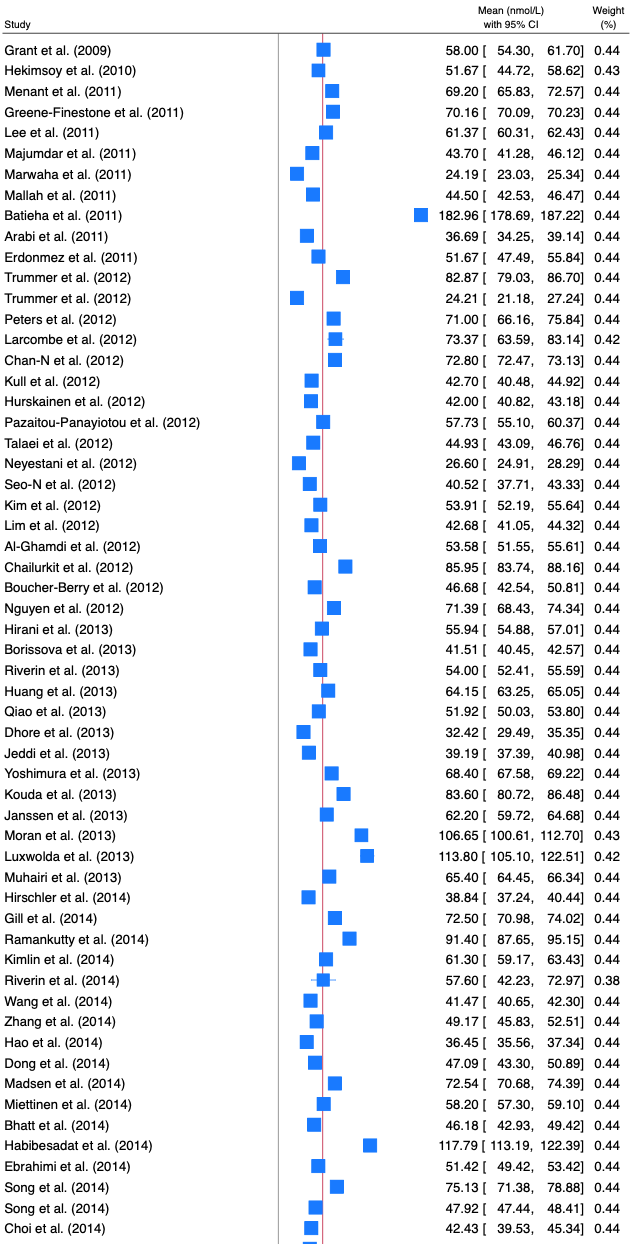


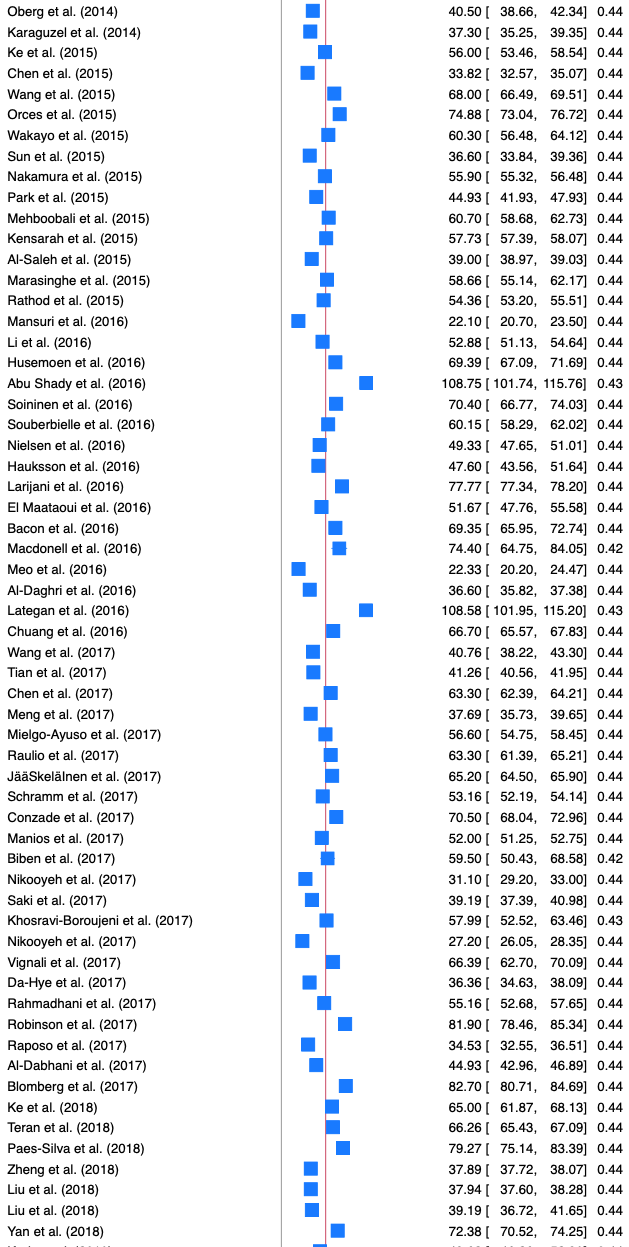


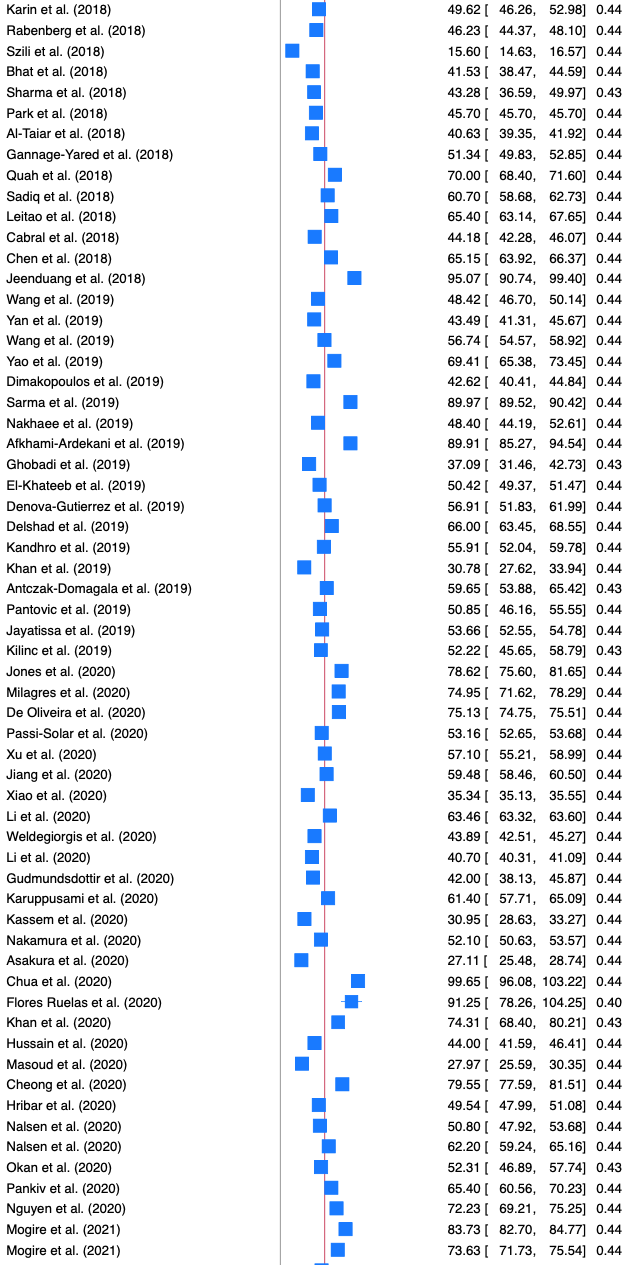


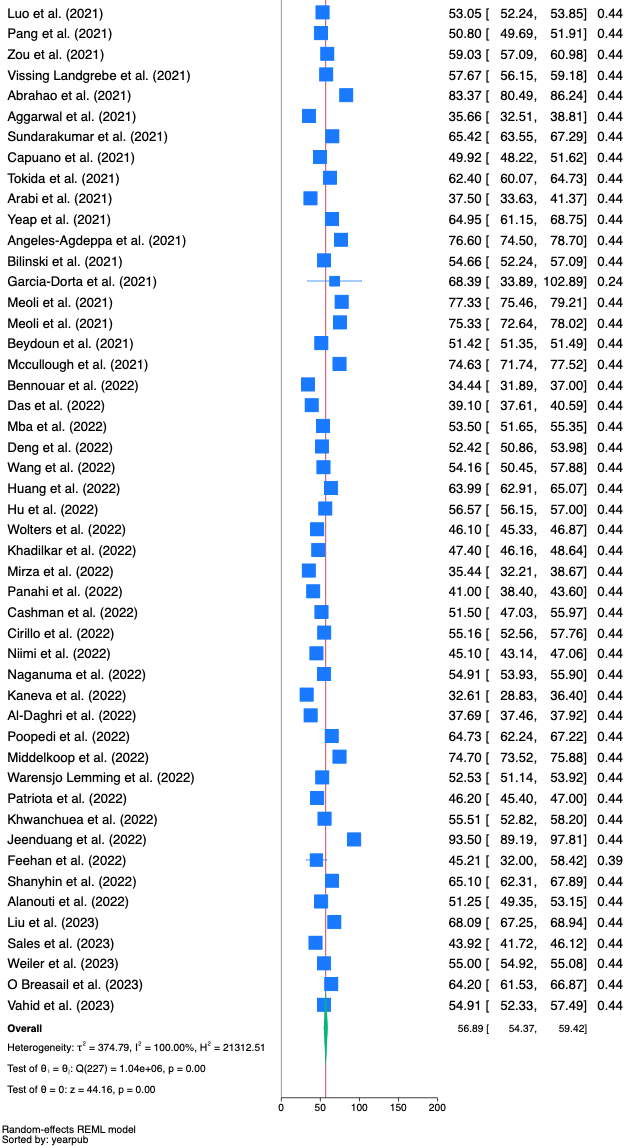


# Supplementary Figure 8: Forest plot for pooled mean circulating 25-hydroxyvitamin D concentration in women


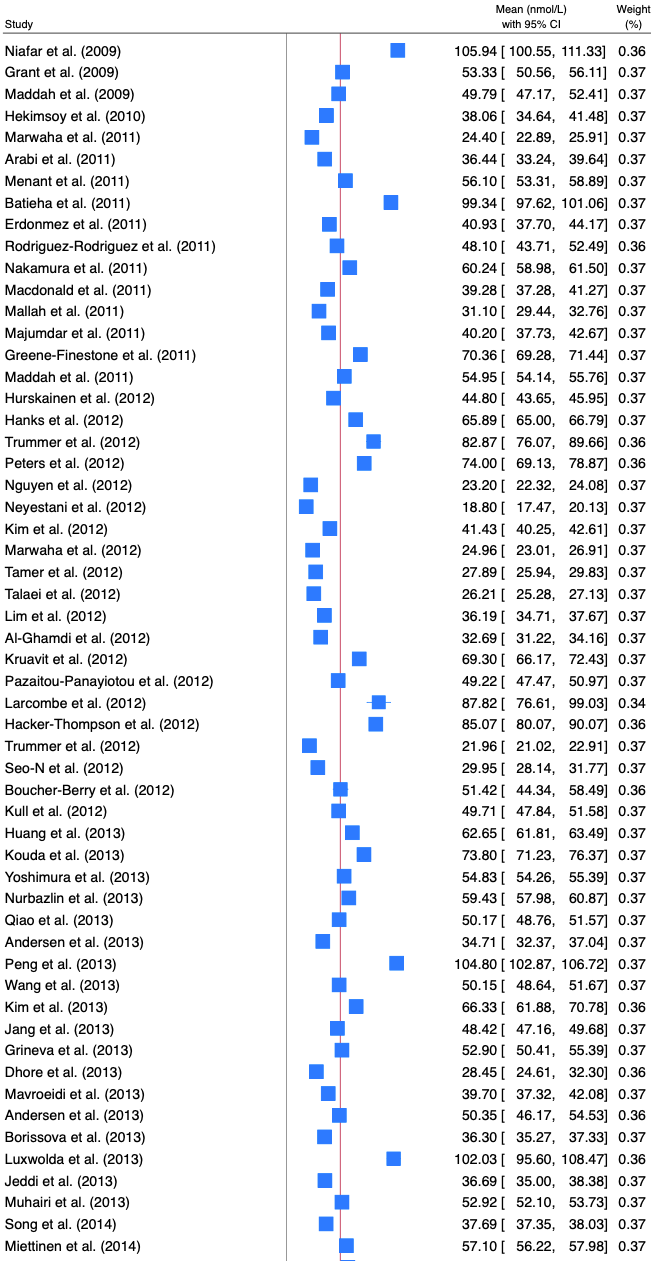


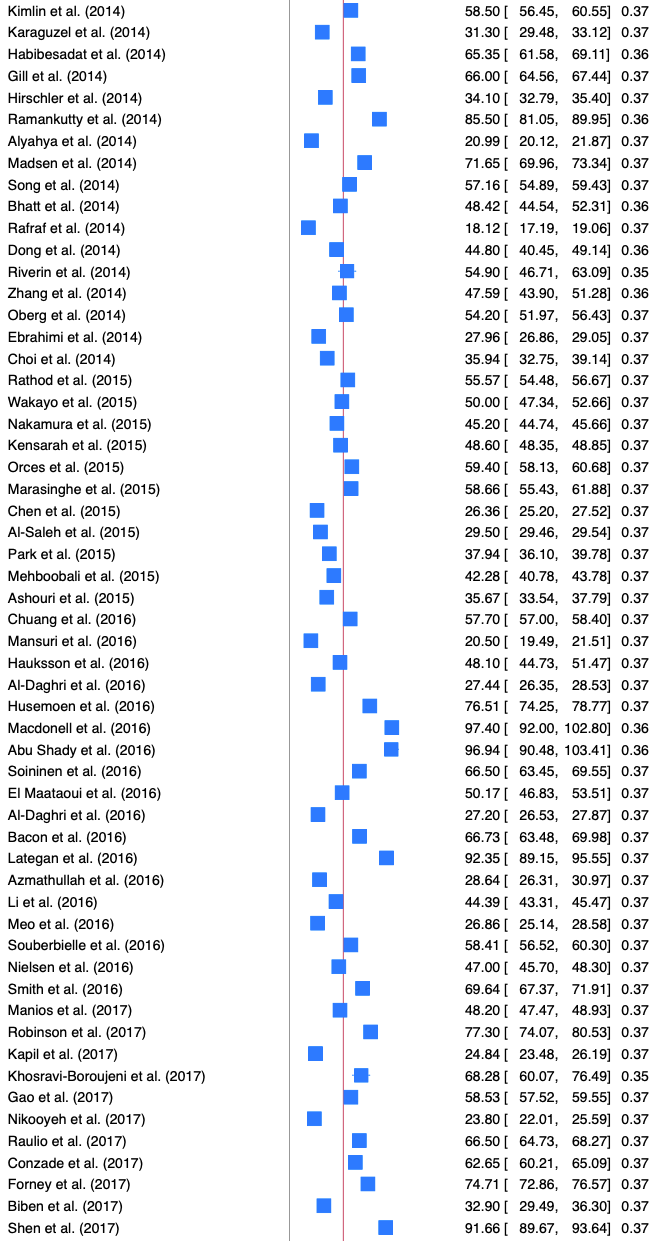


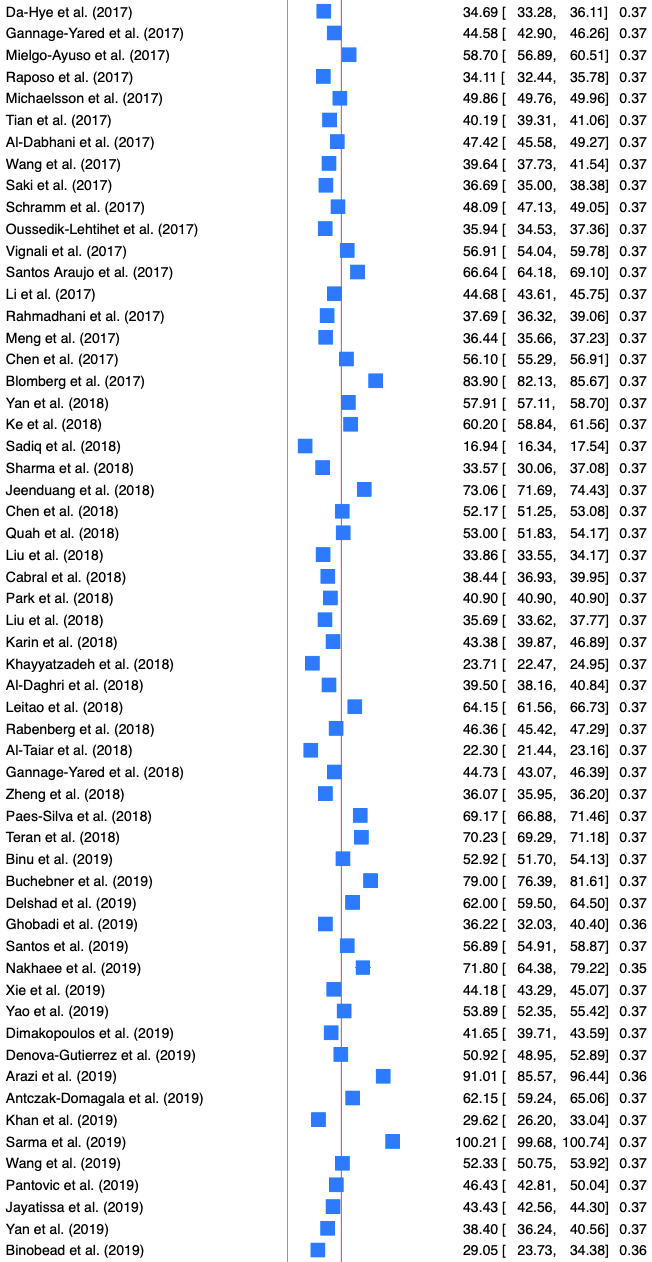


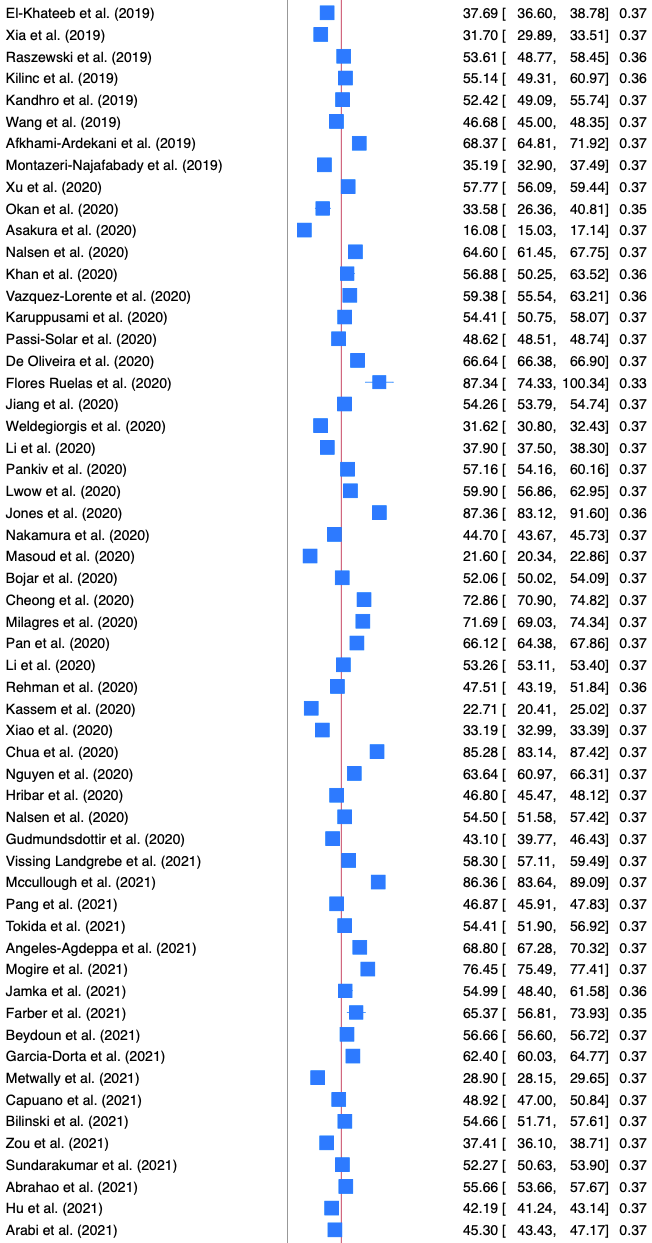


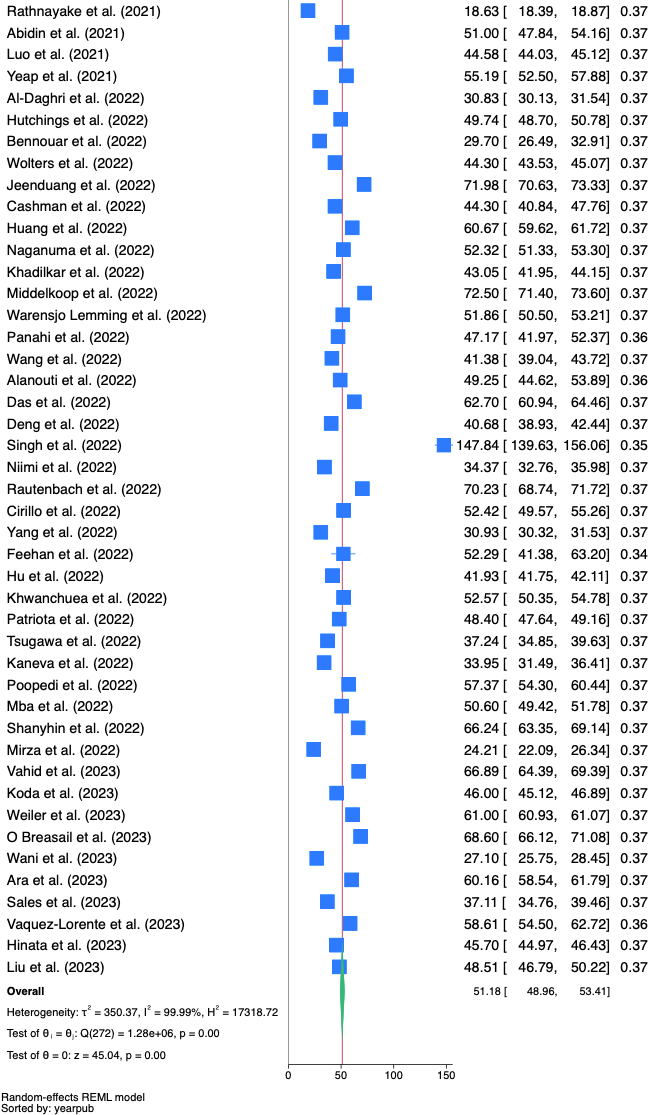


# Supplementary Figure 9: Forest plot for pooled mean circulating 25-hydroxyvitamin D concentration by adults and children


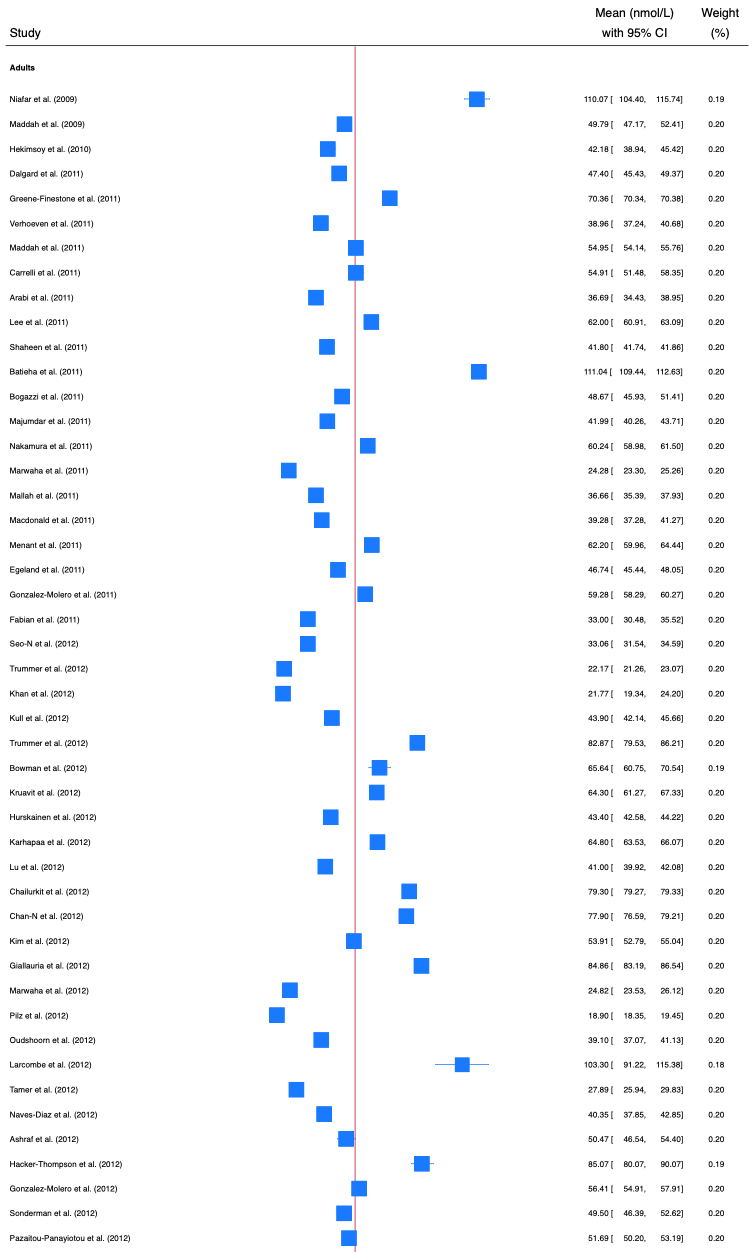


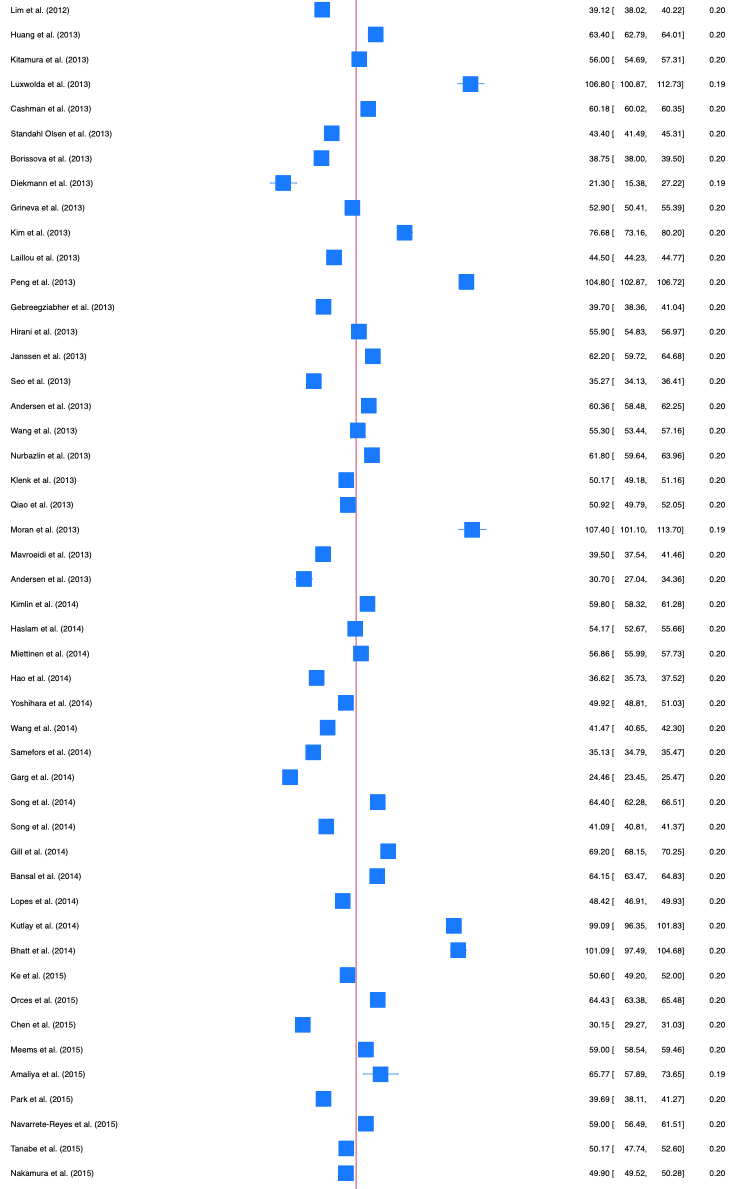


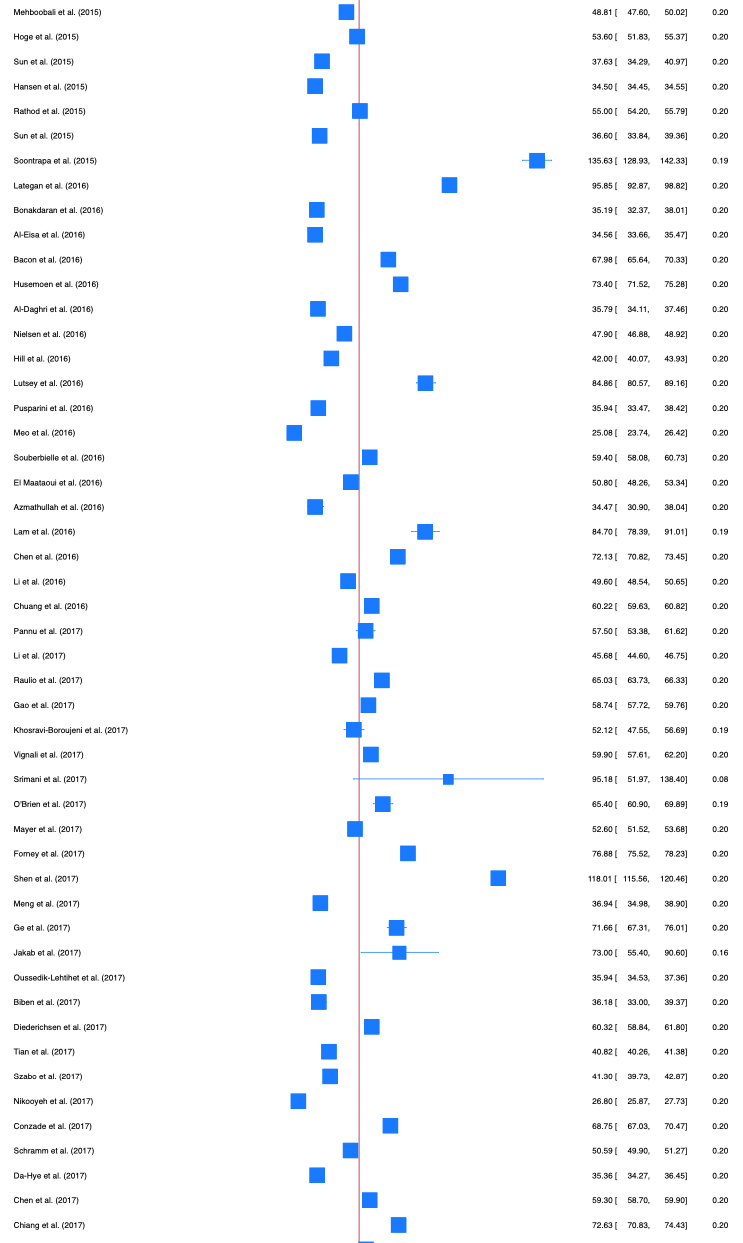


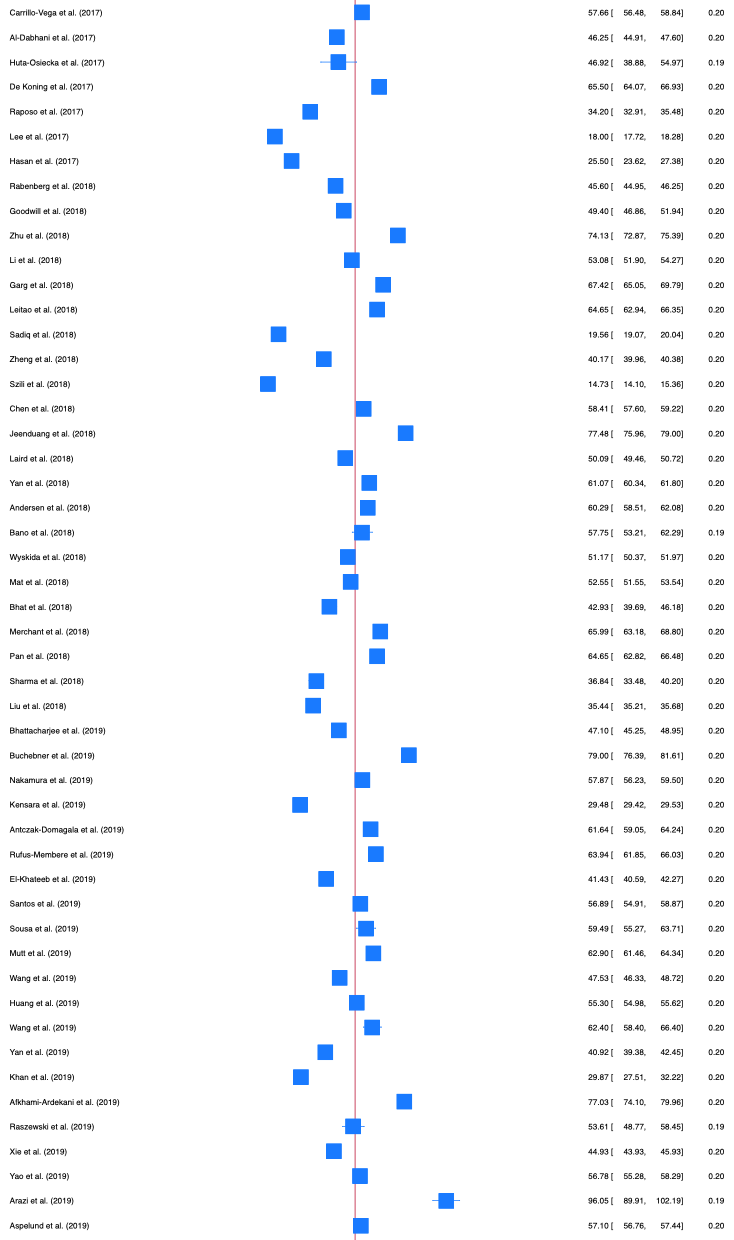


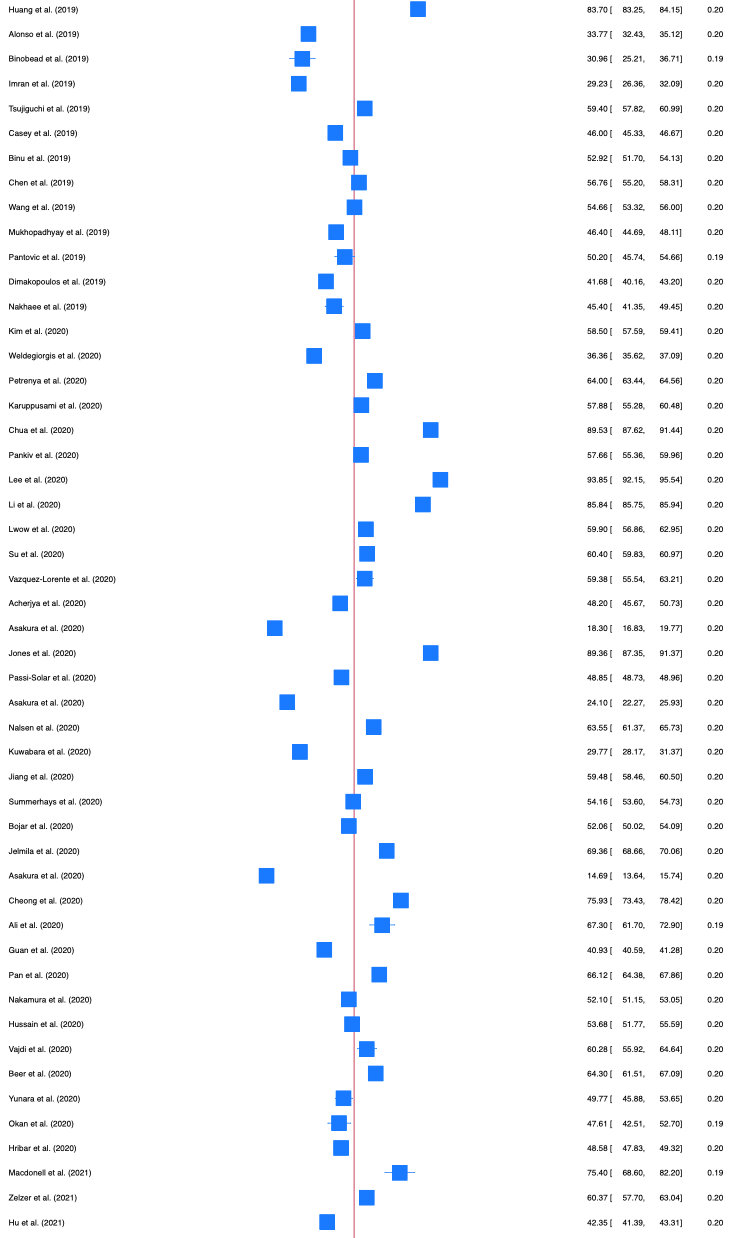


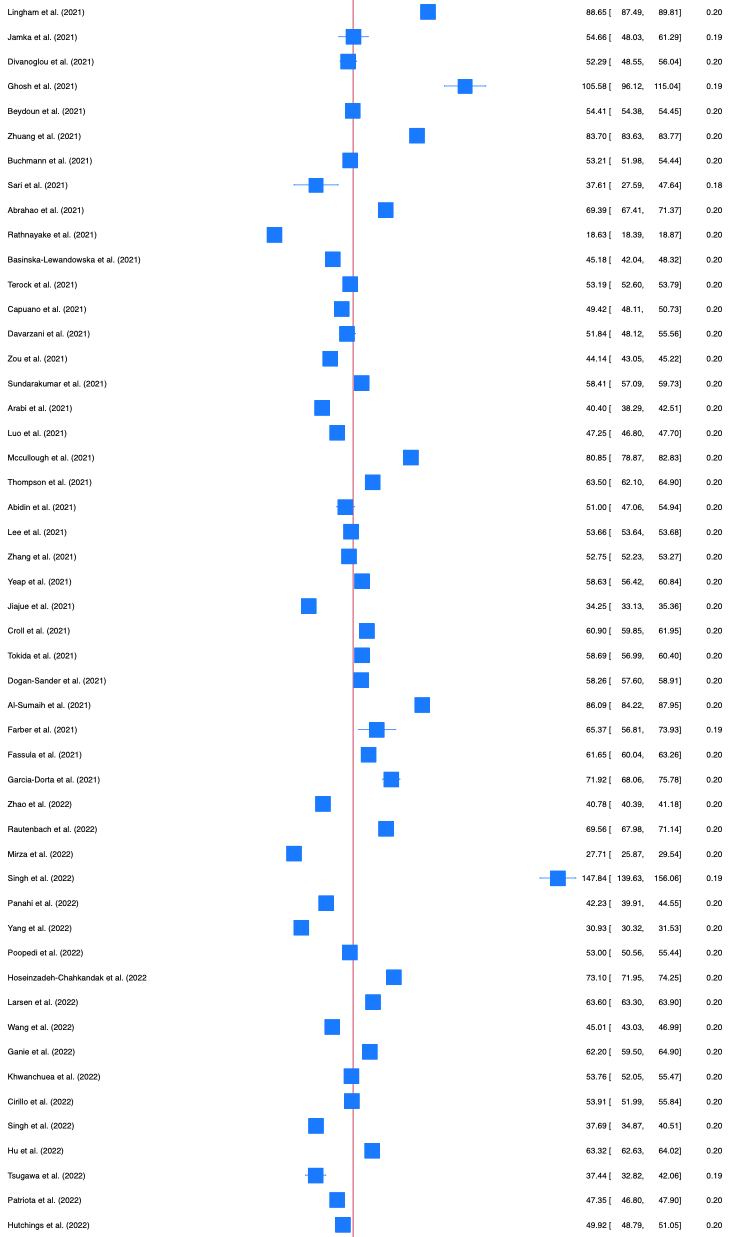


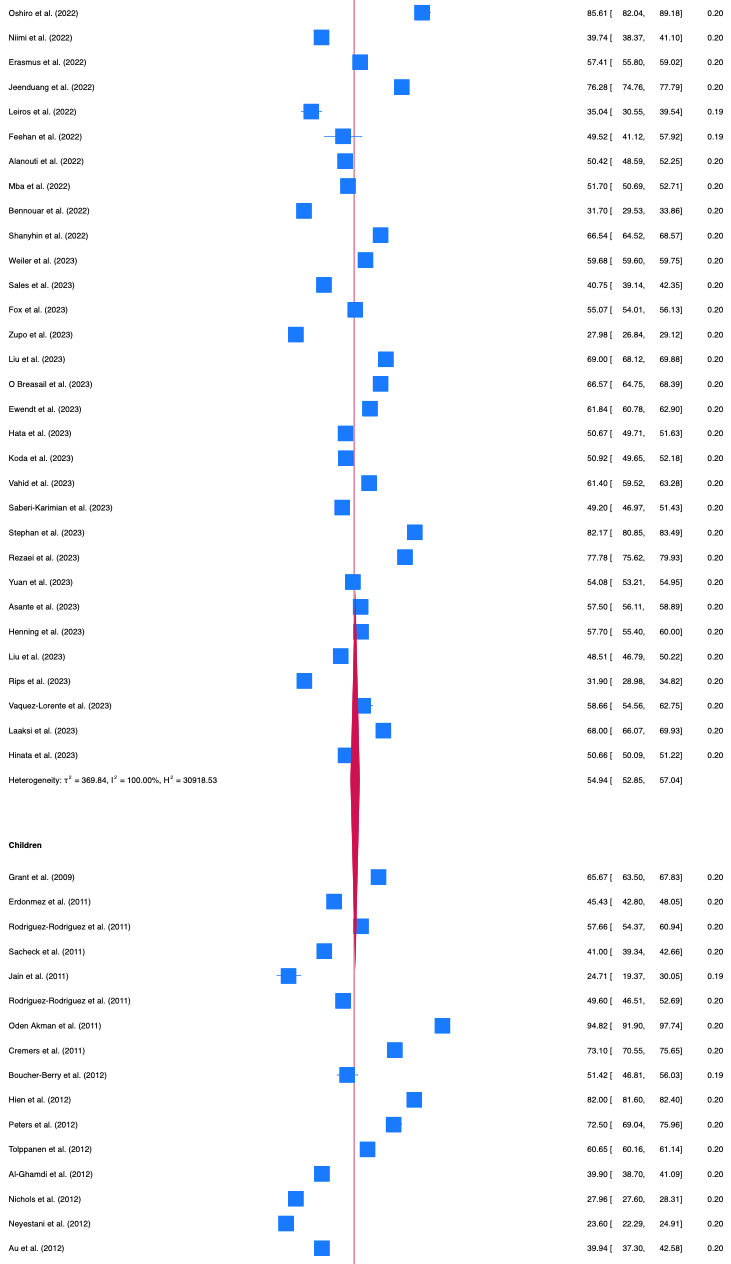


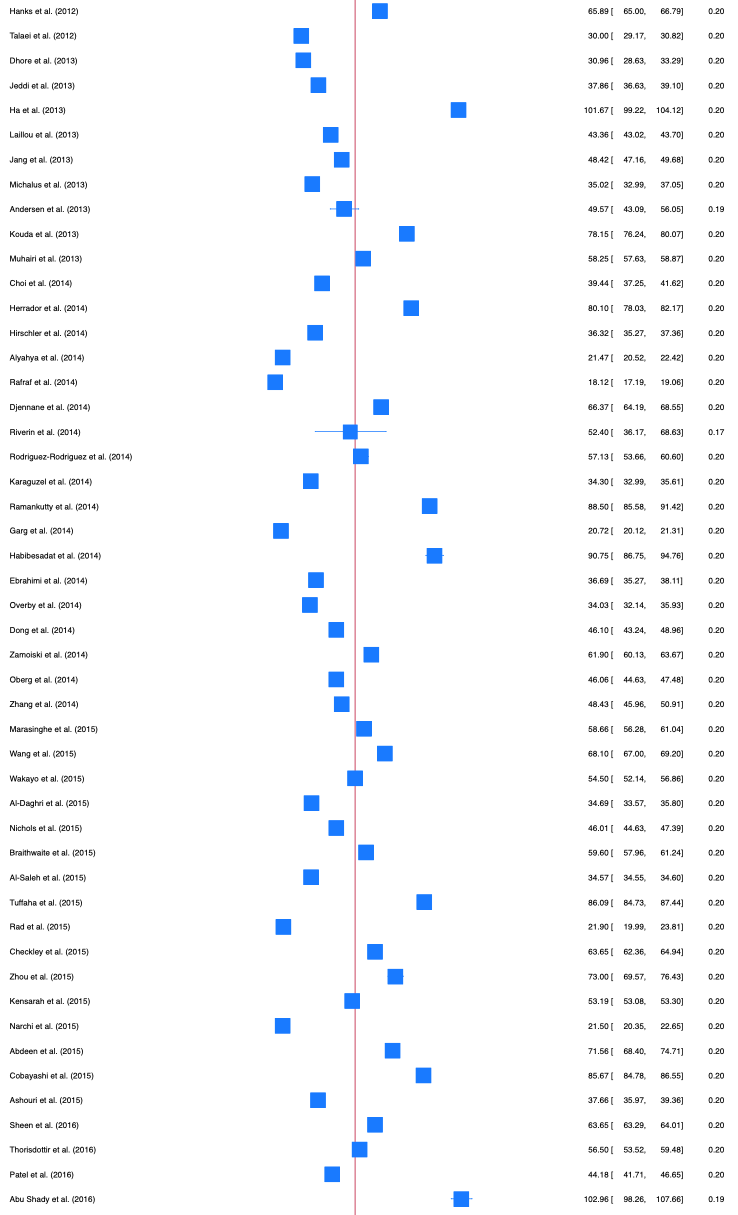


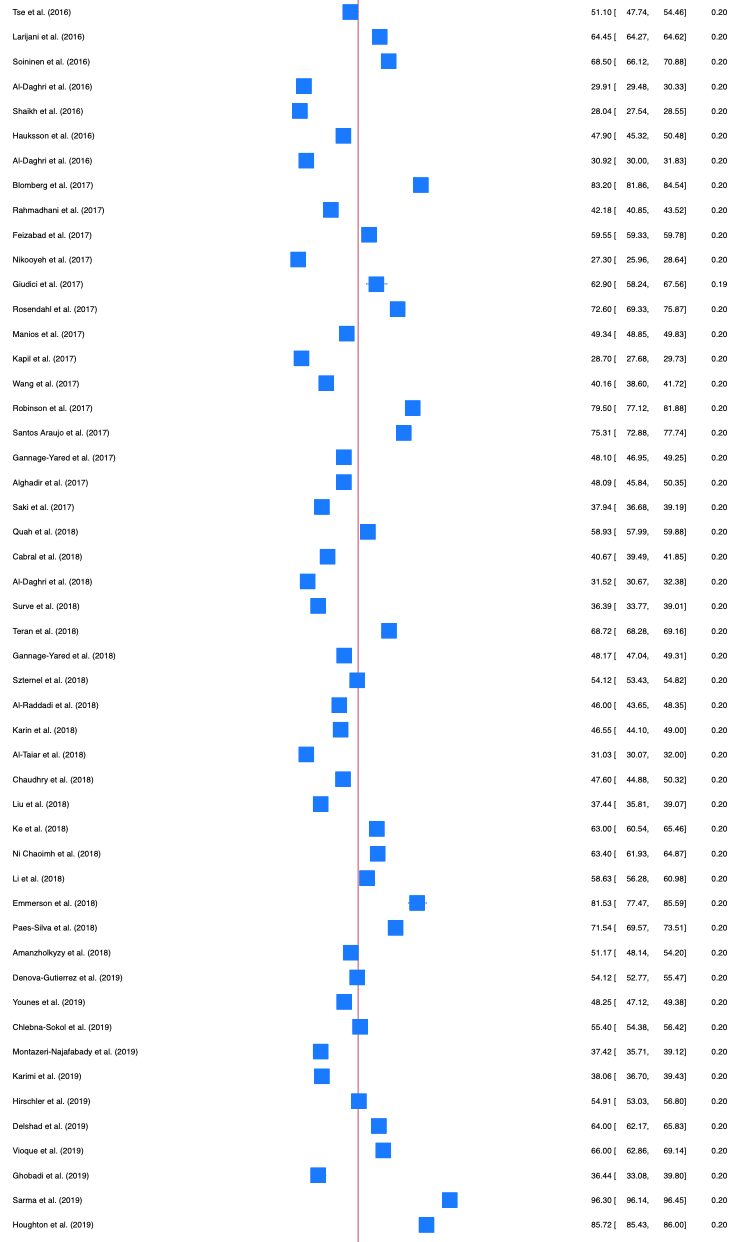


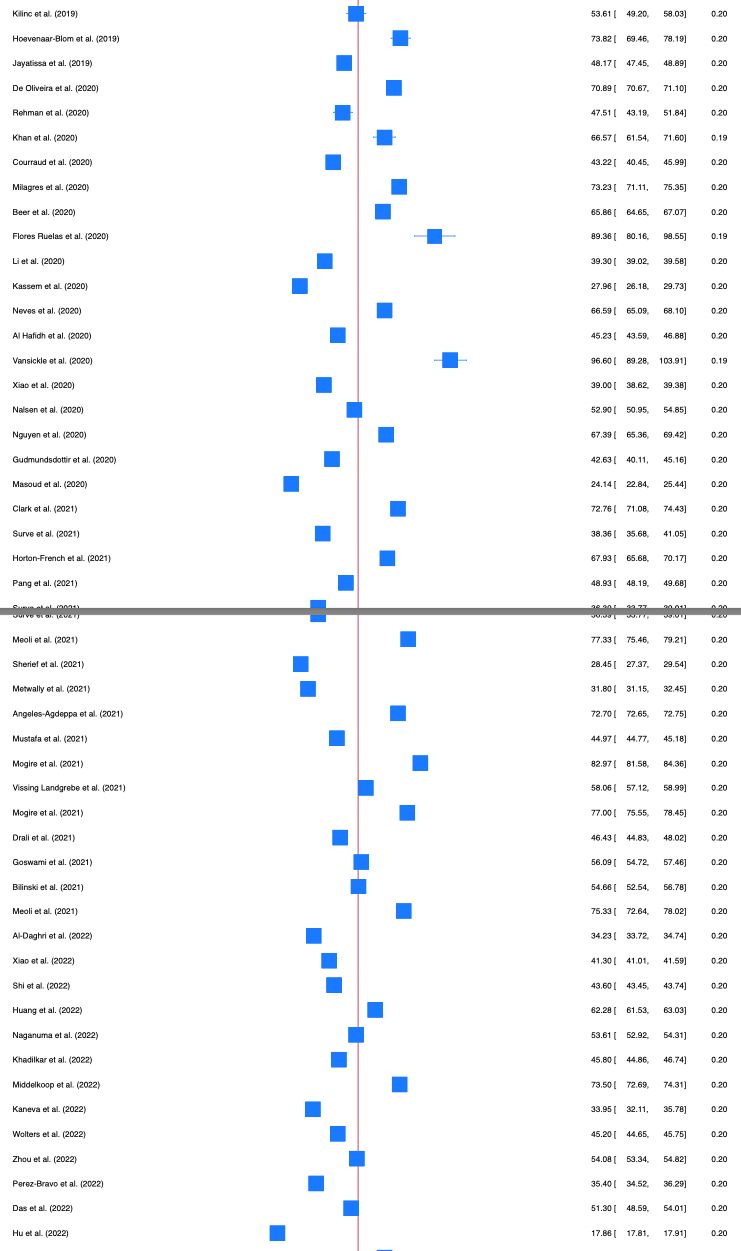


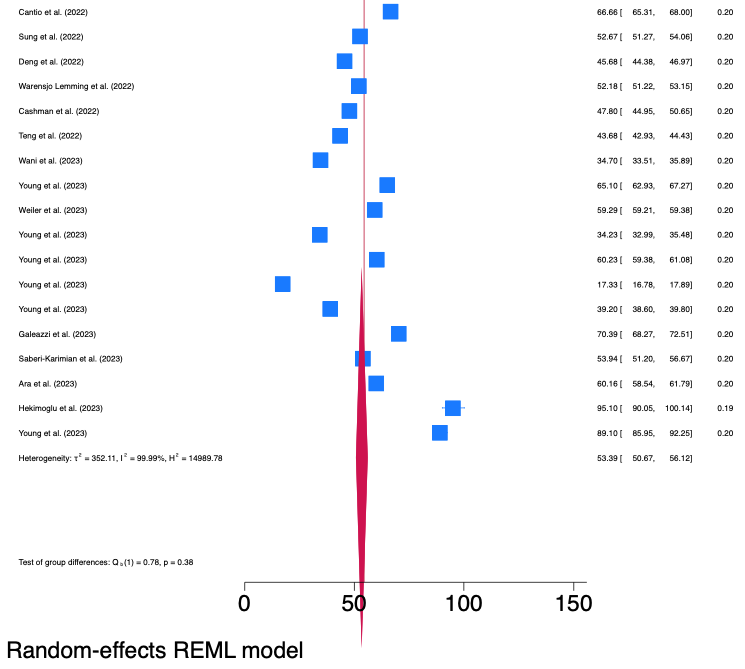


# Supplementary Figure 10: Forest plot for pooled mean circulating 25-hydroxyvitamin D concentration by season


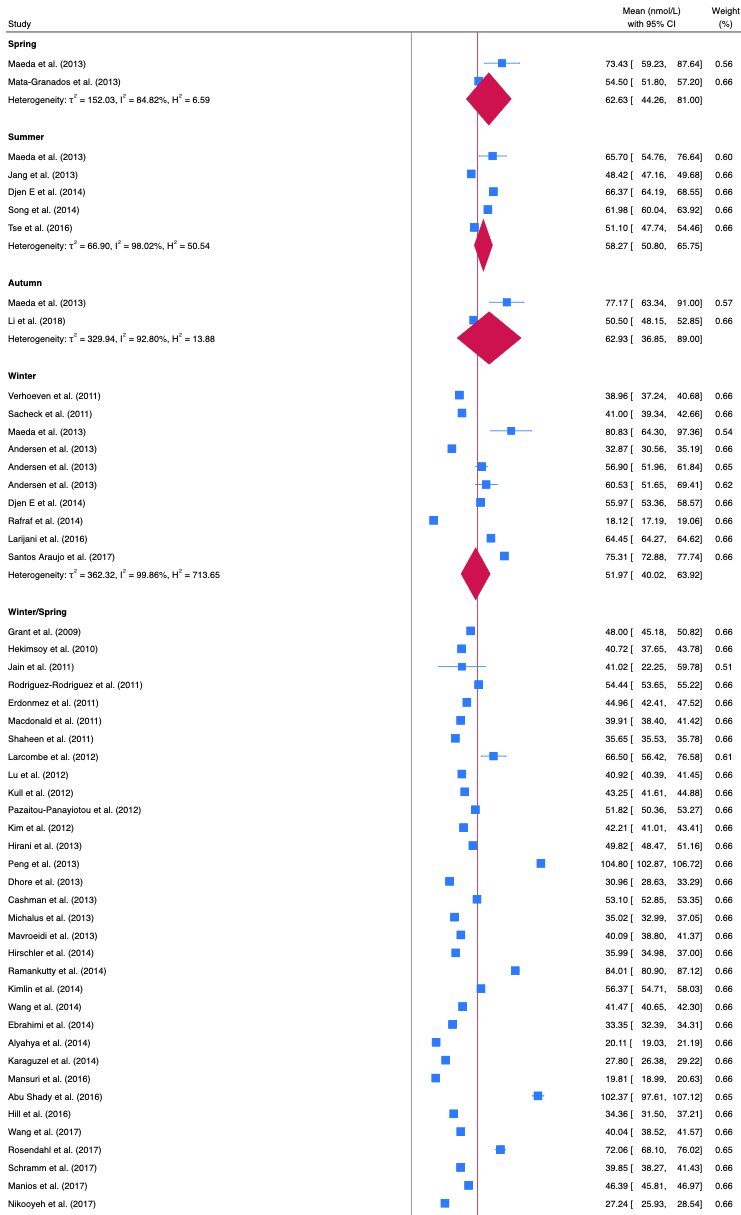


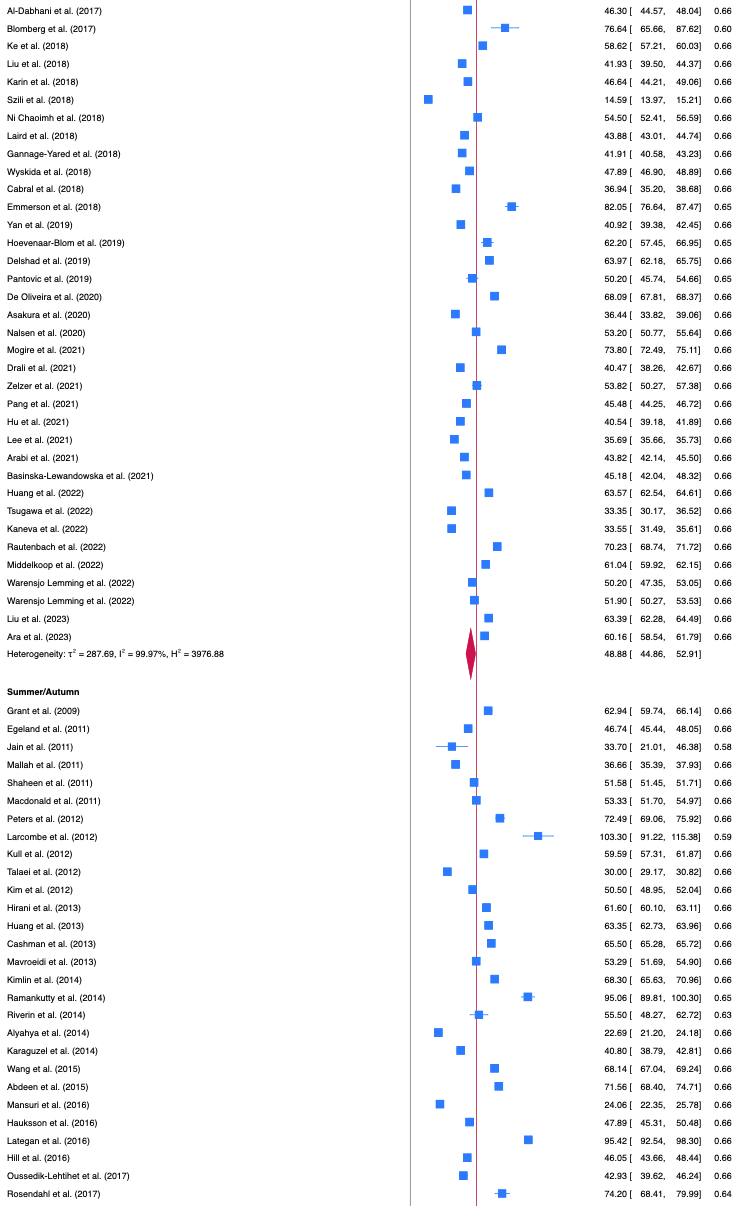

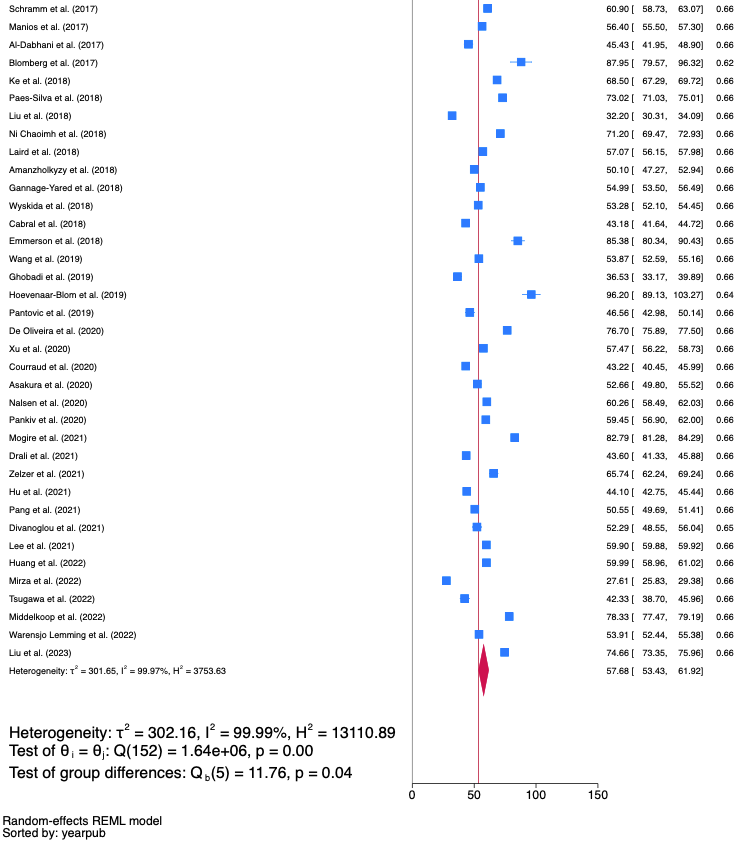


# Supplementary Figure 11: Forest plot for pooled mean circulating 25-hydroxyvitamin D concentration by use of certified assay/harmonised data


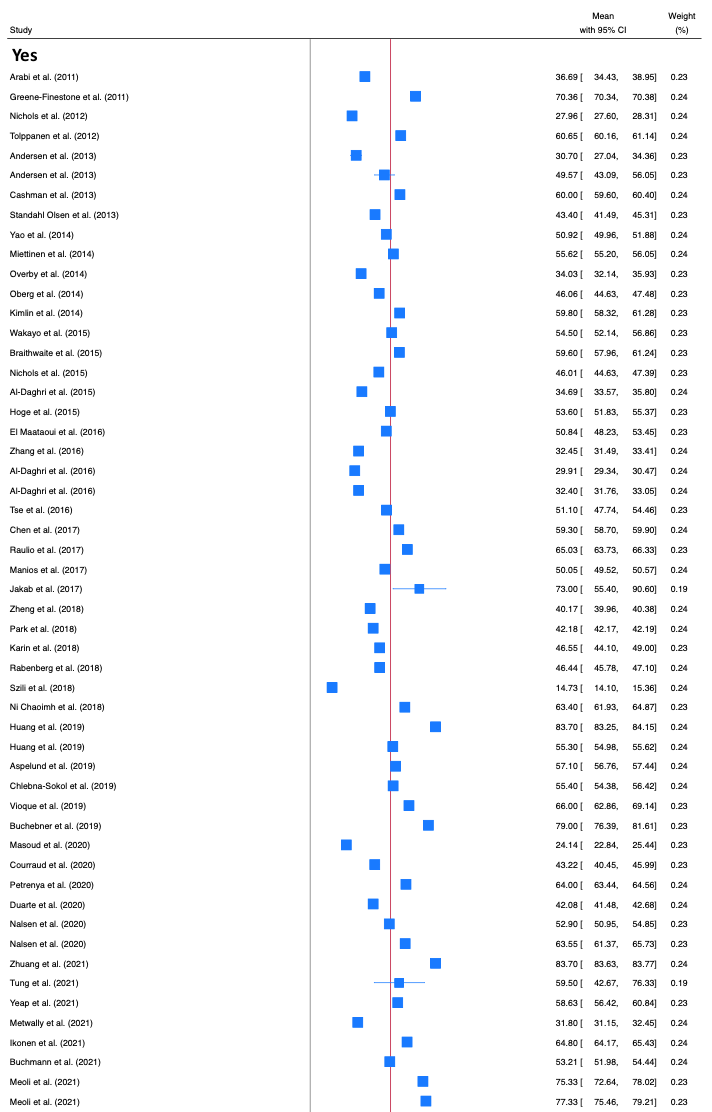


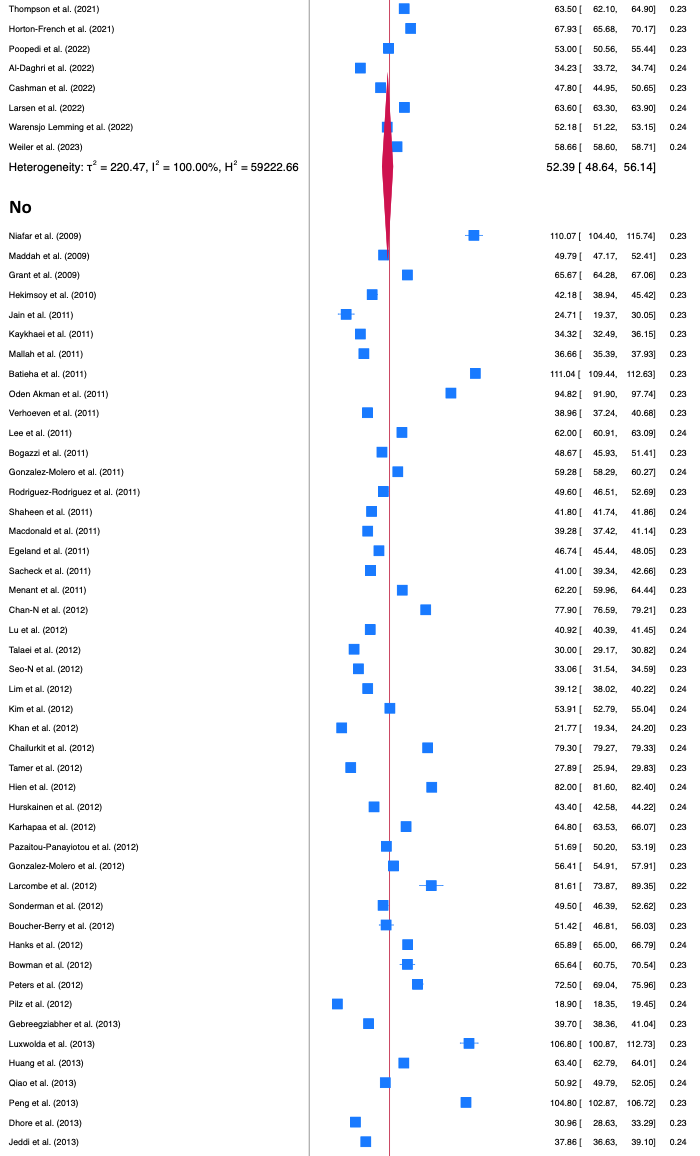


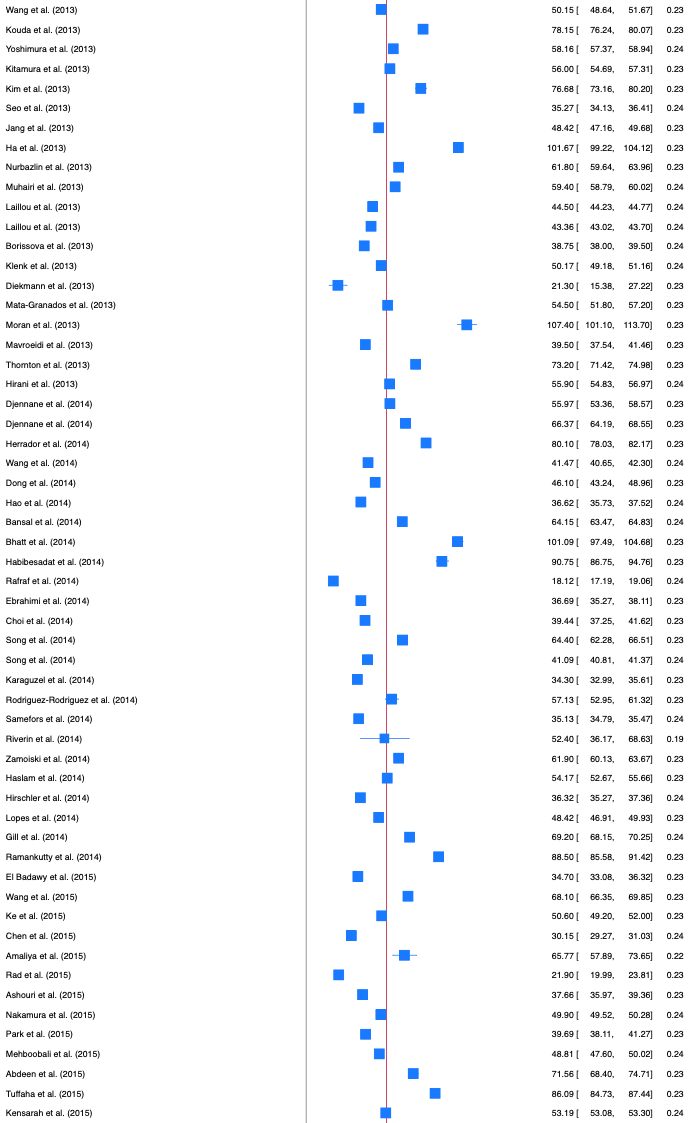

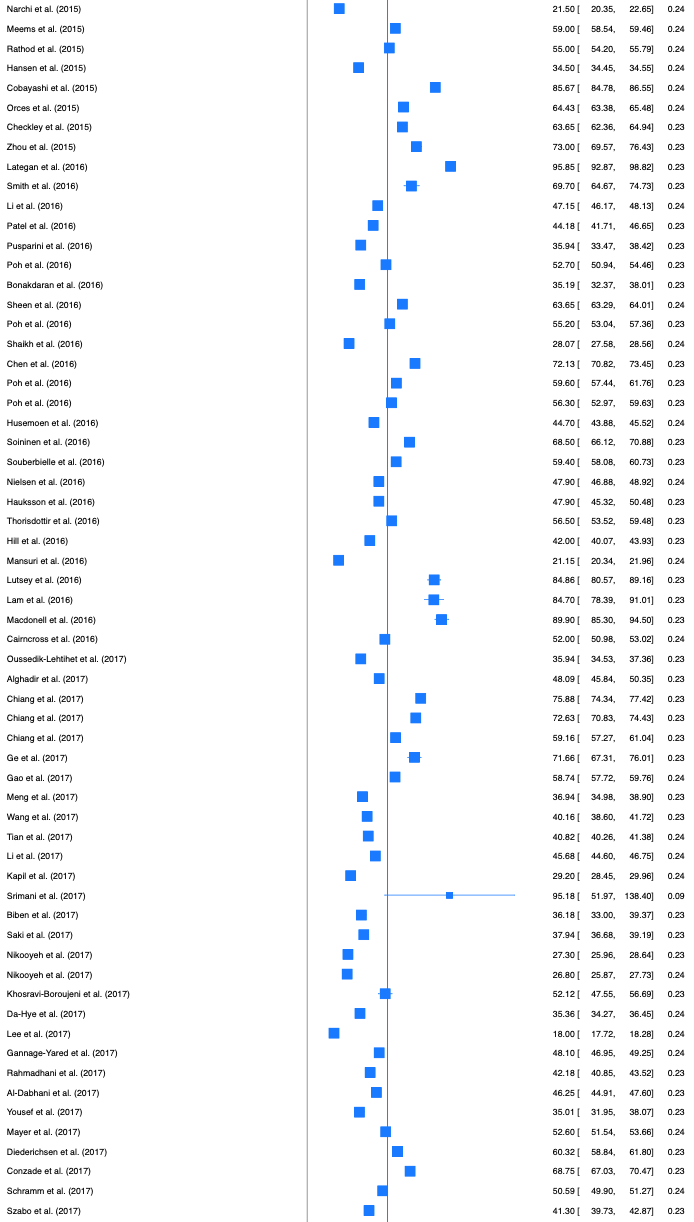


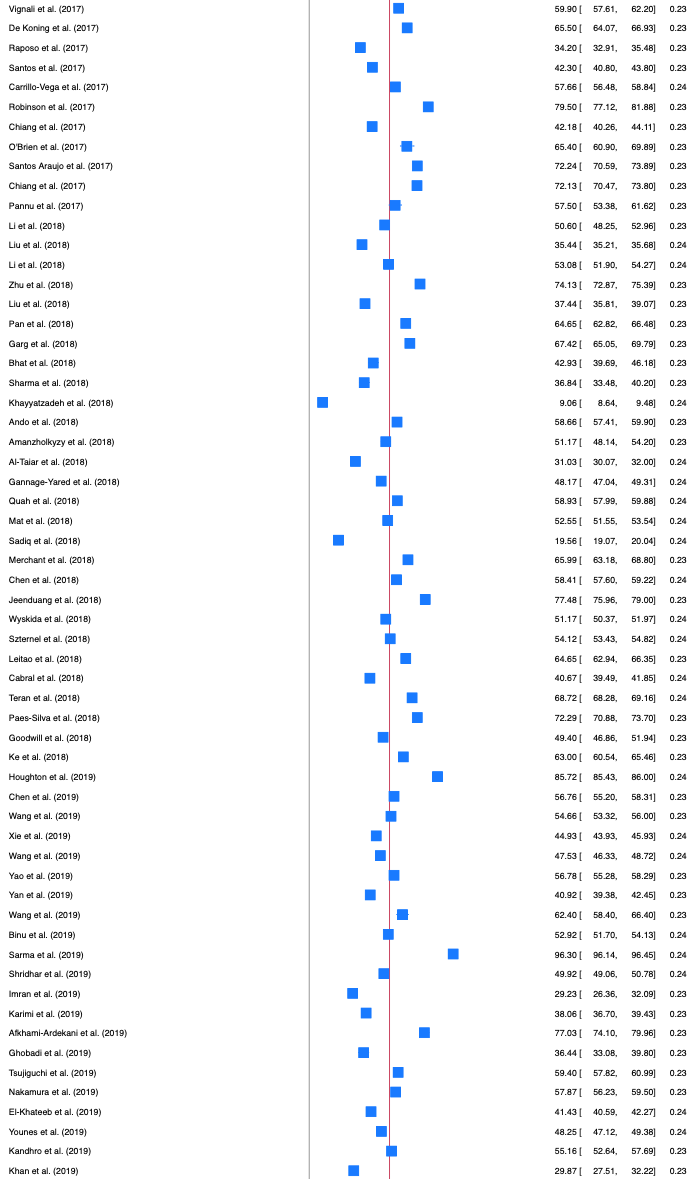


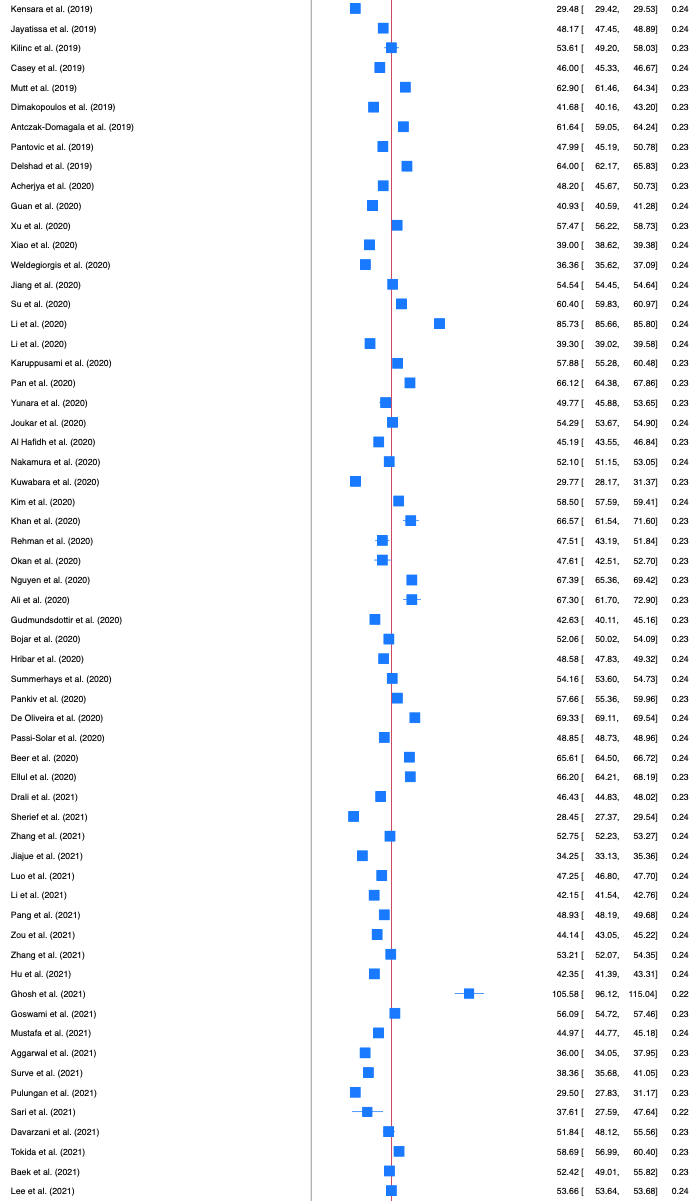

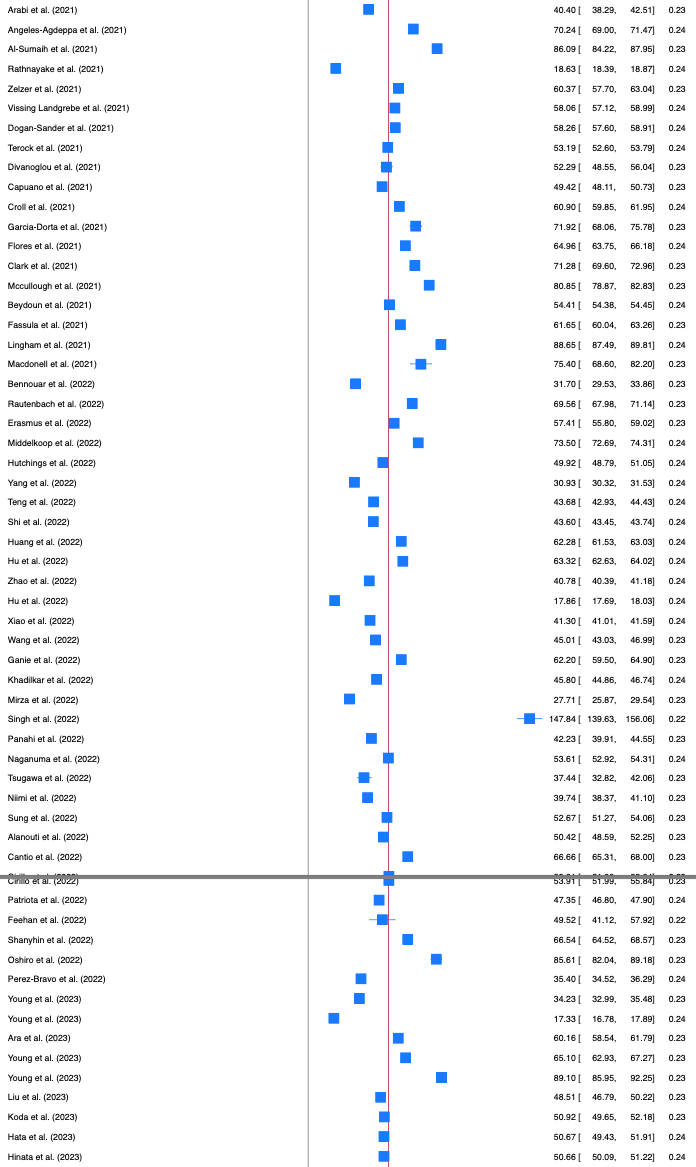

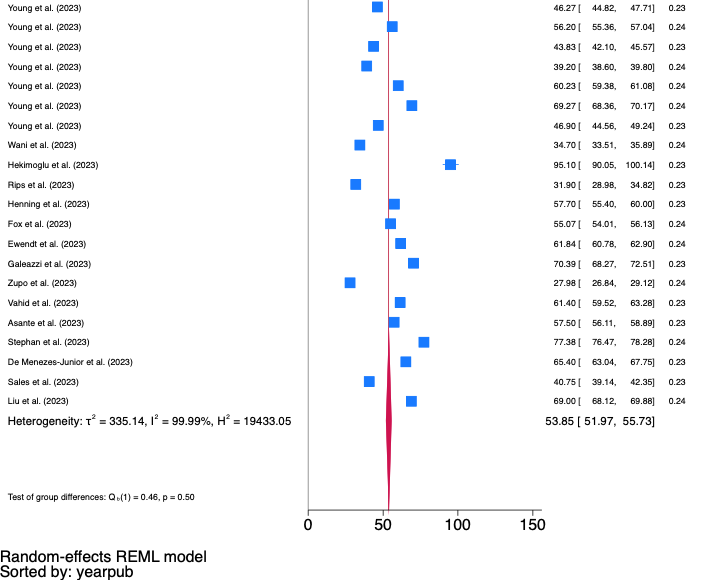


# Supplementary Figure 12: Forest plot for pooled mean circulating 25-hydroxyvitamin D concentration by study quality


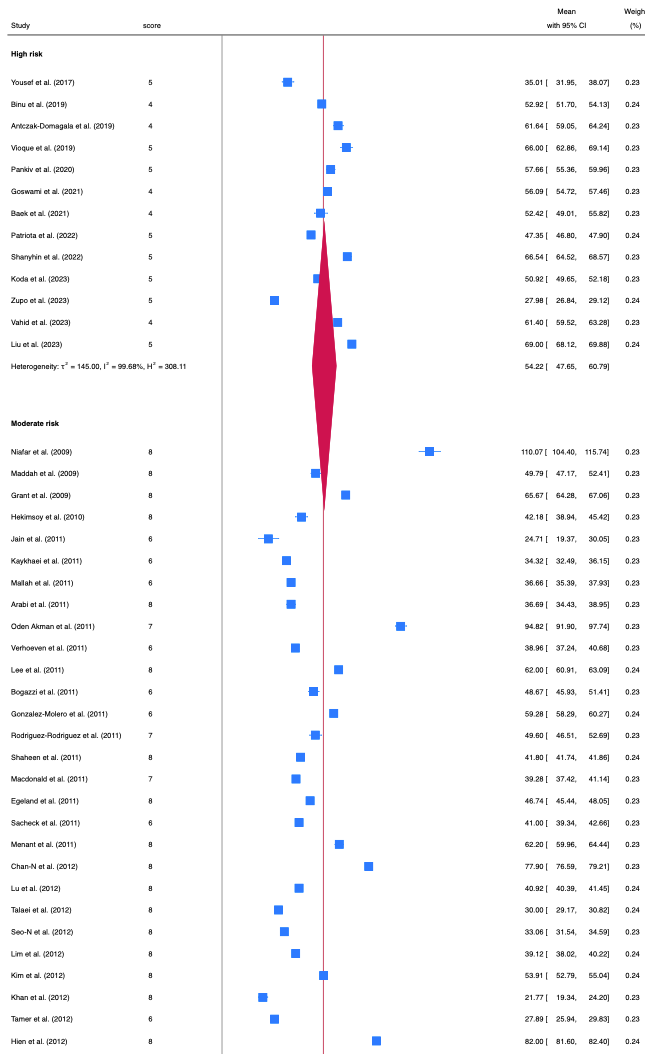


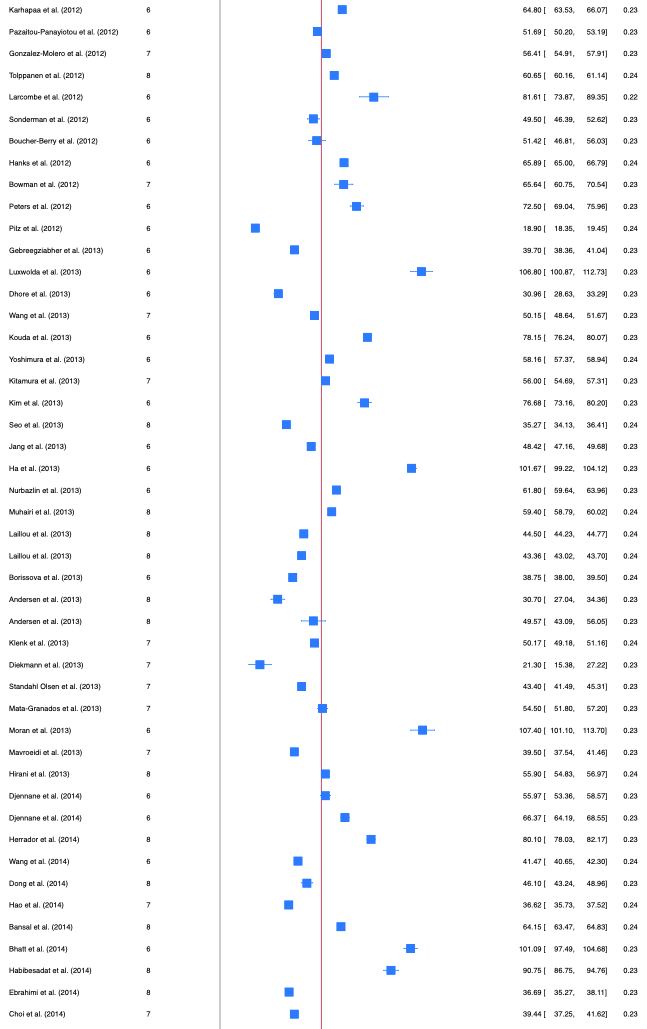

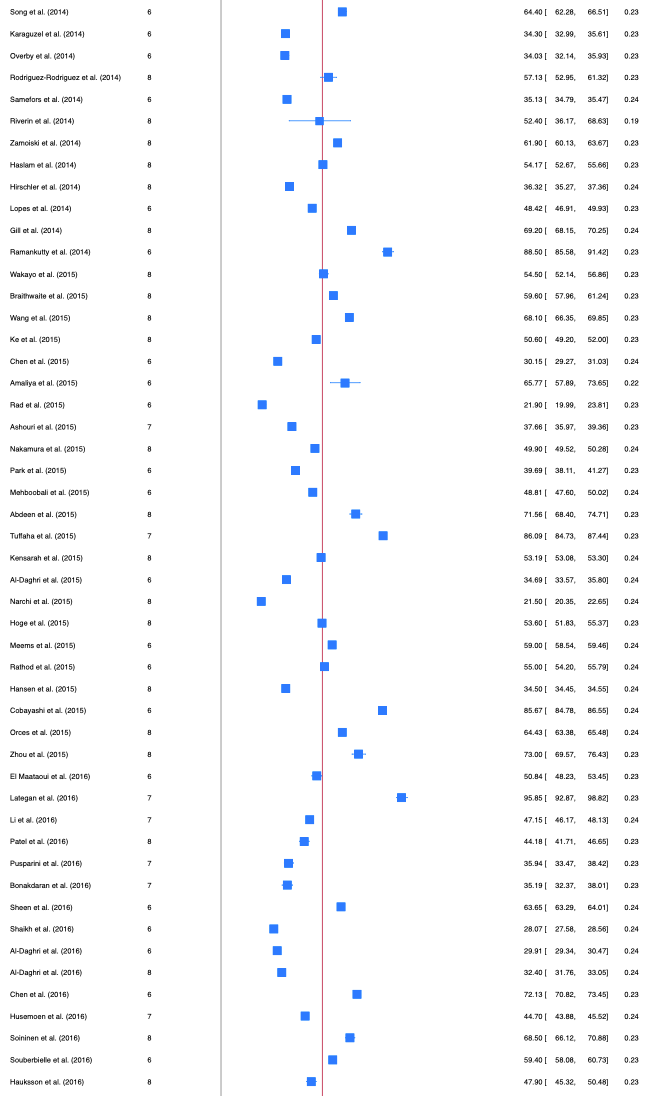


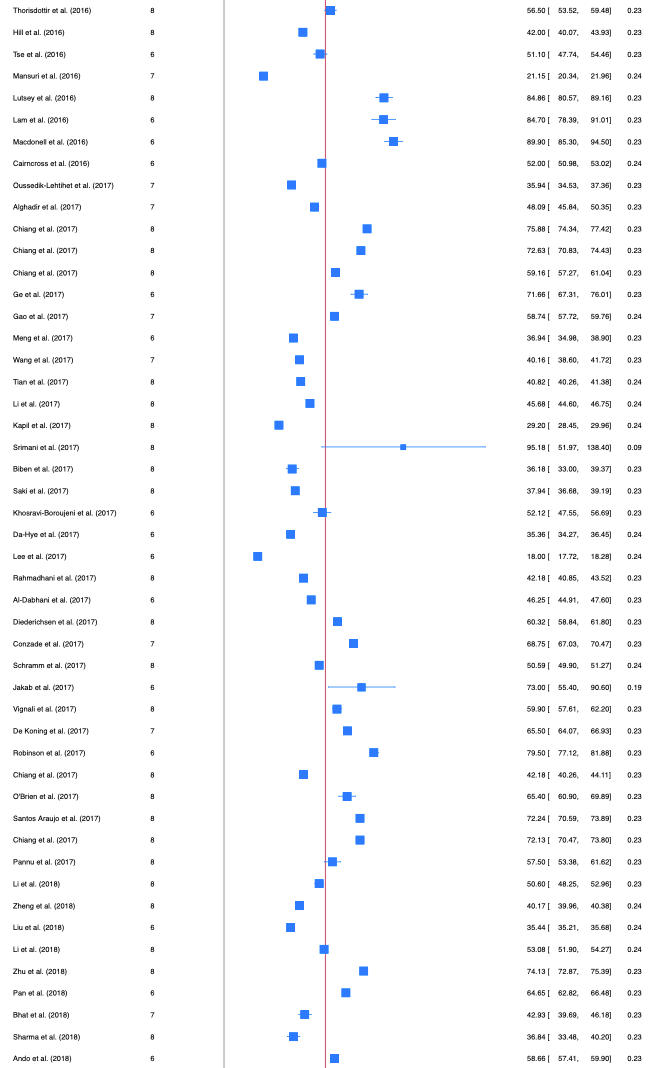


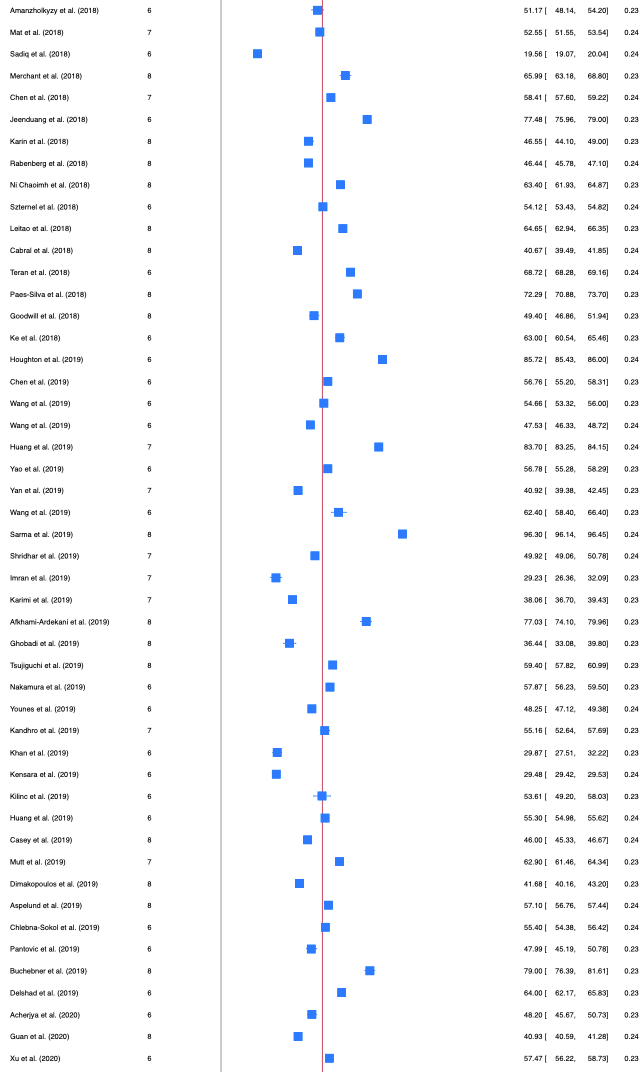


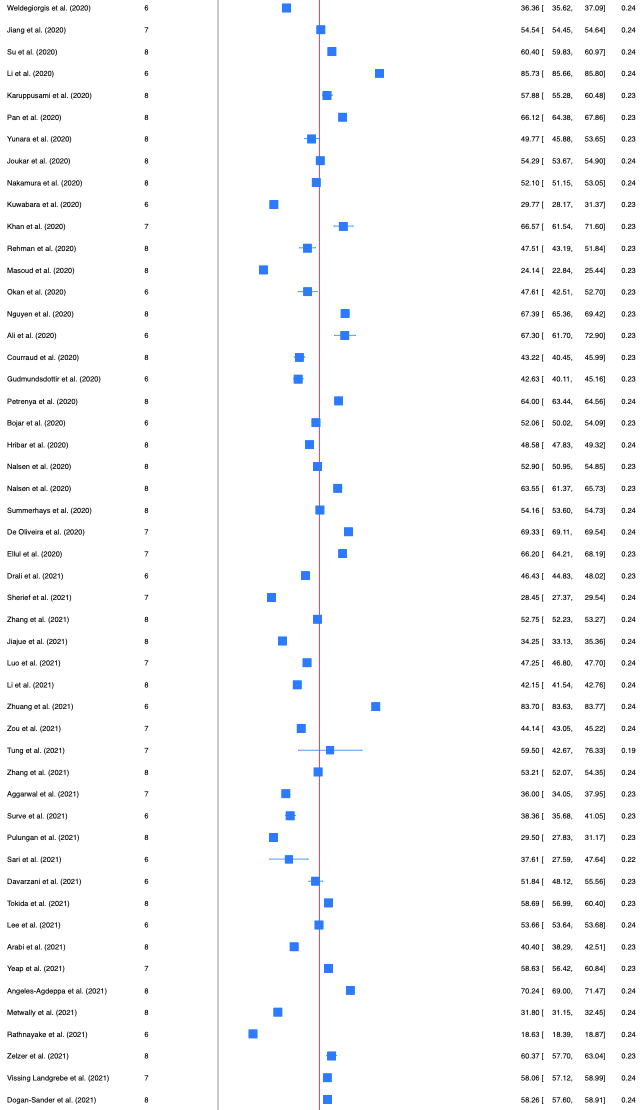


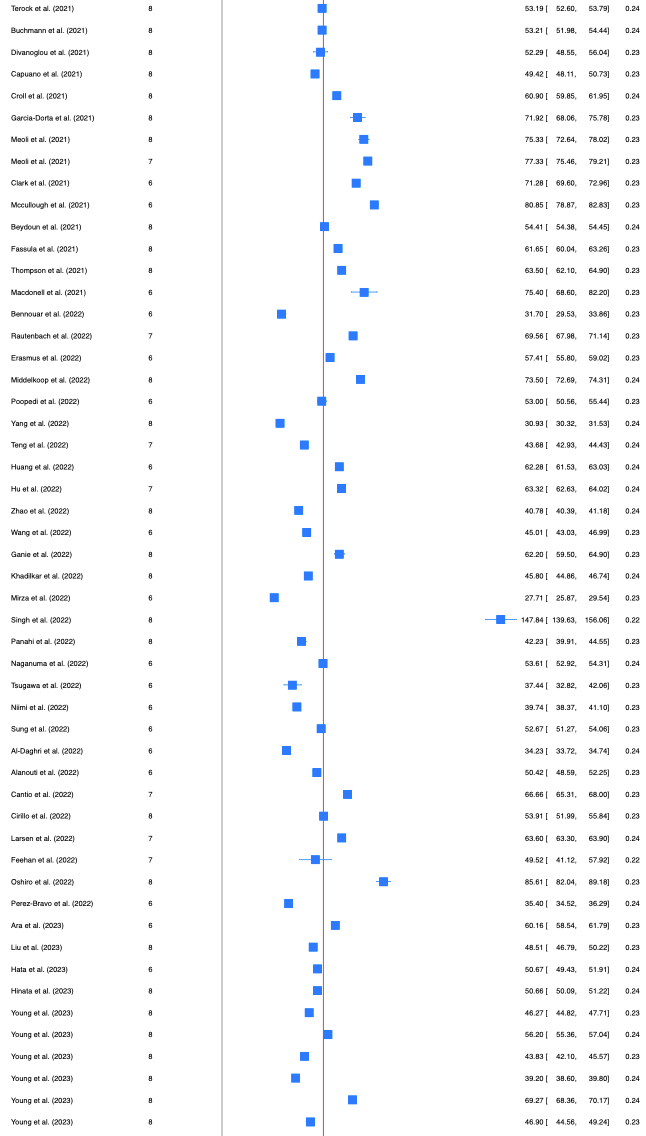


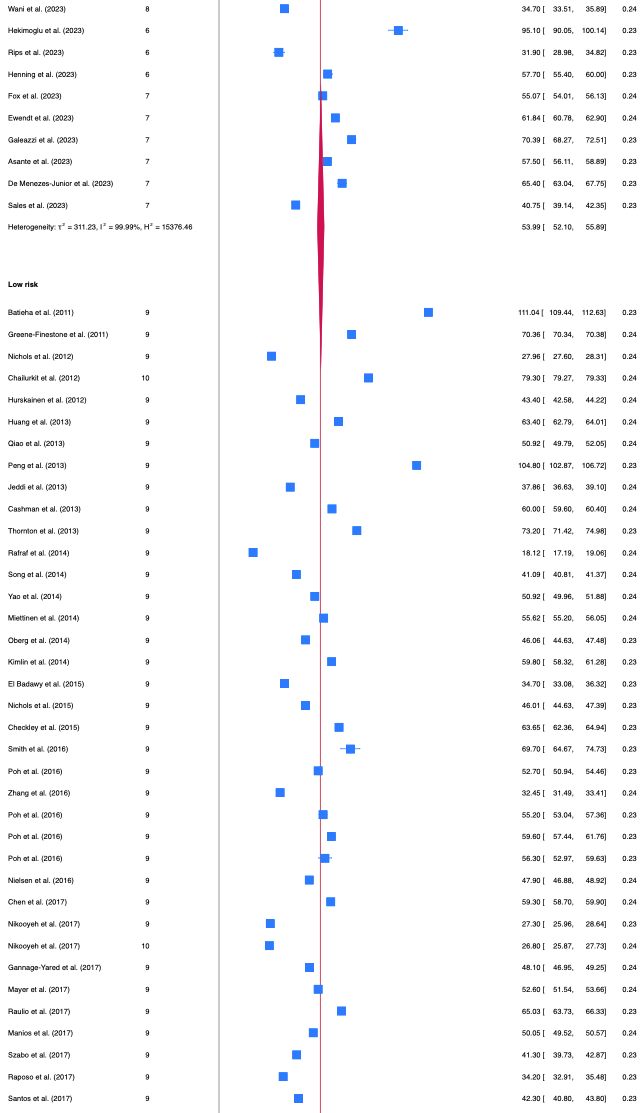


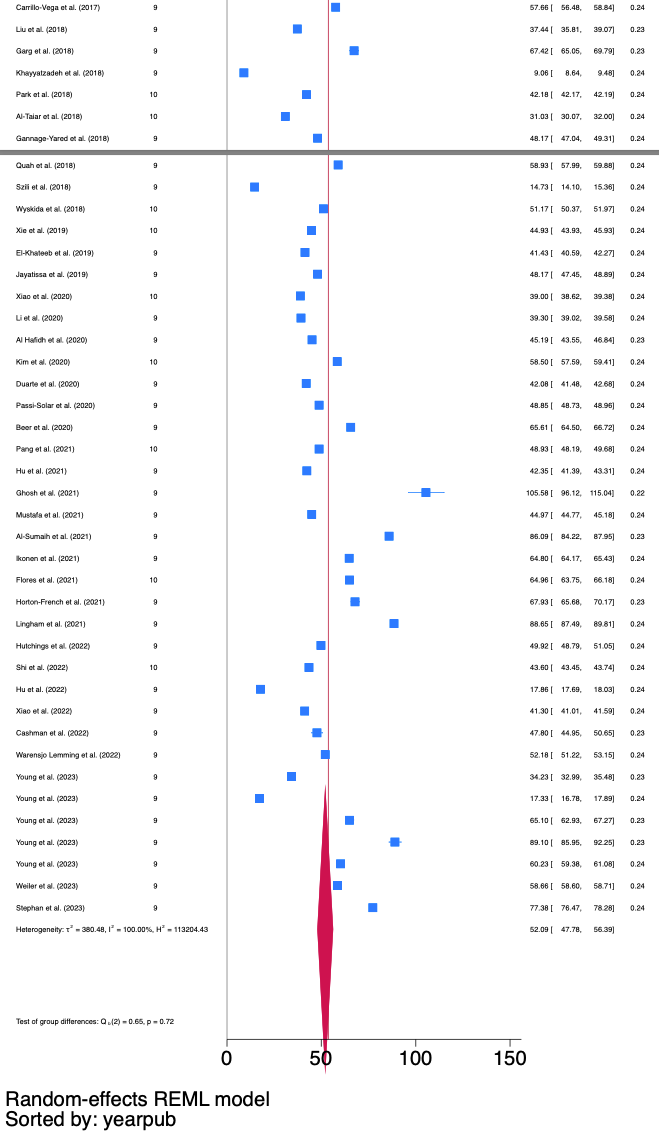


# Supplementary Figure 13: Cumulative* meta-analysis of mean circulating 25(OH)D concentration stratified by mid-point (2012) of all blood draw years


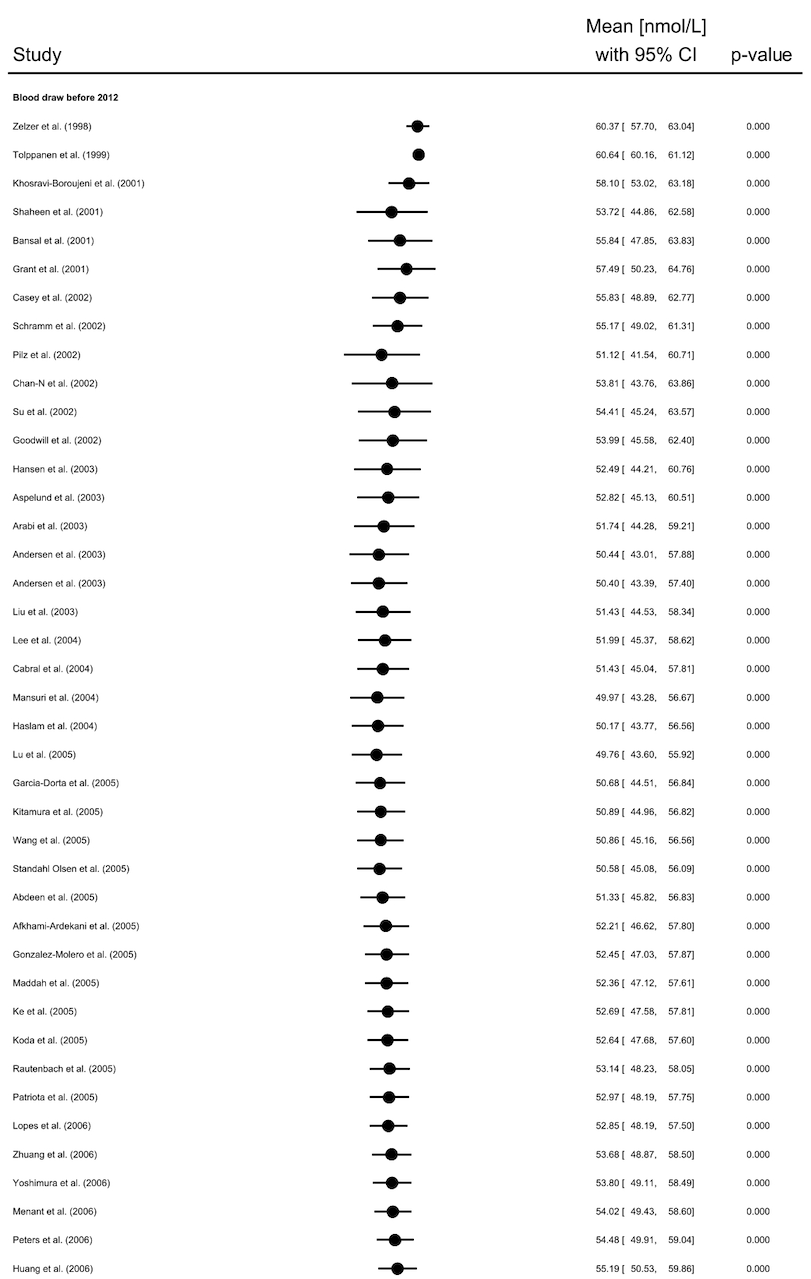


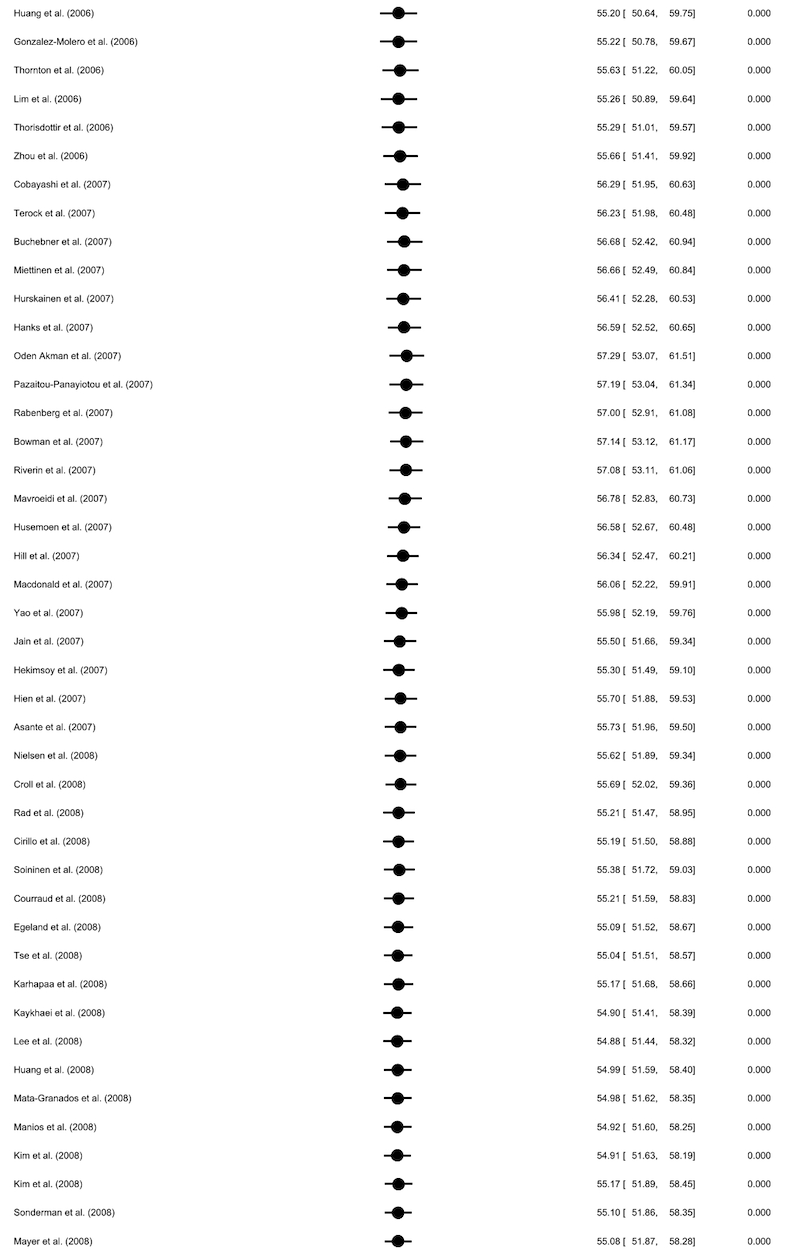

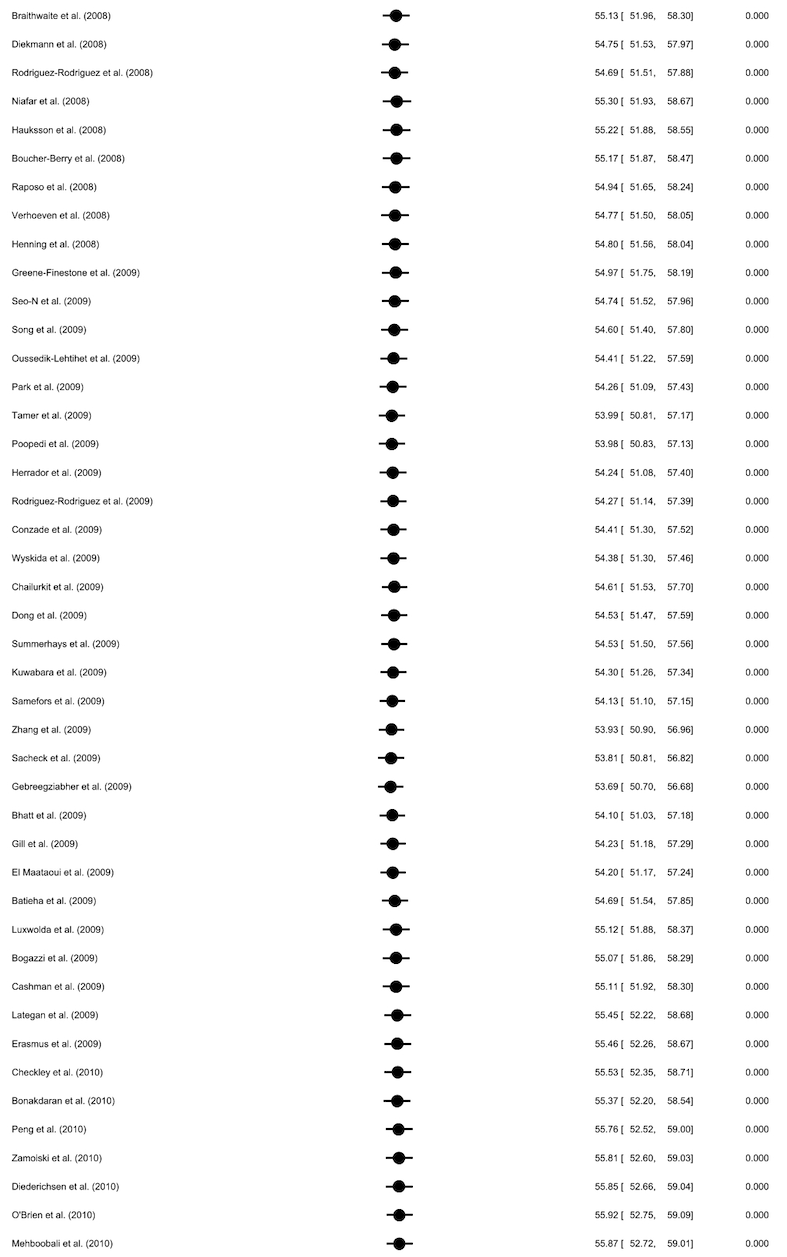


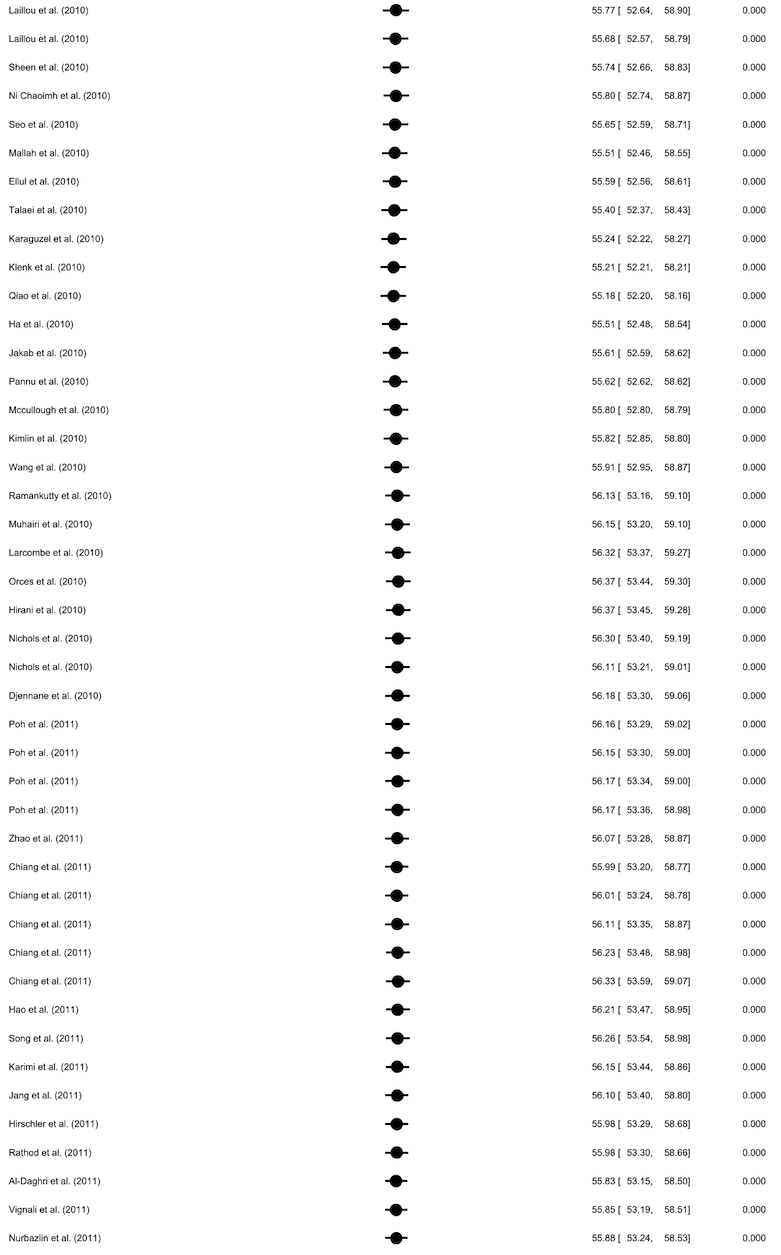


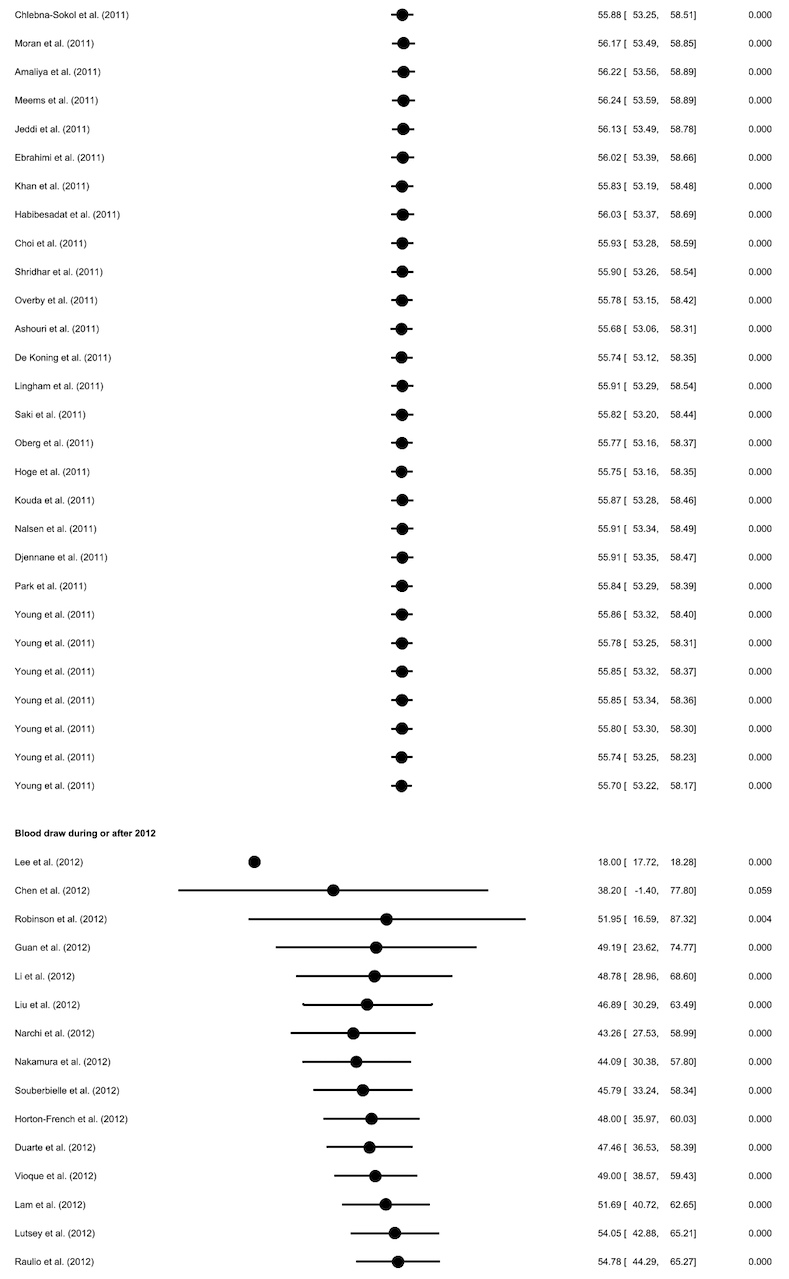

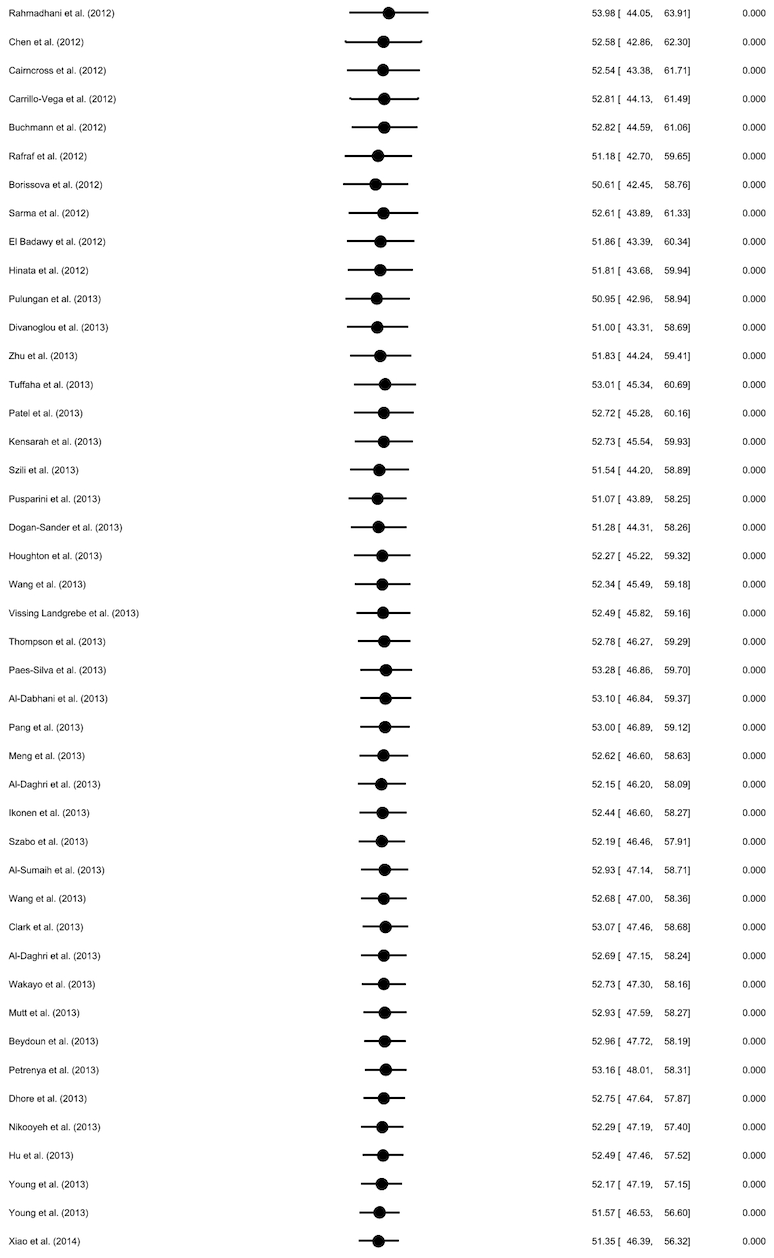


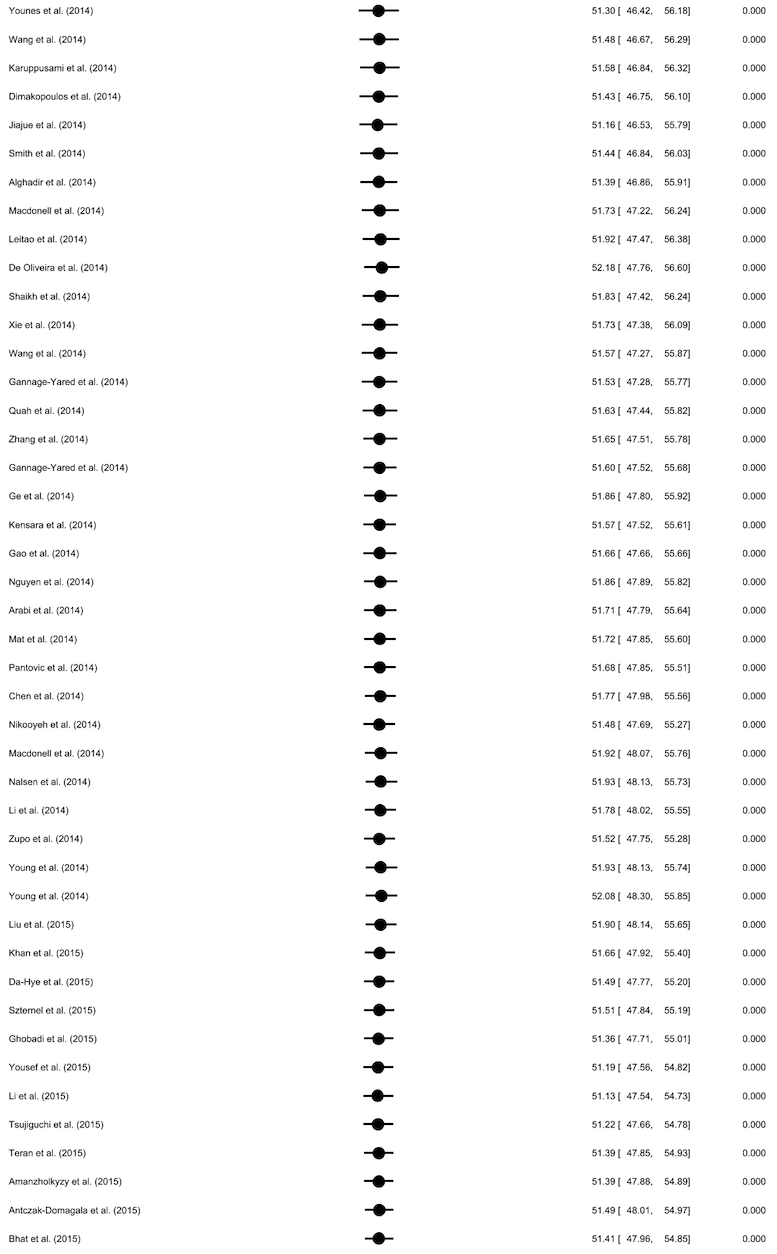

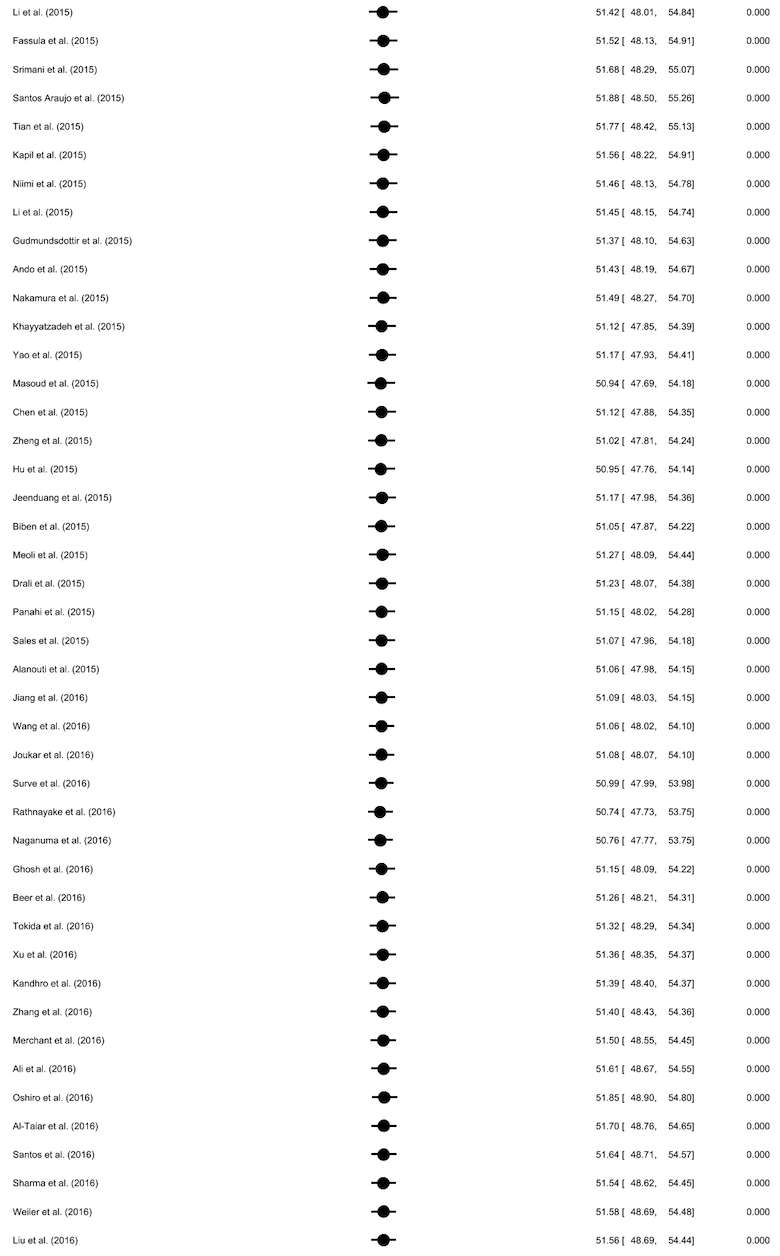

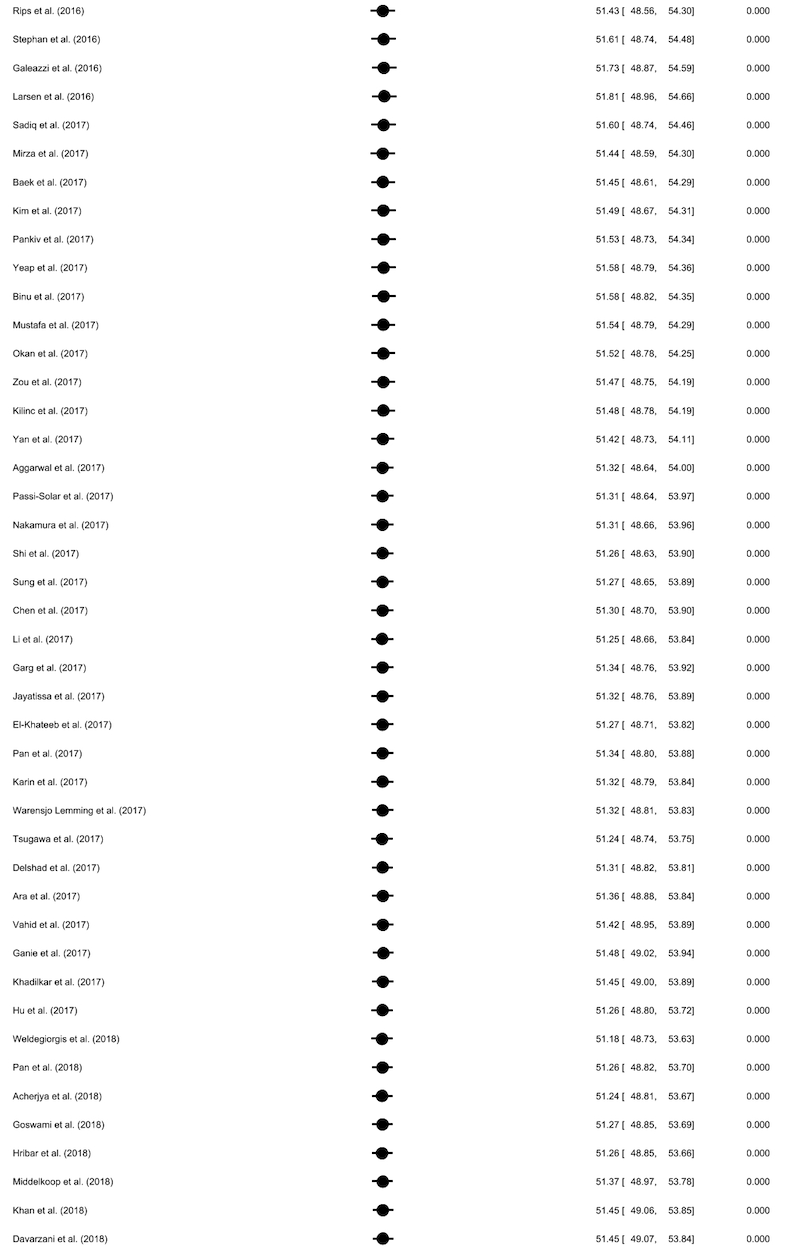


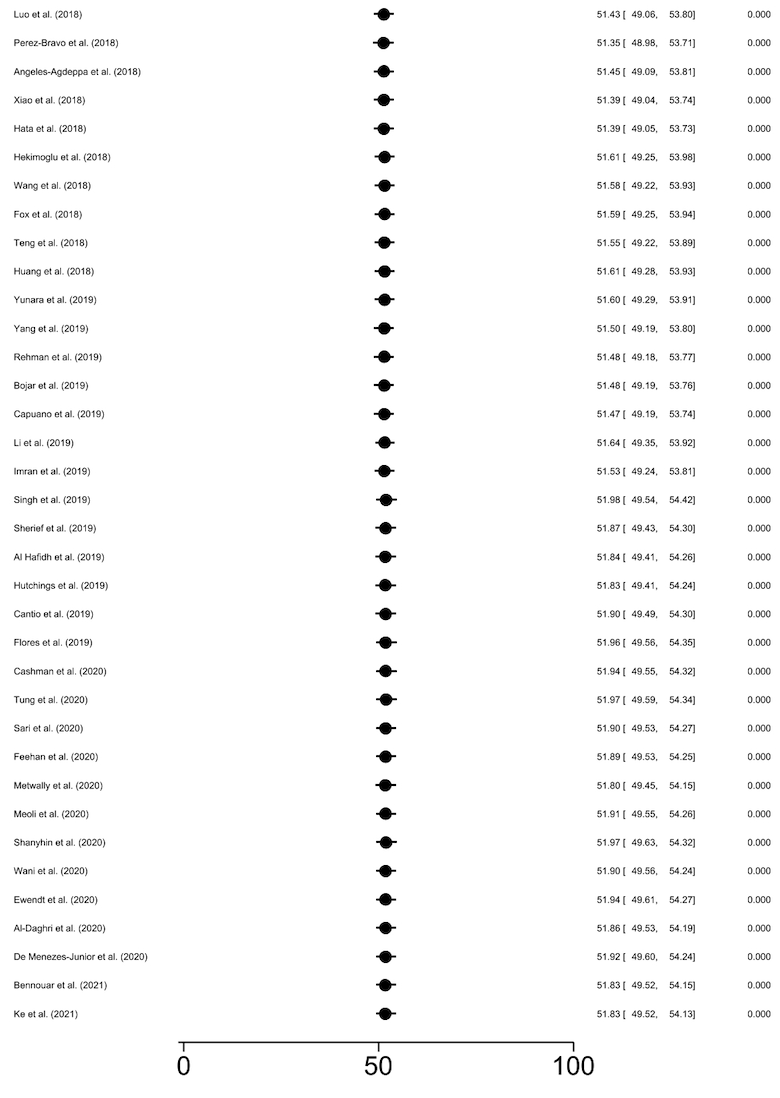


25(OH)D, 25-hydroxyvitamin D; CI, confidence interval

*Based on year of blood collection (single year or mid-point for studies conducted across multiple years)

# Supplementary Figure 14a: Forest plot for pooled prevalence estimate of circulating 25-hydroxyvitamin D concentration <30 nmol/L according to commonly reported thresholds by continent


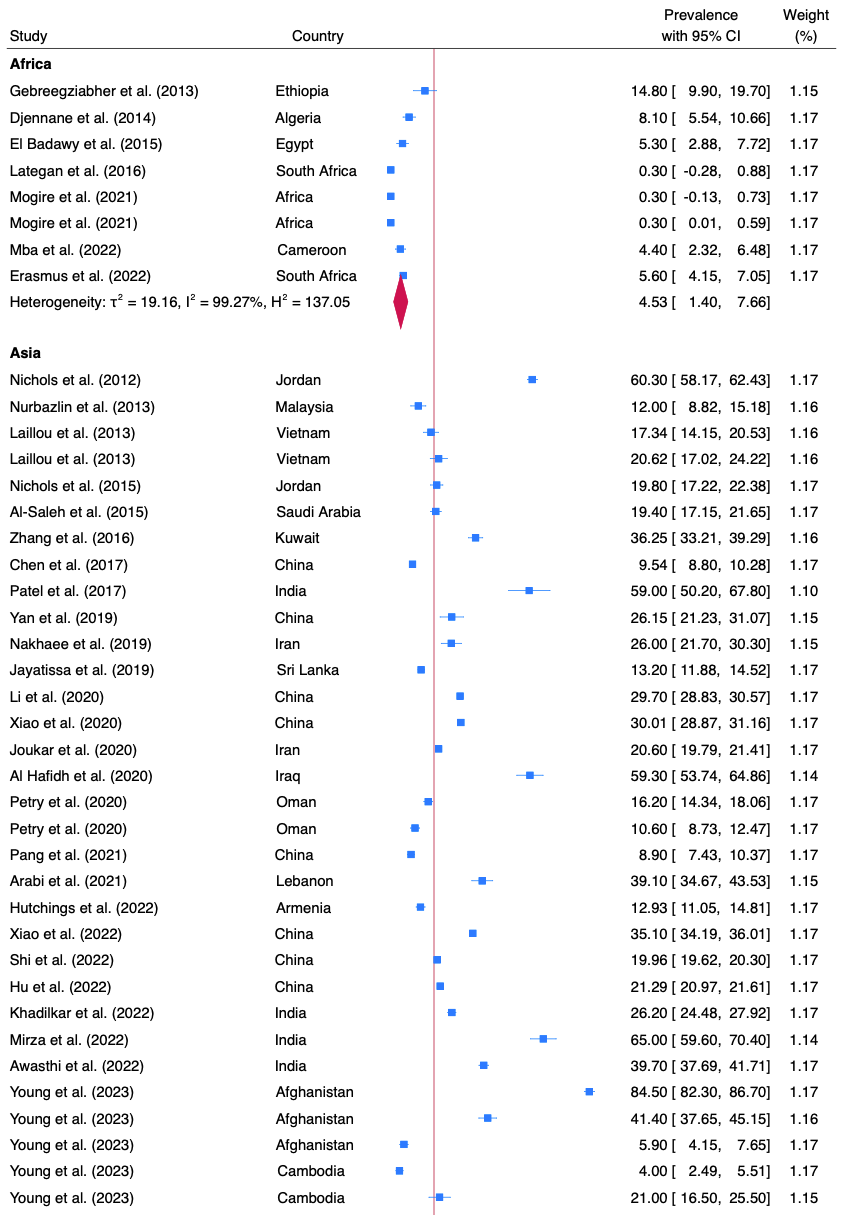


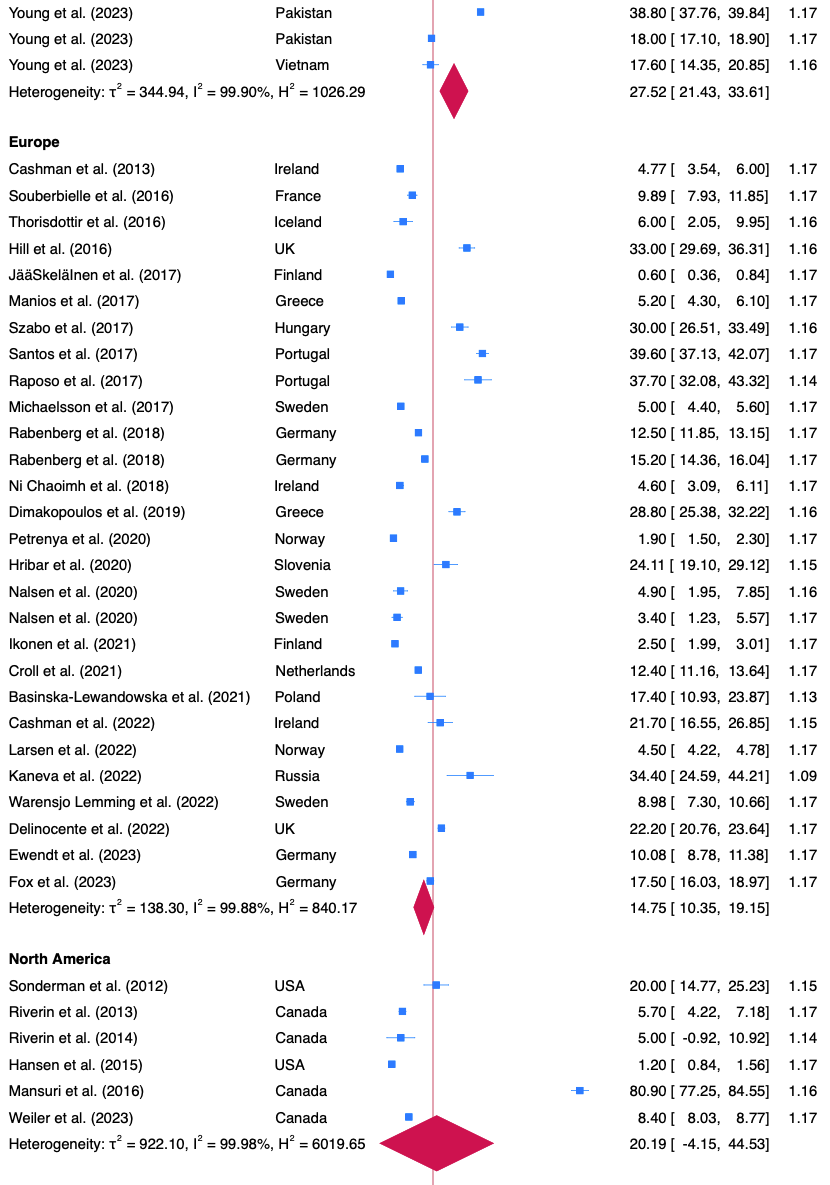

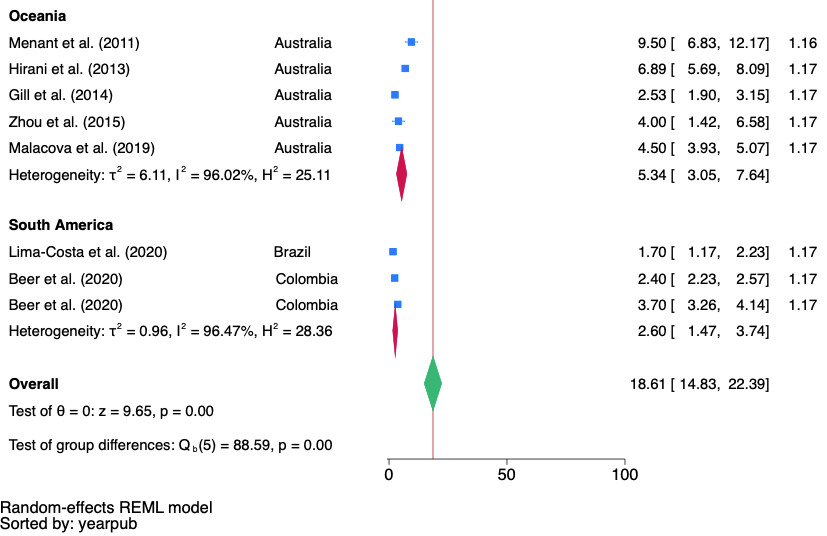


# Supplementary Figure 14b: Forest plot for pooled prevalence estimate of circulating 25-hydroxyvitamin D concentration <50 nmol/L according to commonly reported thresholds by continent


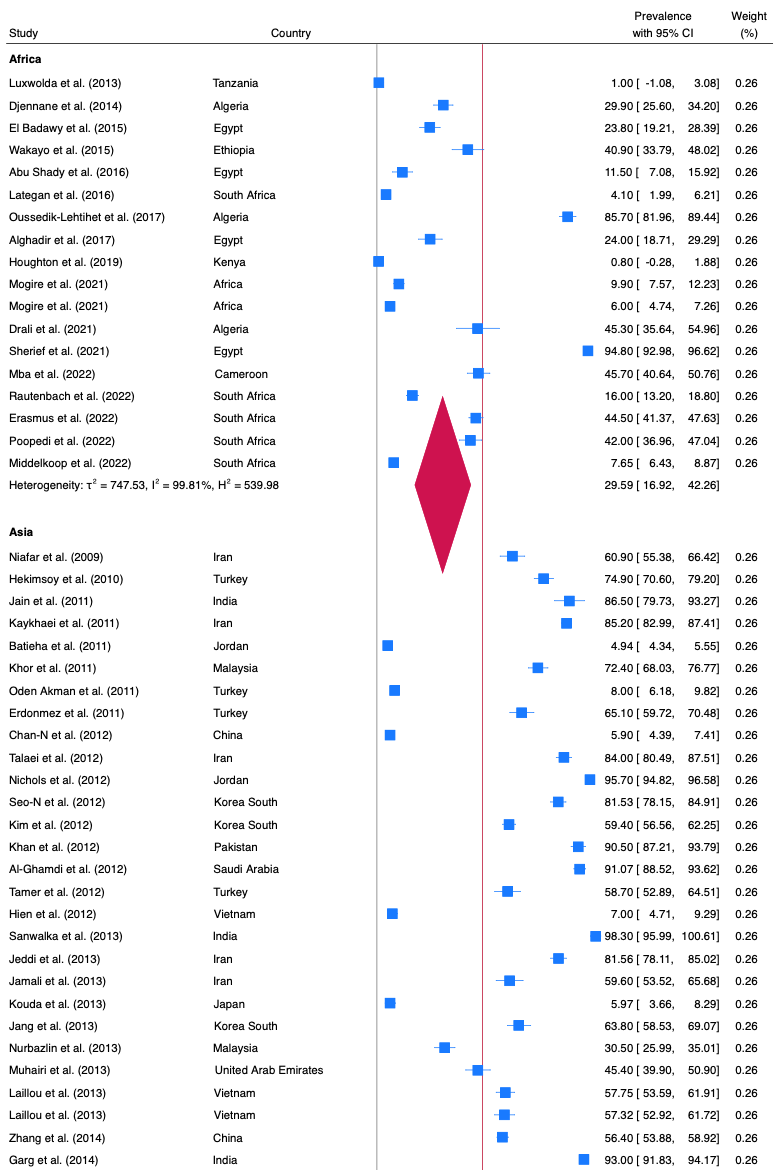


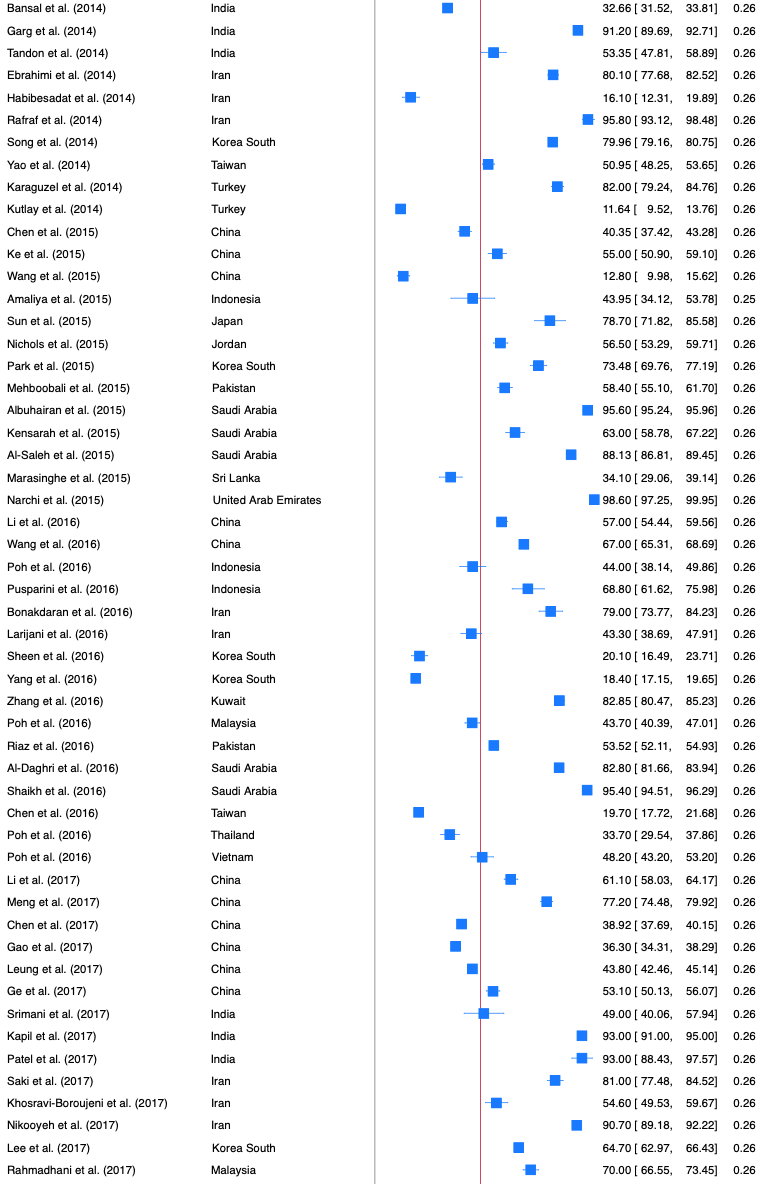

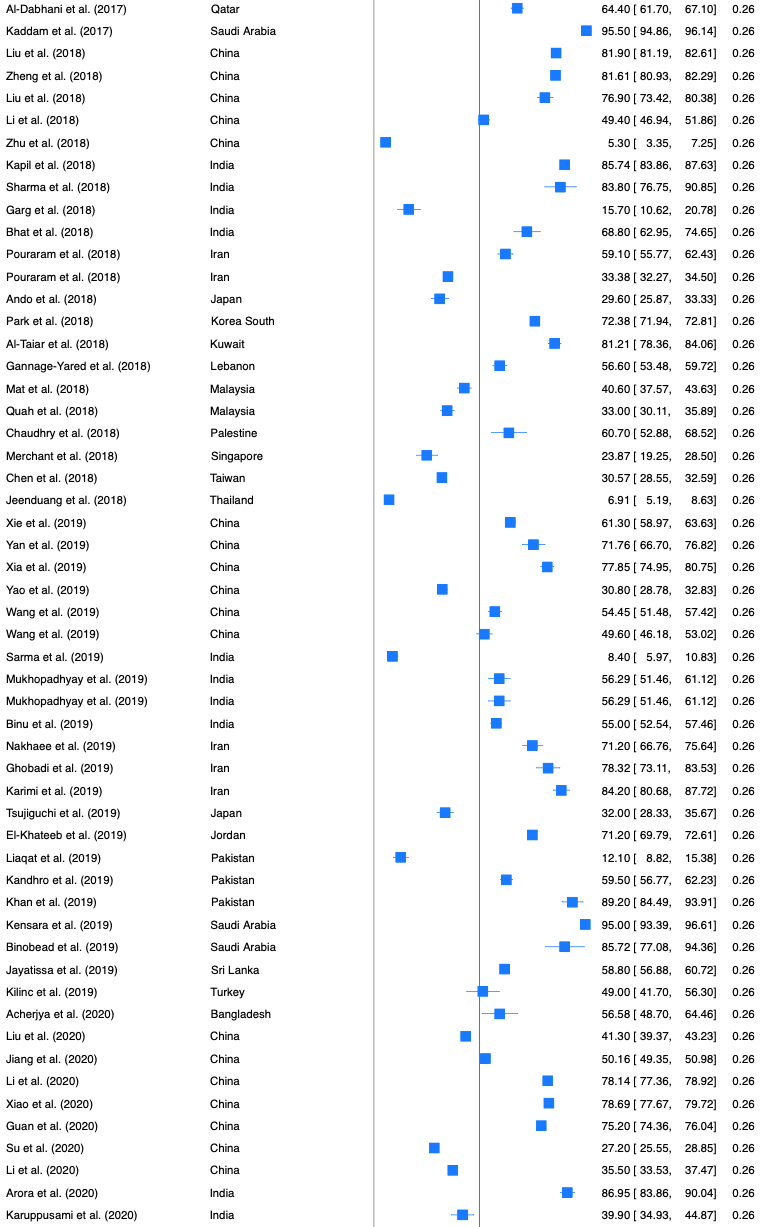


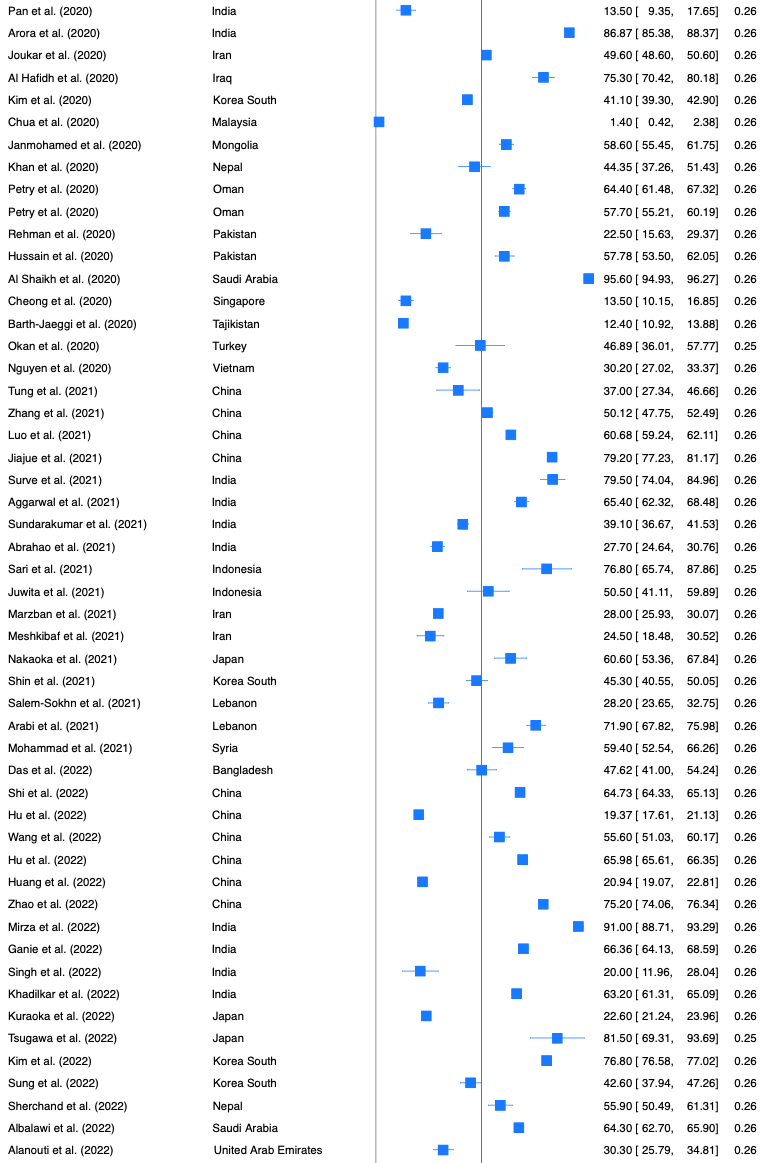


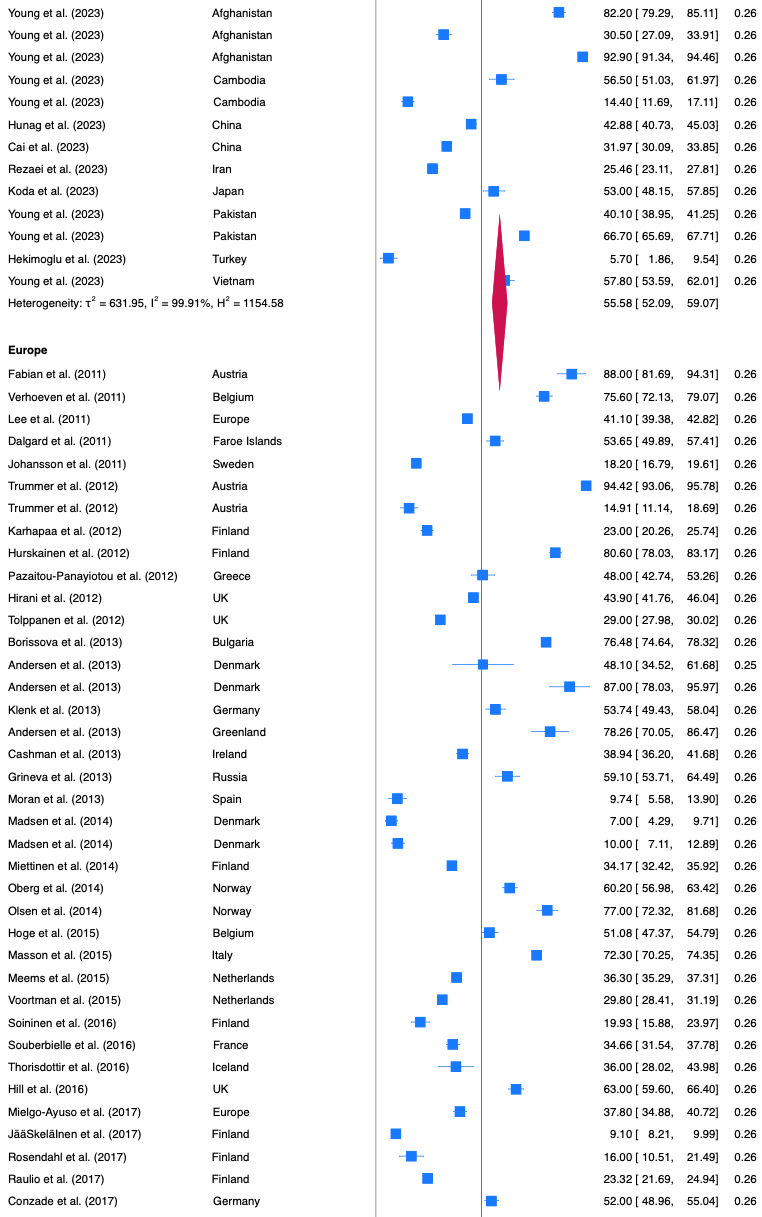

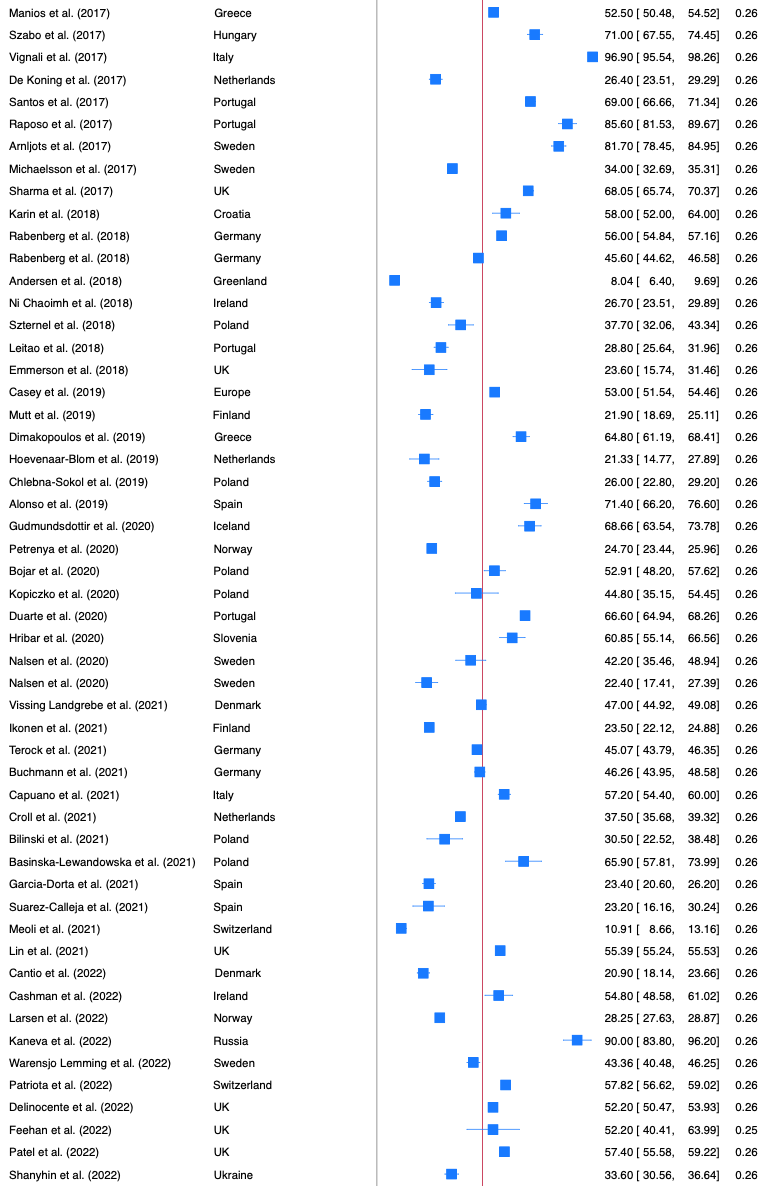

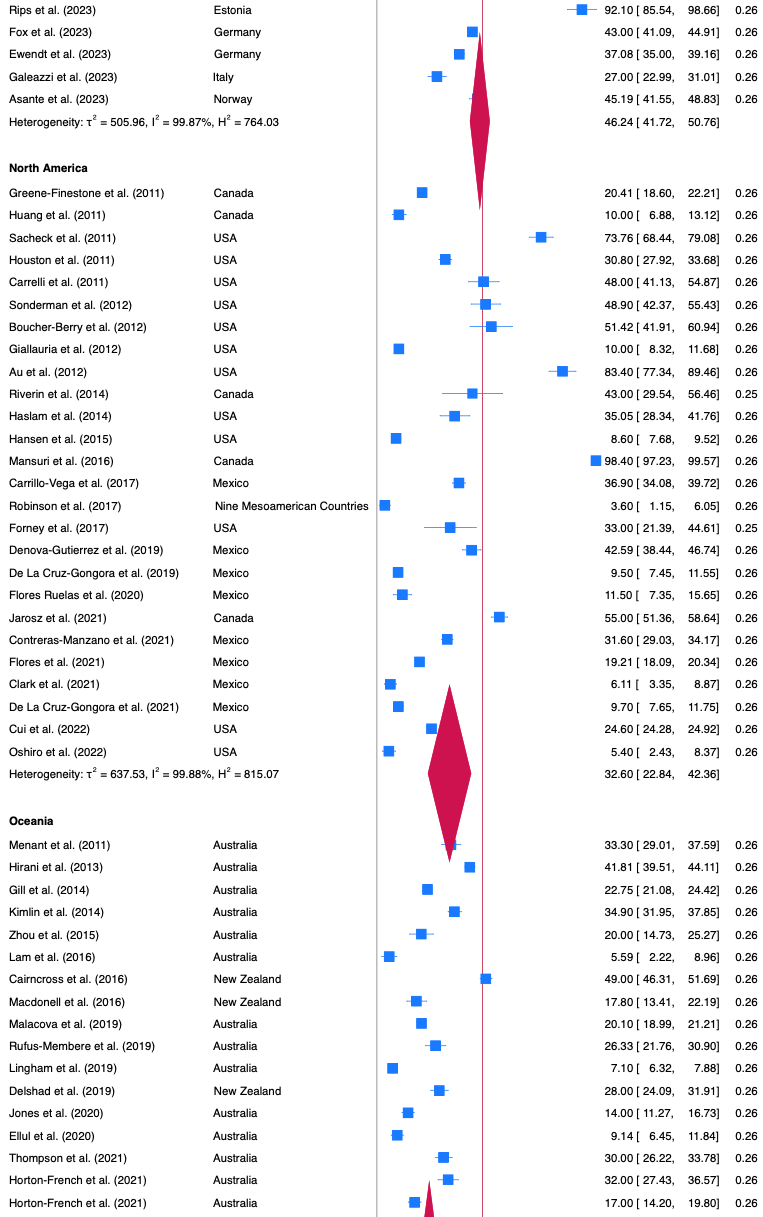


# Supplementary Figure 15a: Forest plot for pooled prevalence estimate of circulating 25-hydroxyvitamin D concentration <30 nmol/L by country

# Supplementary Figure 15b: Forest plot for pooled prevalence estimate of circulating 25-hydroxyvitamin D concentration <50 nmol/L by country – Africa

# Supplementary Figure 15c: Forest plot for pooled prevalence estimate of circulating 25-hydroxyvitamin D concentration <50 nmol/L by country – Asia

# Supplementary Figure 15d: Forest plot for pooled prevalence estimate of circulating 25-hydroxyvitamin D concentration <50 nmol/L by country – Europe

# Supplementary Figure 15e: Forest plot for pooled prevalence estimate of circulating 25-hydroxyvitamin D concentration <50 nmol/L by country – North America

# Supplementary Figure 15f: Forest plot for pooled prevalence estimate of circulating 25-hydroxyvitamin D concentration <50 nmol/L by country – South America

# Supplementary Figure 15g: Forest plot for pooled prevalence estimate of circulating 25-hydroxyvitamin D concentration <50 nmol/L by country – Oceania

# Supplementary Figure 16a: Forest plot for prevalence estimates <30 nmol/L by high (≥40^o^) and low (<40^o^) latitude

# Supplementary Figure 16a: Forest plot for prevalence estimates <50 nmol/L by high (≥40^o^) and low (<40^o^) latitude

# Supplementary Figure 17a: Forest plot for prevalence estimates according to commonly reported thresholds by sex_men

# Supplementary Figure 17b: Forest plot for prevalence estimates according to commonly reported thresholds by sex_women

# Supplementary Figure 18a: Forest plot for prevalence estimates <30 nmol/L by adults and children

# Supplementary Figure 18b: Forest plot for prevalence estimates <50 nmol/L by adults and children

# Supplementary Figure 19a: Forest plot for prevalence estimates <30 nmol/L by season

# Supplementary Figure 19b: Forest plot for prevalence estimates <50 nmol/L by season

# Supplementary Figure 20a: Forest plot for prevalence estimates <30 nmol/L by use of certified assay/harmonised data

# Supplementary Figure 20b: Forest plot for prevalence estimates <50 nmol/L by use of certified assay/harmonised data

# Supplementary Figure 21a: Forest plot for prevalence estimates <30 nmol/L by study quality

# Supplementary Figure 21b: Forest plot for prevalence estimates <50 nmol/L by study quality
