# Supplementary material for: A systematic review and meta-analysis of circulating 25-hydroxyvitamin D concentration and vitamin D status worldwide
Source: J Public Health (Oxf). 2025 Jul 13;47(4):e520–9. doi: 10.1093/pubmed/fdaf080 (PMC12670000; doi:10.1093/pubmed/fdaf080)

*Supplementary File 2*

**Title:** A systematic review and meta-analysis of vitamin D status in healthy populations globally

**Authors:** Eleanor Dunlop ^1,2^, Ngoc Minh Pham ^2^, Dong Van Hoang ^2^, Hajar Mazahery ^2^, Belinda Neo ^2^, Jillian Shrapnel ^2^, Aliki Kalmpourtzidou ^3^, Lucinda J Black ^1,2^

**Author affiliations:**

^1^ Deakin University, Geelong, Institute for Physical Activity and Nutrition (IPAN), School of Exercise and Nutrition Sciences. e.dunlop@deakin.edu.au; lucinda.black@deakin.edu.au

^2^ Curtin School of Population Health, Curtin University, Kent Street, Bentley WA 6102, Australia. eleanor.dunlop@curtin.edu.au; minh.n.pham@curtin.edu.au; vandong.hoang@curtin.edu.au; jill.shrapnel@curtin.edu.au; hajar.mazahery@curtin.edu.au; belinda.neo@curtin.edu.au

^3^ Department of Sustainable Food Process, Università Cattolica Del Sacro Cuore, Cremona, Italy. [alikikalb@hotmail.com](mailto:alikikalb@hotmail.com)

Table of Contents

[Supplementary Figure 22a: Forest plot for nationally representative prevalence estimates <30 nmol/L 2](#_Toc189144329)

[Supplementary Figure 22b: Forest plot for nationally representative prevalence estimates <50 nmol/L 3](#_Toc189144330)

[Supplementary Figure 22c: Forest plot for nationally representative prevalence estimates <30 nmol/L by region and country 5](#_Toc189144331)

[Supplementary Figure 22d: Forest plot for nationally representative prevalence estimates <50 nmol/L by region and country 6](#_Toc189144332)

[Supplementary Figure 22e: Forest plot for nationally representative prevalence estimates <30 nmol/L by sex_men 7](#_Toc189144333)

[Supplementary Figure 22f: Forest plot for nationally representative prevalence estimates <50 nmol/L by sex_men 8](#_Toc189144334)

[Supplementary Figure 22g: Forest plot for nationally representative prevalence estimates <30 nmol/L by sex_women 9](#_Toc189144335)

[Supplementary Figure 22h: Forest plot for nationally representative prevalence estimates <50 nmol/L by sex_women 10](#_Toc189144336)

# Supplementary Figure 22a: Forest plot for nationally representative prevalence estimates <30 nmol/L


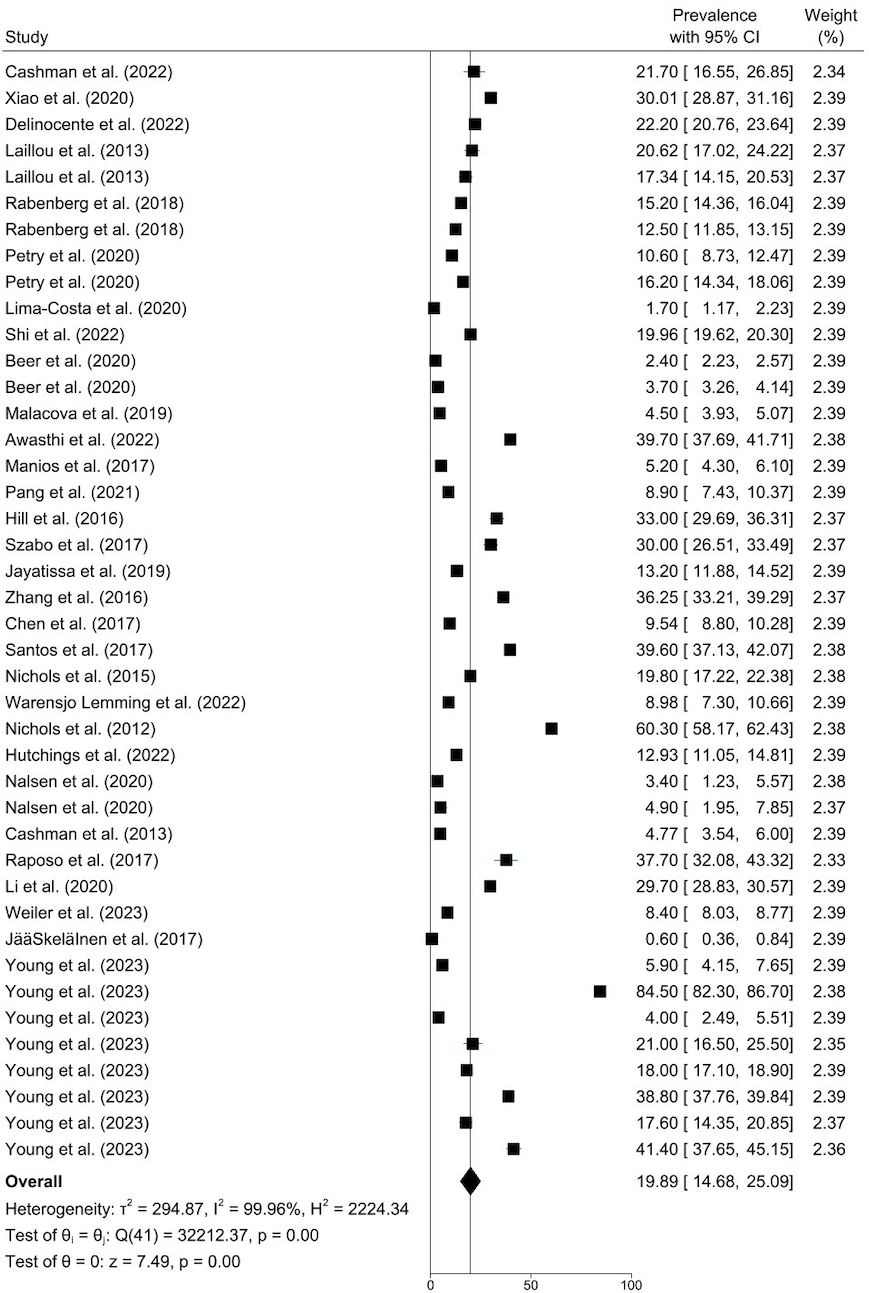


# Supplementary Figure 22b: Forest plot for nationally representative prevalence estimates <50 nmol/L


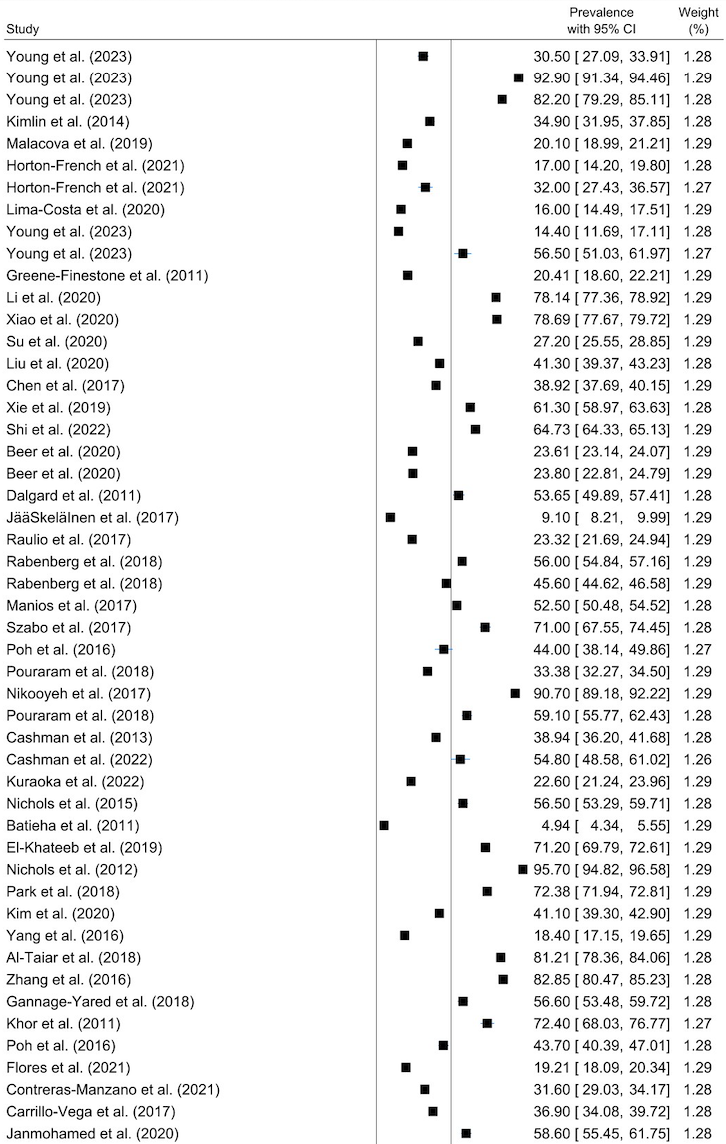


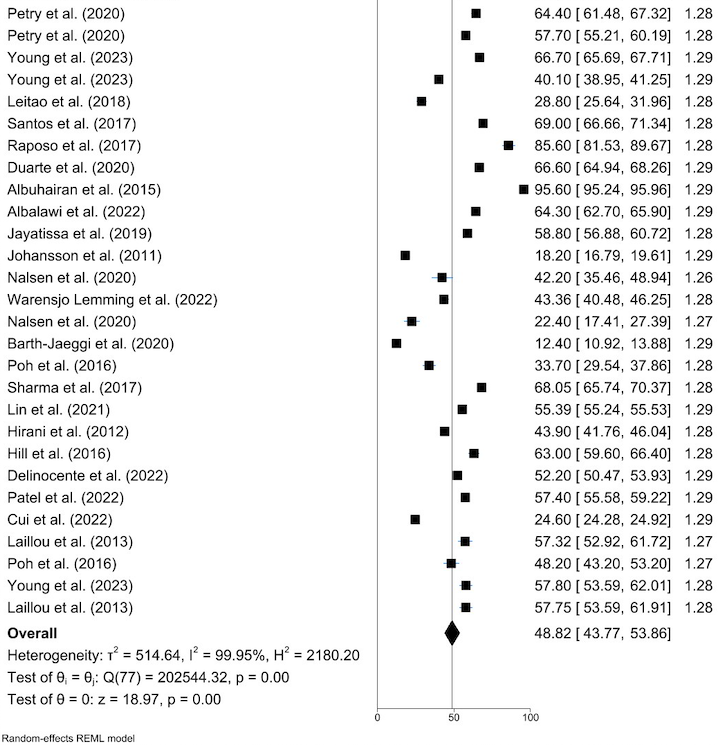


# Supplementary Figure 22c: Forest plot for nationally representative prevalence estimates <30 nmol/L by region and country


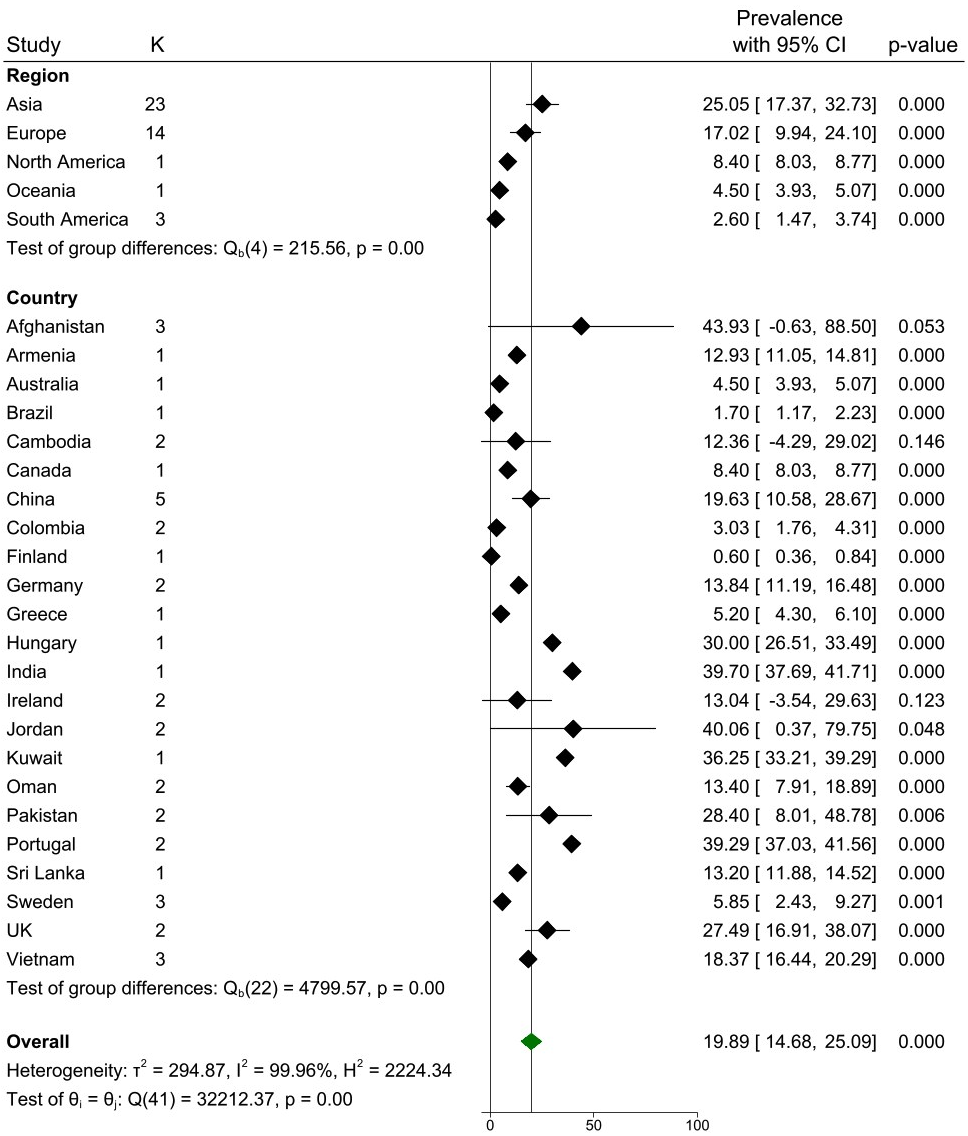


# Supplementary Figure 22d: Forest plot for nationally representative prevalence estimates <50 nmol/L by region and country


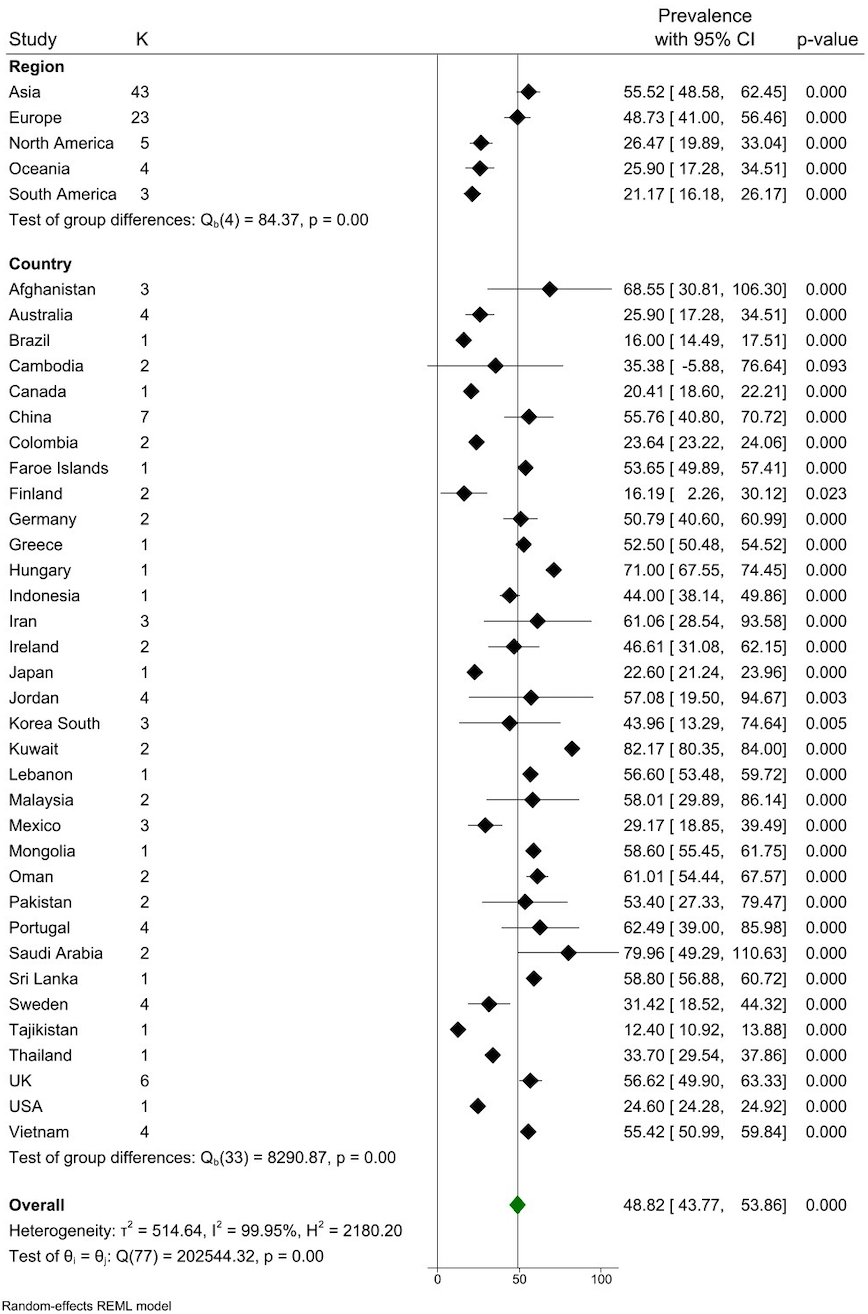


# Supplementary Figure 22e: Forest plot for nationally representative prevalence estimates <30 nmol/L by sex_men


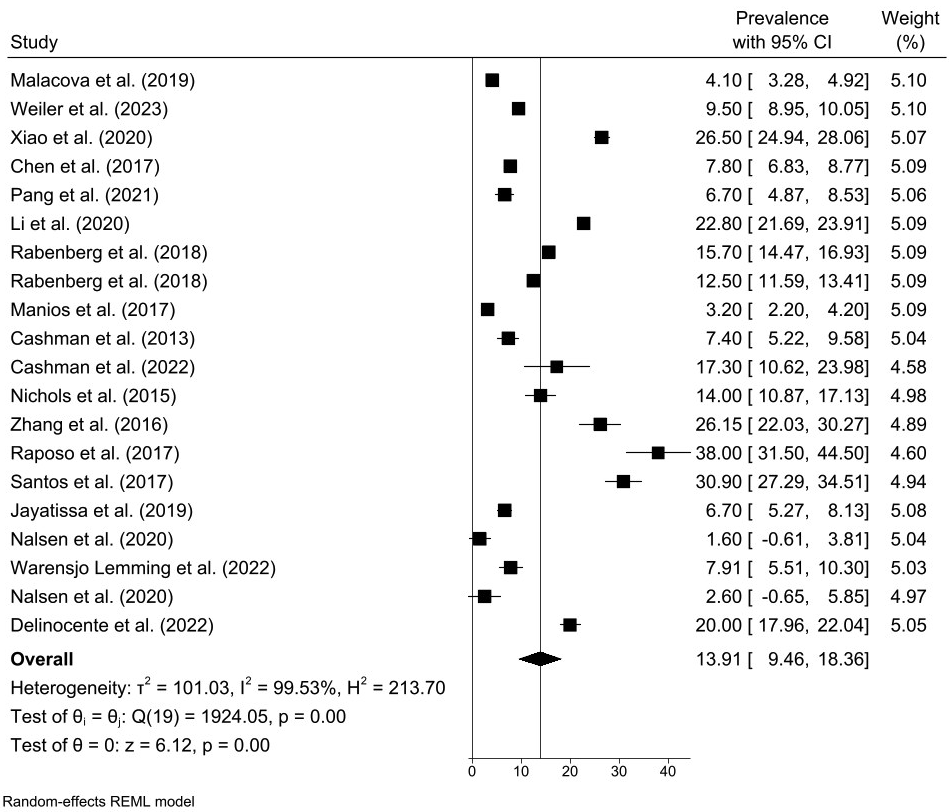


# Supplementary Figure 22f: Forest plot for nationally representative prevalence estimates <50 nmol/L by sex_men


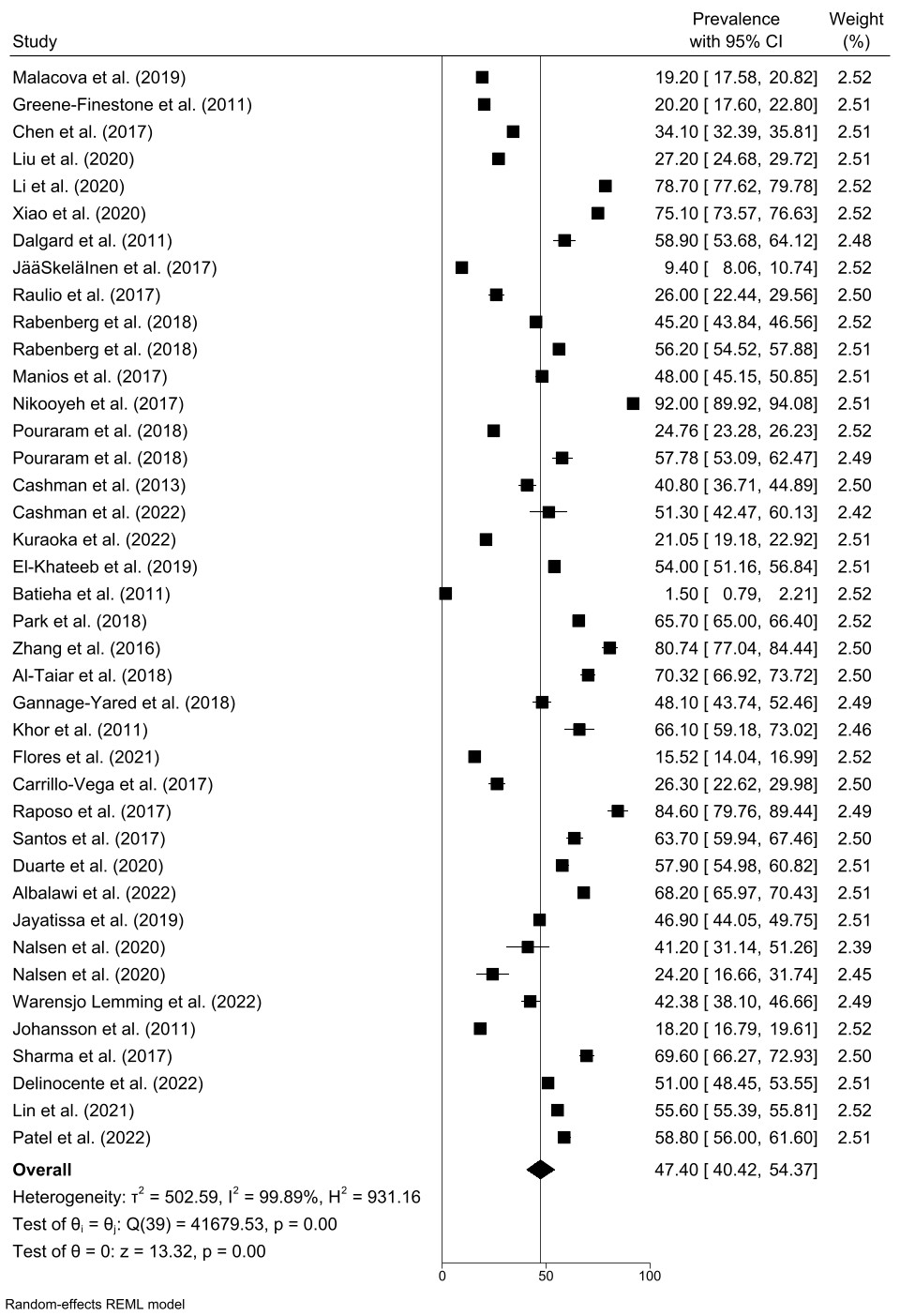


# Supplementary Figure 22g: Forest plot for nationally representative prevalence estimates <30 nmol/L by sex_women


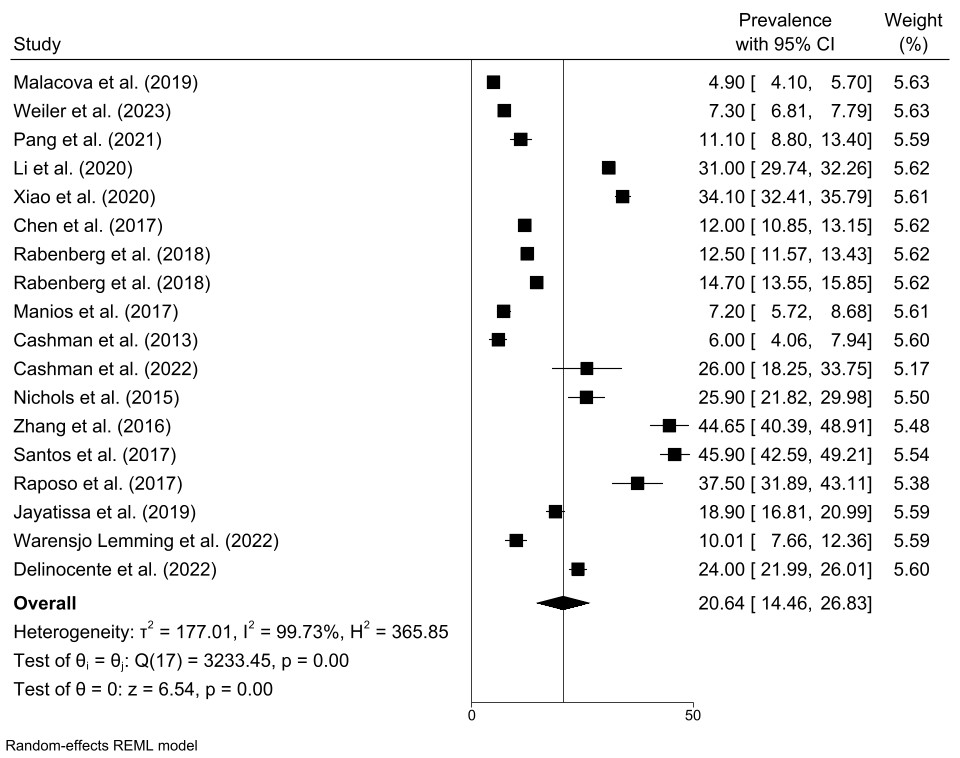


# Supplementary Figure 22h: Forest plot for nationally representative prevalence estimates <50 nmol/L by sex_women


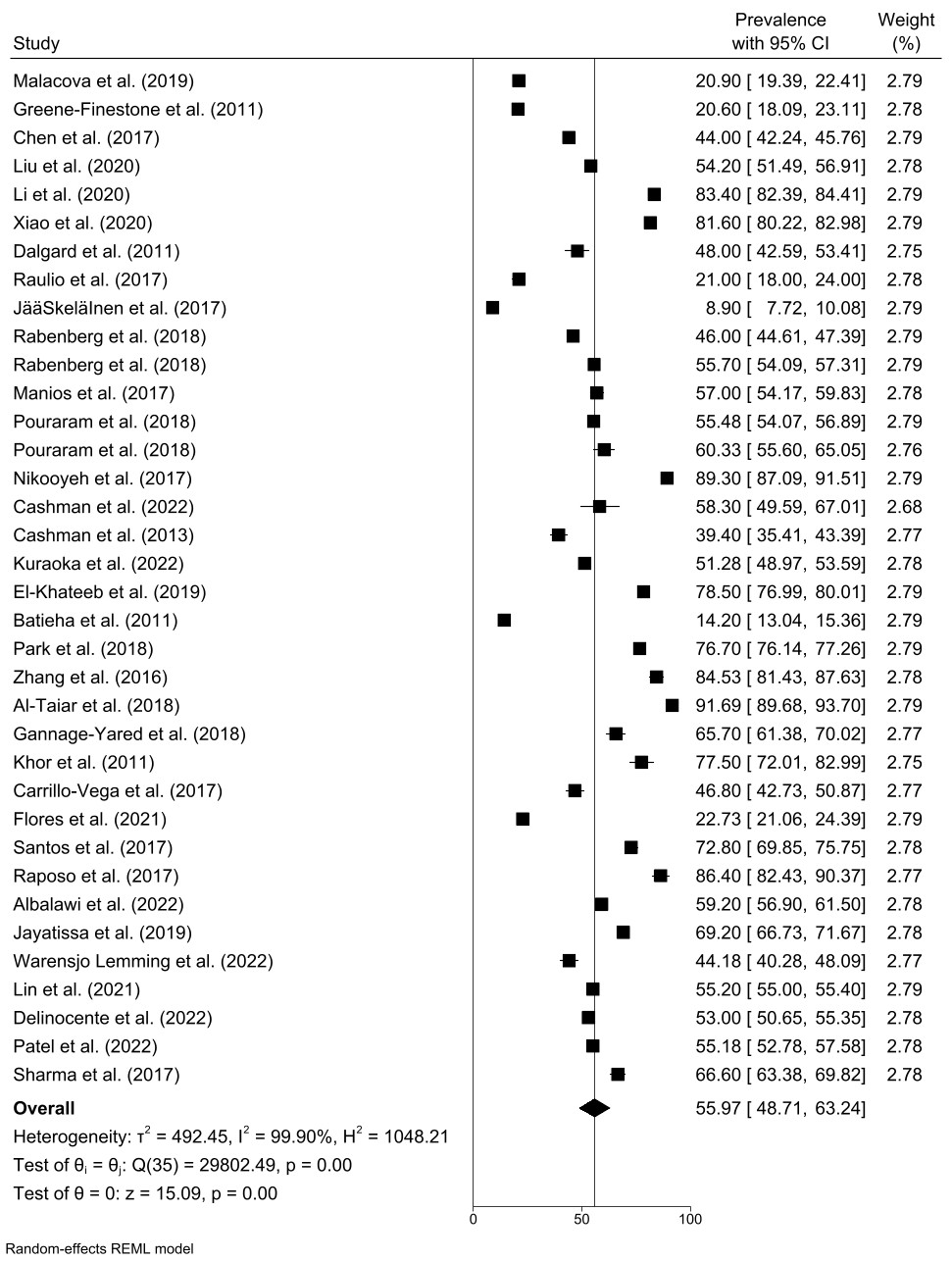

Supplement: Dunlop-et-al-Global-vitamin-D-status-Supplementary-file-2_fdaf080 [file dunlop-et-al-global-vitamin-d-status-supplementary-file-2_fdaf080.docx]
